# Towards the Application of Supramolecular Self-Associating Amphiphiles as Next-Generation Delivery Vehicles

**DOI:** 10.3390/molecules25184126

**Published:** 2020-09-09

**Authors:** Lisa J. White, Jessica E. Boles, Kira L. F. Hilton, Rebecca J. Ellaby, Jennifer R. Hiscock

**Affiliations:** School of Physical Sciences, University of Kent, Canterbury, Kent CT2 7NH, UK; ljw60@kent.ac.uk (L.J.W.); jeb72@kent.ac.uk (J.E.B.); klfh2@kent.ac.uk (K.L.F.H.); rje21@kent.ac.uk (R.J.E.)

**Keywords:** hydrogen bond, supramolecular chemistry, amphiphile, drug delivery

## Abstract

Herein, we present a series of supramolecular self-associating amphiphilic (SSA) salts and establish the potential for these molecular constructs to act as next-generation solution-state molecular delivery vehicles. We characterise the self-association of these SSAs, both alone and when co-formulated with a variety of drug(like) competitive guest species. Single crystal X-ray diffraction studies enable the observation of hydrogen-bonded self-association events in the solid state, whilst high resolution mass spectrometry confirms the presence of anionic SSA dimers in the gas-phase. These same anionic SSA dimeric species are also identified within a competitive organic solvent environment (DMSO-*d*_6_/0.5% H_2_O). However, extended self-associated aggregates are observed to form under aqueous conditions (H_2_O/5.0% EtOH) in both the absence and presence of these competitive guest species. Finally, through the completion of these studies, we present a framework to support others in the characterisation of such systems.

## 1. Introduction

Hydrogen bond formation, electrostatic and charge transfer interactions can all be utilised to drive molecular self-association [1,2]. These events inform the resultant aggregated structure, which leads to the formation of novel functional supramolecular systems, including programmable nanostructures [2,3,4], organic frameworks [5], and supramolecular gels [6,7]. Having originally taken inspiration from natural biological systems, supramolecular chemistry has now come full circle, using biological monomeric units to construct self-associative drug delivery vehicles [8]. Specific examples include work by Nilsson and co-workers [9], who have produced an injectable supramolecular hydrogel capable of effective encapsulation and in vivo delivery of the anti-inflammatory drug diclofenac. Additionally, Yang and co-workers have used peptide amphiphiles to increase the efficacy of chemotherapeutic drugs, cisplatin and 10-hydroxycamptothecine (HCPT) against the cancer cell line A549/DDP [10]. These works highlight the potential of self-associative systems for drug delivery/increasing therapeutic efficacy, achieved through a fundamental understanding of the additive guest species influence on a self-associated system.

The thio/urea motif has been used extensively in the construction of low molecular weight, neutral, hydrogen bond donating organic receptors for the selective coordination of anionic guest species [11]. However, the development of molecular units that contain both a hydrogen bond donating receptor cavity covalently linked to an anion, is a relatively new development in this area of chemistry. One of the first examples of this molecular unit was developed by Gale and co-workers in 2016 [12]. Here, this group produced a series of low molecular weight, urea-based receptors that contain a boronate functionality adjacent to a hydrogen bond donating cavity. This construct was used to enable the selective coordination of neutral phosphate molecules, over competitive anionic guest species in polar organic solvent systems. However, Faustino and co-workers were one of the first groups of researchers to explore the incorporation of the urea-spacer-anion group into the structure of amphiphilic monomers [13]. Here it is hypothesised that the presence of intermolecular hydrogen-bonded urea-anion self-association events are responsible for the comparative decrease in critical micelle concentration (CMC) [14]. Additionally, this type of construct has also been used to design a novel class of protonophoric mitochondrial uncouplers, indicating that this type of urea-spacer-anion motif shows some potential for development towards next-generation therapeutic agents [15].

**Figure 1:**
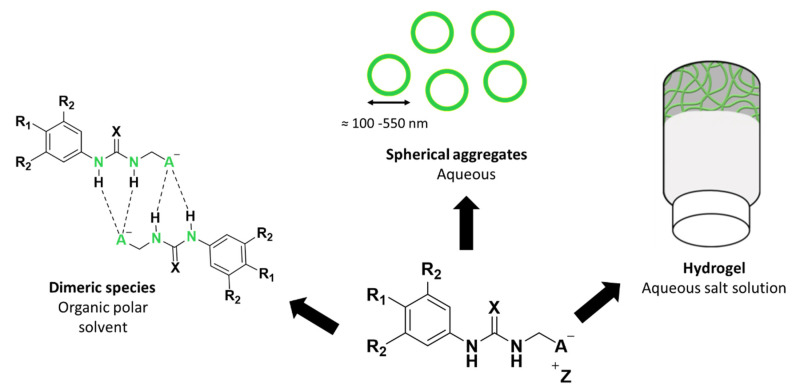
The general chemical structure of a self-associating amphiphilic (SSA) and self-associated SSA species adopted in different solution state environments. R = any group; X = S or O; A^−^ = sulfonate or carboxylate; Z^+^ = counter cation.

Our own work in this area has led to the development of a novel class of supramolecular self-associating amphiphilic salts (SSAs). To date, a library of >50 SSAs and structurally related compounds have been synthesized. The self-associative properties of this compound library and any resultant aggregate structure have also been characterised, using a variety of complementary experimental techniques (Figure 1) [16,17,18,19,20,21,22,23]. A series of solid state, single crystal X-ray diffraction studies have shown the intermolecular binding mode adopted by the anionic component of an SSA to be cation dependent [17]. Here the presence of the weakly coordinating tetrabutylammonium (TBA) counter cation was found to result in the formation of thio/urea-anion dimers however, the formation of thio/urea-thio/urea stacks were induced by the presence of a strongly coordinating counter cation such as sodium or potassium. Whereby, a moderately competitive counter cation such as the pyridinium ion, resulted in the formation of thio/urea-anionic tapes. Additionally, solution state studies have shown the anionic component of an SSA to also produce hydrogen-bonded dimers in a competitive organic solvent system (DMSO-*d*_6_). However, in an aqueous environment, the SSA monomeric units have been shown to form spherical aggregates, with hydrodynamic diameters of c.a. 100–550 nm in size [18,20] and/or hydrogels [23].

**Figure 2:**
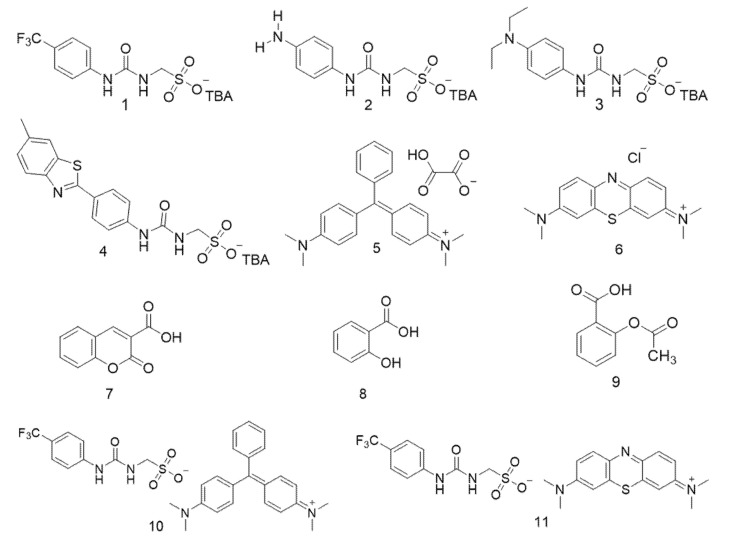
Chemical structures of SSAs **1**–**4**, co-formulant agents **5**–**9** and control SSAs **10**–**11**.

Interestingly, examples from this SSA library have been shown to act as antimicrobial agents against both clinically relevant model Gram-positive (methicillin resistant *Staphylococcus aureus*—MRSA) and Gram-negative (*Escherichia coli*) bacteria [21,22,23]. Within the scope of our studies we have also hypothesised that SSA antimicrobial activity is related to both molecular self-association [23] and selective SSA:phospholipid complexation [24]. However, as SSAs have been shown to arrive at the microbial membrane as self-associated spherical aggregates, we believe this class of compound also has the potential to be developed as next-generation drug delivery vehicles. To investigate this, SSAs (**1**–**4**) were synthesised and co-formulated with various competitive molecular species (**5**–**9**) to produce co-formulations **a**–**j** as shown in Figure 2 and Table 1. Two control SSAs (**10** and **11**) were also synthesised to understand the effects of competitive molecular species addition to the anionic component of **1**. Herein, we will explore the self-associative properties of these novel co-formulations in the solid state, gas phase, and solution state to gain an understanding of the resultant self-association events at the molecular level.

Unlike the anionic component of SSAs **1** and **4**, the molecular design of **2** and **3** may allow for the incorporation of anionic drug/drug-like molecules into these systems via protonation of the terminal amines. Coumarin (**7**) was selected for the ability to deprotonate and form zwitterionic versions of co-formulations **e** and **f**, exploring the presence of a competitive anionic species on molecular self-association. The non-steroidal anti-inflammatory drug (NSAID) aspirin (**9**) and the derivative, salicylic acid (**8**) were also chosen as co-formulants. Compounds **8** and **9** were selected because despite effective and widespread use, the associated gastrointestinal and cardiovascular side effects of these drugs have led to the development of topical formulations and drug incorporation into carriers to reduce toxicity [25,26]. To investigate the potential for SSAs to act in this capacity, these drugs have been incorporated with SSAs **2** and **3** (co-formulations **g**–**j**).

Finally, SSA **4** was used to incorporate two dyes, malachite green (**5**) and methylene blue (**6**), co-formulations **a**–**d**. The dyes selected contain both competitive cationic and anionic guest species (chloride and oxalate respectively), whilst the presence of aromaticity mimics that of many drug molecules. To enable us to understand the potential of SSAs to act as molecular delivery vehicles, it is fundamental we first elucidate the effects of additional competitive guest species on SSA self-association events.

## 2. Results and Discussion

**Figure 3:**
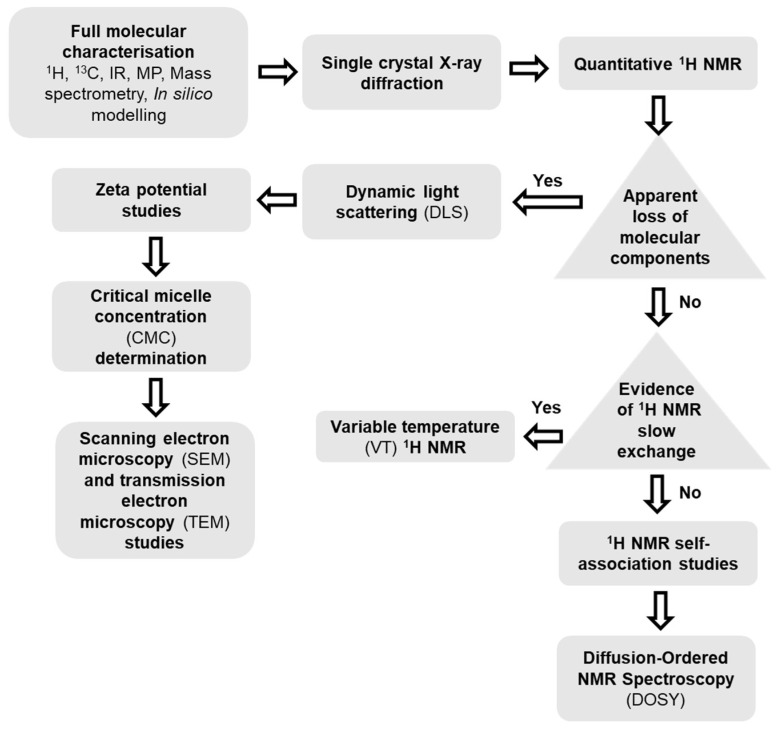
A schematic representation of the techniques used for the characterisation of SSA systems, presented as a flow chart. Square = action; Triangle = decision.

Due to the complex nature of SSA self-association events, and now with the inherent additional complexities of adding competitive species to an SSA system, we have developed an effective multi-component experimental approach to enable characterisation of these complex mixtures. The SSA characterisation process we have developed is shown in Figure 3. Within this flow chart we have included mention of scanning/transmission electron microscopy (SEM/TEM) studies however, these characterisation methods are not discussed further within the scope of this work as SSA self-associated spherical aggregates have previously been shown not to survive SEM/TEM sample preparation methods [16].

In the gas phase, high resolution mass spectrometry experiments allow the observation of any self-associated SSA anion. Single crystal X-ray diffraction allows us to observe molecular self-association events in the solid state. However, although self-associated molecular species are easily identified within the gas phase or solid state using a single technique, the complexities of the solution state self-association events require a combination of complementary techniques. Quantitative ^1^H NMR defines the presence of any larger self-associated species that cannot be observed by solution state NMR. However, self-associated species that are visible using this instrumentation may be further characterised through ^1^H NMR dilution studies (to enable the calculation of association constants) and diffusion ordered spectroscopy (DOSY). For those self-associated structures which are too large to be observed using solution state NMR, we apply a combination of dynamic light scattering (DLS), zeta potential, and critical micelle concentration (CMC) determination to characterise those self-associated species present.

### 2.1. Solid-State Single Crystal X-ray Diffraction Studies

**Figure 4:**
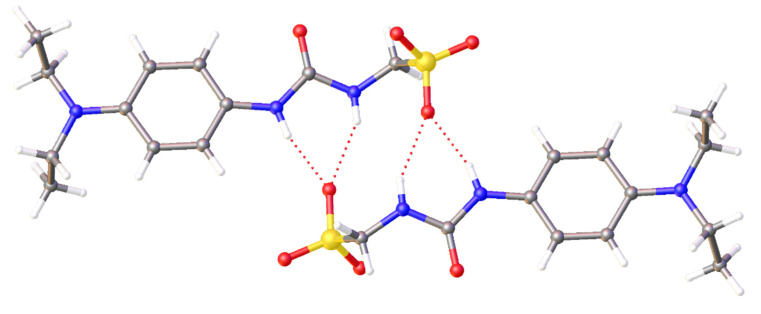
A single crystal X-ray structure of **3**, showing the formation of hydrogen-bonded urea-anion dimers. Here, the tetrabutylammonium (TBA) counter cation has been omitted for clarity. Grey = carbon, blue = nitrogen, red = oxygen, yellow = sulfur, white = hydrogen, red dashed lines = hydrogen bonds. (CCDC–1997432).

To investigate the effects of competitive agent addition upon SSA self-association, a series of single crystal X-ray diffraction studies were undertaken. Figure 4 shows the crystal structure of SSA **3**. As observed previously [17], the presence of the non-competitive TBA counter cation results in urea-anion dimer formation. This dimer is stabilised through the formation of four hydrogen bonds, one from each of the urea NH hydrogen bond donating groups to a different oxygen atom contained within the sulfonate functionality. The interior angle of dimerization was calculated to be 180.00°.

**Figure 5:**
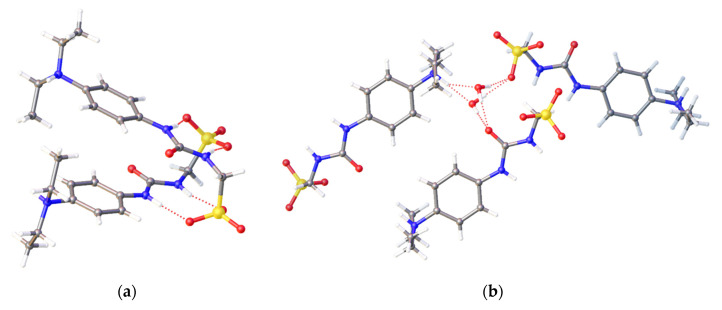
Single crystal X-ray structure obtained by slow evaporation of a methanol solution containing co-formulation **h**. Here SSA **3** is obtained as a zwitterion that is involved in a complex hydrogen-bonded network. This network includes (**a**) urea-anion dimer formation and; (**b**) hydrogen-bonded network formed between the water molecules through the urea, anion, and amine functionalities of the SSA. Grey = carbon, blue = nitrogen, red = oxygen, yellow = sulfur, white = hydrogen, red dashed lines = hydrogen bonds. (CCDC–1997433).

A single crystal obtained from a methanol solution of co-formulation **h** was found to contain the zwitterion of SSA **3**, as shown in Figure 5a. In this instance, the presence of salicylic acid (**11**) within the crystallisation liquor has resulted in the protonation of the tertiary amine group contained within **3** to produce a zwitterion. This zwitterion was also found to dimerize (Figure 5a), through the formation of four urea-anion hydrogen bonds in an identical hydrogen bonding mode to that of the anionic unit (Figure 4). However, we do observe a change in interior angle of dimerization from 180.00° to 13.18°. Figure 5b illustrates additional hydrogen bonding interactions between SSA zwitterions and a disordered water molecule. Here, the water molecule acts as a hydrogen bond donor/acceptor bridging unit between the anionic component of the zwitterion, the oxygen atom of the urea functionality and the protonated hydrogen bond donating tertiary amine group. This provides evidence that when protonated the previously amphiphilic molecule has increased in polarity, causing the coordination of water molecules both at the head and tail end of the SSA.

**Figure 6:**
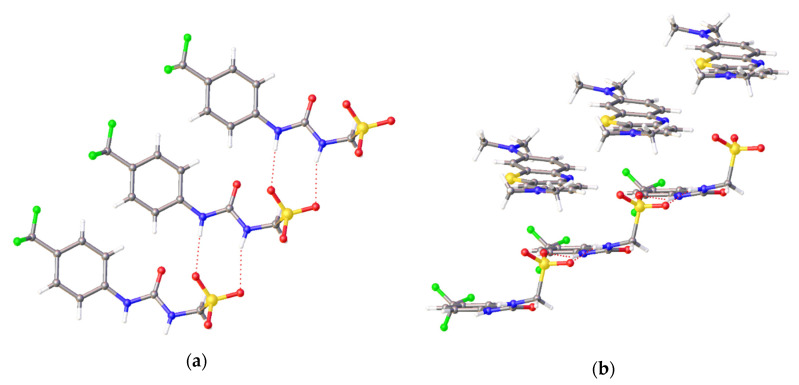
Single crystal X-ray structure of **11**, (**a**) highlights the self-associative urea-sulfonate hydrogen bonds formed between the anionic components of this SSA, while (**b**) highlights alternating anionic and cationic sheets also present within this crystal structure. Atomic disorder has been omitted for clarity and the methylene blue counter cation has been omitted in Figure 6**a**. Grey = carbon, blue = nitrogen, red = oxygen, yellow = sulfur, green = fluorine, white = hydrogen, red dashed lines = hydrogen bonds. (CCDC–1997431).

A crystal structure obtained for compound **11** highlights the effects of an additive cationic, aromatic drug-like species on SSA self-association events. We have previously shown that the presence of a TBA counter-cation results in the formation of thio/urea-anion dimers with an internal angle of dimerization = 22.60° [17]. Where the TBA cation is replaced with the methylene blue cation, the anionic component no longer dimerizes, but instead forms a hydrogen-bonded tape (Figure 6a). The molecular self-association is stabilised through the formation of two intermolecular hydrogen bonds between the urea and sulfonate functionalities. Additionally, the methylene blue cation stacks in sheets above and below those of the SSA anionic components, shown in Figure 6b.

### 2.2. Gas-Phase Self-Association

Previously published high resolution electrospray ionisation (ESI) mass spectrometry studies have shown the anionic portion of most SSAs to exist as dimeric species [16,17,18,19,20,21,22]. Data previously obtained for SSAs **1** [16], **2** [21] and **4** [18] confirmed the presence of SSA anionic dimers under these experimental conditions. Supporting these previous observations, the presence of anionic SSA dimers were also observed for SSAs **3**, **10** and **11**, see Table 2.

### 2.3. Solution State Self-Association

SSA self-association events are known to be dependent on solvent environment [18]. Quantitative ^1^H NMR techniques are used to provide initial evidence for the presence of smaller (dimers) and/or larger SSA aggregates in solution. While also quantifying the proportion of those molecular substituents involved in the construction of higher order species. These larger aggregates adopt solid-like properties and are therefore no longer observable using solution state NMR techniques, so appear to ‘disappear’ or are ‘lost’ from the NMR spectra. Comparative integration against an internal standard allows for the percentage of a molecular component apparently ‘lost’ from a solution to be quantified. It is worth noting that this experimental technique does not confirm the absence of any self-associated species at a concentration below the limit of detection of the NMR spectrometer used. Table 3 shows the results of data analysis performed for solutions **1**–**11** and co-formulations **a**–**j** in: (i) DMSO-*d*_6_, standardised with 1.0% DCM and (ii) D_2_O standardised with 5.0% ethanol. The majority of SSA studies conducted to date show that in DMSO-*d*_6_ based solutions many of those self-associated structures formed are anionic dimers, at concentrations up to 112 mM [18,20,22]. This trend was also observed for the majority of SSAs and co-formulations discussed herein under the same experimental conditions.

Interestingly, co-formulation **d** showed an apparent ‘loss’ of SSA **4** in the presence of co-formulant **9** in a DMSO-*d*_6_ solution. However, the formation of larger SSA aggregates was not observed in the absence of **9** (SSA **4** only). Furthermore, when comparing the percentage of anionic and cationic components of SSA **4** that form the larger self-associated structures in co-formulation **d**, there is an imbalance; an observed 74% ‘loss’ of anion and 12% ‘loss’ of TBA cation. We therefore hypothesise these larger self-associated constructs incorporate the methylene blue cation in preference to the TBA (**6**). Experiments conducted with **6** only, show that at this same concentration methylene blue is able to form higher ordered species independently. Interestingly, co-formulation **c**, which incorporates methylene blue but with SSA **1**, does not show any evidence for the formation of higher ordered structures under the same experimental conditions. It is therefore plausible to hypothesise that interactions between the extended planar ring systems found in the benzothiazole moiety (SSA **4**) and compound **6** are responsible for the formation of those larger structures in DMSO-*d*_6_ at 112 mM.

Moving into aqueous conditions (5.56 mM), SSAs **1**, **3**, **4**, **10** and **11** all show evidence for the formation of higher-order self-associated aggregates in solution. This same observation was made for all co-formulations (Table 3). Interestingly, the presence of larger self-associated aggregates of SSA **2** were not confirmed by this method. We hypothesise this is due to the presence of hydrophilic moieties at both ends of the SSA anion. Where SSAs **1** and **4** are co-formulated with a secondary species, the proportion of the anionic SSA component incorporated into these larger, higher ordered structures increases. Co-formulations **a**–**d** show 100% of the SSA anion and co-formulant cation to be involved in self-associated aggregation events under aqueous conditions. This leads us to hypothesise that the interactions of the SSA anion and the co-formulant cation are highly favorable, an observation also made within DMSO-*d*_6_. To test this hypothesis, control SSAs **10** and **11** were synthesised and the results of comparative study showed that under the same experimental conditions, 100% of both the anionic and cationic component were found to be involved in extended aggregate formation. Despite the complete ‘loss’ of SSA anion and co-formulant cation from co-formulations **a**–**d**, the ‘loss’ of a proportion of TBA from solution shows the presence of a second salt within these aggregated species. Co-formulation **a** shows a ‘loss’ of 15%, compared to **c** which incorporates nearly double the amount of TBA, at 28%. This observation is attributed to the co-formulant present. However, when comparing the effect of SSA exchange, the presence of SSA **1** (co-formulation **a**) results in the incorporation of 15% of TBA into the extended aggregate structure, compared to SSA **4** (co-formulation **b**), the presence of which results in 37% of the SSA cation to become incorporated within the larger self-associated aggregates produced. 

Unlike SSAs **1** and **4**, SSAs **2** and **3** both have an amino protonatable site appended from the phenyl ring system of the SSA anion. In aqueous conditions, the co-formulation of SSA **3** results in a decrease in the proportion of anionic SSA component incorporated into the extended self-associated structures, whereas the inverse is true for the aqueous co-formulations incorporating SSA **2**. Co-formulations **f** and **h** both contain SSA **3** but different co-formulants, coumarin (**7**) and salicylic acid (**8**). These systems exhibit a very similar ‘loss’ of SSA anionic and cationic components from solution, with a 42% and 44% ‘loss’ for **f** and 41% and 43% ‘loss’ for **h**, when considering the SSA’s anionic and cationic components respectively. However, under analogous experimental conditions, a significantly larger amount of co-formulant **7**, coumarin (81%) has been ‘lost’ from solution compared to co-formulant **8**, salicylic acid (34%). It is hypothesised that in aqueous conditions, SSA **3** forms a spherical aggregate species, similar to that of SSA **4** which has been visualized within the scope of previous studies via fluorescence microscopy [18]. The ‘loss’ of coumarin (**7**) and salicylic acid (**8**) could therefore be a result of encapsulation within the SSA self-associated structure, which now acts as a carrier for these co-formulants.

Co-formulations **g** and **h** both contain co-formulant **8**, salicylic acid but different SSA anionic components (**2** and **3**). In both cases, results from our quantitative ^1^H NMR analysis show 44% of the TBA has become incorporated into these larger self-associated structures. The proportion of salicylic acid to be ‘lost’ from solution however differs. An additional 24% of the salicylic acid is incorporated into larger aggregate species with co-formulation **g** when compared to co-formulation **h**. This increase in salicylic acid incorporation coincides with a further 20% incorporation of the anionic component of SSA **2** into the extended aggregate structures. We therefore hypothesise that the anionic component of SSA **2** has become protonated by a proportion of the salicylic acid (which remains in the bulk solution, now as the TBA salt) creating an SSA zwitterion. This process is analogous to that shown in Figure 5, which creates an aggregate structure capable of absorbing the additional 24% of the salicylic acid as a neutral species. 

Co-formulations **e** and **g** both incorporate SSA **2** but substitute coumarin (**7**) for salicylic acid (**8**). The presence of the coumarin results in an increase in the proportion of TBA to become trapped within the larger self-associated structures, which increases from 44% (co-formulation **g**) to 55% (co-formulation **e**). This coincides with a ≈ 30% increased incorporation of co-formulant **7** (coumarin). The self-associated structures produced from co-formulation **e** exhibit very similar levels of both SSA anionic and cationic component incorporation. Unlike co-formulation **g**, there is a lack of evidence to support the formation and incorporation of the SSA anion as a zwitterion. Considering the pKa of both co-formulants [27,28], we would expect zwitterions of SSA **2** to be produced in the presence of the coumarin as we had previously observed with salicylic acid. However, we believe the increased hydrophobicity of the co-formulant present, coumarin (**7**) compared to salicylic acid (**8**), drives a greater proportion (11%) of SSA **2** into forming self-associated aggregate structures.

As illustrated in Figure 3, those solutions which exhibited no apparent ‘loss’ of molecular components in DMSO-*d*_6_ are taken forward for ^1^H NMR self-association constant determination studies. To quantify the strength of the hydrogen-bonded self-association events undertaken by the anionic SSA components, a series of ^1^H NMR dilution studies were conducted in a DMSO-*d*_6_/0.5% H_2_O solution, results of which are shown in Table 4. Self-association constants were derived using BindFit v0.5 [29]. However, these models are limited to fitting one component, one dimensional, homogeneous aggregation events [30]. Here, the ^1^H NMR dilution study data were fitted to both the cooperative equal K (CoEK) model, which assumes the first self-association event is different from all subsequent identical self-association events and the dimerization/equal K (EK) model, where the association constants for all self-associative events are equal [31,32,33]. When comparing the fit of ^1^H NMR dilution data for SSAs **1**–**4** to both the EK and CoEK binding isotherms, lower fitting errors were observed consistently for the (dimerization)EK over the CoEK model. This is consistent with previous observations made for >30 SSAs studied to date [18,20,22].

To confirm the size of the self-associated SSA structures within the ^1^H NMR dilution study environment, and thus support the reporting of the EK dimerization constant, a series of ^1^H NMR DOSY experiments were performed. These studies conducted with SSAs **1**–**4** show the anionic component of these salts to have a hydrodynamic diameter of ≤1.61 nm, therefore this would support (alongside our previous observations with similar systems [18,20,22], gas phase (Table 2) and solid state (Figure 4 andFigure 5) data) the formation of lower-order hydrogen-bonded dimeric species. These studies also show that the SSA anionic component is not strongly coordinated to the TBA counter cation. As seen in Table 5, when SSAs **1**–**4** are co-formulated, small increases in hydrodynamic diameter of the anionic components are observed. The size of these species however is still indicative of lower order self-associated/complex structures. Therefore, these data gathered also support our findings from the fitting of our ^1^H NMR dilution study data.

Proton NMR dilution study data obtained for co-formulations **e**–**j**, show no observable change in chemical shift for any resonances attributed to the co-formulants (**7**–**9**). Therefore, there is no evidence that these co-formulants are involved in the self-association events of any SSA present under these experimental conditions. This conclusion is corroborated by the results of ^1^H NMR DOSY studies, which also show no evidence of co-formulant: SSA complexation. In addition, as with the SSA alone, these ^1^H NMR dilution study data obtained for the SSA anionic component of co-formulations **e**–**j** were found to fit the (dimerization)EK binding isotherm in preference to the CoEK binding isotherm, further supporting the presence of SSA anionic dimers. The derived association constants (K_dim_) for co-formulations **e**–**g** and **j** are lower than those obtained for the corresponding SSA, providing evidence that the addition of a secondary species into the matrix has weakened the strength of the self-associated complex. However, with co-formulation **h**, the presence of salicylic acid (**8**) causes an increase in the strength of the self-associated complex formed from K_dim_ = 0.6 M^−1^ (SSA **3**) to K_dim_ = 1.2 M^−1^ (co-formulation **h**).

Unlike co-formulations **e-j**, co-formulations **a** and **c** show the anionic component of SSA and the co- formulant to exhibit identical diffusion constant coefficients (Table 5 and ESI Table S4 in supplementary materials). These data further support those observations made from the analysis of quantitative ^1^H NMR studies, which showed evidence of SSA counter cation exchange. In comparison to TBA, both malachite green (**5**) and methylene blue (**6**) are strongly coordinating counter cations. These ^1^H NMR DOSY studies show **5** and **6** to diffuse through the solution at the same rate as the SSA anion, therefore providing evidence of anion:cation coordination. It is because of this observation that those association constants produced through the fitting for ^1^H NMR to the (dimerization)EK and CoEK models should be treated with caution. The derived dimerization constants for the SSA anionic component of co-formulation **a** (K_dim_ = 1.0 M^−1^) and **c** (K_dim_ = 1.5 M^−1^) are lower than that of the SSA alone (K_dim_ = 2.7 M^−1^), suggesting that the presence of the co-formulant has weakened the strength of the SSA anion self-associated complex.

Those solutions which exhibit an apparent ‘loss’ of solute from the solution during quantitative ^1^H NMR studies are not further characterised by solution-state NMR, as those molecules used to construct the larger self-associated aggregate species adopt solid-like properties (Figure 3). Instead, a variety of complementary experimental methods including tensiometry, zeta potential and DLS measurements are utilised to characterise the SSA aggregates. An overview of these results are summarised in Table 6.

Surface tension data derived from tensiometry measurements were used to calculate critical micelle concentration (CMC) at 298 K. The CMC value for each SSA or co-formulation was determined as the point at which the surface tension of the solution was no longer found to decrease with increasing compound concentration [34]. However, at compound concentrations below CMC stable aggregates can still exist in solution [35], which allows observation of these larger self-associated aggregates at concentrations lower than CMC. The CMC values calculated for a H_2_O 5.0% EtOH solution are listed in Table 6. Comparing these values, we observe a >70-fold increase in CMC between SSAs **4** (CMC = 0.5 mM) and **3** (CMC = 35.3 mM). We hypothesise that this variation in CMC value is due to a combination of preferential π-π stacking interactions, lipophobicity of the phenyl ring systems and intermolecular hydrogen bond strength, as supported by our previous observations [20]. Due to compound co-formulation solubility, CMC values could only be calculated for those co-formulations containing SSA **3**, where a decrease in the CMC value was observed for all co-formulations when compared to the SSA alone. Co-formulations **f** and **h** containing coumarin (**7**) and salicylic acid (**8**) respectively, were found to be the most effective agents, lowering the CMC from 35.3 mM (**3** alone) to ≈20.0 mM. However, the addition of aspirin (**9**) in co-formulation **j** was only found to lower the CMC to 24.2 mM. We believe that this may be due to the type of self-associated species produced in this instance as the presence of aspirin (**9**) within co-formulation **j** is found to substitute ≈50% less of the SSA anionic component within the larger self-associated structures formed compared to co-formulations **f** and **h** (Table 3).

Zeta potential measurements (Table 6) obtained for SSAs **1**–**4** (5.56 mM) in a H_2_O/ 5.0% EtOH solution confirmed the presence of stable aggregates (−30 mV ≥ zeta potential ≥ +30 mV). Interestingly, the aggregates produced by SSAs **10** (−14 mV) and **11** (−7 mV) are shown to be less stable than those of SSA **1** (−76 mV). Here the substitution of the TBA cation for the cationic dye molecules has caused a reduction in the stability of those self-associated structures produced by SSA **1**. A comparative decrease in aggregate stability was also observed when SSA **3** was co-formulated with coumarin (co-formulation **f**), salicylic acid (co-formulation **h**) and aspirin (co-formulation **j**) with zeta potential values of −65 mV, −43 mV, −36 mV and −21 mV respectively. When comparing the CMC and zeta potential values for co-formulations **f**, **h** and **j**, a decrease in CMC was found to correlate with a decrease in zeta potential value. Interestingly, when SSA **2** is co-formulated with a secondary species, the stability of the aggregates produced is greater than that of SSA **2** alone. The decrease in zeta potential value is irrespective of co-formulant, as a decrease from −30 mV to ≈−52 mV is observed upon the addition of both coumarin (co-formulation **e**) and salicylic acid (co-formulation **g**). We believe that this reversal of comparative SSA/co-formulation stability observed with SSAs **2** and **3** is due to the propensity of **2** to protonate, forming zwitterionic species under these environmental conditions. Therefore, the formation of SSA zwitterions appear to stabilise the larger self-associated species formed.

To determine the size of these self-associated aggregate species in solution, DLS studies were performed. The peak maxima obtained from average intensity size distributions are presented in Table 6. The size of aggregate obtained from these studies should be treated with caution as they assume the presence of spherical aggregated species [36]. SSAs **1**–**4** show a single distribution of these large aggregated structures, exhibiting the following trend: **4** (396 nm) > **2** (342 nm) > **1** (164 nm) > **3** (159 nm). The co-formulation of SSAs **2** and **3** with co-formulant agents **7**–**9** resulted in the increase of the hydrodynamic diameter of those self-associated aggregates formed. The presence of coumarin (**7**) and salicylic acid (**8**) with SSA **2** resulted in a significant increase in hydrodynamic diameter from 342 nm (SSA **2**) to 1317 nm and 2391 nm for co-formulations **e** and **g** respectively.

## 3. Materials and Methods

### 3.1. Synthesis

The synthesis of **1** [16], **2** [20], and **4** [18] have been previously published. SSA **3** was prepared through the reaction of diethylphenyldiamine and triphosgene in chloroform. This was followed by the addition of TBA aminomethanesulfonate. The pure product was obtained as a brown oil in a yield of 55%. Compounds **10** and **11** were both synthesised through the reaction of aminomethanesulfonic acid and trifluoromethylphenyl isocyanate in pyridine, to produce a pyridinium SSA intermediate [20]. The oxalate salt of malachite green (**10**) or the chloride salt of methylene blue (**11**) were added to a solution of this intermediate in methanol as appropriate. Following further purification SSA **10** was obtained as a blue/brown solid with a yield of 72% and **11** as a blue solid with a yield of 65%. Co-formulations **a**–**j** were prepared by dissolving the appropriate SSA (**1**–**4**) in methanol, followed by the addition of the secondary competitive species (one equivalent).

### 3.2. Single Crystal X-ray Diffraction Studies

A suitable crystal was selected and mounted on a Rigaku Oxford Diffraction Supernova diffractometer. Data were collected using Cu Kα radiation at 100 K. Structures were solved with the ShelXT [37] or ShelXS structure solution programs via Direct Methods and refined with ShelXL [37] on Least Squares minimisation. Olex2 [38] was used as an interface to all ShelX programs. CCDC deposition numbers for those structures shown in Figure 4, Figure 5, Figure 6 = 1997431−1997433.

## 4. Conclusions

We have explored the effects of competitive guest molecule addition on the self-associated structures produced by supramolecular self-associating amphiphiles (SSAs), highlighting the future potential of these systems as drug/molecule delivery vehicles. The self-associative properties of SSAs **1**–**4** are characterised in the gas phase, solution state and solid state, both alone and when co-formulated with various competitive molecular species to produce SSA co-formulations **a**–**j** (Table 1). Due to the complex nature of SSA self-association, and the additional complexities associated with the characterisation of these systems in the presence of additional guest species, we show how a combination of complementary experimental techniques may be used to form an understanding of the molecular interactions involved within these systems. This process has been summarised within the characterisation flow chart, shown in Figure 3.

Single crystal X-ray diffraction studies have shown the anionic component of SSA **3** to self-associate through urea-anion hydrogen bond formation to produce dimeric species (Figure 4). However, in the presence of salicylic acid, this same SSA anion is found to protonate, forming a zwitterion (Figure 5). Additionally, replacing the weakly coordinating TBA counter cation with a more strongly coordinating cation (**6**) results in the SSA anion forming a hydrogen-bonded tape rather than an anionic dimer. The presence of SSA anion dimerization was also confirmed in the gas phase for all SSAs and co-formulations studied.

Moving into the solution state, ^1^H NMR dilution and DOSY studies indicate the presence of predominantly lower ordered dimeric species in a DMSO-*d*_6_/0.5% H_2_O solution. This data provided little evidence that the co-formulants present were involved directly in SSA dimerization processes. However, the presence of the co-formulant agents were found to decrease the complexation strength of the SSA anionic dimers formed. Moving from competitive organic into aqueous (D_2_O/5.0% EtOH) solutions, quantitative ^1^H NMR studies confirmed the presence of larger self-associated aggregate species with all SSAs and co-formulations with the exception of SSA **2**. These experimental data show a high proportion of the co-formulant present incorporated in the larger self-associated structures produced by SSAs **1**–**4**. In addition, these data suggest that where a co-formulant is present the SSA aggregation process may be driven and the resultant aggregate further stabilised. Despite SSA **2** and **3** both containing protonatable sites appended from the phenyl ring of the SSA anion, zwitterion formation was only observed with co-formulations containing SSA **2**. We believe this is due to the balance of hydrophobic/hydrophilic moieties within the structure of SSAs **2** and **3**.

Finally, we have been able to show that when SSAs are co-formulated, we observe a general decrease in CMC value, an increase in extended self-associated aggregate size, and a retention of aggregate stability. Through these studies, we hope that we have been able to provide an experimental framework to support other researchers investigating complex, multi-component self-association events. Additionally, the fundamental results presented here are currently being used to guide the development of SSA molecular delivery systems.

## Figures and Tables

**Table 1:** Molecular components contained within co-formulations **a**–**j**.

Co-Formulation	Molecular Components	Co-Formulation	Molecular Components
**a**	**1** + **5**	**f**	**3** + **7**
**b**	**4** + **5**	**g**	**2** + **8**
**c**	**1** + **6**	**h**	**3** + **8**
**d**	**4** + **6**	**i**	**2** + **9**
**e**	**2** + **7**	**j**	**3** + **9**

**Table 2:** High resolution electrospray ionisation (ESI) −ve mass spectrometry theoretical and experimentally derived values.

	*m/z* [M]^-^		*m/z* [M + M + H^+^]^-^	
Compound	Theoretical	Actual	Theoretical	Actual
**3**	300.1024	300.1012	601.2048	601.2091
**10**	297.0162	297.0276	595.0324	595.0596
**11**	297.0162	297.0157	595.0324	595.0384

**Table 3:** Overview of the results from quantitative ^1^H NMR studies obtained from (i) DMSO-*d*_6_, standardised with 1.0% DCM at 112 mM and; (ii) D_2_O standardised with 5.0% ethanol at 5.56 mM. Values given in % represent the observed proportion of compound to become NMR silent. All quantitative ^1^H NMR experiments were conducted with a delay time (*d*_1_) of 60 s at 298 K.

Co-Formulation	Compound	Solvent System	Anion	Cation	Co-Formulant	Anion	Cation
n/a	**1** [16]	DMSO-*d*_6_	0	0	n/a	n/a	n/a
		D_2_O	51	50			
n/a	**2**	DMSO-*d*_6_	0	0	n/a	n/a	n/a
		D_2_O	0	0			
n/a	**3** [21]	DMSO-*d*_6_	0	0	n/a	n/a	n/a
		D_2_O	65	21			
n/a	**4** [18]	DMSO-*d*_6_	0	0	n/a	n/a	n/a
		D_2_O	10	8			
n/a	**10**	DMSO-*d*_6_	0	0	n/a	n/a	n/a
		D_2_O	100	100			
n/a	**11**	DMSO-*d*_6_	0	0	n/a	n/a	n/a
		D_2_O	100	100			
**a**	**1**	DMSO-*d*_6_	0	0	**5**	0	
		D_2_O	100	15		100	
**b**	**4**	DMSO-*d*_6_	0	0	**5**	0	
		D_2_O	100	37		100	
**c**	**1**	DMSO-*d*_6_	0	0	**6**	0	
		D_2_O	100	28		100	
**d**	**4**	DMSO-*d*_6_	74	12	**6**	*a*	
		D_2_O	100	0		100	
**e**	**2**	DMSO-*d*_6_	0	0	**7**	n/a	0
		D_2_O	52	55			86
**f**	**3**	DMSO-*d*_6_	0	0	**7**	n/a	0
		D_2_O	41	43			81
**g**	**2**	DMSO-*d*_6_	0	0	**8**	n/a	0
		D_2_O	63	44			58
**h**	**3**	DMSO-*d*_6_	0	0	**8**	n/a	0
		D_2_O	42	44			34
**i**	**2**	DMSO-*d*_6_	12	2	**9**	n/a	4
		D_2_O	*a*	*a*			*a*
**j**	**3**	DMSO-*d*_6_	0	0	**9**	n/a	0
		D_2_O	22	40			19

Cells have been merged where compound/co-formulant is neither anionic nor cationic. *a* = Could not be calculated due to compound solubility. n/a = not applicable.

**Table 4:** Self-association constants (M^−1^) calculated for compounds **1**–**11** and co-formulations **a**–**j** in a DMSO-*d*_6_/0.5% H_2_O solution at 298 K. These constants were obtained from the fitting of ^1^H NMR dilution data and refined to equal K (EK) and cooperative equal K (CoEK) models using BindFit v0.5 [29].

Compound/	EK Model (M^−1^)		CoEK (M^−1^)		
Co-Formulation	K_e_	K_dim_	K_e_	K_dim_	ρ
**1** [16]	5.3 (±0.6%)	2.7 (±0.3%)	13.0 (±0.7%)	6.5 (±0.4%)	0.5 (±2.1%)
**2** [21]	3.6 (±1.5%)	1.8 (±0.7%)	4.6 (±8.3%)	2.3 (±4.1%)	0.9 (±13.4%)
**3**	1.2 (±1.0%)	0.6 (±0.5%)	8.9 (±2.1%)	4.5 (±1.1%)	0.3 (±5.7%)
**4** [18]	5.3 (±0.6%)	2.7 (±0.3%)	13.0 (±0.7%)	6.5 (±0.3%)	0.5 (±2.0%)
**5**	*a*	*a*	*a*	*a*	*a*
**6**	*a*	*a*	*a*	*a*	*a*
**7**	*a*	*a*	*a*	*a*	*a*
**8**	*a*	*a*	*a*	*a*	*a*
**9**	*a*	*a*	*a*	*a*	*a*
**10**	5.2 (±1.2%)	2.6 (±0.6%)	13.9 (±2.3%)	6.9 (±1.1%)	0.5 (±6.8%)
**11**	3.5 (±0.7%)	1.8 (±0.4%)	8.6 (±2.3%)	4.3 (±1.1%)	0.6 (±5.1%)
**a** *^d^*	2.0 (±0.8%)	1.0 (±0.4%)	0.9 (±20.2%)	0.5 (±10.1%)	1.6 (±22.8%)
**b** *^d^*	*b*	*b*	*b*	*b*	*b*
**c** *^d^*	3.0 (±1.3%)	1.5 (±0.6%)	8.3 (±4.0%)	4.1 (±2.0%)	0.5 (±9.2%)
**d** *^d^*	*c*	*c*	*c*	*c*	*c*
**e** *^d^*	0.4 (±0.9%)	0.2 (±0.5%)	0.2 (± 826.6%)	0.1 (±413.3%)	1.5 (±829.5%)
**f** *^d^*	0.4 (±0.6%)	0.2 (±0.3%)	1.9 (±7.5%)	0.9 (±3.8%)	0.6 (±9.7%)
**g** *^d^*	2.2 (±0.7%)	1.1 (±0.3%)	4.0 (±4.3%)	2.0 (±2.1%)	0.7 (±6.7%)
**h** *^d^*	2.4 (±0.6%)	1.2 (±0.3%)	1.1 (±12.5%)	0.6 (±6.2%)	1.6 (±14.4%)
**i** *^d^*	*c*	*c*	*c*	*c*	*c*
**j** *^d^*	0.6 (±1.3%)	0.3 (±0.6%)	0.3 (±92.5%)	0.1 (±46.5%)	1.5 (± 96.5%)

*a* = Not applicable due to compounds being purchased/ previously known compounds. *b* = Multiple association events prevent data fitting. *c* = Loss of compound observed in ^1^H quantitative NMR studies. *d* = Values to be treated with caution due to multiple component solution.

**Table 5:** Overview of hydrodynamic diameters (nm) for compounds **1**–**11** and co-formulations **a**–**j** in DMSO-*d*_6_ at 298 K.

Co-Formulation	Compound	Anion	Cation	Co-Formulant	Anion	Cation
n/a	**1** [16]	1.15	1.08	n/a	n/a	n/a
n/a	**2** [21]	1.43	1.32	n/a	n/a	n/a
n/a	**3**	1.46	2.38	n/a	n/a	n/a
n/a	**4** [18]	1.61	1.51	n/a	n/a	n/a
n/a	**5**	*a*	*a*	n/a	n/a	n/a
n/a	**6**	*a*	*a*	n/a	n/a	n/a
n/a	**7**	*a*	*a*	n/a	n/a	n/a
n/a	**8**	*a*	*a*	n/a	n/a	n/a
n/a	**9**	*a*	*a*	n/a	n/a	n/a
n/a	**10**	1.02	1.04	n/a	n/a	n/a
n/a	**11**	*b*	*b*	n/a	n/a	n/a
**a**	**1**	1.42	1.27	**5**	1.54	
**b**	**4**	*b*	*b*	**5**	*b*	*b*
**c**	**1**	1.52	1.36	**6**	1.52	
**d**	**4**	*c*	*c*	**6**	*c*	*c*
**e**	**2**	1.59	1.43	**7**	1.15	n/a
**f**	**3**	1.62	1.39	**7**	1.12	n/a
**g**	**2**	1.78	1.49	**8**	1.03	n/a
**h**	**3**	1.84	1.46	**8**	1.05	n/a
**i**	**2**	*c*	*c*	**9**	*c*	*c*
**j**	**3**	1.58	1.38	**9**	1.03	n/a

Cells have been merged where compound/co-formulant is neither anionic nor cationic. *a* = n/a (not applicable) due to compounds being purchased/known compounds. *b* = Could not be determined due to peak overlap. *c* = Loss of compound observed in ^1^H quantitative NMR studies.

**Table 6:** Overview of average intensity particle size distribution peak maxima (nm), zeta potential (mV), CMC (mM) and surface tension at CMC (mN/m), measurements obtained for a H_2_O/ 5.0% EtOH solution of an SSA or co-formulation (5.56 mM) at 298 K.

Compound/ Co-Formulation	Peak Maxima	Zeta Potential	CMC	Surface Tension at CMC
**1** [16]	164	−76	10.4	37.5
**2** [21]	342	−30	*e*	*e*
**3**	159	−65	35.3	32.2
**4** [18]	396	−101	0.5	46.5
**5**	*a*	*a*	*a*	*a*
**6**	*a*	*a*	*a*	*a*
**7**	*a*	*a*	*a*	*a*
**8**	*a*	*a*	*a*	*a*
**9**	*a*	*a*	*a*	*a*
**10**	*b*	−14	*e*	*e*
**11**	*c*	−7	*e*	*e*
**a**	*c*	*c*	*e*	*e*
**b**	*c*	*c*	*e*	*e*
**c**	*c*	*c*	*e*	*e*
**d**	*c*	*c*	*e*	*e*
**e**	1317	−52	*e*	*e*
**f**	768	−43	19.9	38
**g**	2391	−52	*e*	*e*
**h**	427	−36	19.8	38.1
**i**	*c*	*c*	*e*	*e*
**j**	466*^d^*	−21	24.2	67.3

*a* = n/a (not applicable) purchased/known compounds. *b* = Poor correlation function prevented fitting. *c* = Could not be calculated due to compound solubility. *d* = Sample suspected to be unstable during measurement, treat with caution. *e* = CMC value was determined to be greater than saturation point of the solution.

## Supplementary Materials

The following are available online: details of all experimental procedures, materials and supporting experimental data for quantitative NMR, DOSY NMR, NMR dilution studies, single crystal X-ray studies, high resolution mass spectrometry, CMC determination, DLS and zeta potential studies.

## Sample Availability

Samples of these compounds may be made available depending on availability.

## References

[1] Wang C., Wang Z., Zhang X. (2012). Amphiphilic Building Blocks for Self-Assembly: From Amphiphiles to Supra-amphiphiles. Acc. Chem. Res..

[2] Yu G., Jie K., Huang F. (2015). Supramolecular Amphiphiles Based on Host–Guest Molecular Recognition Motifs. Chem. Rev..

[3] Kumar S., Ludwig K., Schade B., Von Berlepsch H., Papp I., Tyagi R., Gulia M., Haag R., Böttcher C. (2016). Introducing Chirality into Nonionic Dendritic Amphiphiles and Studying Their Supramolecular Assembly. Chem. Eur. J..

[4] Thota B.S.N., Urner L. H., Haag R. (2016). Supramolecular Architectures of Dendritic Amphiphiles in Water. Chem. Rev..

[5] Tian J., Chen L., Zhang D.W., Liu Y., Li Z.T. (2016). Supramolecular organic frameworks: Engineering periodicity in water through host–guest chemistry. Chem. Commun..

[6] Sangeetha N.M., Maitra U. (2005). Supramolecular gels: Functions and uses. Soc. Rev..

[7] Steed J.W. (2011). Supramolecular gel chemistry: Developments over the last decade. Chem. Commun..

[8] Webber M.J., Langer R. (2017). Drug delivery by supramolecular design. Chem. Soc. Rev..

[9] Raymond D.M., Abraham B.L., Fujita T., Watrous M.J., Toriki E.S., Takano T., Nilsson B.L. (2019). Low-Molecular-Weight Supramolecular Hydrogels for Sustained and Localized in Vivo Drug Delivery. ACS Appl. Biol. Mater..

[10] Cai Y., Shen H., Zhan J., Lin M., Dai L., Ren C., Shi Y., Liu J., Gao J., Yan Z. (2017). Supramolecular "Trojan Horse" for Nuclear Delivery of Dual Anticancer Drugs. J. Am. Chem. Soc..

[11] Gale P.A., Caltagirone C. (2015). Anion sensing by small molecules and molecular ensembles. Chem. Soc. Rev..

[12] Hiscock J.R., Wells N.J., Ede J.A., Gale P.A., Sambrook M.R. (2016). Biasing hydrogen bond donating host systems towards chemical warfare agent recognition. Org. Biomol. Chem..

[13] Faustino C.M.C., Calado A.R.T., Garcia-Rio L. (2009). New Urea-Based Surfactants Derived from α,ω-Amino Acids. J. Phys. Chem. B.

[14] Faustino C.M.C., Calado A.R.T., Garcia-Rio L. (2010). Dimeric and monomeric surfactants derived from sulfur-containing amino acids. J. Colloid. Interface Sci..

[15] Rawling T., MacDermott-Opeskin H., Roseblade A., Pazderka C., Clarke C., Bourget K., Wu X., Lewis W., Noble B., Gale P.A. (2020). Aryl urea substituted fatty acids: A new class of protonophoric mitochondrial uncoupler that utilises a synthetic anion transporter. Chem. Sci..

[16] Hiscock J.R., Bustone G.P., Wilson B., Belsey K.E., Blackholly L.R. (2016). In situ modification of nanostructure configuration through the manipulation of hydrogen bonded amphiphile self-association. Soft. Matter..

[17] Blackholly L.R., Shepherd H.J., Hiscock J.R. (2016). ‘Frustrated’ hydrogen bond mediated amphiphile self-assembly–A solid state study. CrystEngComm.

[18] White L.R., Wells N.J., Blackholly L.R., Shepherd H.J., Wilson B., Bustone G.P., Runacres T.J., Hiscock J.R. (2017). Towards quantifying the role of hydrogen bonding within amphiphile self-association and resultant aggregate formation. Chem. Sci..

[19] Gumbs T.L., White L.J., Wells N.J., Shepherd H.J., Hiscock J.R. (2018). ‘Frustrated’ hydrogen-bonded self-associated systems as templates towards DNA incorporated nanostructure formation. Supramol. Chem..

[20] White L.J., Tyuleva S.N., Wilson B., Shepherd H.J., Ng K.K.L., Holder S.J., Clark E.R., Hiscock J.R. (2018). Towards the Prediction of Global Solution State Properties for Hydrogen Bonded, Self-Associating Amphiphiles. Chem. Eur. J..

[21] Tyuleva S.N., Allen N., White L.J., Pépés A., Shepherd H.J., Saines P.J., Ellaby R.J., Mulvihill D.P., Hiscock J.R. (2019). A symbiotic supramolecular approach to the design of novel amphiphiles with antibacterial properties against MSRA. Chem. Commun..

[22] Ng K.K.L., Dimitrovski M., Boles J.E., Ellaby R.J., White L.J., Hiscock J.R. (2020). Towards the use of (pseudo) nucleobase substituted amphiphiles as DNA nucleotide mimics and antimicrobial agents. Supramol. Chem..

[23] White L.J., Boles J.E., Allen N., Alesbrook L., Sutton J.M., Hind C., Hilton K.L.F., Blackholly L.R., Ellaby R.J., Williams G.T. (2020). Controllable hydrogen bonded self-association for the formation of multifunctional antimicrobial materials. J. Mater. Chem. B.

[24] Townshend G., Thompson G.S., White L.J., Hiscock J.R., Ortega-Roldan J.L. (2020). The elucidation of phospholipid bilayer–small molecule interactions using a combination of phospholipid nanodiscs and solution state NMR techniques. Chem. Commun..

[25] Li J., Kuang Y., Shi J., Gao Y., Zhou J., Xu B. (2013). The conjugation of nonsteroidal anti-inflammatory drugs (NSAID) to small peptides for generating multifunctional supramolecular nanofibers/hydrogels. Beilstein J. Org. Chem..

[26] Thakur S., Riyaz B., Patil A., Kaur A., Kapoor B., Mishra V. (2018). Novel drug delivery systems for NSAIDs in management of rheumatoid arthritis: An overview. Biomed. Pharmacother..

[27] Lide D. (2004). CRC Handbook of Chemistry and Physics.

[28] Coumarin-3-Carboxylic Acid | 531-81-7. https://www.chemicalbook.com/ChemicalProductProperty_EN_CB4399823.htm.

[29] Supramolecular.org-Binding Constant Calculators | Bindfit V0.5. http://supramolecular.org/.

[30] Von Krbek L.K.S., Schalley C.A., Thordarson P. (2017). Assessing cooperativity in supramolecular systems. Chem. Soc. Rev..

[31] Stoesser P.R., Gill S.J. (1967). Calorimetric study of self-association of 6-methyl-purine in water. J. Phys. Chem..

[32] Evstigneev M.P., Buchelnikov A.S., Kostjukov V.V., Pashkova I.S., Evstigneev V.P. (2013). Indistinguishability of the models of molecular self-assembly. Supramol. Chem..

[33] Martin R.B. (1996). Comparisons of Indefinite Self-Association Models. Chem. Rev..

[34] Piñeiro Á., Banquy X., Pérez-Casas S., Tovar E., García A., Villa A., Amigo A., Mark A.E., Costas M. (2007). On the Characterization of Host−Guest Complexes:  Surface Tension, Calorimetry, and Molecular Dynamics of Cyclodextrins with a Non-ionic Surfactant. J. Phys. Chem. B.

[35] Ruckenstein E., Nagarajan R. (1975). Critical micelle concentration. Transition point for micellar size distribution. J. Phys. Chem..

[36] Stetefeld J., McKenna S.A., Patel T.R. (2016). Dynamic light scattering: A practical guide and applications in biomedical sciences. Biophys. Rev..

[37] Sheldrick G.M. (2015). Crystal structure refinement with SHELXL. Acta Crystallogr..

[38] Dolomanov O.V., Bourhis L.J., Gildea R.J., Howard J.A.K., Puschmann H. (2009). OLEX2: A complete structure solution, refinement and analysis program. J. Appl. Crystallogr..

