# Supplementary material for: Towards the Application of Supramolecular Self-Associating Amphiphiles as Next-Generation Delivery Vehicles
Source: Molecules. 2020 Sep 9;25(18):4126. doi: 10.3390/molecules25184126 (PMC7570936; doi:10.3390/molecules25184126)
Supplement: Supplementary file 1 [file molecules-25-04126-s001.zip › supplimentary information/ESI.pdf]

Towards the application of supramolecular self-associating amphiphiles as next-generation delivery vehicles

Lisa J. White [1], Jessica E. Boles [1], Kira Hilton [1], Rebecca J. Ellaby [1] and Jennifer R. Hiscock [1*]

Contents

Tables of data 2
Experimental 5
Chemical structures 6
Chemical synthesis 8
NMR 12
Characterisation NMR 12
^{1}H NMR quantitative studies 26
^{1}H NMR DOSY studies 41
Overview 62
^{1}H NMR self-association studies 64
Timed ^{1}H NMR 84
Dynamic Light Scattering data 85
Overview 99
Surface Tension and Stability data 100
Zeta Potential 100
Surface Tension Measurements and Critical Micelle Concentration (CMC) Determination 104
Overview 106
Single crystal X-ray structures 107
Low level *in silico* modelling 110
Overview 114
Mass Spectrum data 115
Overview 117
References 118

Tables of data

Table S1 – Overview of gaseous and solution state studies observed for compounds **1-11** and co-formulation ***a-j***.

Compound	Gas Phase dimer	K_{dim} (M^{-1})	Size (nm)	Zeta potential (mV)	CMC (mM)	Surface tension (mN m^{-1})
**1** [1]	Y	2.70	164	-76	10.39	37.45
**2** [2]	Y	1.81	340	-30	*f*	*f*
**3**	Y	0.59	159	-65.2	32.27	32.21
**4** [3]	Y	2.70	122	-101	0.50	46.50
**5**	*a*	*a*	*a*	*a*	*a*	*a*
**6**	*a*	*a*	*a*	*a*	*a*	*a*
**7**	*a*	*a*	*a*	*a*	*a*	*a*
**8**	*a*	*a*	*a*	*a*	*a*	*a*
**9**	*a*	*a*	*a*	*a*	*a*	*a*
**10**	Y	2.60	*c*	-13.6	*f*	*f*
**11**	Y	1.75	*d*	-7.4	*f*	*f*
a	*a*	0.98	*d*	*d*	*f*	*f*
b	*a*	*b*	*d*	*d*	*f*	*f*
c	*a*	1.52	*d*	*d*	*f*	*f*
d	*a*	*b*	*d*	*d*	*f*	*f*
e	*a*	0.22	1317	-51.8	*f*	*f*
f	*a*	0.22	768	-42.7	19.81	37.95
g	*a*	1.10	2319	-52.1	*f*	*f*
h	*a*	1.20	427	-35.8	19.76	38.10
i	*a*	*b*	*d*	*d*	*f*	*f*
j	*a*	0.29	466[e]	-20.7	24.23	67.28

a — n/a purchased compounds/known compounds.
b – Multiple association events prevent data fitting.
c – Poor correlation function preventing data fitting.
d – Could not be calculated due to compound solubility.
e – Sample suspected to be unstable during measurement, treat with caution.
f – CMC value was found to be greater than saturation point.

Table S2 – Overview of the results from quantitative ^{1}H NMR studies. Values given in % represent the observed proportion of compound that became NMR silent.

Co-formulation	Compound	Solvent system	Anion	Cation	Co-formulant	Anion	Cation
n/a	**1** [1]	DMSO-d_6	0	0	n/a	n/a	n/a
		D_2O	51	50			
n/a	**2**	DMSO-d_6	0	0	n/a	n/a	n/a

		D_2O	0	0			
n/a	**3** [2]	DMSO-d_6	0	0	n/a	n/a	n/a
		D_2O	65	21			
n/a	**4** [3]	DMSO-d_6	0	0	n/a	n/a	n/a
		D_2O	10	8			
n/a	**10**	DMSO-d_6	0	0	n/a	n/a	n/a
		D_2O	100	100			
n/a	**11**	DMSO-d_6	0	0	n/a	n/a	n/a
		D_2O	100	100			
**a**	**1**	DMSO-d_6	0	0	**5**	0	
		D_2O	100	15		100	
**b**	**4**	DMSO-d_6	0	0	**5**	0	
		D_2O	100	37		100	
**c**	**1**	DMSO-d_6	0	0	**6**	0	
		D_2O	100	28		100	
**d**	**4**	DMSO-d_6	74	12	**6**	*a*	
		D_2O	100	0		100	
**e**	**2**	DMSO-d_6	0	0	**7**	n/a	0
		D_2O	52	55			86
**f**	**3**	DMSO-d_6	0	0	**7**	n/a	0
		D_2O	41	43			81
**g**	**2**	DMSO-d_6	0	0	**8**	n/a	0
		D_2O	63	44			58
**h**	**3**	DMSO-d_6	0	0	**8**	n/a	0
		D_2O	42	44			34
**i**	**2**	DMSO-d_6	12	2	**9**	n/a	4
		D_2O	*a*	*a*			*a*
**j**	**3**	DMSO-d_6	0	0	**9**	n/a	0
		D_2O	22	40			19

Cells have been merged where compound/co-formulant is neither anionic nor cationic.
a – Could not be calculated due to compound solubility.

Table S3 – Overview of the calculated E_{max}, E_{min} and Log*P* values using semi empirical PMS modelling methods of the anionic components of compounds **2**, **3**, **5 - 9** and co-formulations ***e*** and ***f***.

Compound	E_{max} (KJ mol^{-1})	E_{min} (KJ mol^{-1})	Log*P*
**2**	-727.631	-12.261	-0.42
**3**	-726.001	-70.648	0.79
**anionic 5**	-786.835	-151.654	-1.32
**cationic 5**	156.555	328.968	1.94 *a*/ 2.33 *b*
**6**	17.5163	354.054	1.38 *a*/ 2.39 *b*
**8**	-683.365	-200.281	0.97
**9**	-754.765	-147.64	1.02
e	-533.121	518.742	-1.30
f	-545.206	380.695	-0.21

a - with Cl^-

b - without Cl^-

Experimental

General remarks: A positive pressure of nitrogen and oven dried glassware were used for all reactions. All solvents and starting materials were purchased from known chemical suppliers or available stores and used without any further purification unless specifically stipulated. The NMR spectra were obtained using a Burker AV2 400 MHz or AVNEO 400 MHz spectrometer. The data was processed using ACD Labs, MestReNova or Topspin software. NMR Chemical shift values are reported in parts per million (ppm) and calibrated to the centre of the residual solvent peak set (s = singlet, br = broad, d = doublet, t = triplet, q = quartet, m = multiplet). Tensiometry measurements were undertaken using the Biolin Scientific Theta Attension optical tensiometer. The data was processed using Biolin OneAttension software. A Hamilton (309) syringe was used for the measurements. The melting point for each compound was measured using Stuart SMP10 melting point apparatus. High resolution mass spectrometry was performed using a Bruker microTOF-Q mass spectrometer and spectra recorded and processed using Bruker's Compass Data Analysis software. Infrared spectra were obtained using a Shimadzu IR-Affinity-1 model Infrared spectrometer. The data are analysed in wavenumbers (cm^{-1}) using IRsolution software. DLS and Zeta Potential studies were carried out using Anton Paar Litesizer™ 500 and processed using KalliopeTM Professional.

Mass Spectrometry: Approximately 1 mg of each compound was dissolved in 1 mL of methanol. This solution was further diluted 100-fold before undergoing analysis where 10 µL of each sample was then injected directly into a flow of 10 mM ammonium acetate in 95 % water (flow rate = 0.02 mL/min).

Self-association constant calculation: Self-association constants were determined using Bindfit v0.5 (http://app.supramolecular.org/bindfit/). All the data can be accessed online using the hyperlinks provided.

Tensiometry Studies: All the samples were prepared in an EtOH:H_2O (1:19) solution. All samples underwent an annealing process in which the various solutions were heated to approximately 40 °C before being allowed to cool to room temperature, allowing each sample to reach a thermodynamic minimum. All samples were prepared through serial dilution of the most concentrated sample. Three surface tension measurements were obtained for each sample at a given concentration, using the pendant drop method. The average values were then used to calculate the critical micelle concentration (CMC).

DLS Studies: All vials used for preparing the samples were clean dry. All solvents used were filtered to remove any particulates that may interfere with the results obtained. Samples of differing concentrations were obtained through serial dilution of a concentrated solution. All samples underwent an annealing process, in which they were heated to 40 °C before being allowed to cool to 25 °C. A series of 9 or 10 runs were recorded at 25 °C.

Zeta Potential Studies: All vials used for preparing the samples were clean dry. All solvents used were filtered to remove any particulates that may interfere with the results obtained. All samples underwent an annealing process in which the various solutions were heated to approximately 40 °C before cooling to room temperature, allowing each sample to reach a thermodynamic minimum. The final zeta potential value given is an average of the number of experiments conducted at 25 °C.

Single Crystal X-ray Studies: A suitable crystal of each amphiphile was selected and mounted on a Rigaku Oxford Diffraction Supernova diffractometer. Data were collected using Cu Kα radiation at 100 K or 293 K as necessary due to crystal instability at lower temperatures. Structures were solved with the ShelXT or ShelXS structure solution programs via Direct Methods and refined with ShelXL by Least Squares minimisation. Olex2 was used as an interface to all ShelX programs (CCDC 1997431-1997433).

Chemical structures

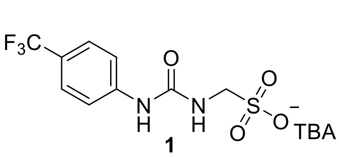

1

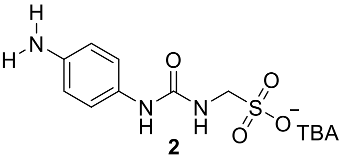

2

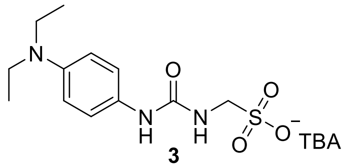

3

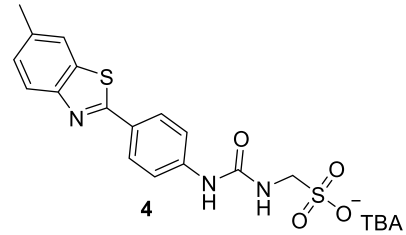

4

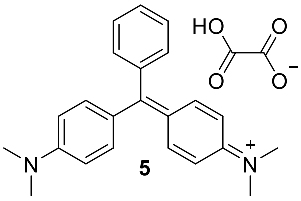

5

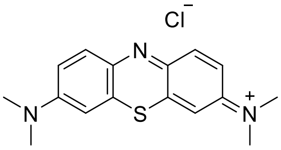

6

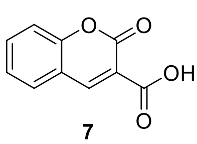

7

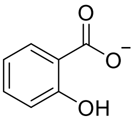

8

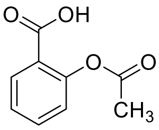

9

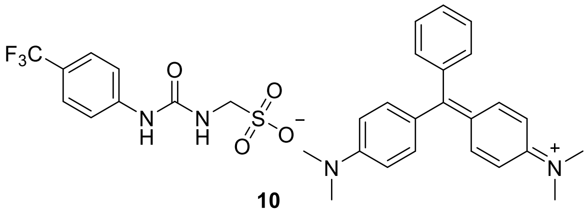

10

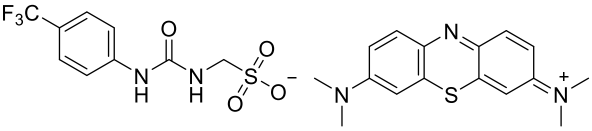

11

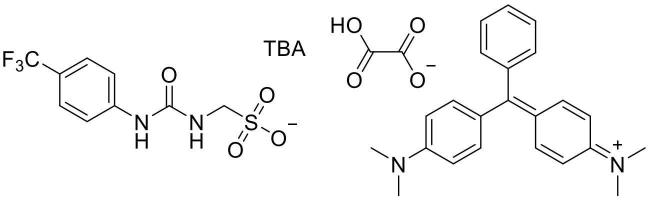

Co-formulation *a*

Figure S1 – TBA = tetrabutylammonium

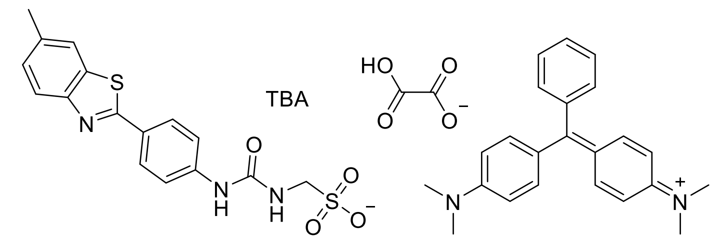

Co-formulation *b*

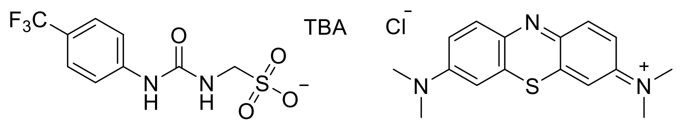

Co-formulation *c*

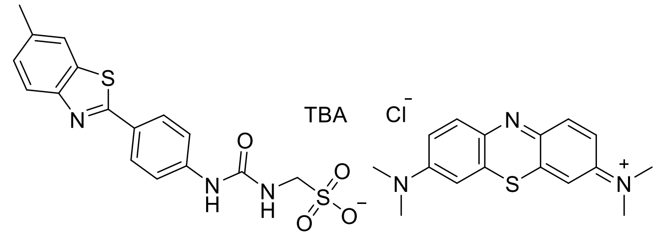

Co-formulation *d*

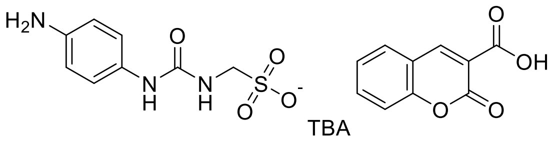

Co-formulation *e*

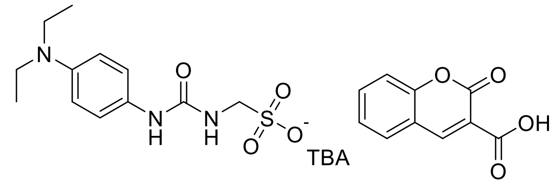

Co-formulation *f*

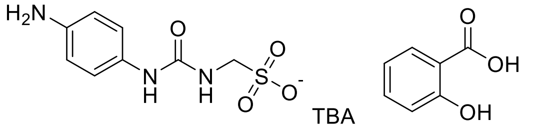

Co-formulation *g*

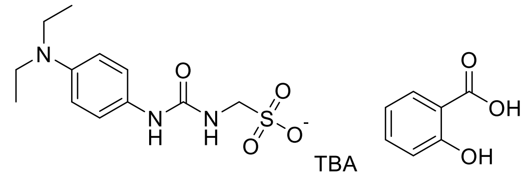

Co-formulation *h*

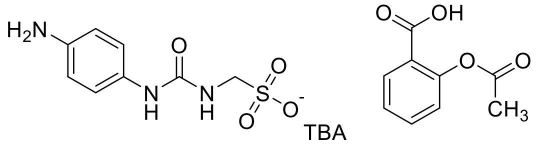

Co-formulation *i*

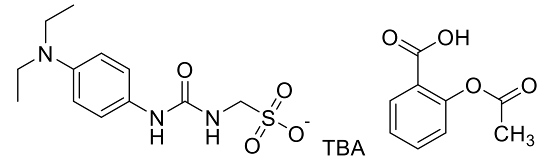

Co-formulation *j*

Figure S2 – TBA = tetrabutylammonium

Chemical synthesis

Compound 1: This compound was synthesized in line with our previously published methods [1]. ^{1}H NMR (400 MHz, 298 K, DMSO-d_6): δ: 9.33 (s, 1H), 7.55 (d, *J* = 7.80 Hz, 2H), 7.40 (m, 3H), 4.00 (d, *J* = 5.04 Hz, 2H), 3.15 (m, 8H), 1.54 (s, 8H), 1.29 (m, 8H), 0.91 (t, *J* = 6.88 Hz, 12H.

Compound 2: This compound was synthesized in line with our previously published method [2]. ^{1}H NMR (400 MHz, 298 K, DMSO-d_6): δ: 8.23 (s, 1H), 6.99 (d, *J* = 8.72 Hz, 2H), 6.45 (t, *J* = 6.80 Hz, 2H), 6.19 (s, 1H), 4.64 (s, 2H), 3.82 (d, *J* = 5.76 Hz, 2H), 3.16 (m, 8H), 1.57 (m, 8H), 0.93 (t, *J* = 7.24 Hz, 12H).

Compound 3: Triphosgene (0.59 g, 2.0 mM) was added to diethylphenyldiamine (0.33 mL, 2.0 mM) and a saturated solution of sodium bicarbonate (20 mL) in chloroform (20 mL) and left to stir at RT for 4 hours. The organic layer was then separated and magnesium sulfate added to remove excess water. Aminomethanesulfonic acid (0.22 g, 2.0 mM) was dissolved in tetrabutylammonium hydroxide in methanol (2.0 mL, 2.0 mM). Tetrabutylammonium aminomethanesulfonate (2.0 mM) was dissolved in chloroform (10 mL) and added to a stirring solution of the isocyanate in chloroform (20 mL), refluxed at 80 °C under an inert atmosphere over night. The organic phase was then twice washed with H_2O (20 mL) and the organic layer taken to dryness to give a brown oil with a yield of 55 % (0.60 g, 1.1 mM); Melting Point: oil; ^{1}H NMR (400 MHz, 298 K, DMSO-d_6): δ: 8.32 (s, 1H), 7.14 (d, *J* = 9.00 Hz, 2H), 6.59 (d, *J* = 8.96 Hz, 2H), 6.19 (t, *J* = 5.24 Hz, 1H), 3.84 (d, *J* = 5.76 Hz, 2H), 3.24 (q, *J* = 7.16 Hz, 4H), 3.16 (t, *J* = 8.32 Hz, 8H), 1.57 (m, 8H), 1.31 (m, 8H), 1.04 (t, *J* = 7.16 Hz, 6H), 0.94 (t, *J* = 7.32 Hz, 12H); $^{13}C\{^{1}H\}$ NMR (100 MHz, 298 K, DMSO-d_6): δ: 155.4 (CO), 143.4 (ArC), 130.2 (ArC), 120.3 (ArCH), 113.4 (ArCH), 58.0 (CH_2), 56.6 (CH_2), 44.4 (CH_2), 23.5 (CH_2), 19.7 (CH_2), 14.0 (CH_3), 12.9 (CH_3); IR (film): ν = 3258 (NH stretch), 1615, 1217, 1180, 881; HRMS for the sulfonate-urea ion ($C_{12}H_{18}N_3O_4S^-$) (ESI$^-$): m/z: act: 300.1012 [M]$^-$ cal: 300.1024 [M]$^-$.

Compound 4: This compound was synthesized in line with our previously published methods [3]. ^{1}H NMR (400 MHz, 298 K, DMSO-d_6): δ: 9.15 (s, 1H), 7.90 (m, 4H), 7.56 (d, *J* = 8.84 Hz, 2H), 7.32 (m, 1H), 6.62 (s, 1H), 3.89 (d, *J* = 5.76 Hz, 2H), 3.15 (m, 8H), 2.44 (s, 3H), 1.56 (m, 8H), 1.30 (m, 8H), 0.93 (t, *J* = 7.24 Hz, 12H).

Compound 10: Aminomethanesulfonic acid (0.22 g, 2.00 mM) was added to a stirring solution of 4-(trifluoromethyl)phenyl isocyanate (0.37 g, 2.00 mM) in pyridine (20 mL). The reaction was then refluxed at 60 °C under an inert atmosphere over night and filtered to give a white solid with a yield of 90 % (0.68 g, 1.80 mM). To the resultant compound (0.19 g, 0.50 mM) malachite green oxalate salt (0.21 g, 0.50 mM) was added in methanol (20 mL) and taken to dryness. Dissolved in chloroform (20 mL) and twice washed with H_2O (20 mL), the organic phase taken to dryness to give a blue/brown solid with a yield of 72 % (0.23 g, 0.40 mM); Melting Point: 96 °C; ^{1}H NMR (400 MHz, 298 K, DMSO-d_6): δ: 9.26 (s, 1H), 7.44 (t, *J* = 9.00 Hz, 1H), 6.61 (t, *J* = 7.56 Hz, 2H), 7.53 (d, *J* = 8.44 Hz, 2H), 7.43 (d, *J* = 8.56 Hz, 2H), 7.30 (m, 6H), 7.07 (m, 5H), 3.93 (d, *J* = 5.92 Hz, 2H), 3.29 (s, 12H); $^{13}C\{^{1}H\}$ NMR (100 MHz, 298 K, DMSO-d_6): δ: 175.8 (tetra C), 156.9 (CO), 154.7 (ArC), 144.6 (ArC), 140.6 (ArCH), 139.8 (ArC), 134.7 (ArCH), 133.4 (ArCH), 129.1 (ArCH), 127.0 (ArCH), 126.4 (ArC), 121.9-121.0 (q, *J* = 26.19 Hz, CF_3), 117.6 (ArCH), 114.5 (ArCH), 56.5 (CH_2), 41.0 (CH_3); IR (film): ν = 3258 (NH stretch), 1615, 1217, 1180, 881; HRMS for the sulfonate-urea ion ($C_9H_8F_3N_2O_4S^-$) (ESI$^-$): m/z: act: 297.0276 [M]$^-$ cal: 297.0162 [M]$^-$.

Compound 11: Aminomethanesulfonic acid (0.22 g, 2.00 mM) was added to a stirring solution of 4-(trifluoromethyl)phenyl isocyanate (0.37 g, 2.00 mM) in pyridine (20 mL). The reaction was then refluxed at 60 °C under an inert atmosphere over night and filtered to give a white solid with a yield of 90 % (0.68 g, 1.80 mM). To the resultant compound (0.19 g, 0.50 mM) methylene blue chloride (0.17 g, 0.50 mM) was added in methanol (20 mL) and taken to dryness. Dissolved in chloroform (20 mL) and washed with H_2O (20 mL), resulting in precipitation this was then filtered to give a blue solid with a yield of 65 % (0.19 g, 0.30 mM); Melting Point: 98 °C; ^{1}H NMR (400 MHz, 298 K, DMSO-d_6): δ: 9.20 (s, 1H), 7.86 (d, *J* = 9.16 Hz, 2H), 7.54-7.42 (m, 8H), 6.81 (s, 1H), 3.92 (d, *J* = 5.80 Hz, 2H), 3.32 (s, 12H); $^{13}C\{^{1}H\}$ NMR (100 MHz, 298 K, DMSO-d_6): δ: 154.7 (CO), 154.2 (ArC), 144.6 (ArC), 138.2 (ArCH),

138.0-123.8 (q, *J* = 538.67 Hz, ArC), 135.4 (ArC), 133.9, (ArC), 126.5-119.2 (q, *J* = 31.68 Hz, CF_3), 126.3 (ArCH), 119.4 (ArCH), 117.6 (ArCH), 107.1 (ArCH), 56.4 (CH_2), 41.5 (CH_3); IR (film): ν = 3235 (NH stretch), 1601, 1200, 1179, 798; HRMS for the sulfonate-urea ion ($C_9H_8F_3N_2O_4S^-$) (ESI^-): m/z: act: 297.0157 $[M]^-$ cal: 297.0162 $[M]^-$.

Co-formulation *a*: Aminomethanesulfonic acid (0.22 g, 2.00 mM) was added to a stirring solution of 4-(trifluoromethyl)phenyl isocyanate (0.37 g, 2.00 mM) in pyridine (20 mL). The reaction was then refluxed at 60 °C under an inert atmosphere over night and filtered to give a white solid with a yield of 90 % (0.68 g, 1.80 mM). To the resultant compound (0.19 g, 0.50 mM) malachite green oxalate salt (0.21 g, 0.50 mM) was added in methanol (20 mL) and taken to dryness to give a blue/brown solid with a yield of 100 % (0.50 g, 0.50 mM); Melting Point: 133 °C; ^1H NMR (400 MHz, 298 K, DMSO-d_6): δ: 9.34 (s, 1H), 7.74 (t, *J* = 7.48 Hz, 1H), 7.61 (t, *J* = 7.60 Hz, 1H), 7.55 (d, *J* = 8.56 Hz, 2H), 7.44 (d, *J* = 8.60 Hz, 2H), 7.33-7.29 (m, 3H), 7.25-7.17 (m, 3H), 7.06 (d, *J* = 9.16 Hz, 2H), 6.97 (d, *J* = 8.84 Hz, 2H), 6.61 d, *J* = 8.92 Hz, 2H), 3.97 (d, *J* = 6.00 Hz, 2H), 3.28 (s, 6H), 3.19-3.14 (m, 8H), 2.84 (s, 6H), 1.60-1.52 (m, 8H), 1.34-1.26 (m, 8H), 0.92 (t, *J* = 7.36 Hz, 12H); $^{13}C\{^1H\}$ NMR (100 MHz, 298 K, DMSO-d_6): δ: 175.3 (tetra C), 162.9 (CO), 156.5 (CO), 154.3 (CO), 149.1 (ArC), 148.9 (ArC), 144.4 (ArC), 140.1 (ArCH), 139.3 (ArC), 136.3 (ArC), 134.2 (ArCH), 132.8 (ArCH), 128.6 (ArCH), 128.5 (ArCH), 127.7 (ArCH), 127.2 (ArCH), 126.5 (ArCH), 125.7-125.4 (q, *J* = 3.92 Hz, CF_3), 123.3 (ArC), 121.2-120.3 (q, *J* = 31.69 Hz, ArC), 117.2 (ArCH), 114.0 (ArCH), 111.4 (ArCH), 57.6 (CH_2), 55.9 (CH_2), 40.5 (CH_3), 40.2 (CH_3), 23.1 (CH_2), 19.2 (CH_2), 13.5 (CH_3); HRMS for the sulfonate-urea ion ($C_9H_8F_3N_2O_4S^-$) (ESI^-): m/z: act: 297.0156 $[M]^-$ cal: 297.0162 $[M]^-$.

Co-formulation *b*: Aminomethanesulfonic acid (0.23 g, 2.10 mM) was added to tetrabutylammonium hydroxide in methanol (2.08 mL, 2.10 mM) and taken to dryness. Triphosgene (0.31 g, 1.00 mM) was added to a stirring solution of 4-(6-methylbenzothiazol)aniline (0.50 g, 2.00 mM) in ethyl acetate (30 mL) and the mixture was then refluxed at 80 °C for 4 hours. The tetrabutylammonium salt was then dissolved in ethyl acetate (10 mL) and added to the reaction mixture and refluxed overnight. The organic solvent was then decanted and additional ethyl acetate (20 mL) added to the oil, sonicated for one hour to give the pure product as a pale yellow solid with a yield of 95 % (1.17 g, 1.90 mM). To the resultant compound (0.31 g, 0.50 mM) malachite green oxalate salt (0.21 g, 0.50 mM) was added in methanol (20 mL) and taken to dryness to give a blue/brown solid with a yield of 100 % (0.54 g, 0.50 mM); Melting Point: 128 °C; ^1H NMR (400 MHz, 298 K, DMSO-d_6): δ: 9.27 (s, 1H), 7.89-7.56 (m, 8H), 7.31-7.20 (m, 6H), 7.05-6.61 (m, 6H), 3.97 (d, *J* = 5.92 Hz, 2H), 3.28 (s, 8H), 3.17-3.13 (m, 8H), 2.84 (s, 4H), 2.43 (s, 3H), 1.59-1.51 (m, 8H), 1.34-1.25 (m, 8H), 0.93 (t, *J* = 7.28 Hz, 12H); $^{13}C\{^1H\}$ NMR (100 MHz, 298 K, DMSO-d_6): δ: 175.3 (tetra C), 166.2 (CO), 162.9 (CO), 156.4 (CO), 154.3 (ArC), 151.9 (ArC), 149.1 (ArC), 148.9 (ArC), 143.6 (ArC), 140.1 (ArCH), 139.3 (ArC), 136.3 (ArC), 134.7 (ArC), 134.3 (ArCH), 132.9 (ArCH), 132.2 (ArCH), 128.7 (ArCH), 128.5 (ArCH), 127.9 (ArCH), 127.8 (ArCH), 127.8 (ArCH), 127.7 (ArCH), 127.2 (ArCH), 126.5 (ArCH), 125.6 (ArC), 122.0 (ArCH), 121.7 (ArCH), 117.6 (ArCH), 114.0 (ArCH), 111.4 (ArCH), 57.5 (CH_2), 56.0 (CH_2), 40.5 (CH_3), 40.3 (CH_3), 23.1 (CH_2), 21.1 (CH_3), 19.3 (CH_2), 13.6 (CH_3); HRMS for the sulfonate-urea ion ($C_{16}H_{14}N_3O_4S_2^-$) (ESI^-): m/z: act: 376.0402 $[M]^-$ cal: 376.0432 $[M]^-$.

Co-formulation *c*: Aminomethanesulfonic acid (0.22 g, 2.00 mM) was added to a stirring solution of 4-(trifluoromethyl)phenyl isocyanate (0.37 g, 2.00 mM) in pyridine (20 mL). The reaction was then refluxed at 60 °C under an inert atmosphere over night and filtered to give a white solid with a yield of 90 % (0.68 g, 1.80 mM). To the resultant compound (0.19 g, 0.50 mM) methylene blue chloride (0.17 g, 0.50 mM) was added in methanol (20 mL) and taken to dryness to give a blue solid with a yield of 100 % (0.43 g, 0.50 mM); Melting Point: 120 °C; ^1H NMR (400 MHz, 298 K, DMSO-d_6): δ: 9.44 (s, 1H), 7.75 (bs, 1H), 7.56 (d, *J* = 8.80 Hz, 2H), 7.44 (d, *J* = 8.88 Hz, 2H) 7.40-7.36 (m, 2H), 7.14 (t, *J* = 6.04 Hz, 1H), 3.98 (d, *J* = 6.08 Hz, 2H), 3.38 (s, 12H), 3.19-3.15 (m, 8H), 1.61-1.53 (m, 8H), 1.35-1.28 (m, 8H), 0.93 (t, *J* = 7.28 Hz, 12H); $^{13}C\{^1H\}$ NMR (100 MHz, 298 K, DMSO-d_6): δ: 154.2 (CO), 144.3 (ArC), 137.6 (ArC), 128.7 (ArC), 126.0 (ArC), 125.6 (q, *J* = 3.93 Hz, ArC), 123.3 (ArCH), 121.2-120.2 (q, *J* = 31.68 Hz,

CF_3), 120.6 (ArC), 118.8 (ArC), 117.1 (ArCH), 106.5 (ArC), 57.5 (CH_2), 55.9 (CH_2), 41.0 (CH_3), 23.1 (CH_2), 19.2 (CH_2), 13.5 (CH_3); IR (film): ν = 3265 (NH stretch), 1671, 1312, 1099, 821; HRMS for the sulfonate-urea ion ($C_9H_8F_3N_2O_4S^-$) (ESI$^-$): m/z: act: 297.0282 $[M]^-$ cal: 297.0162 $[M]^-$.

Co-formulation *d*: Aminomethanesulfonic acid (0.23 g, 2.10 mM) was added to tetrabutylammonium hydroxide in methanol (2.08 mL, 2.10 mM) and taken to dryness. Triphosgene (0.31 g, 1.00 mM) was added to a stirring solution of 4-(6-methylbenzothiazol)aniline (0.50 g, 2.00 mM) in ethyl acetate (30 mL) and the mixture was then refluxed at 80 °C for 4 hours. The tetrabutylammonium salt was then dissolved in ethyl acetate (10 mL) and added to the reaction mixture and refluxed overnight. The organic solvent was then decanted and additional ethyl acetate (20 mL) added to the oil, sonicated for one hour to give the pure product as a pale yellow solid with a yield of 95 % (1.17 g, 1.90 mM). To the resultant compound (0.31 g, 0.50 mM) methylene blue chloride (0.19 g, 0.50 mM) was added in methanol (20 mL) and taken to dryness to give a blue solid with a yield of 100 % (0.47 g, 0.50 mM); Melting Point: 128 °C; ^{1}H NMR (400 MHz, 298 K, DMSO-d_6): δ: 9.34 (s, 0.5H), 7.89-7.69 (m, 3.5H), 3.95 (s, 1H), 3.46-3.36 (m, 7H), 2.45 (s, 1H), 1.56-0.91 (m, 28H); HRMS for the sulfonate-urea ion ($C_{16}H_{14}N_3O_4S_2^-$) (ESI$^-$): m/z: act: 376.0408 $[M]^-$ cal: 376.0432 $[M]^-$.

Co-formulation *e*: Coumarin-3-carboxylic acid (0.38 g, 2.0 mM) was added to compound **2** (0.98 g, 2.0 mM) in methanol and taken to dryness to give a dark orange solid with a yield of 100 % (1.35 g, 2.0 mM); ^{1}H NMR (400 MHz, 298 K, DMSO-d_6): δ: 8.73 (s, 1H), 8.27 (s, 1H), 7.91 (d, *J* = 7.72 Hz, 1H), 7.72 (t, *J* = 7.16 Hz, 1H), 7.42 (m, 2H), 7.00 (d, *J* = 8.72 Hz, 2H), 6.46 (d, *J* = 8.64 Hz, 2H), 6.27 (t, *J* = 5.04 Hz, 1H), 3.83 (d, *J* = 5.76 Hz, 2H), 3.15 (t, *J* = 8.72 Hz, 8H), 1.55 (m, 8H), 1.30 (m, 8H), 0.92 (t, *J* = 7.36 Hz, 12H); $^{13}C\{^1H\}$ NMR (100 MHz, 298 K, DMSO-d_6): δ: 164.5 (CO), 157.2 (CO), 155.4 (CO), 154.9 (CO), 148.6 (ArC), 143.3 (ArC), 134.7 (ArCH), 130.6 (ArC), 130.4 (ArCH), 125.3 (ArCH), 120.2 (ArCH), 119.1 (ArC), 118.5 (ArC), 116.6 (ArCH), 114.9 (ArCH), 57.9 (CH_2), 56.7 (CH_2), 25.5 (CH_2), 19.7 (CH_2), 14.0 (CH_3).

Co-formulation *f*: Coumarin-3-carboxylic acid (0.38 g, 2.0 mM) was added to compound **3** (1.09 g, 2.0 mM) in methanol and taken to dryness to give a brown solid with a yield of 100 % (1.47 g, 2.0 mM); ^{1}H NMR (400 MHz, 298 K, DMSO-d_6): δ: 8.71 (s, 1H), 8.44 (s, 1H), 7.90 (d, *J* = 6.88 Hz, 1H), 7.72 (t, *J* = 8.44 Hz, 1H), 7.41 (m, 2H), 7.18 (d, *J* = 8.92 Hz, 2H), 6.62 (d, *J* = 8.80 Hz, 2H), 6.45 (t, *J* = 5.88 Hz, 1H), 3.90 (d, *J* = 5.88 Hz, 2H), 3.20 (m, 12H), 1.56 (m, 8H), 1.31 (m, 8H), 1.03 (t, *J* = 6.96 Hz, 6H), 0.93 (t, *J* = 7.36 Hz, 12H); $^{13}C\{^1H\}$ NMR (100 MHz, 298 K, DMSO-d_6): δ: 164.5 (CO), 157.2 (CO), 155.4 (CO), 154.9 (ArC), 148.5 (ArCH), 142.7 (ArC), 134.7 (ArCH), 130.9 (ArC), 130.6 (ArCH), 125.3 (ArCH), 120.3 (ArCH), 119.2 (ArC), 118.5 (ArC), 116.6 (ArCH), 113.9 (ArCH), 58.0 (CH_2), 56.8 (CH_2), 44.9 (CH_2), 23.5 (CH_2), 19.7 (CH_2), 13.9 (CH_3), 12.7 (CH_3).

Co-formulation *g*: Salicylic acid (0.28 g, 2.0 mM) was added to compound **2** (0.98 g, 2.0 mM) in methanol and taken to dryness to give a dark orange solid with a yield of 100 % (1.25 g, 2.0 mM); ^{1}H NMR (400 MHz, 298 K, DMSO-d_6): δ: 8.54 (s, 1H), 7.76 (dd, *J* = 6.24 Hz, 1H), 7.39 (td, *J* = 5.44 Hz, 1H), 7.16 (d, *J* = 8.80 Hz, 2H), 6.86-6.81 (m, 2H), 6.69 (d, *J* = 8.80 Hz, 2H), 6.56 (t, *J* = 6.08 Hz, 1H), 3.93 (d, *J* = 6.04 Hz, 2H), 3.13 (t, *J* = 8.52 Hz, 8H), 1.54 (m, 8H), 1.29 (m, 8H), 0.91 (t, *J* = 7.44 Hz, 12H); $^{13}C\{^1H\}$ NMR (100 MHz, 298 K, DMSO-d_6): δ: 171.9 (CO), 169.2 (CO), 167.7 (CO), 166.0 (CO), 163.1 (ArC), 155.0 (ArC), 154.6 (ArC), 150.2 (ArC), 136.0 (ArC), 134.2 (ArCH), 133.8 (ArCH), 133.0 (ArC), 131.4 (ArCH), 130.1 (ArCH), 126.1 (ArCH), 124.1 (ArC), 123.8 (ArCH), 119.7 (ArCH), 119.5 (ArCH), 118.1 (ArCH), 117.8 (ArCH), 116.6 (ArCH), 115.7 (ArCH), 115.3 (ArC), 57.5 (CH_2), 56.3 (CH_2), 56.1 (CH_2), 23.8 (CH_3), 23.1 (CH_2), 21.0 (CH_3), 19.2 (CH_2), 13.5 (CH_3).

Co-formulation *h*: Salicylic acid (0.28 g, 2.0 mM) was added to compound **3** (1.09 g, 2.0 mM) in methanol and taken to dryness to give a brown solid with a yield of 100 % (1.36 g, 2.0 mM); ^{1}H NMR (400 MHz, 298 K, DMSO-d_6): δ: 8.56 (s, 1H), 7.77 (d, *J* = 7.76 Hz, 1H), 7.44 (t, *J* = 8.36 Hz, 1H), 7.24 (d, *J* = 8.88 Hz, 2H), 6.84 (m, 4H), 6.46 (t, *J* = 5.88 Hz, 1H), 3.88 (d, *J* = 5.88 Hz, 2H), 3.20 (m, 12H), 1.56 (m, 8H), 1.30 (m, 8H), 0.95 (m, 18H); $^{13}C\{^1H\}$ NMR (100 MHz, 298 K, DMSO-d_6): δ: 172.5 (CO), 161.9 (CO),

155.2 (CO), 138.7 (ArC), 135.0 (ArCH), 134.4 (ArC), 130.7 (ArCH), 119.9 (ArCH), 118.9 (ArCH), 117.2 (ArCH), 117.0 (ArCH), 115.4 (ArC), 58.0 (CH_2), 56.6 (CH_2), 47.6 (CH_2), 23.5 (CH_2), 19.6 (CH_2), 13.9 (CH_3), 11.9 (CH_3).

Co-formulation *i*: Acetylsalicylic acid (0.36 g, 2.0 mM) was added to compound **2** (0.98 g, 2.0 mM) in methanol and taken to dryness to give a dark orange solid with a yield of 100 % (1.34 g, 2.0 mM); ^{1}H NMR (400 MHz, 298 K, DMSO-d_6): δ: 8.36 (s, 1H), 7.92 (d, J = 7.72 Hz, 1H), 7.63 (t, J = 7.84 Hz, 1H), 7.37 (t, J = 7.56 Hz, 1H), 7.18 (t, J = 8.00 Hz 1H), 7.03 (d, J = 8.28 Hz, 2H), 6.48 (d, J = 8.24 Hz, 2H), 6.43 (t, J = 5.64 Hz, 1H), 3.89 (d, J = 5.72 Hz, 2H), 3.13 (t, J = 8.16 Hz, 8H), 2.24 (s, 3H), 1.54 (m, 8H), 1.29 (m, 8H), 0.92 (t, J = 7.24 Hz, 12H); ^{13}C{^{1}H} NMR (100 MHz, 298 K, DMSO-d_6): δ: 172.1 (CO), 169.3 (CO), 167.7 (CO), 165.7 (CO), 161.5 (CO), 154.8 (CO), 154.7 (ArC), 150.2 (ArC), 136.0 (ArC), 135.0 (ArCH), 134.2 (ArC), 134.2 (ArC), 133.9 (ArCH), 133.1 (ArC), 131.4 (ArCH), 130.3 (ArCH), 126.1 (ArCH), 124.1 (ArC), 123.8 (ArCH), 119.5 (ArCH), 119.2 (ArCH), 118.7 (ArCH), 118.0 (ArCH), 117.9 (ArCH), 117.0 (ArCH), 114.3 (ArC), 57.5 (CH_2), 56.1 (CH_2), 23.9 (CH_3), 23.1 (CH_2), 21.0 (CH_3), 19.2 (CH_2), 13.6 (CH_3).

Co-formulation *j*: Acetylsalicylic acid (0.36 g, 2.0 mM) was added to compound **3** (1.09 g, 2.0 mM) in methanol and taken to dryness to give a brown solid with a yield of 100 % (1.45 g, 2.0 mM); ^{1}H NMR (400 MHz, 298 K, DMSO-d_6): δ: 8.43 (s, 1H), 7.92 (d, J = 7.80 Hz, 1H), 7.62 (t, J = 7.60 Hz, 1H), 7.37 (t, J = 7.64 Hz, 1H), 7.17 (m, 3H), 6.60 (d, J = 8.84 Hz, 2H), 6.42 (t, J = 5.88 Hz, 1H), 3.89 (d, J = 5.88 Hz, 2H), 3.20 (m, 12H), 1.56 (m, 8H), 1.31 (m, 8H), 1.03 (t, J = 6.96 Hz, 6H), 0.93 (t, J = 7.36 Hz, 12H); ^{13}C{^{1}H} NMR (100 MHz, 295 K, DMSO-d_6): δ: 169.4 (CO), 165.8 (CO), 155.2 (CO), 150.4 (CO), 134.0 (ArCH), 131.6 (ArCH), 126.3 (ArCH), 124.3 (CO), 124.0 (ArCH), 120.0 (ArCH), 113.6 (ArCH), 57.7 (CH_2), 56.5 (CH_2), 44.6 (CH_2), 23.3 (CH_2), 21.1 (CH_3), 19.4 (CH_2), 13.7 (CH_3), 12.5 (CH_3).

NMR

Characterisation NMR

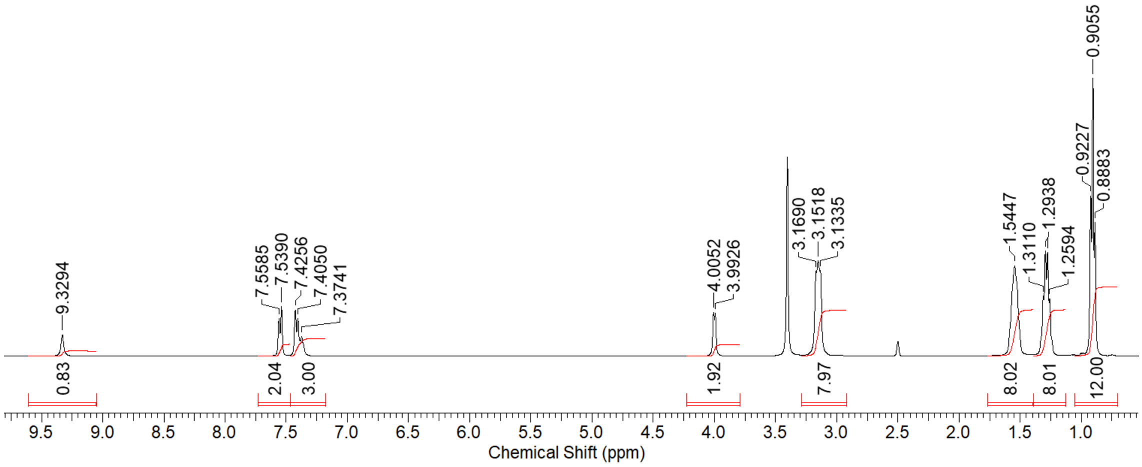

Figure S3 – ^{1}H NMR of compound **1** in DMSO-d_6 conducted at 298 K.

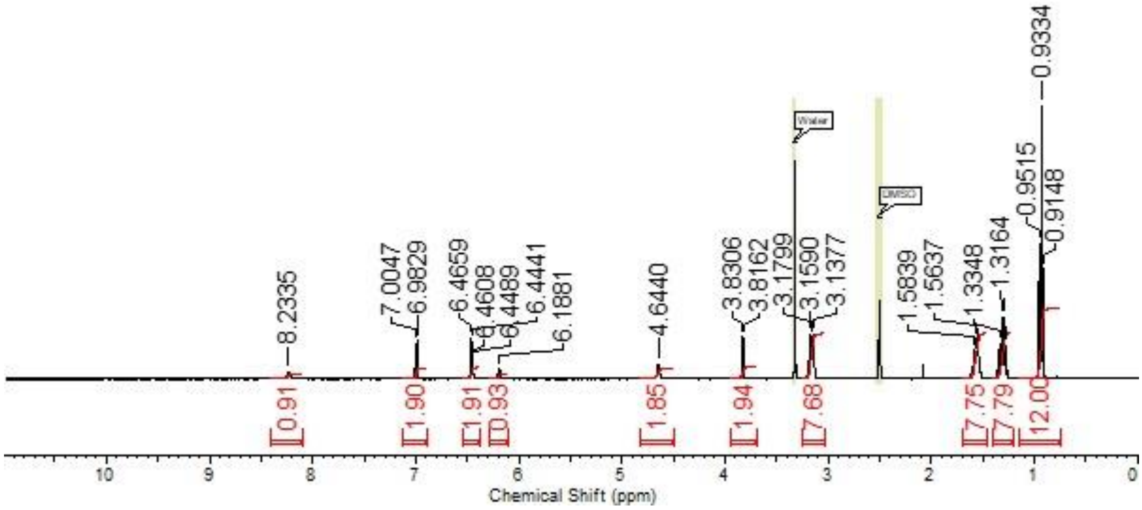

Figure S4 - ^{1}H NMR of compound **2** in DMSO-d_6 conducted at 298 K.

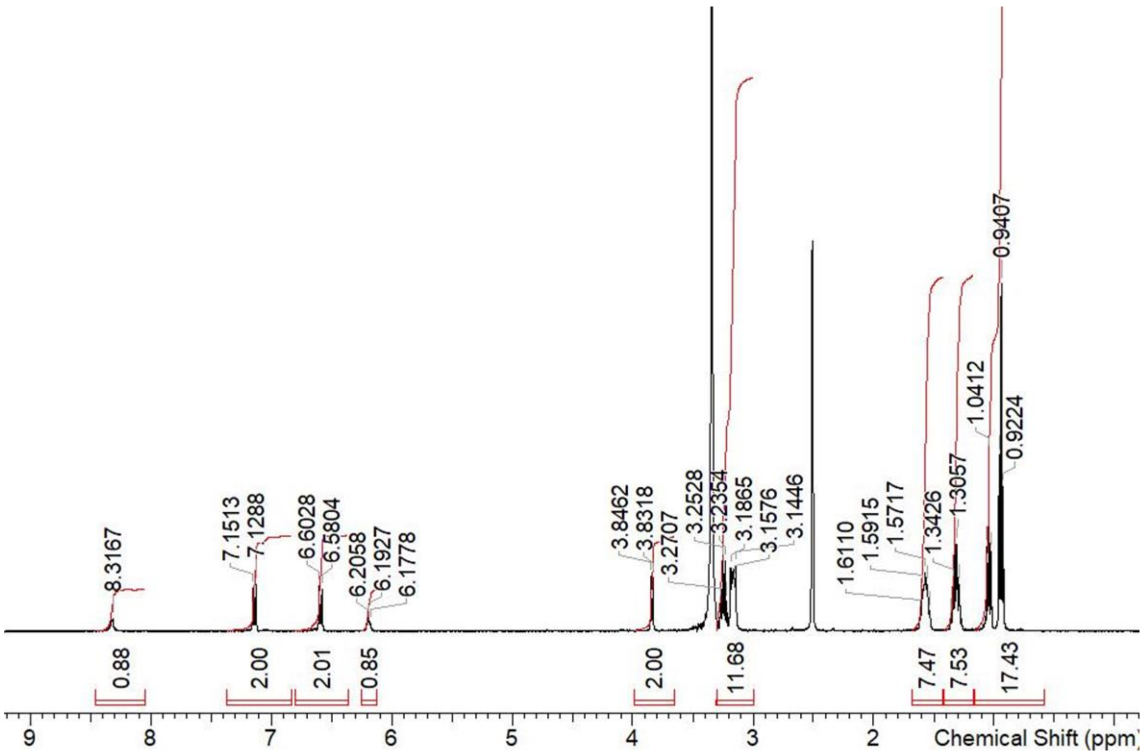

Figure S5 - ^{1}H NMR of compound **3** in DMSO-d_6 conducted at 298 K.

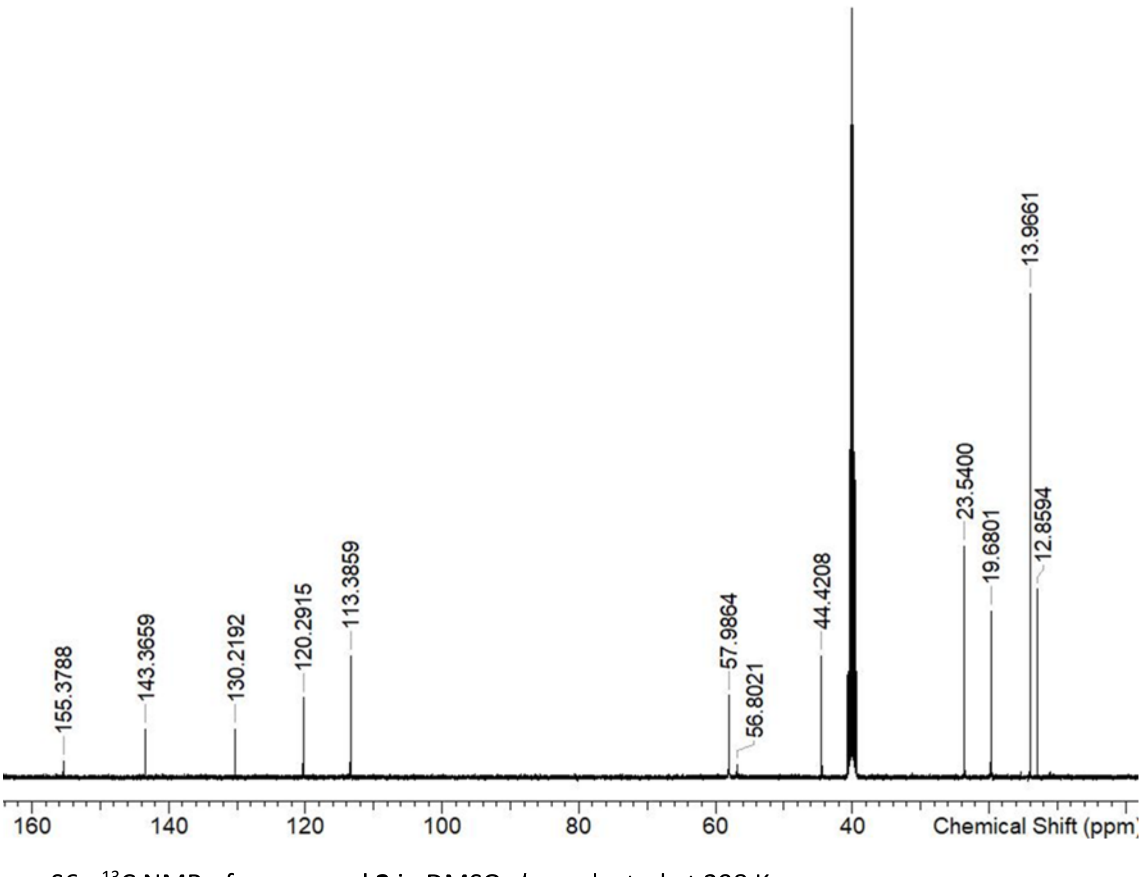

Figure S6 - ^{13}C NMR of compound **3** in DMSO-d_6 conducted at 298 K.

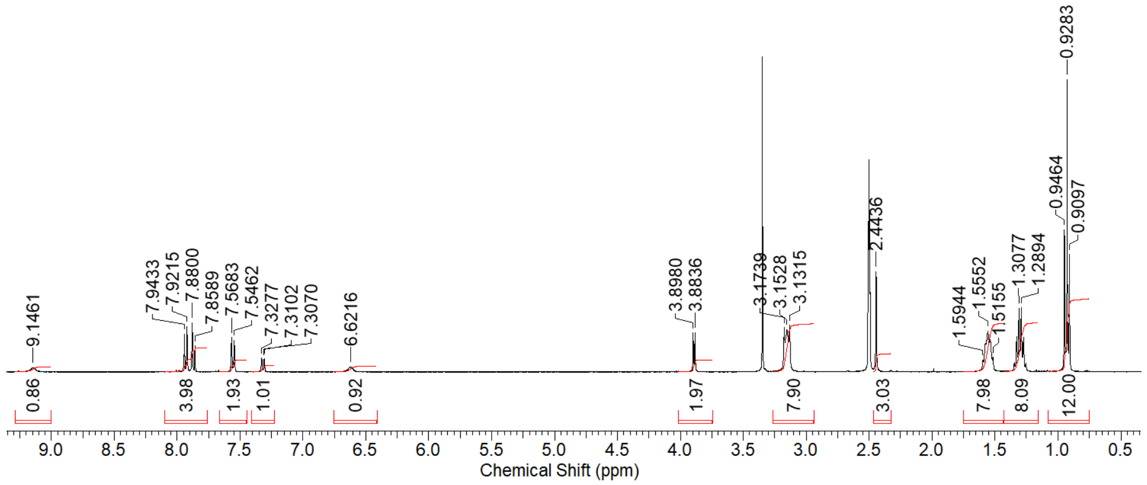

Figure S7 - ^{1}H NMR of compound **4** in DMSO-d_6 conducted at 298 K.

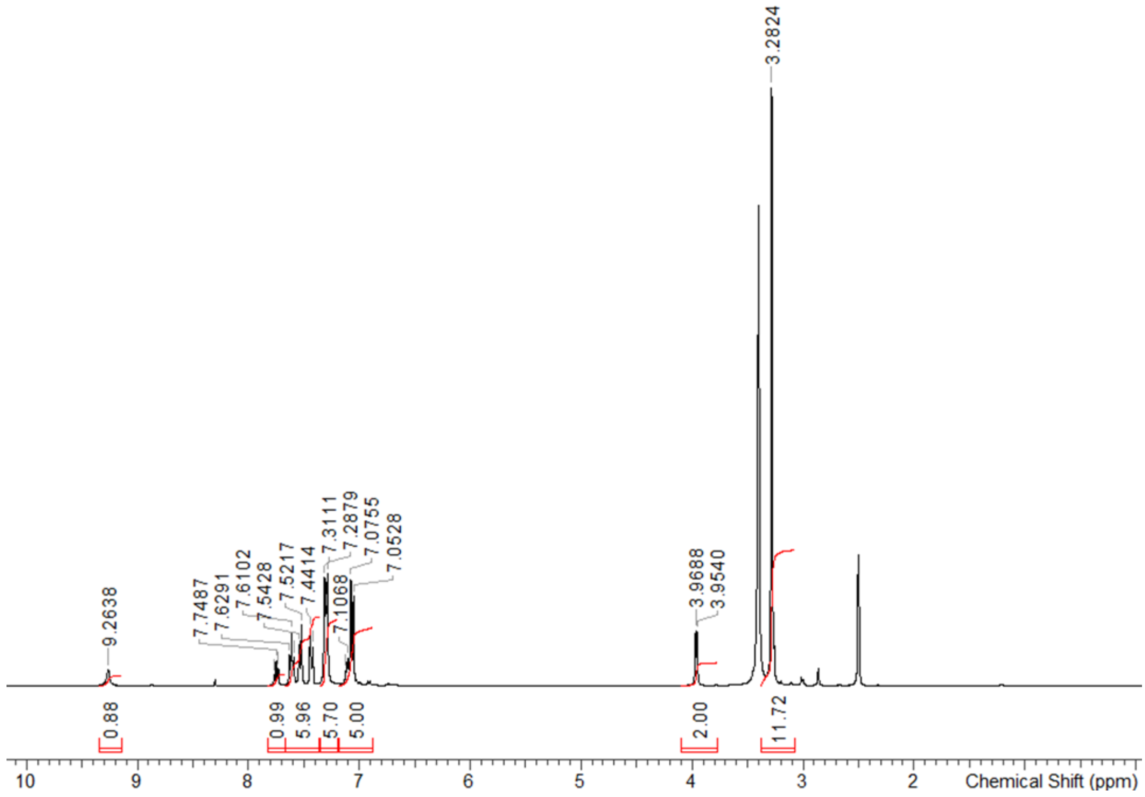

Figure S8 - ^{1}H NMR of compound **10** in DMSO-d_6 conducted at 298 K.

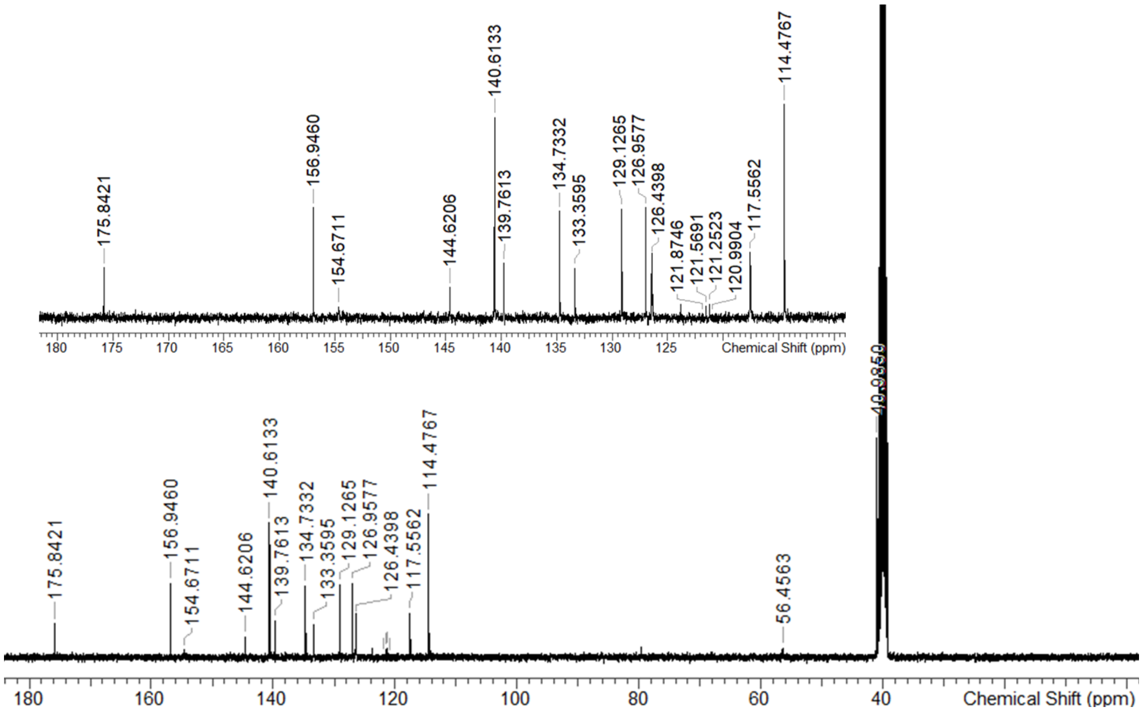

Figure S9 - ^{13}C NMR of compound **10** in DMSO-d_6 conducted at 298 K.

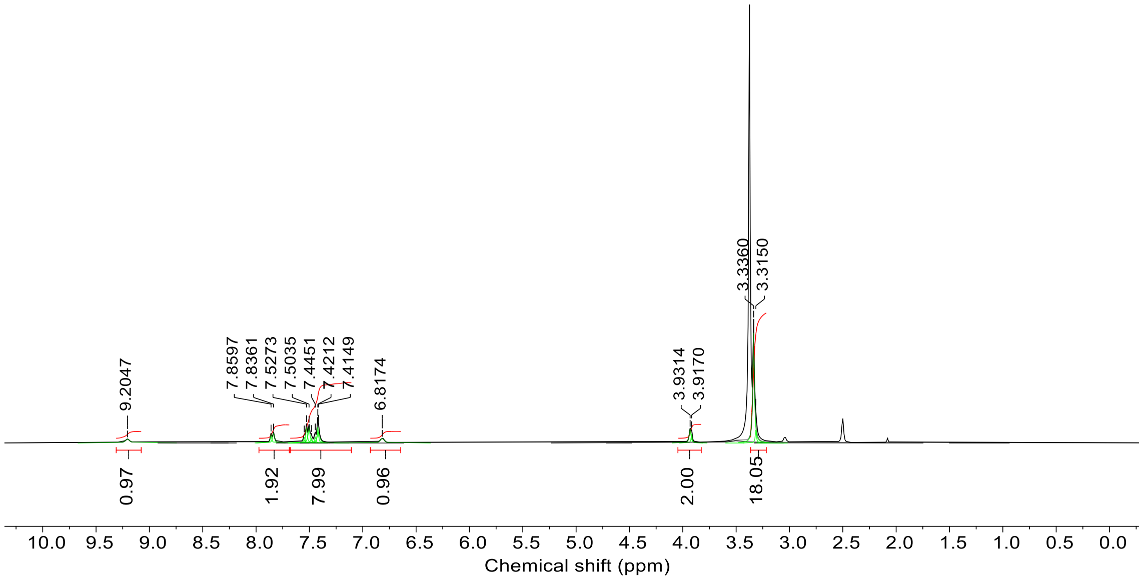

Figure S10 - ^{1}H NMR of compound **11** in DMSO-d_6 conducted at 298 K.

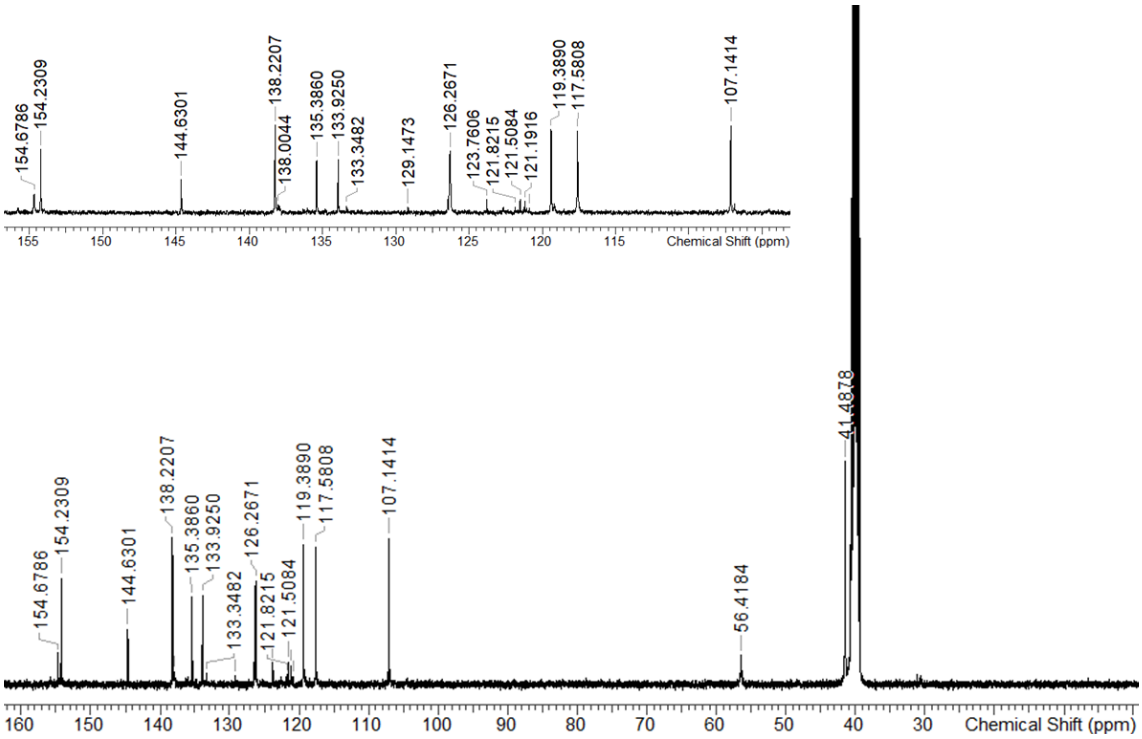

Figure S11 - ^{13}C NMR of compound **11** in DMSO-d_6 conducted at 298 K.

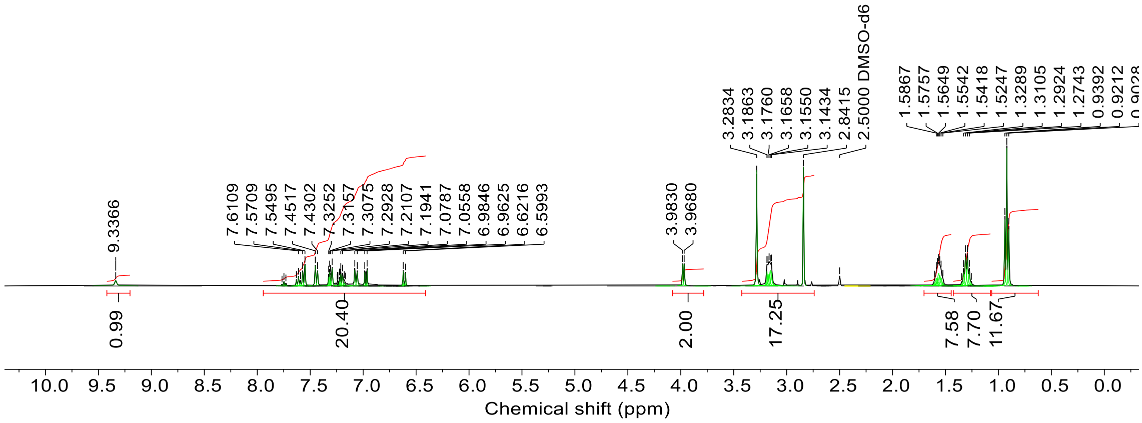

Figure S12 - ^{1}H NMR of **Co-formulation *a*** in DMSO-d_6 conducted at 298 K.

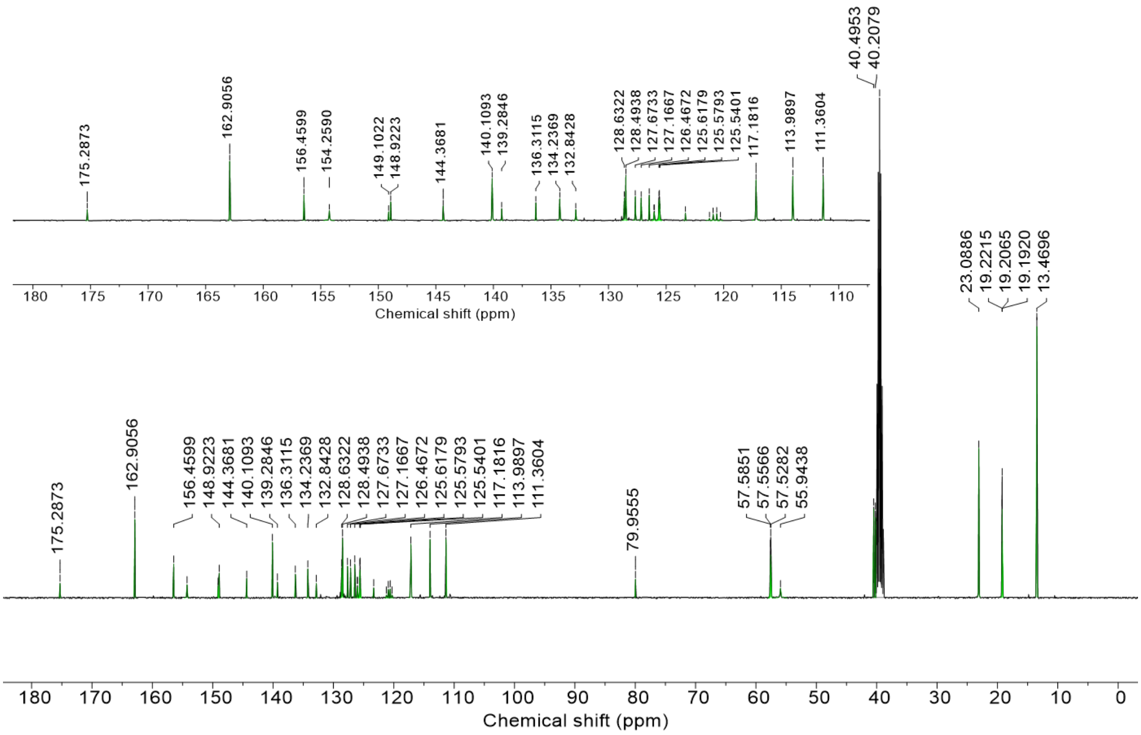

Figure S13 - ^{13}C NMR of co-formulation ***a*** in DMSO-d_6 conducted at 298 K.

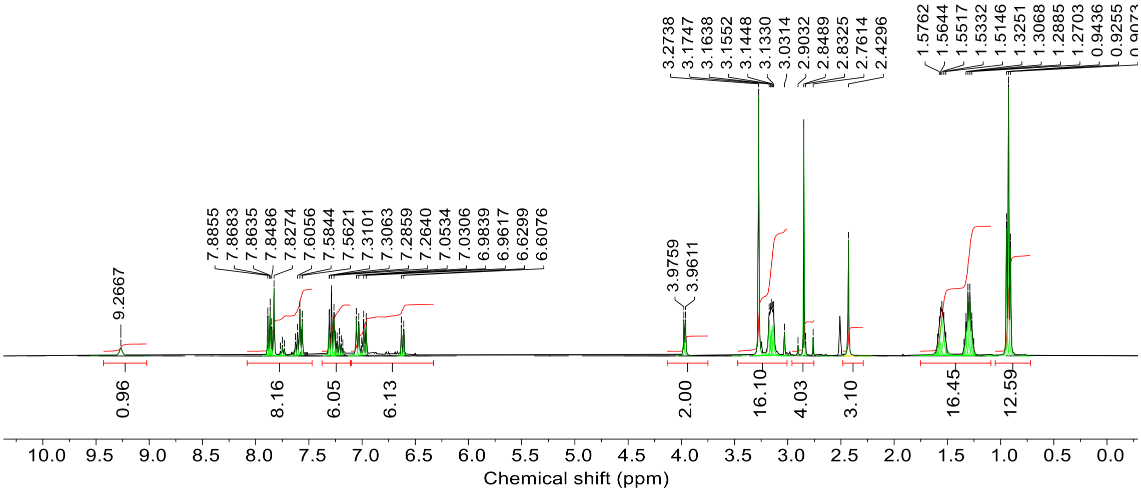

Figure S14 - ^{1}H NMR of co-formulation ***b*** in DMSO-d_6 conducted at 298 K.

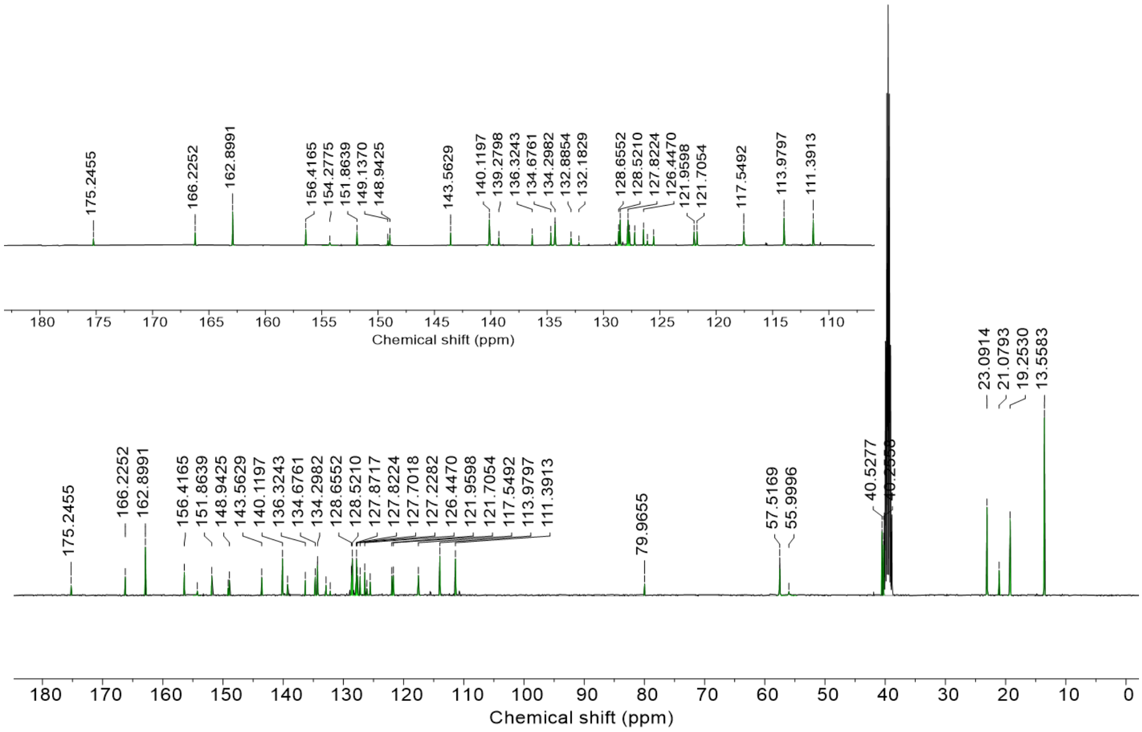

Figure S15 - ^{13}C NMR of co-formulation ***b*** in DMSO-d_6 conducted at 298 K.

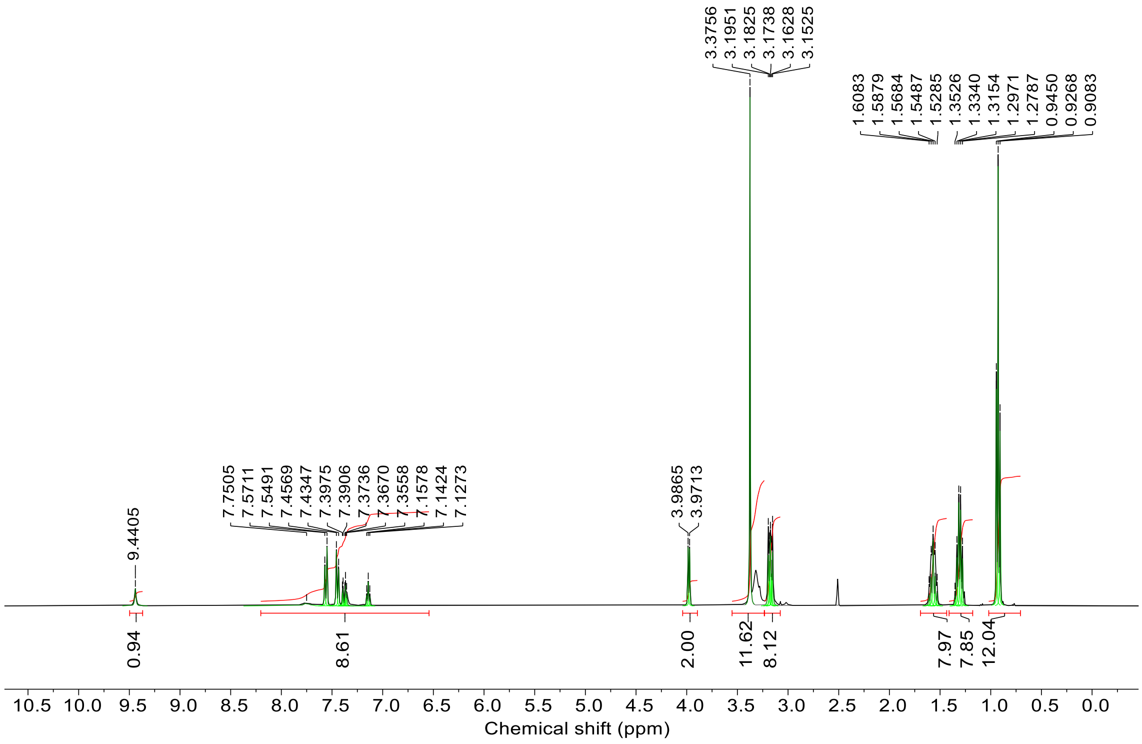

Figure S16 - ^{1}H NMR of co-formulation ***c*** in DMSO-d_6 conducted at 298 K.

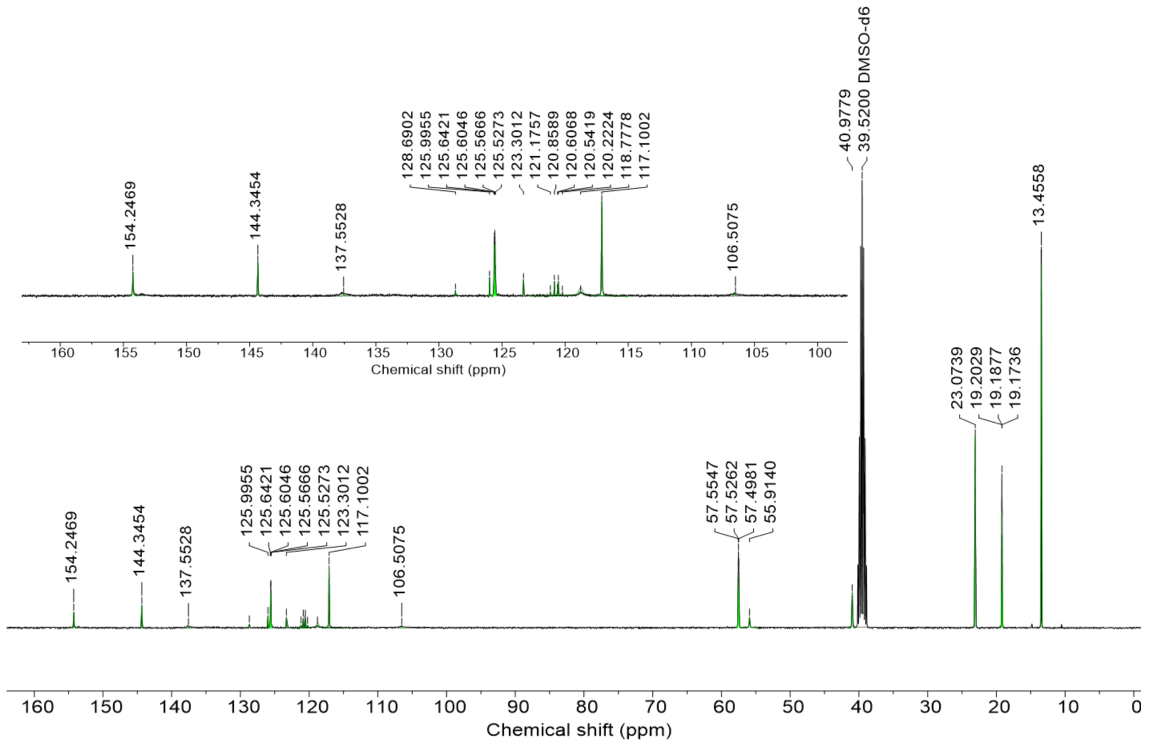

Figure S17 - ^{13}C NMR of co-formulation ***c*** in DMSO-d_6 conducted at 298 K.

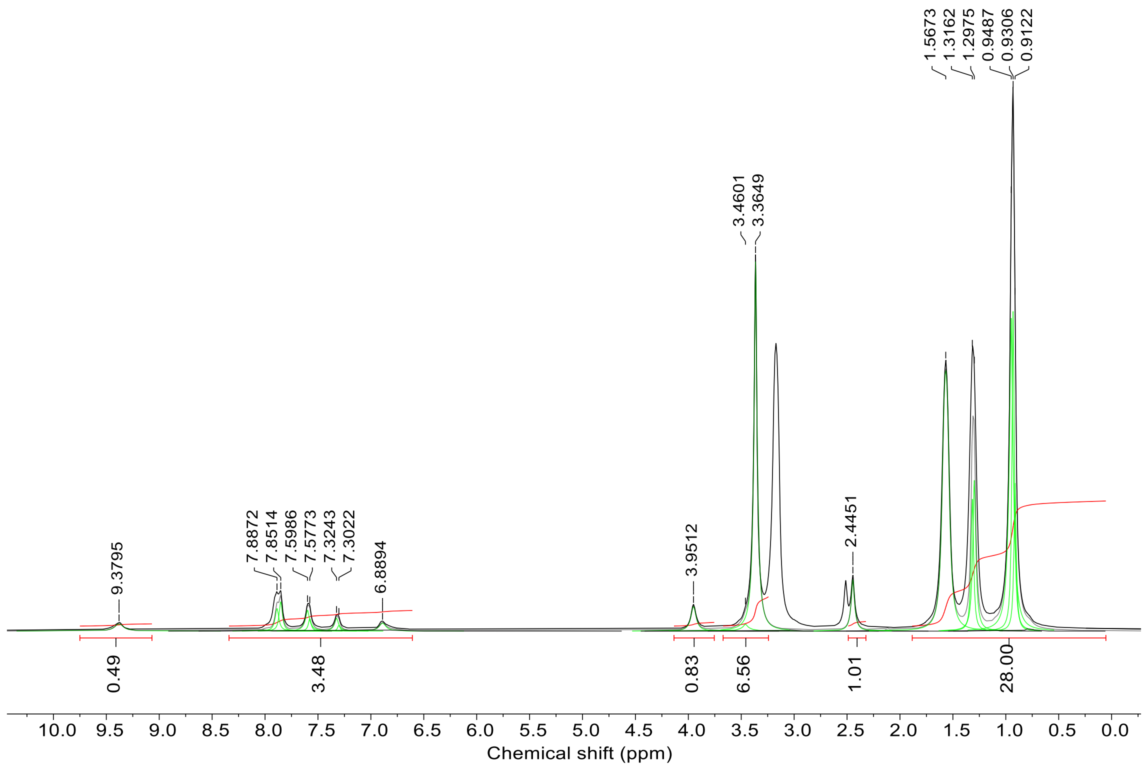

Figure S18 - ^{1}H NMR of co-formulation ***d*** in DMSO-d_6 conducted at 298 K.

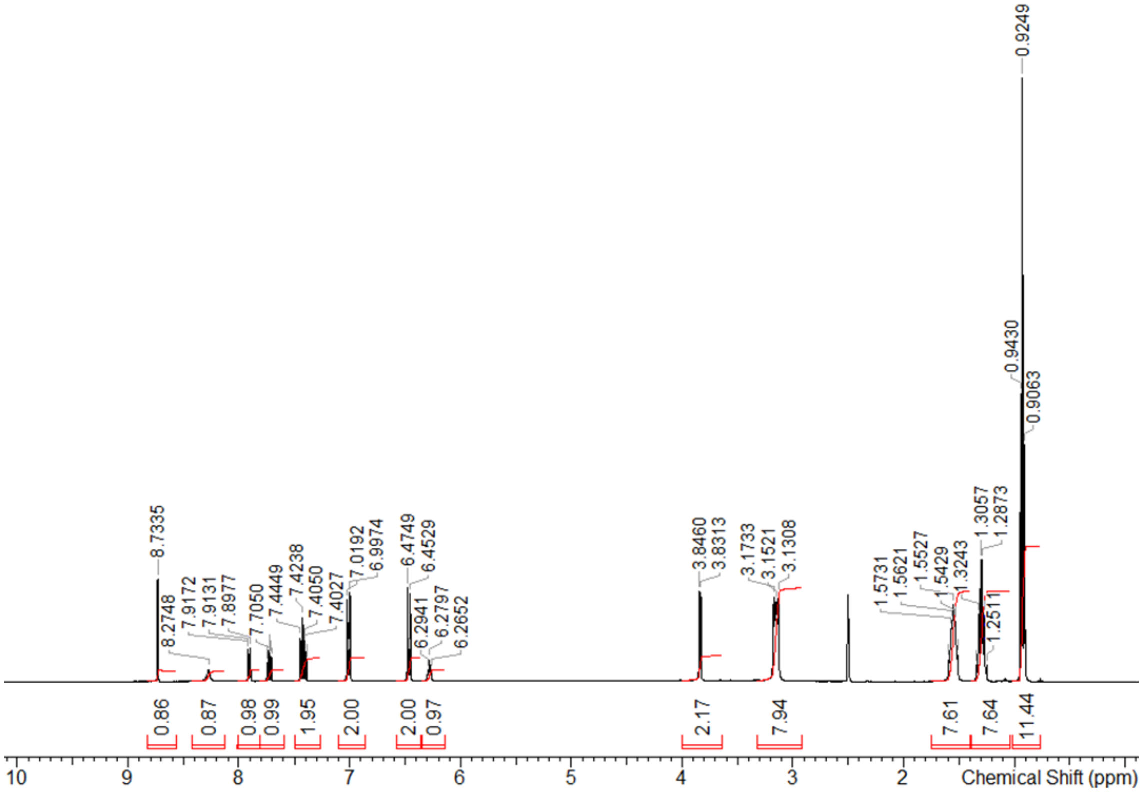

Figure S19 - ^{1}H NMR of co-formulation ***e*** in DMSO-d_6 conducted at 298 K.

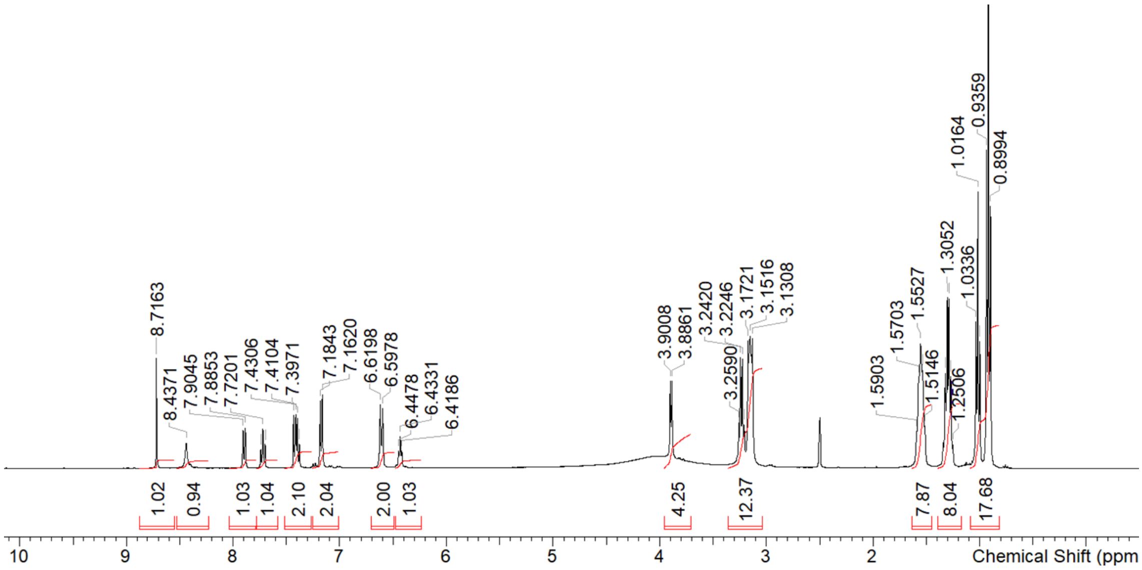

Figure S20 - ^{1}H NMR of co-formulation ***f*** in DMSO-d_6 conducted at 298 K.

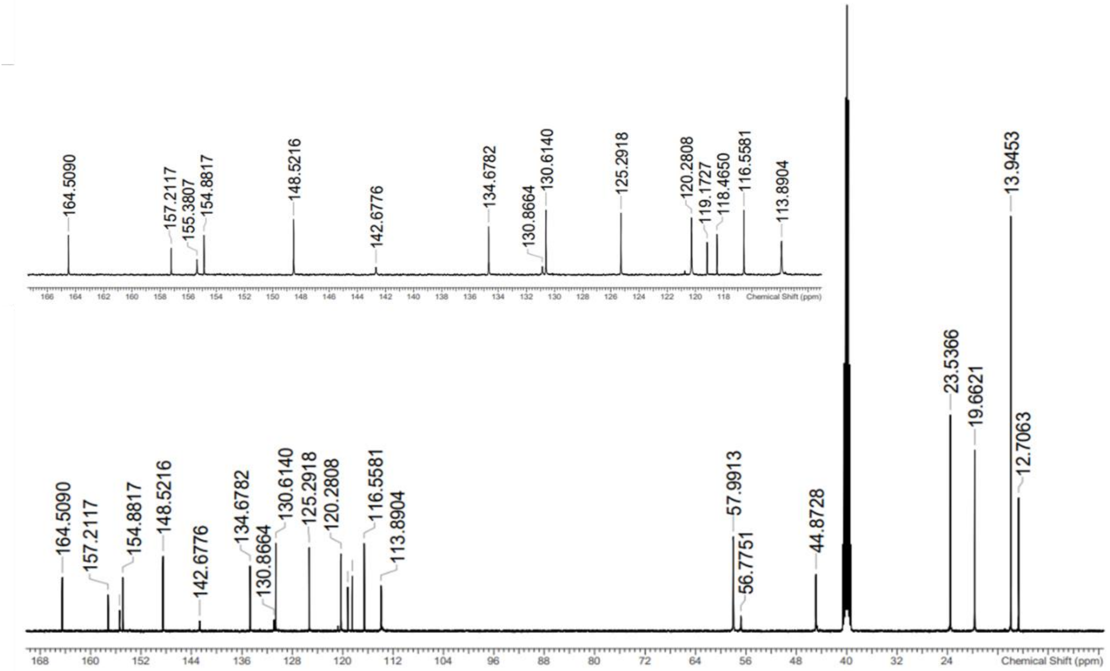

Figure S21 - ^{13}C NMR co-formulation ***f*** in DMSO-d_6 conducted at 298 K.

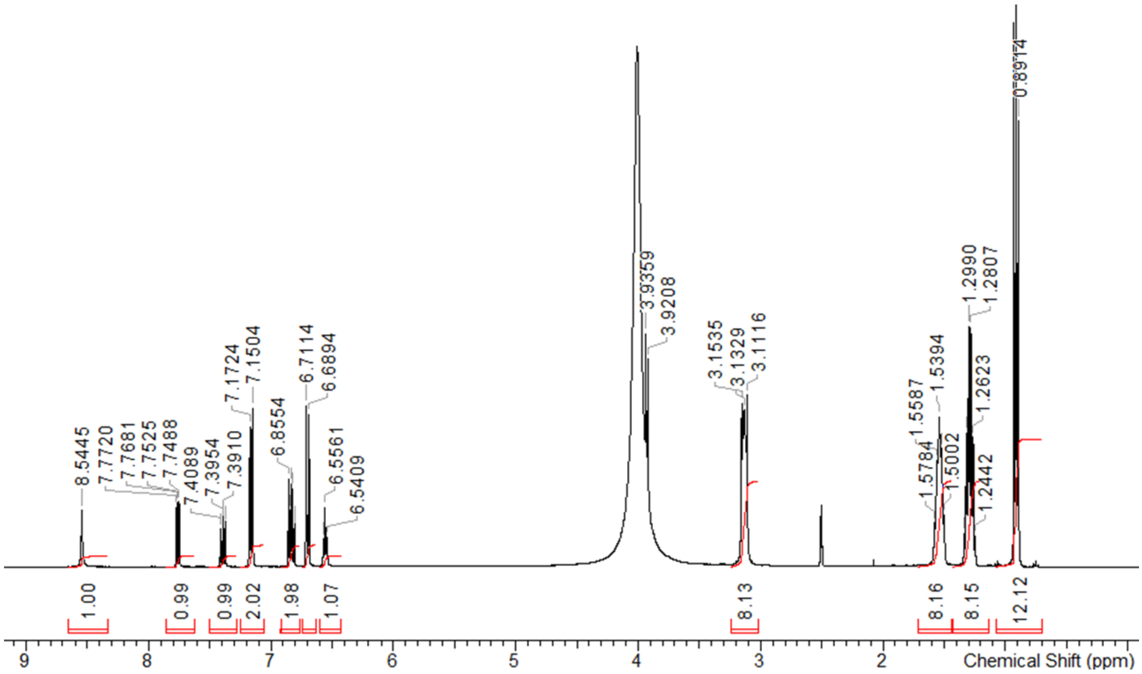

Figure S22 - ^{1}H NMR of co-formulation ***g*** in DMSO-d_6 conducted at 298 K.

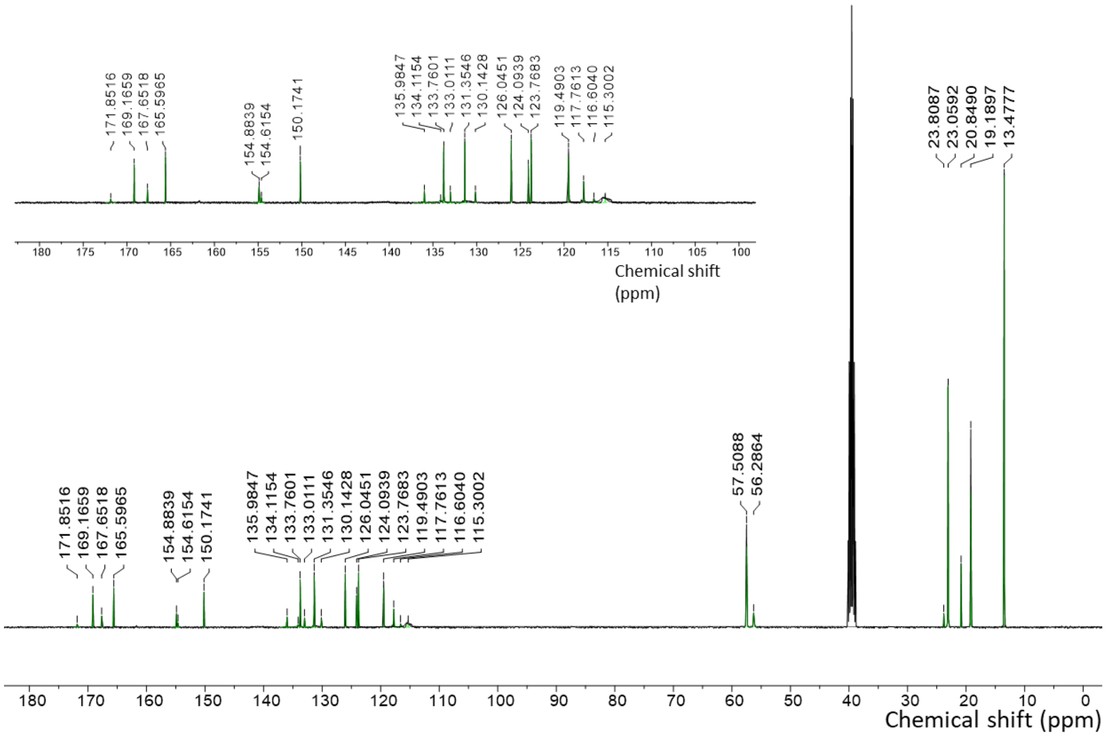

Figure S23 - ^{13}C NMR of co-formulation ***g*** in DMSO-d_6 conducted at 298 K.

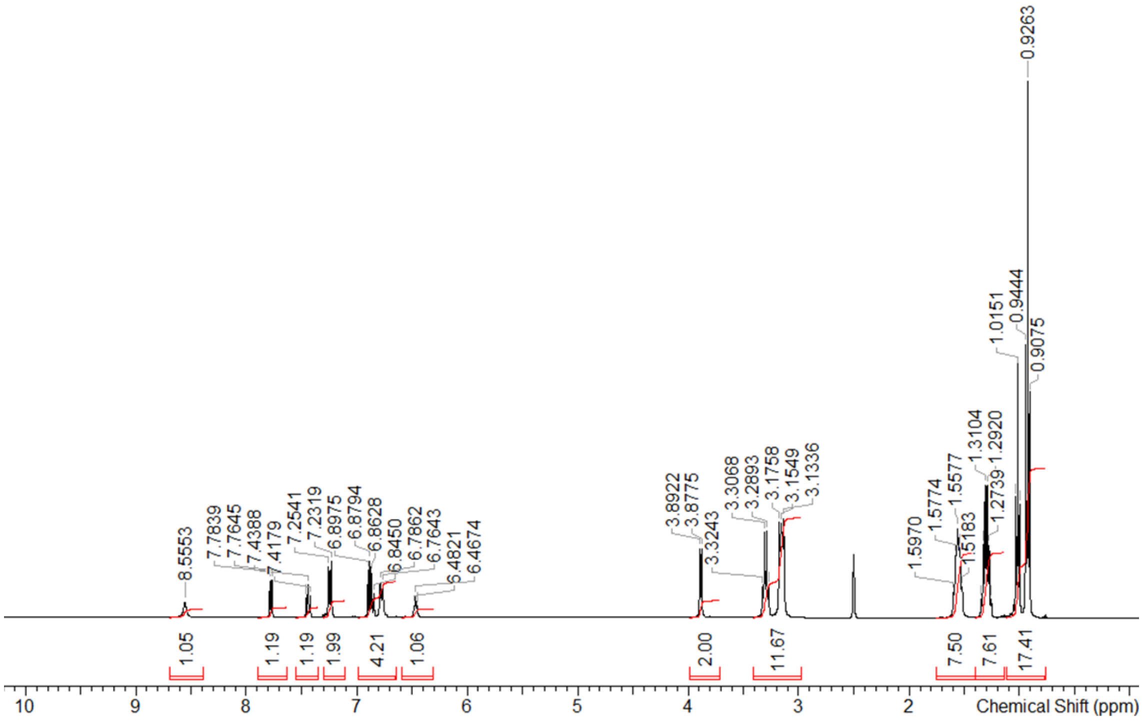

Figure S24 - ^{1}H NMR of co-formulation ***h*** in DMSO-d_6 conducted at 298 K.

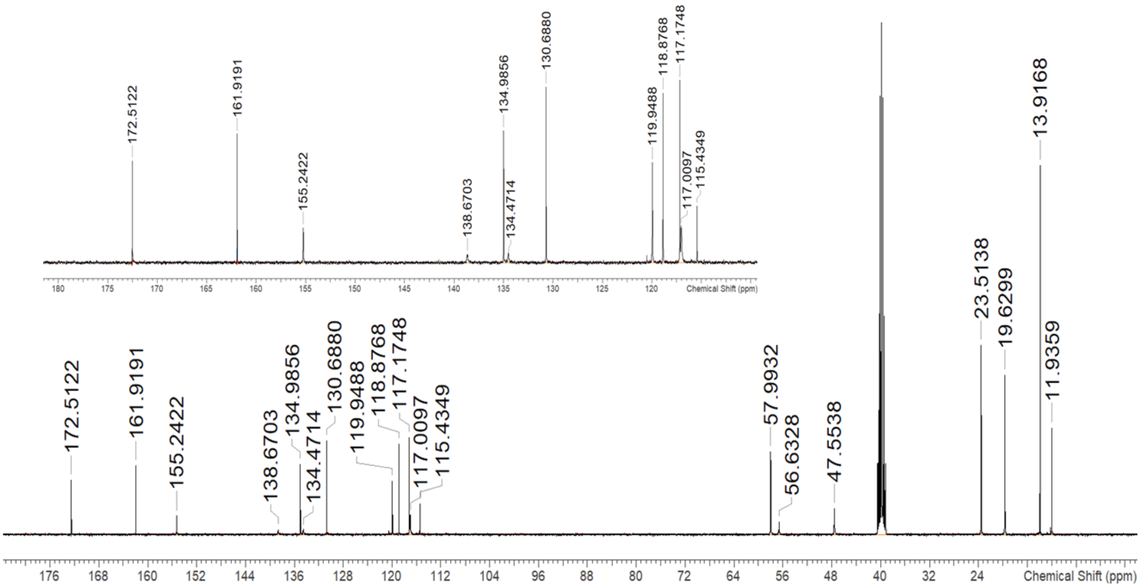

Figure S25 - ^{13}C NMR of co-formulation ***h*** in DMSO-d_6 conducted at 298 K.

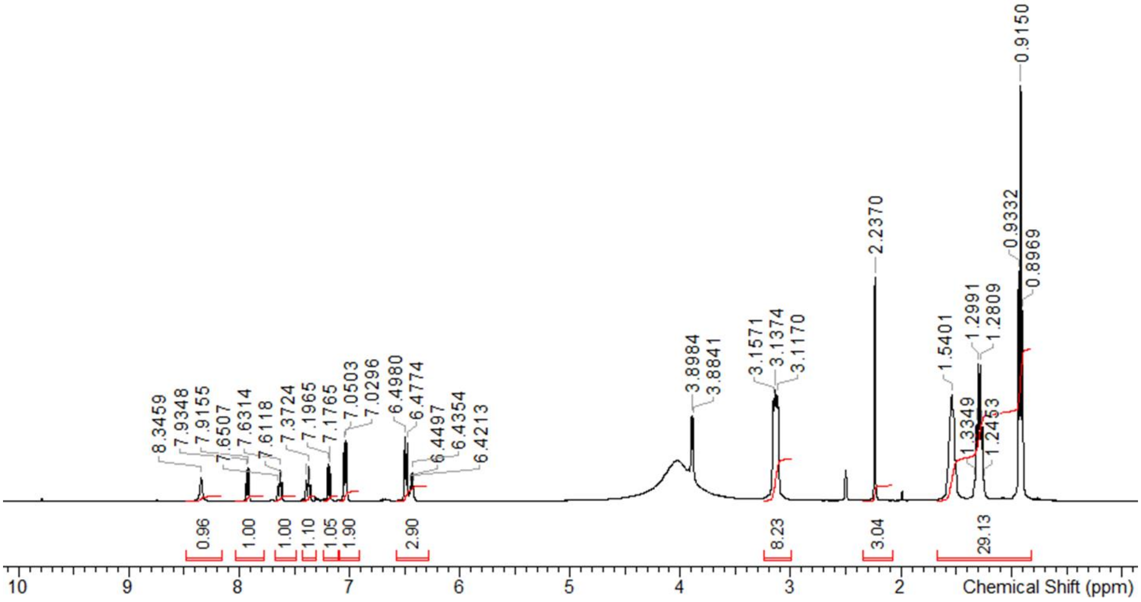

Figure S26 - ^{1}H NMR of co-formulation ***i*** in DMSO-d_6 conducted at 298 K.

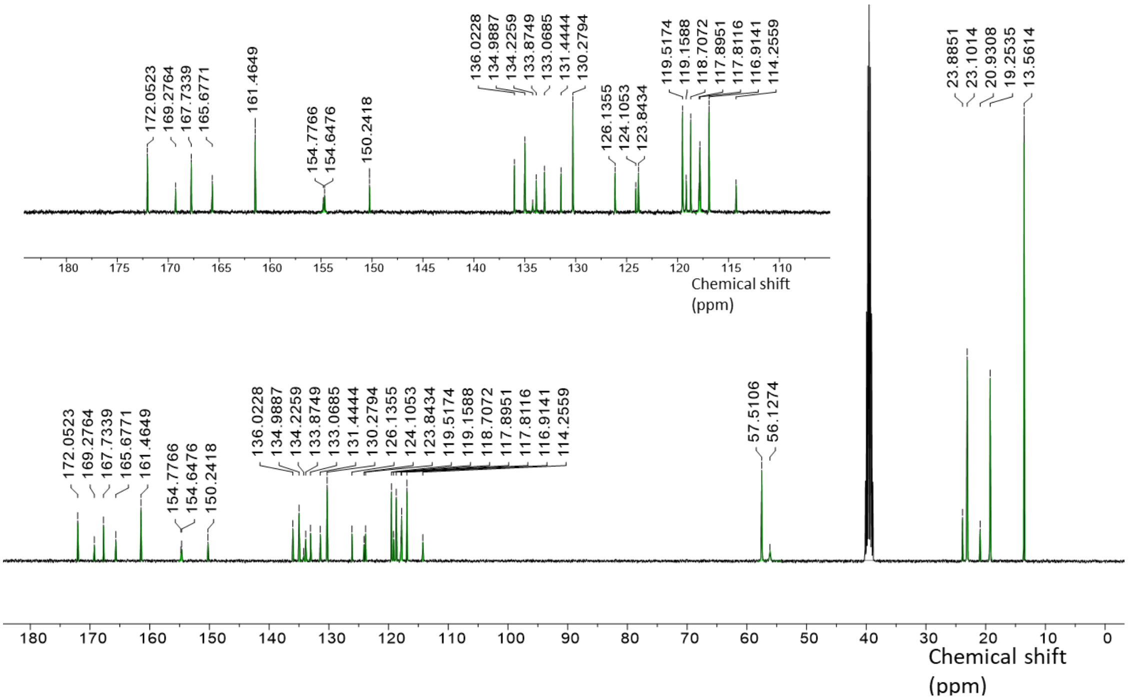

Figure S27 - ^{13}C NMR of co-formulation ***i*** in DMSO-d_6 conducted at 298K.

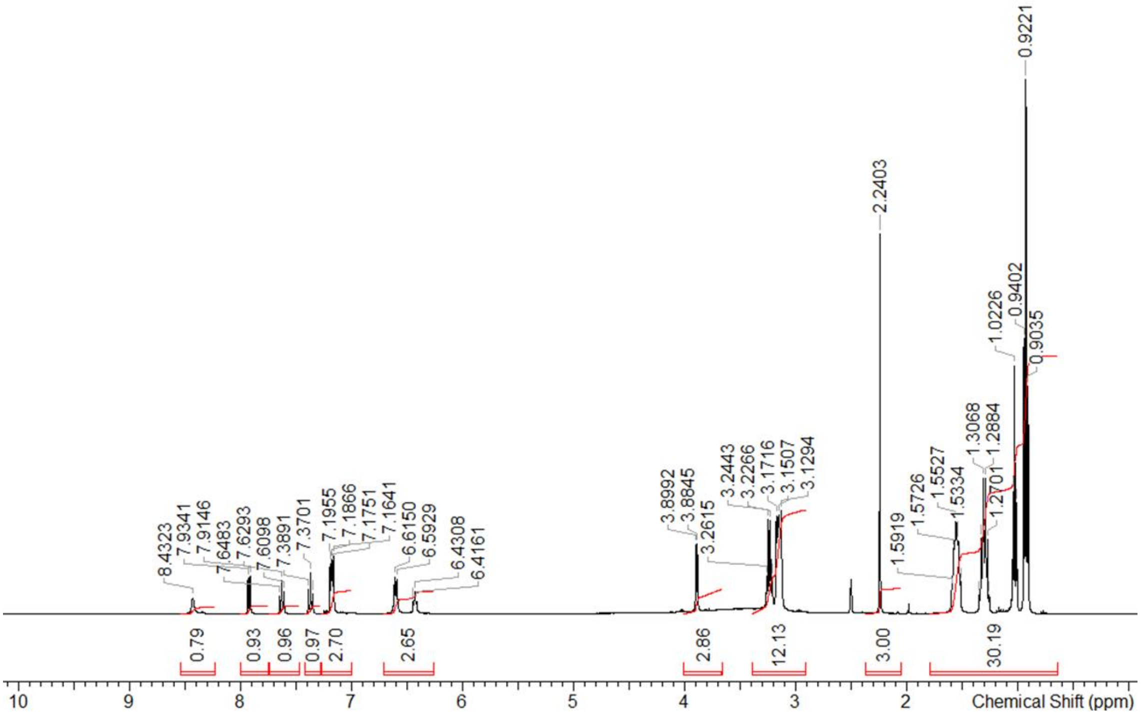

Figure S28 - ^{1}H NMR of co-formulation ***j*** in DMSO-d_6 conducted at 298 K.

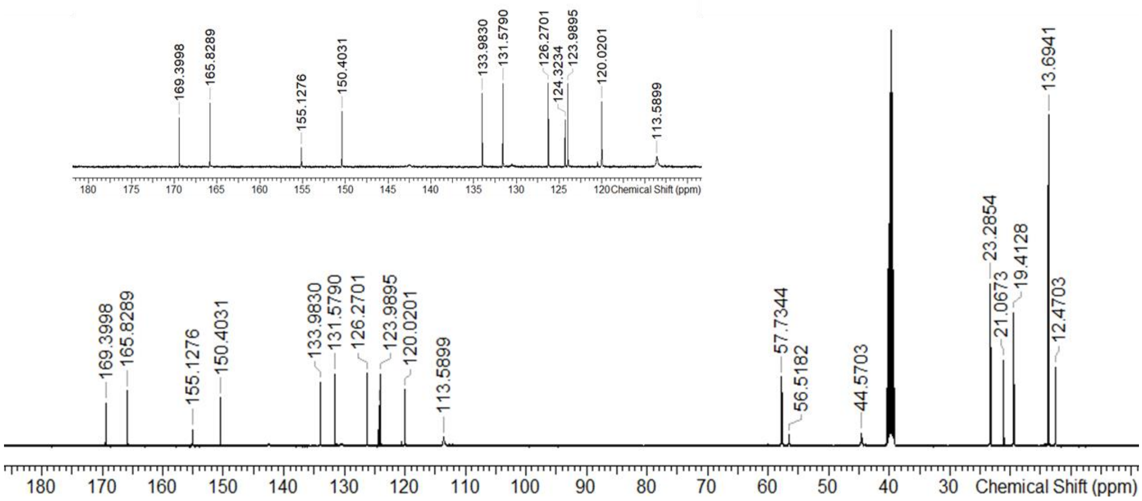

Figure S29 - ^{13}C NMR of co-formulation ***j*** in DMSO-d_6 conducted at 298 K.

^{1}H NMR quantitative studies

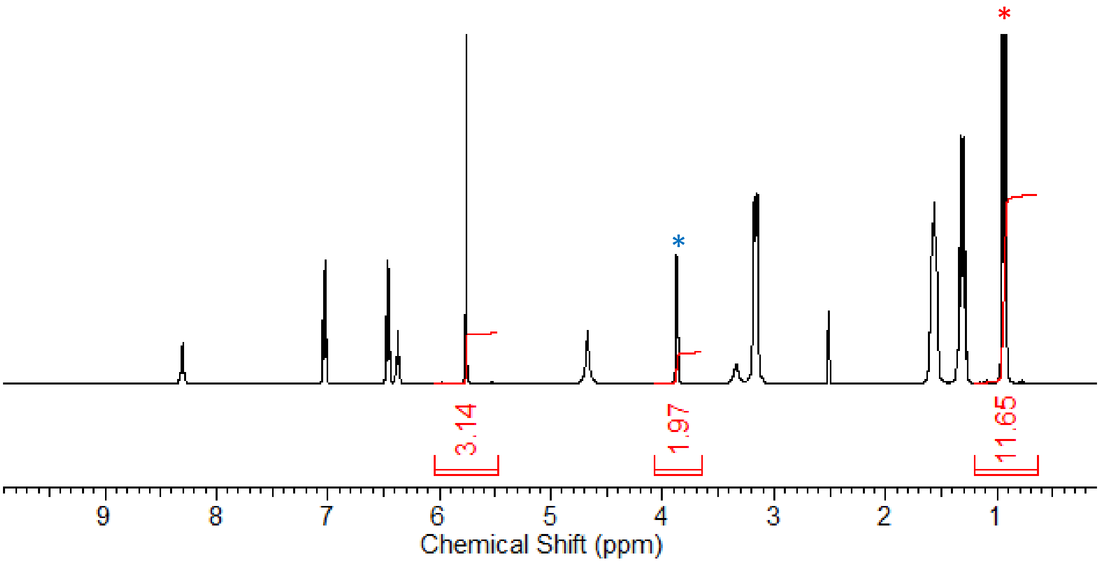

Figure 30 - ^{1}H NMR spectrum with a delay (d_1 = 60 s) of compound **2** (101.9 mM) in DMSO-d_6/ 1.0 % DCM. Comparative integration indicated 0 % of sample has become NMR silent (anionic component of SSA*, TBA*).

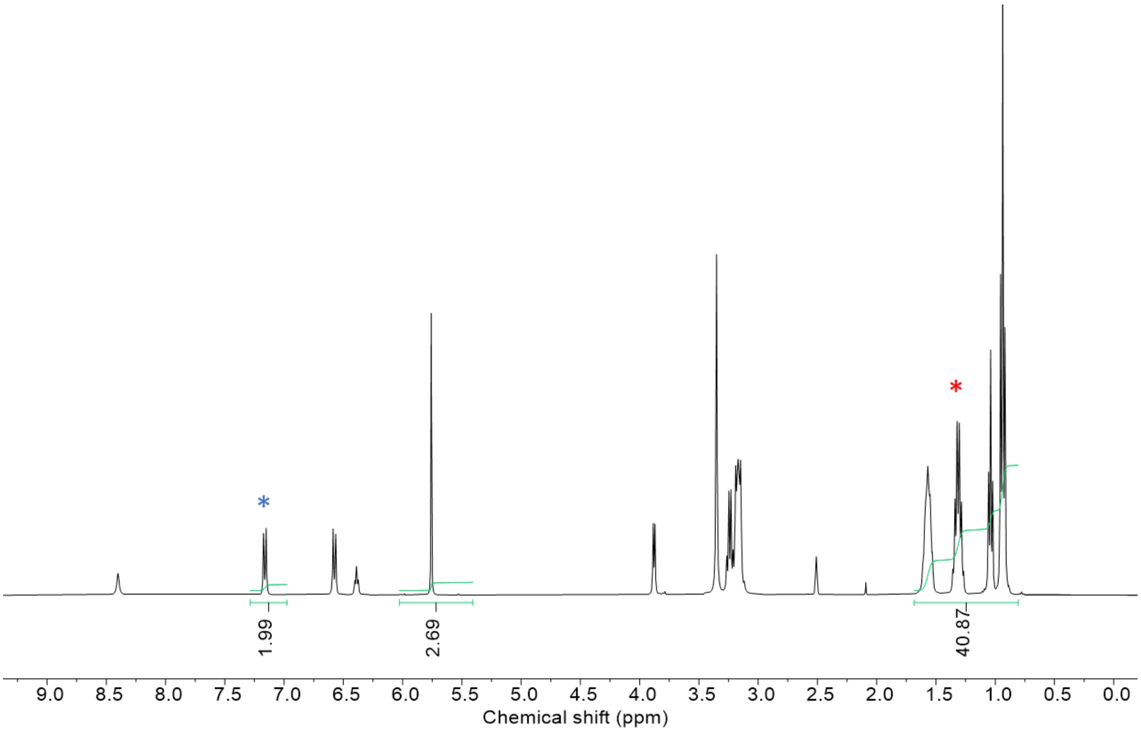

Figure S31 - ^{1}H NMR spectrum with a delay (d_1 = 60 s) of compound **3** (106.8 mM) in DMSO-d_6/ 1.0 % DCM. Comparative integration indicated 0 % of sample has become NMR silent (anionic component of SSA*, TBA*).

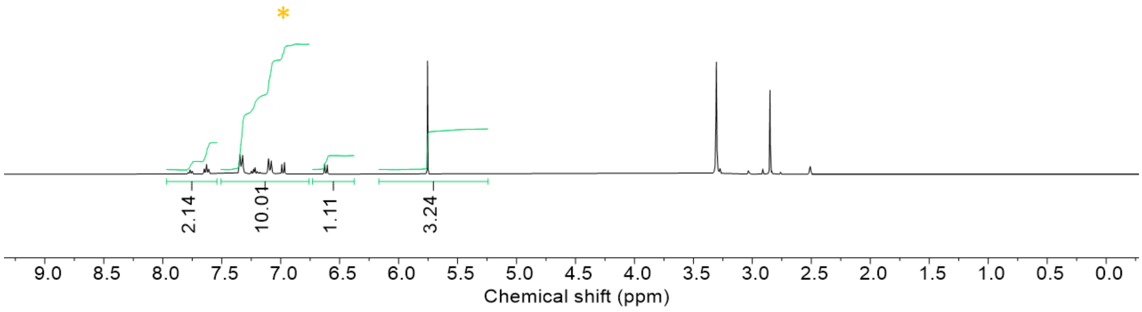

Figure S32 - ^{1}H NMR spectrum with a delay (d_1 = 60 s) of compound **5** (99.0 mM) in DMSO-d_6/ 1.0 % DCM. Comparative integration indicated 0 % of the sample has become NMR silent (malachite green*).

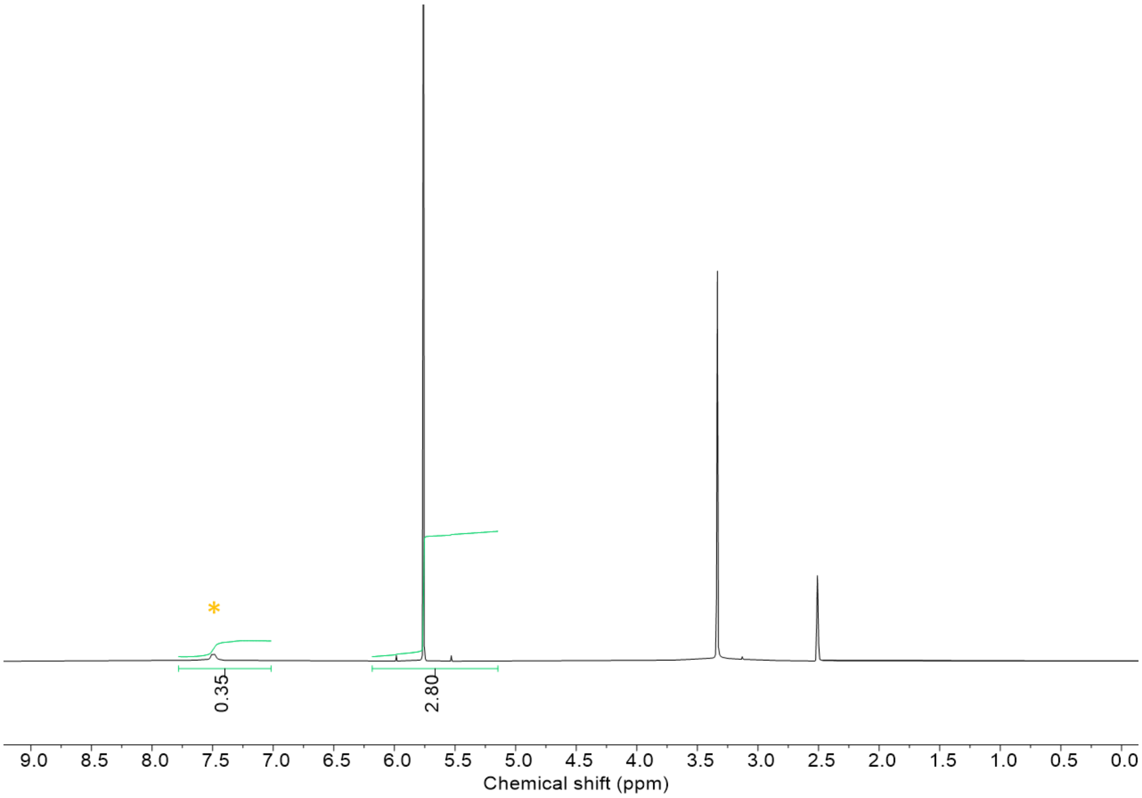

Figure S33 - ^{1}H NMR spectrum with a delay (d_1 = 60 s) of compound **6** (112.9 mM) in DMSO-d_6/ 1.0 % DCM. Comparative integration indicated 94 % of the sample has become NMR silent (methylene blue*).

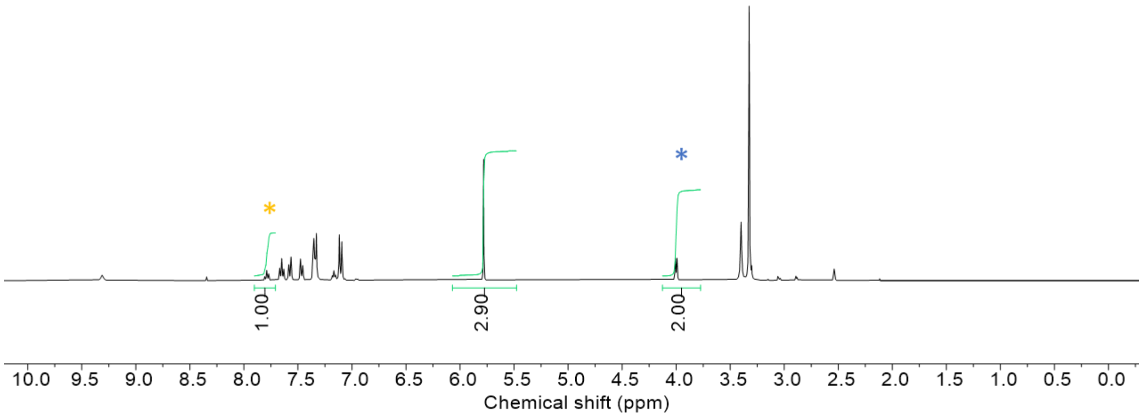

Figure S34 - ^{1}H NMR spectrum with a delay (d_1 = 60 s) of compound **10** (106.6 mM) in DMSO-d_6/ 1.0 % DCM. Comparative integration indicated 0 % of the sample has become NMR silent (green*, anionic component of SSA*).

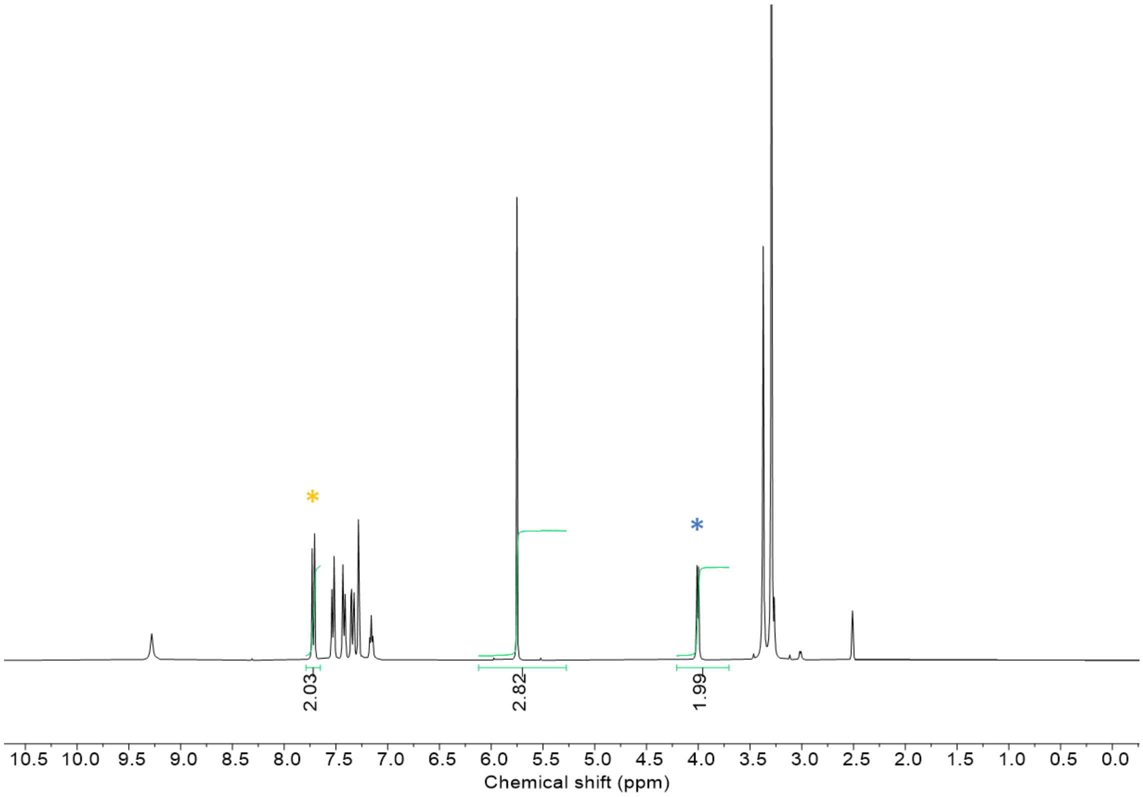

Figure S35 - ^{1}H NMR spectrum with a delay (d_1 = 60 s) of compound **11** (109.1 mM) in DMSO-d_6/ 1.0 % DCM. Comparative integration indicated 0 % of the sample has become NMR silent (methylene blue*, anionic component of SSA*).

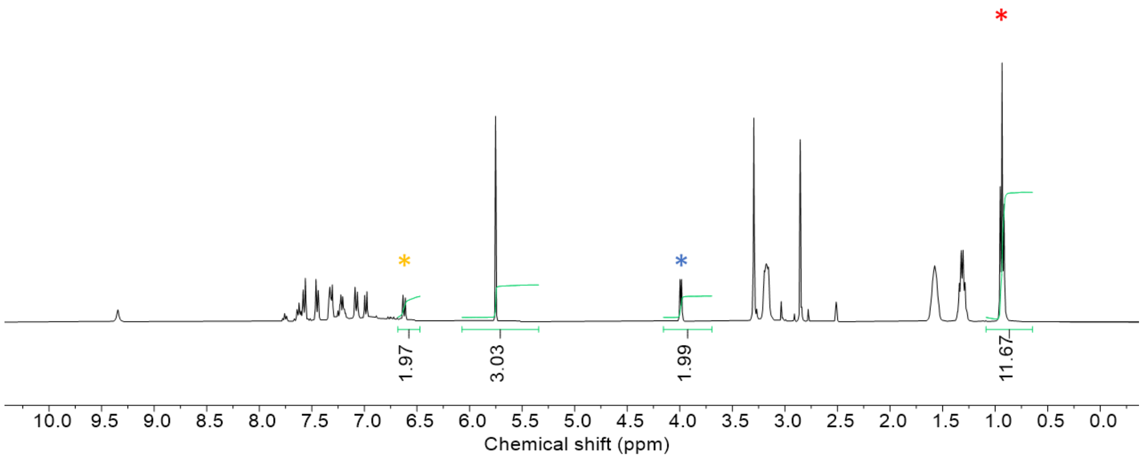

Figure S36 - ^{1}H NMR spectrum with a delay (d_1 = 60 s) of co-formulation ***a*** (105.6 mM) in DMSO-d_6/ 1.0 % DCM. Comparative integration indicated 0 % of the sample has become NMR silent (malachite green*, anionic component of SSA*, TBA*).

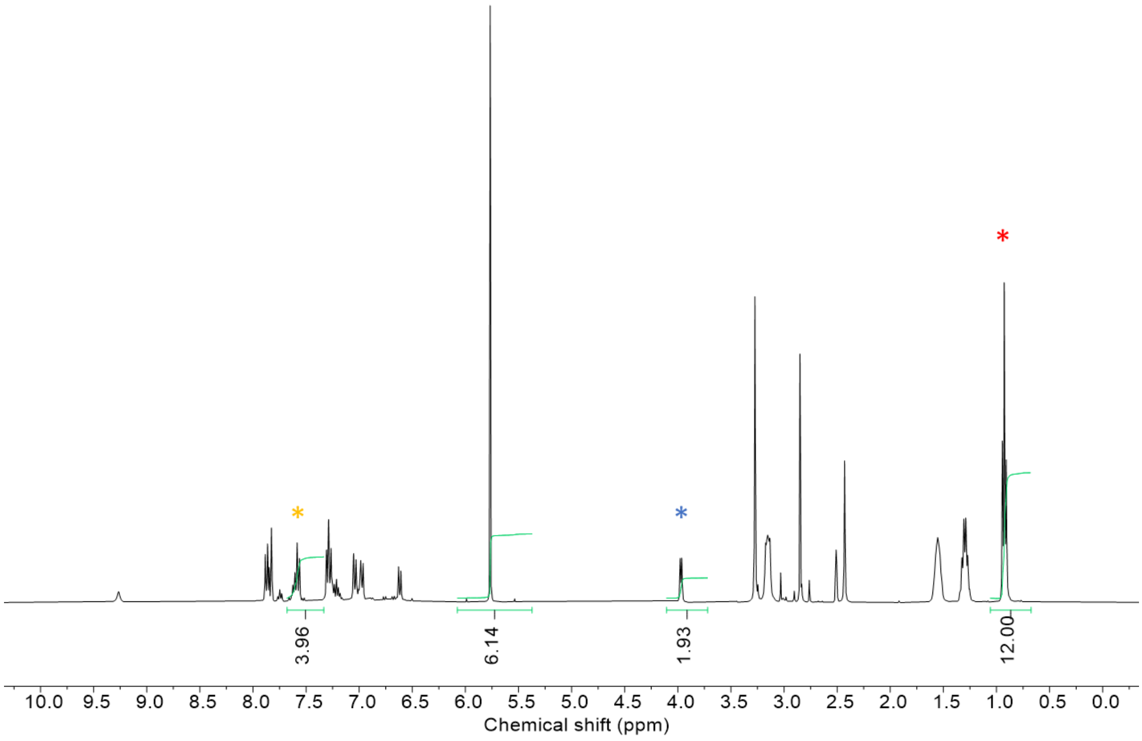

Figure S37 - ^{1}H NMR spectrum with a delay (d_1 = 60 s) of co-formulation ***b*** (109.8 mM) in DMSO-d_6/ 1.0 % DCM. Comparative integration indicated 0 % of the sample has become NMR silent (malachite green*, anionic component of SSA*, TBA*).

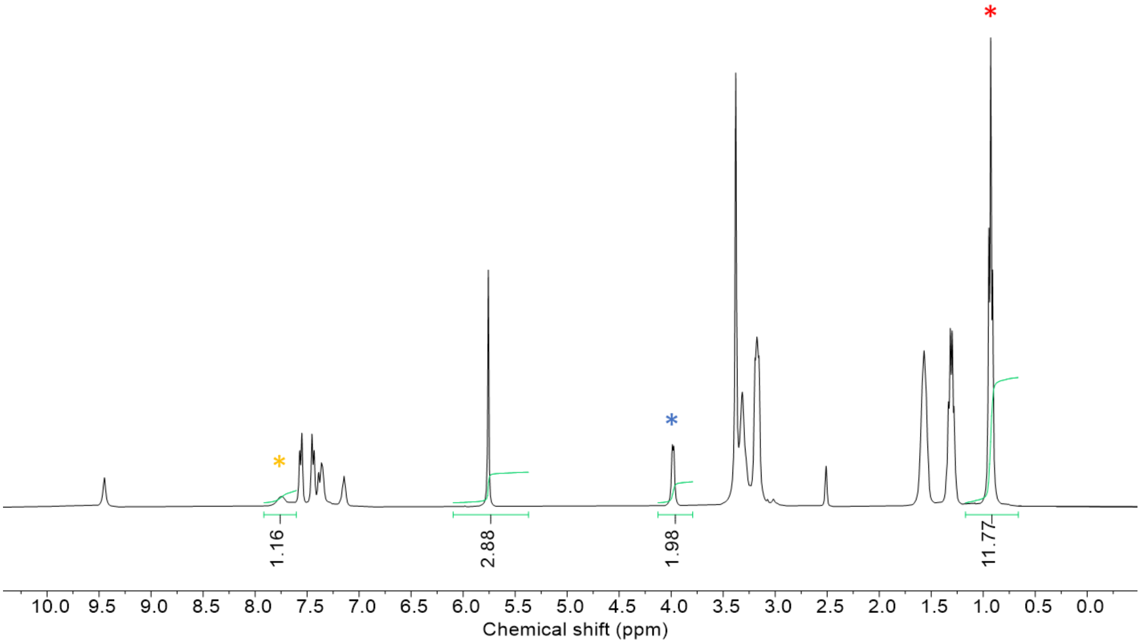

Figure S38 - ^{1}H NMR spectrum with a delay (d_1 = 60 s) of co-formulation ***c*** (111.1 mM) in DMSO-d_6/ 1.0 % DCM. Comparative integration indicated 0 % of the sample has become NMR silent (methylene blue*, anionic component of SSA*, TBA*).

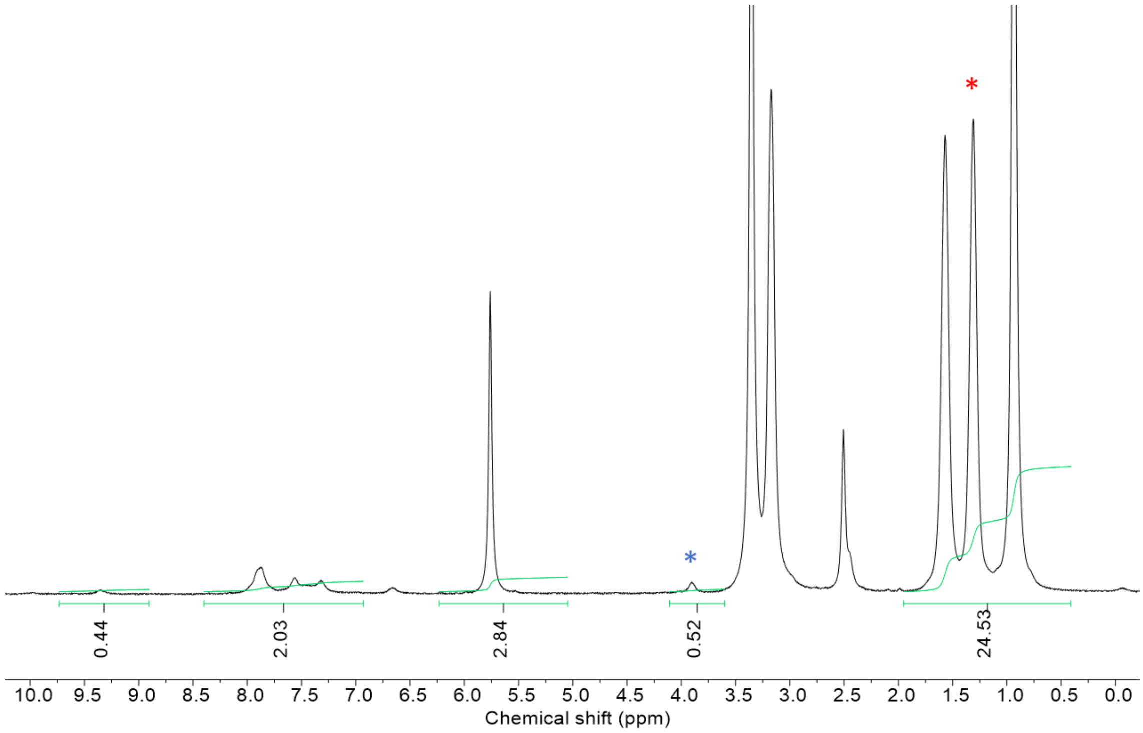

-

Figure S39 - ^{1}H NMR spectrum with a delay (d_1 = 60 s) of co-formulation ***d*** (111.2 mM) in DMSO-d_6/ 1.0 % DCM. Comparative integration indicated 74 % of the aromatic anionic component of SSA, 12 % of TBA and an undetermined % of the methylene blue has become NMR silent (anionic component of SSA*, TBA*).

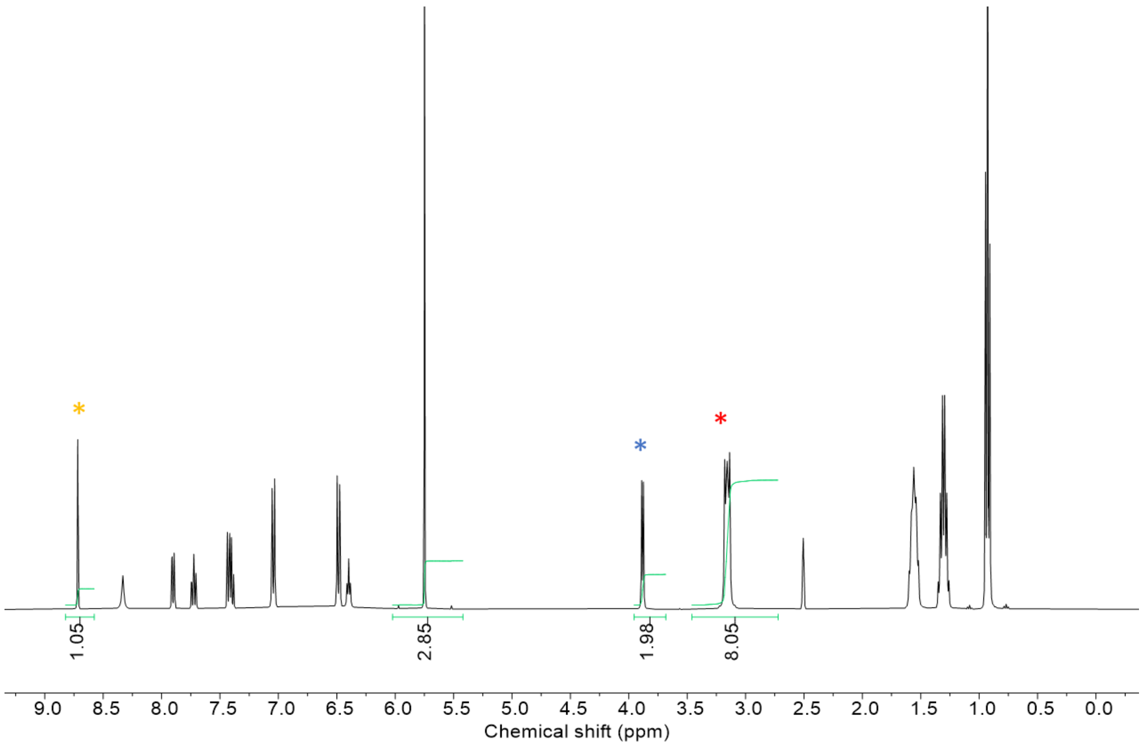

Figure S40 - ^{1}H NMR spectrum with a delay (d_1 = 60 s) of co-formulation ***e*** (114.9 mM) in DMSO-d_6/ 1.0 % DCM. Comparative integration indicated 0 % of the sample has become NMR silent (coumarin*, anionic component of SSA*, TBA*).

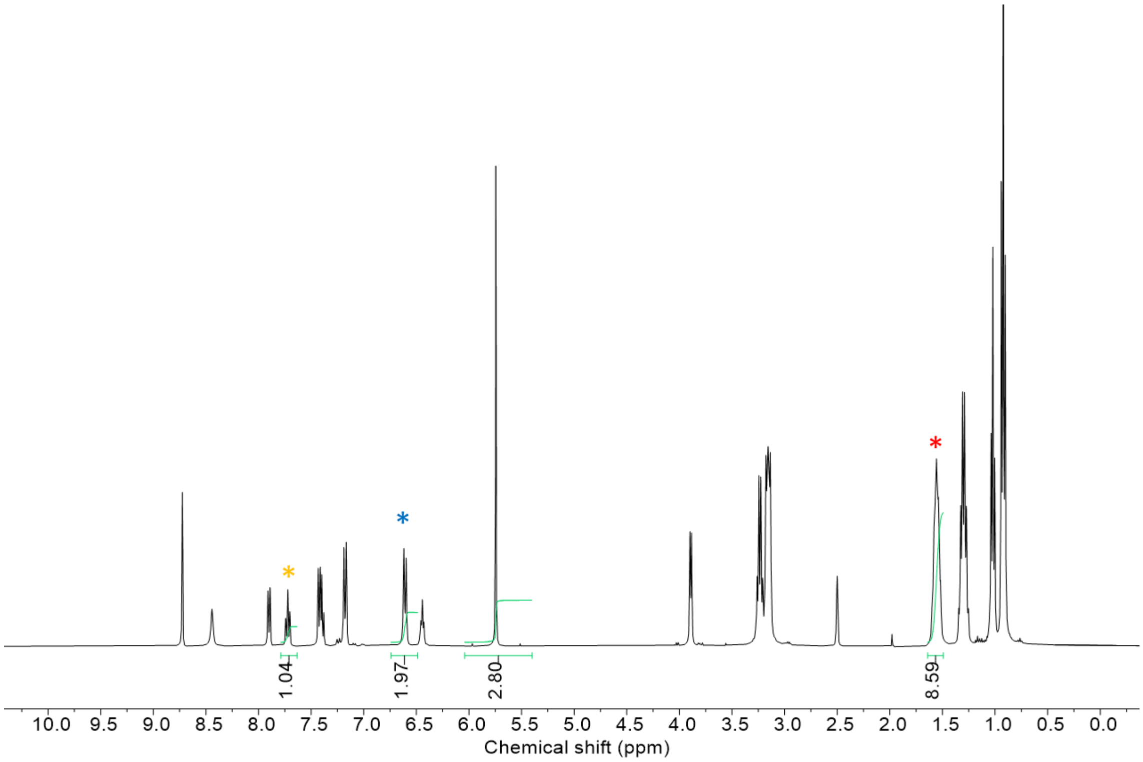

Figure S41 - ^{1}H NMR spectrum with a delay (d_1 = 60 s) of co-formulation ***f*** (115.9 mM) in DMSO-d_6/ 1.0 % DCM. Comparative integration indicated 0 % of sample has become NMR silent (Coumarin*, anionic component of SSA*, TBA*).

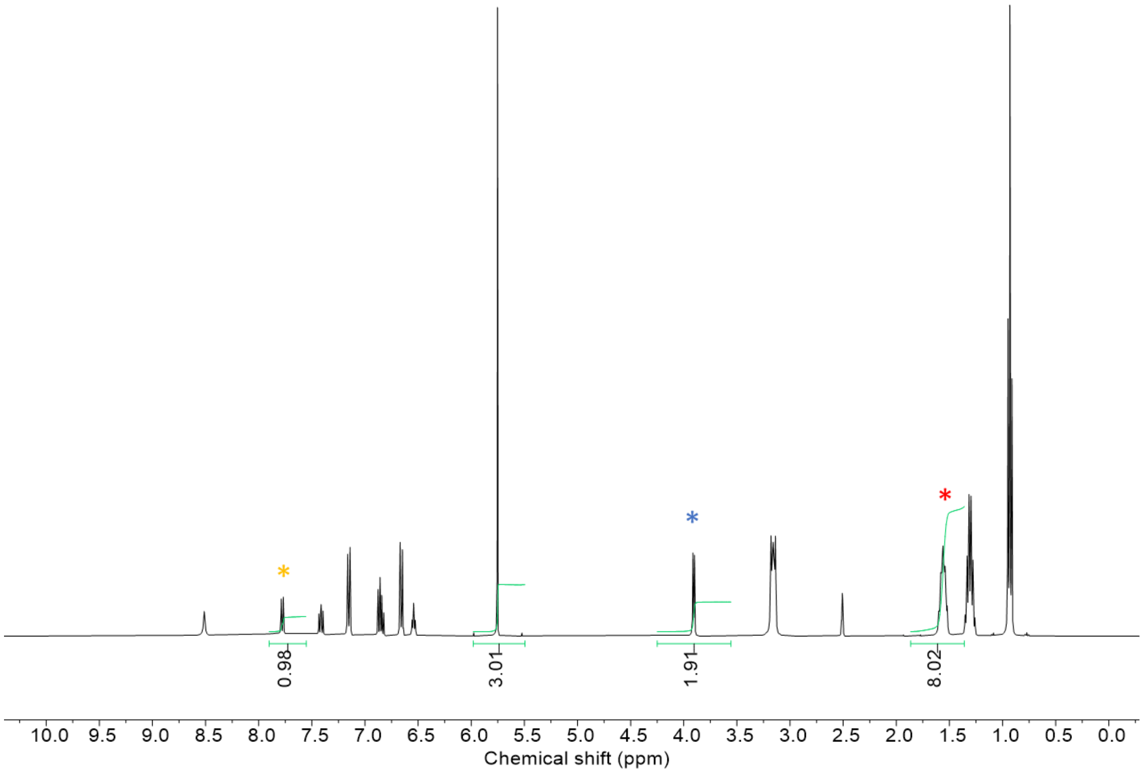

Figure S42 - ^{1}H NMR spectrum with a delay (d_1 = 60 s) of co-formulation ***g*** (110.9 mM) in DMSO-d_6/ 1.0 % DCM. Comparative integration indicated 0 % of the sample has become NMR silent (salicylic acid*, anionic component of SSA*, TBA*).

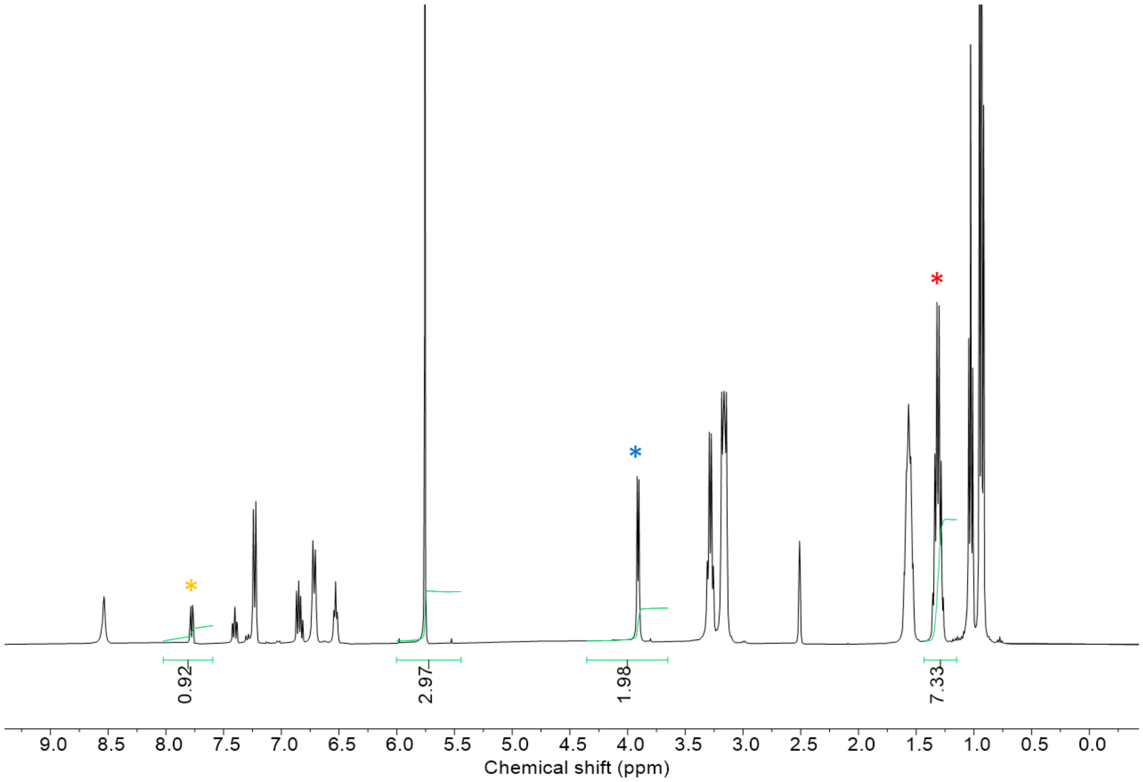

Figure S43 - ^{1}H NMR spectrum with a delay (d_1 = 60 s) of co-formulation ***h*** (107.4 mM) in DMSO-d_6/ 1.0 % DCM. Comparative integration indicated 0 % of the sample has become NMR silent (salicylic acid*, anionic component of SSA*, TBA*).

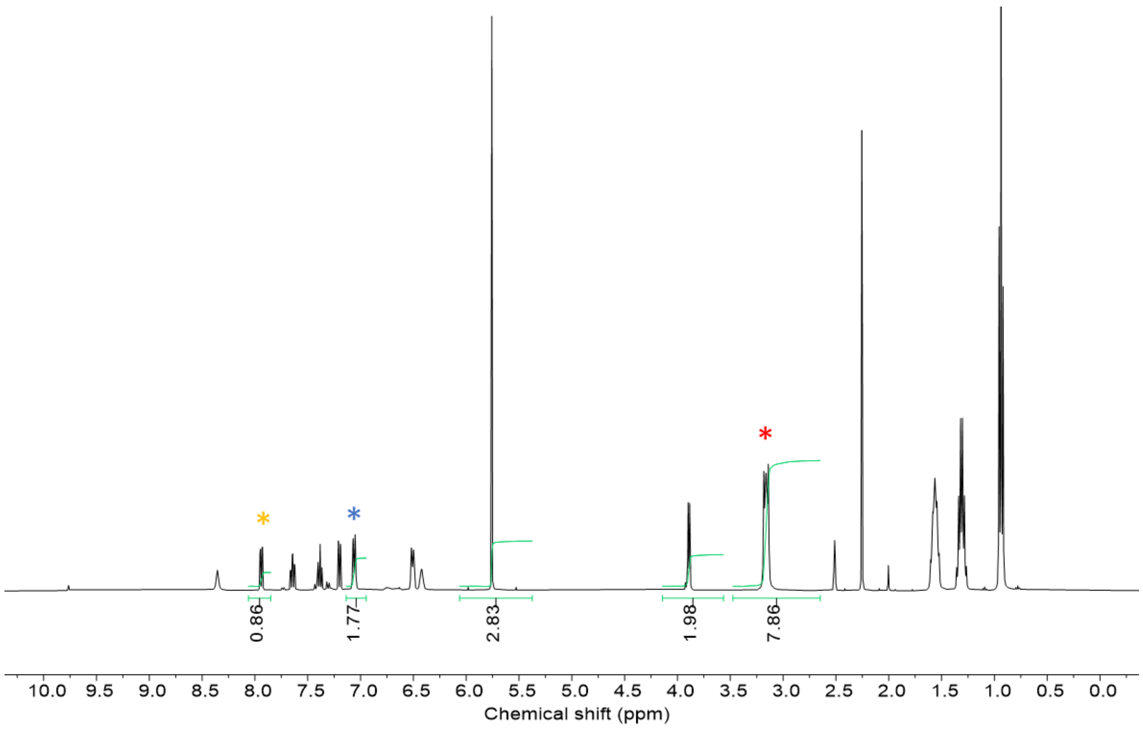

Figure S44 - ^{1}H NMR spectrum with a delay (d_1 = 60 s) of co-formulation ***i*** (112.8 mM) in DMSO-d_6/ 1.0 % DCM. Comparative integration indicated 12 % of the aromatic anionic component of SSA, 2 % of TBA and 14 % of the acetylsalicylic acid has become NMR silent (acetylsalicylic acid*, anionic component of SSA*, TBA*).

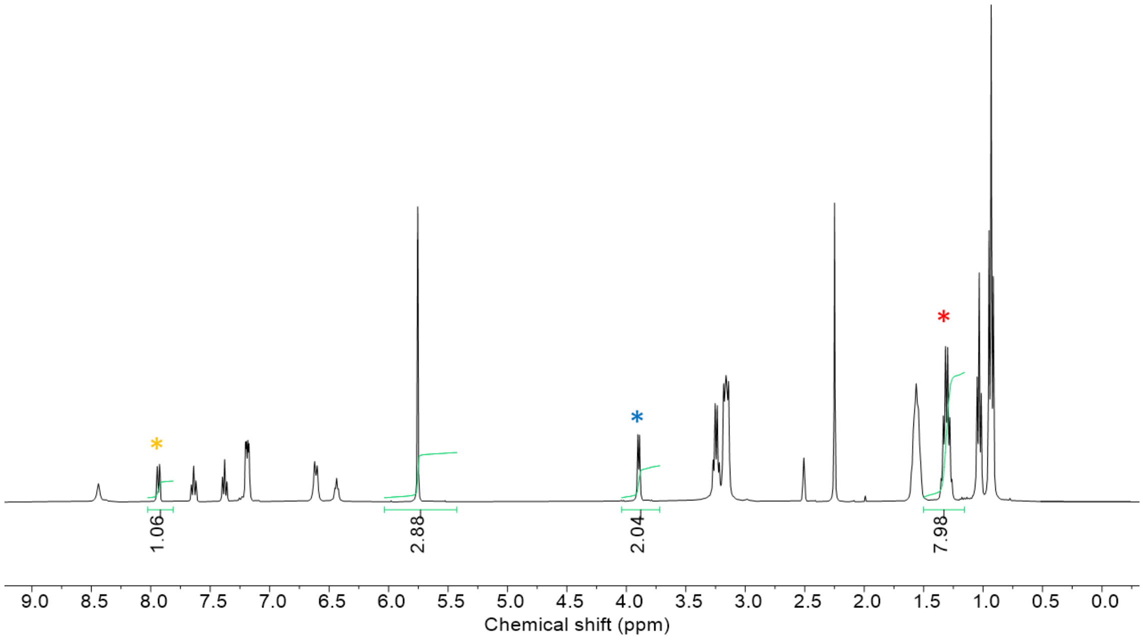

Figure S45 - ^{1}H NMR spectrum with a delay (d_1 = 60 s) of co-formulation ***j*** (111.1 mM) in DMSO-d_6/ 1.0 % DCM. Comparative integration indicated 0 % of the sample has become NMR silent (acetylsalicylic acid*, anionic component of SSA*, TBA*).

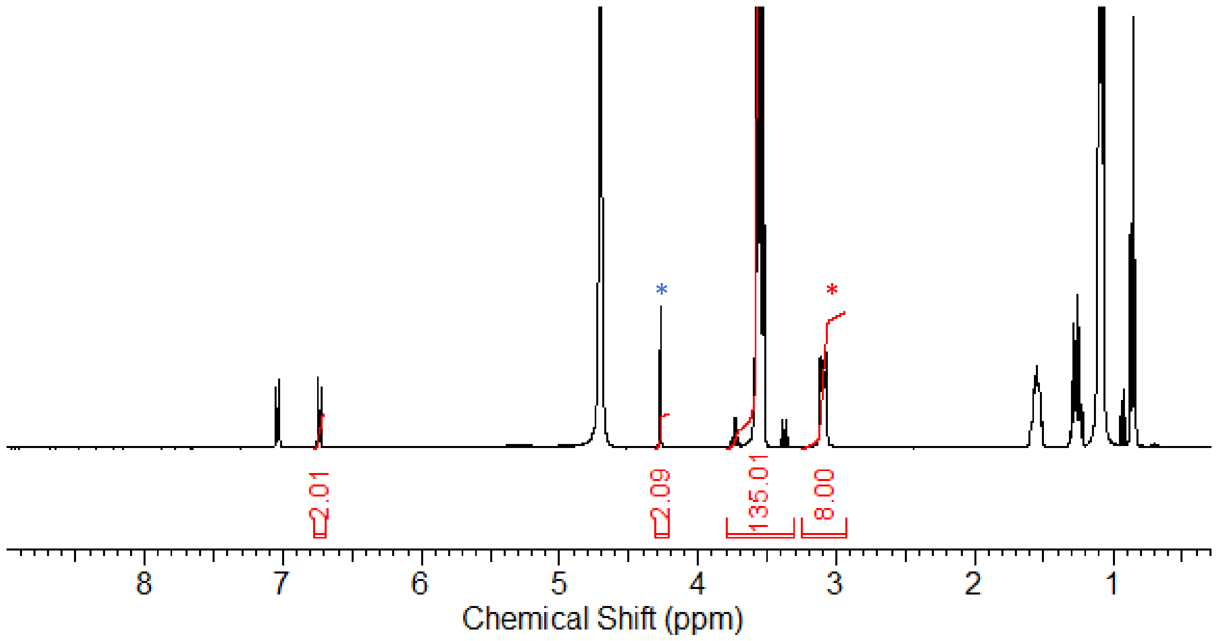

Figure S46 - ^{1}H NMR spectrum with a delay (d_1= 60 s) of compound **2** (12.74 mM) in D_2O/ 5.0 % EtOH. Comparative integration indicated 0 % of the sample has become NMR silent (anionic component of SSA*, TBA*).

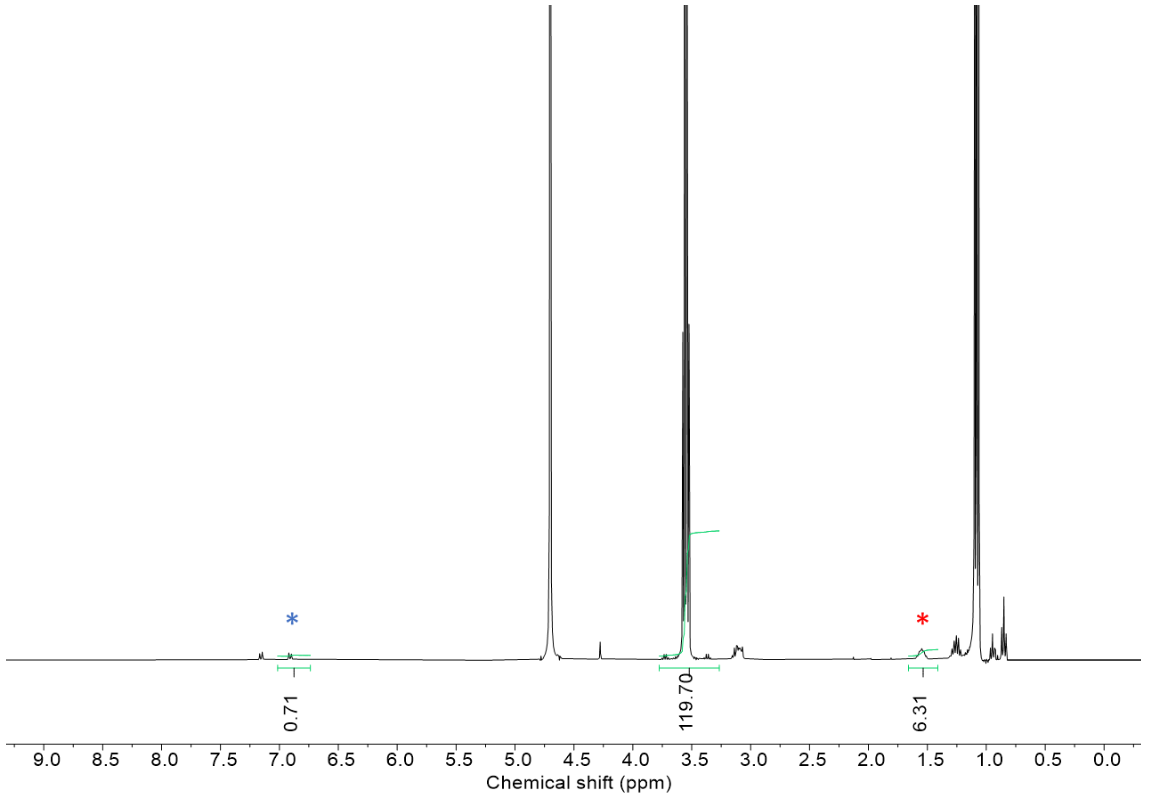

Figure S47 - ^{1}H NMR spectrum with a delay (d_1= 60 s) of compound **3** (11.10 mM) in D_2O/ 5.0 % EtOH. Comparative integration indicated 65 % of the anionic component of SSA and 21 % of TBA has become NMR silent (anionic component of SSA*, TBA*).

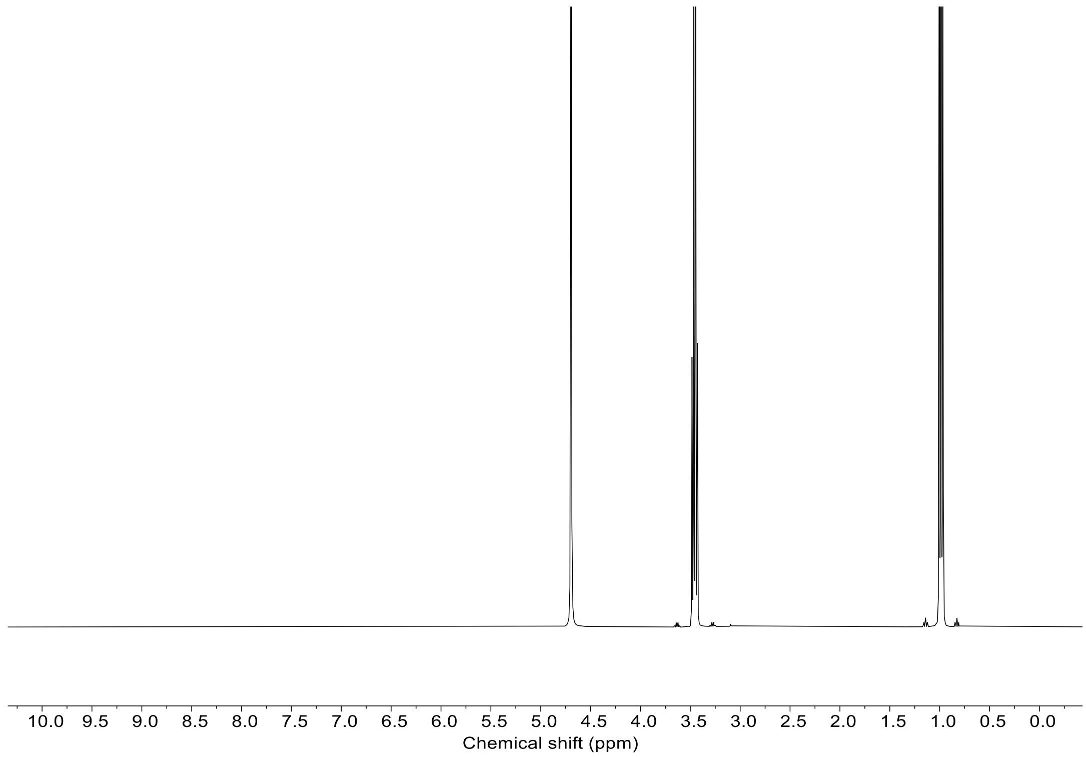

Figure S48 - ^{1}H NMR spectrum with a delay (d_1 = 60 s) of compound **10** (11.48 mM) in D_2O/ 5.0 % EtOH. Comparative integration indicated 100 % of the anionic component of SSA and 100 % of the malachite green has become NMR silent.

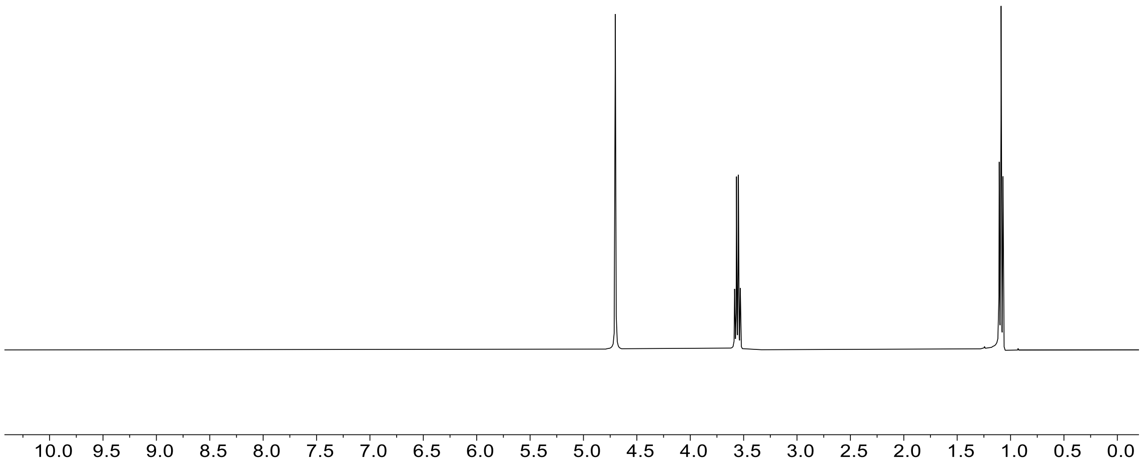

Figure S49 - ^{1}H NMR spectrum with a delay (d_1 = 60 s) of compound **11** (11.02 mM) in D_2O/ 5.0 % EtOH. Comparative integration indicated 100 % of the anionic component of SSA and 100 % of the methylene blue has become NMR silent.

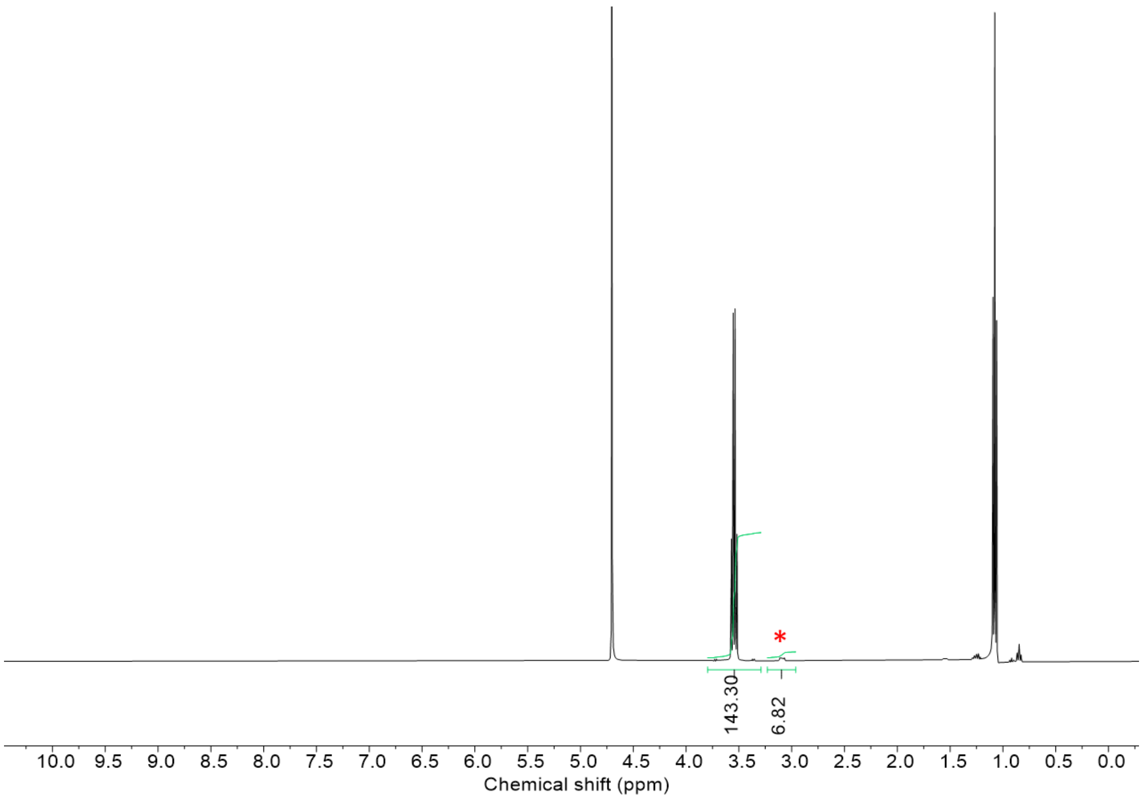

Figure S50 - ^{1}H NMR spectrum with a delay (d_1 = 60 s) of co-formulation ***a*** (12.00 mM) in D_2O/ 5.0 % EtOH. Comparative integration indicated 100 % of the anionic component of SSA, 100 % of the malachite green and 15 % of TBA has become NMR silent (TBA*).

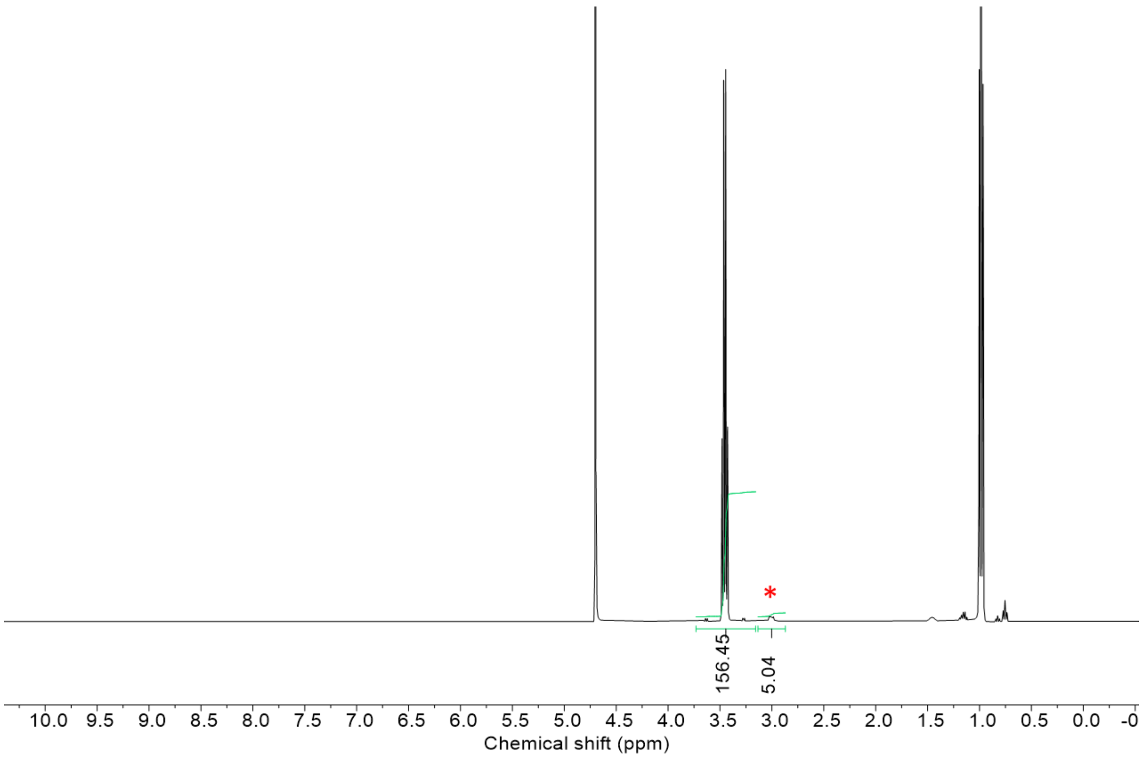

Figure S51 - ^{1}H NMR spectrum with a delay (d_1 = 60 s) of co-formulation ***b*** (11.00 mM) in D_2O/ 5.0 % EtOH. Comparative integration indicated 100 % of the anionic component of SSA, 100 % of the malachite green and 37 % of TBA has become NMR silent (TBA*).

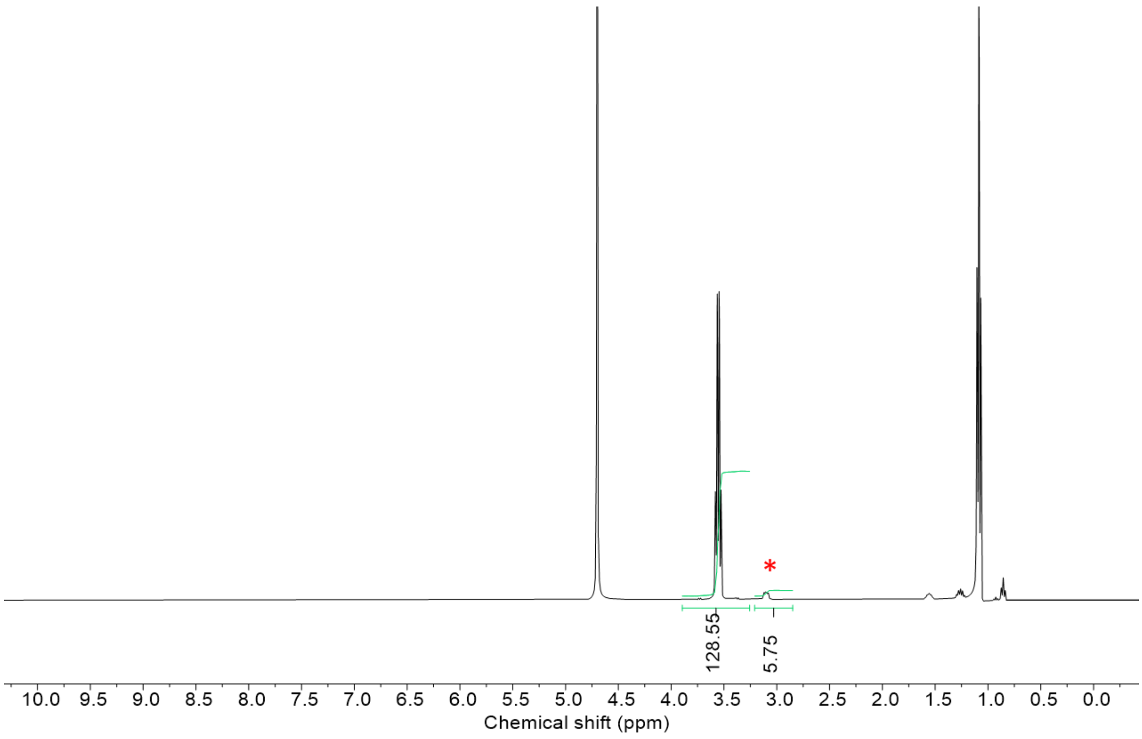

Figure S52 - ^{1}H NMR spectrum with a delay (d_1 = 60 s) of co-formulation ***c*** (13.40 mM) in D_2O/ 5.0 % EtOH. Comparative integration indicated 100 % of the anionic component of SSA, 100 % of the methylene blue and 28 % of TBA has become NMR silent (TBA*).

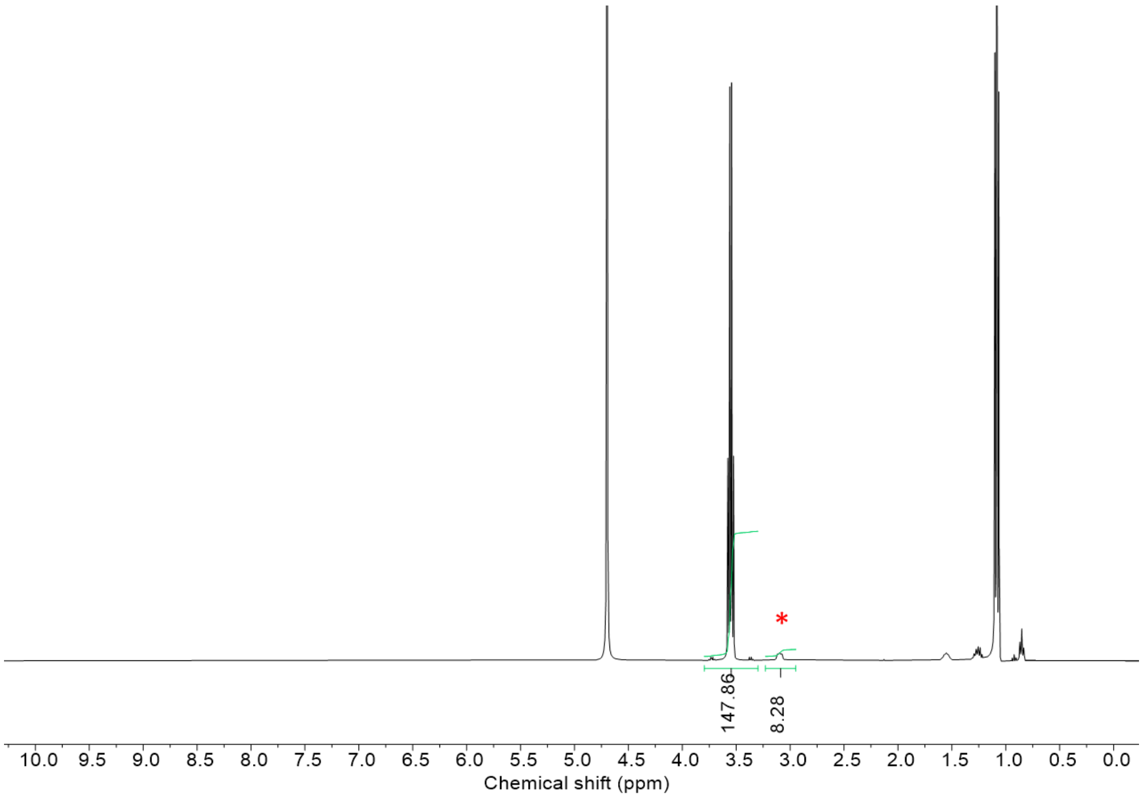

Figure S53 - ^{1}H NMR spectrum with a delay (d_1 = 60 s) of co-formulation ***d*** (11.64 mM) in D_2O/ 5.0 % EtOH. Comparative integration indicated 100 % of the anionic component of SSA, 100 % of the methylene blue and 0 % of TBA has become NMR silent (TBA*).

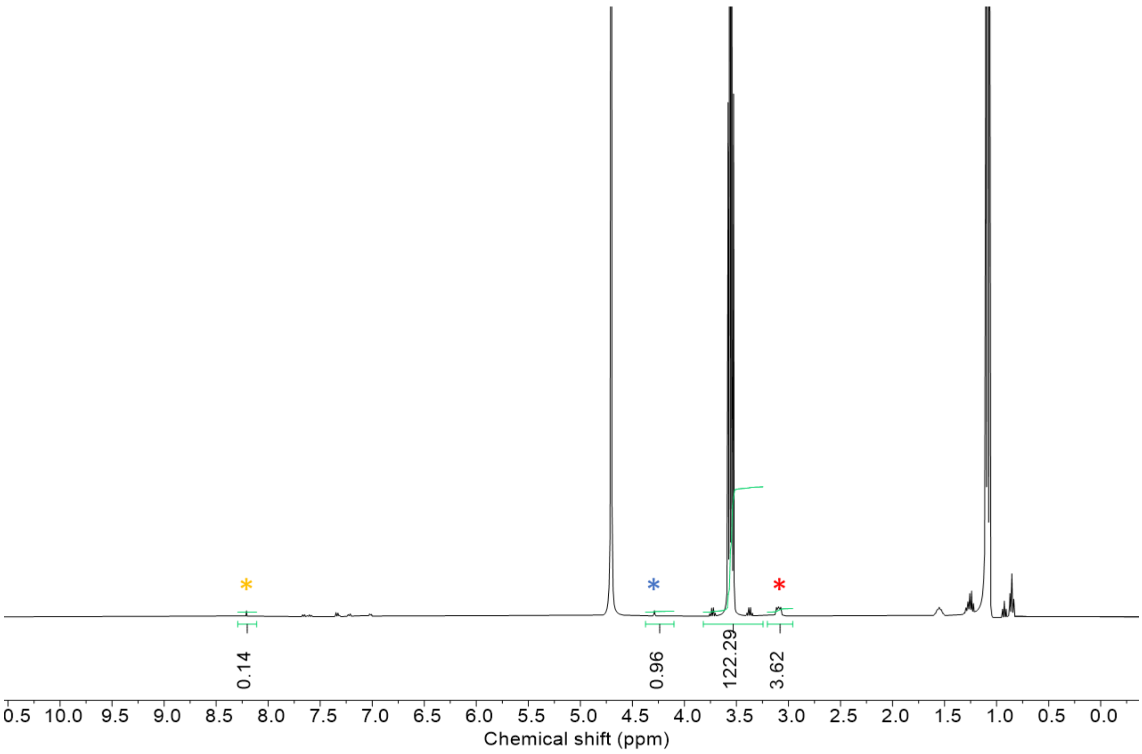

Figure S54 - ^{1}H NMR spectrum with a delay (d_1 = 60 s) of co-formulation ***e*** (14.06 mM) in D_2O/ 5.0 % EtOH. Comparative integration indicated 52 % of the anionic component of SSA, 55 % of TBA and 86 % of the coumarin has become NMR silent (coumarin*, anionic component of SSA*, TBA*).

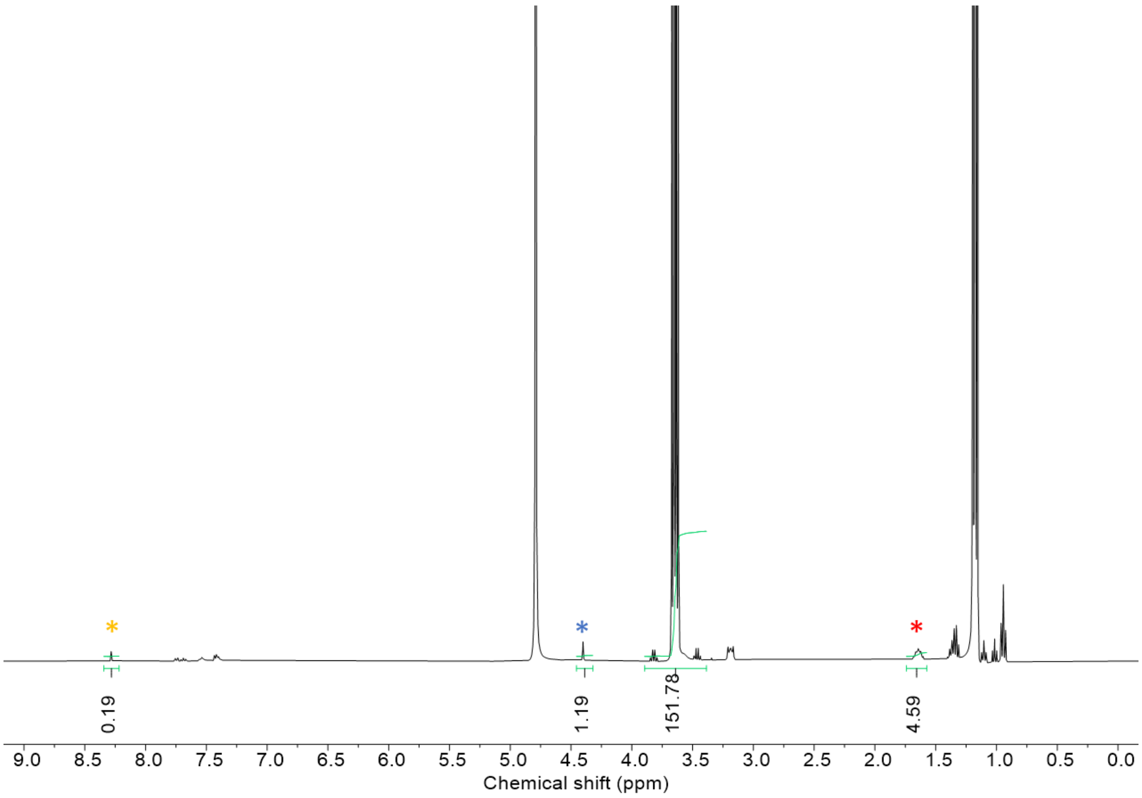

Figure S55 - ^{1}H NMR spectrum with a delay (d_1 = 60 s) of co-formulation ***f*** (11.40 mM) in D_2O/ 5.0 % EtOH. Comparative integration indicated 41 % of the anionic component of SSA, 43 % of TBA and 81 % of the coumarin has become NMR silent (coumarin*, anionic component of SSA*, TBA*).

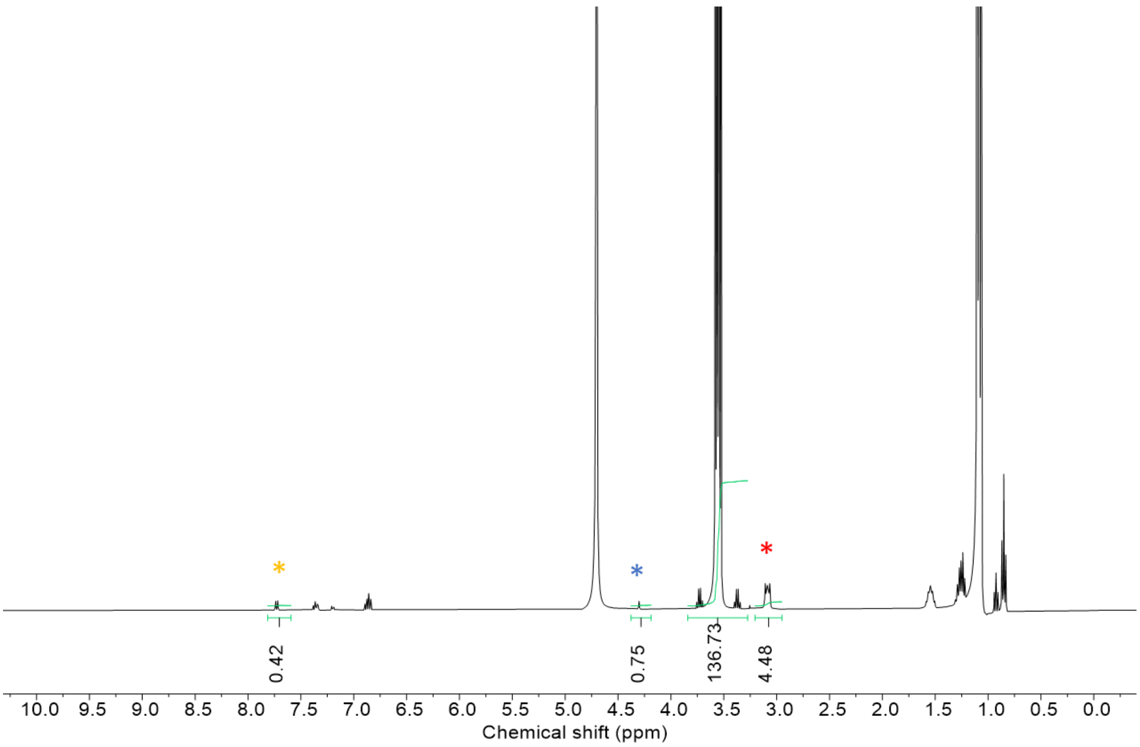

Figure S56 - ^{1}H NMR spectrum with a delay (d_1 = 60 s) of co-formulation ***g*** (13.84 mM) in D_2O/ 5.0 % EtOH. Comparative integration indicated 63 % of the anionic component of SSA, 44 % of TBA and 58 % of the coumarin has become NMR silent (coumarin*, anionic component of SSA*, TBA*).

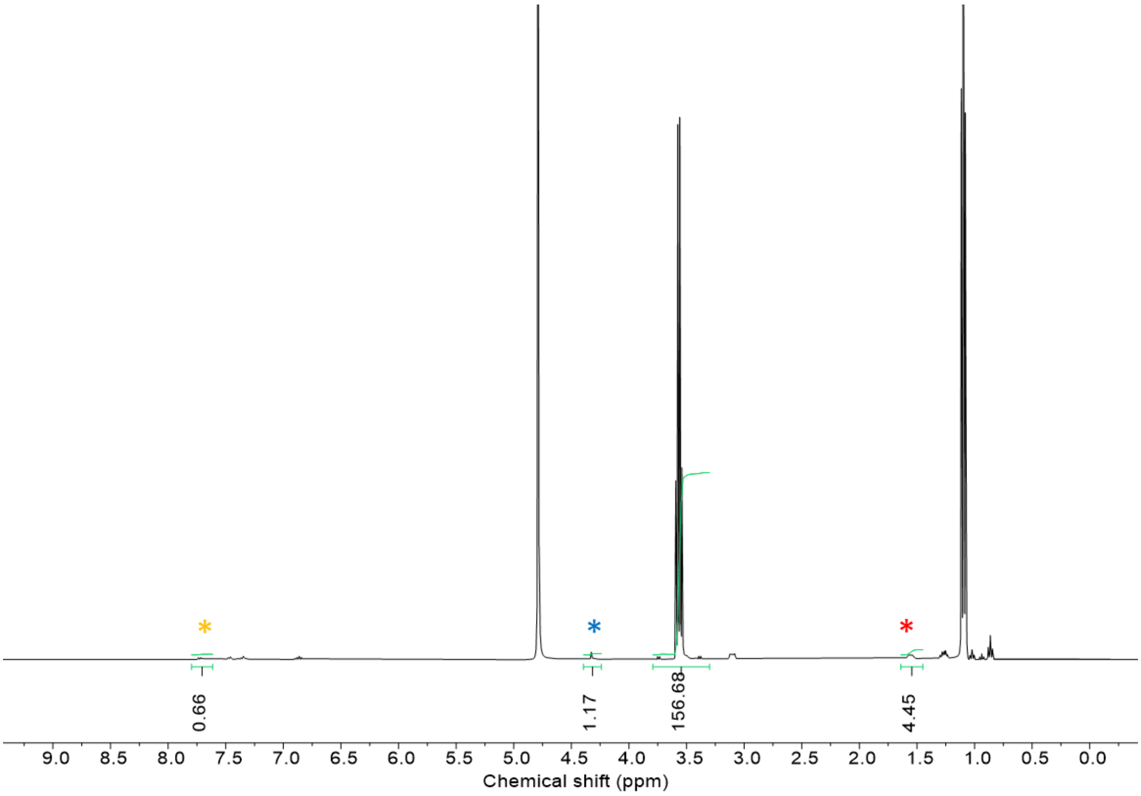

Figure S57 - ^{1}H NMR spectrum with a delay (d_1= 60 s) of co-formulation ***h*** (11.06 mM) in D_2O/ 5.0 % EtOH. Comparative integration indicated 42 % of the anionic component of SSA, 44 % of TBA and 34 % of the salicylic acid has become NMR silent (salicylic acid*, anionic component of SSA*, TBA*).

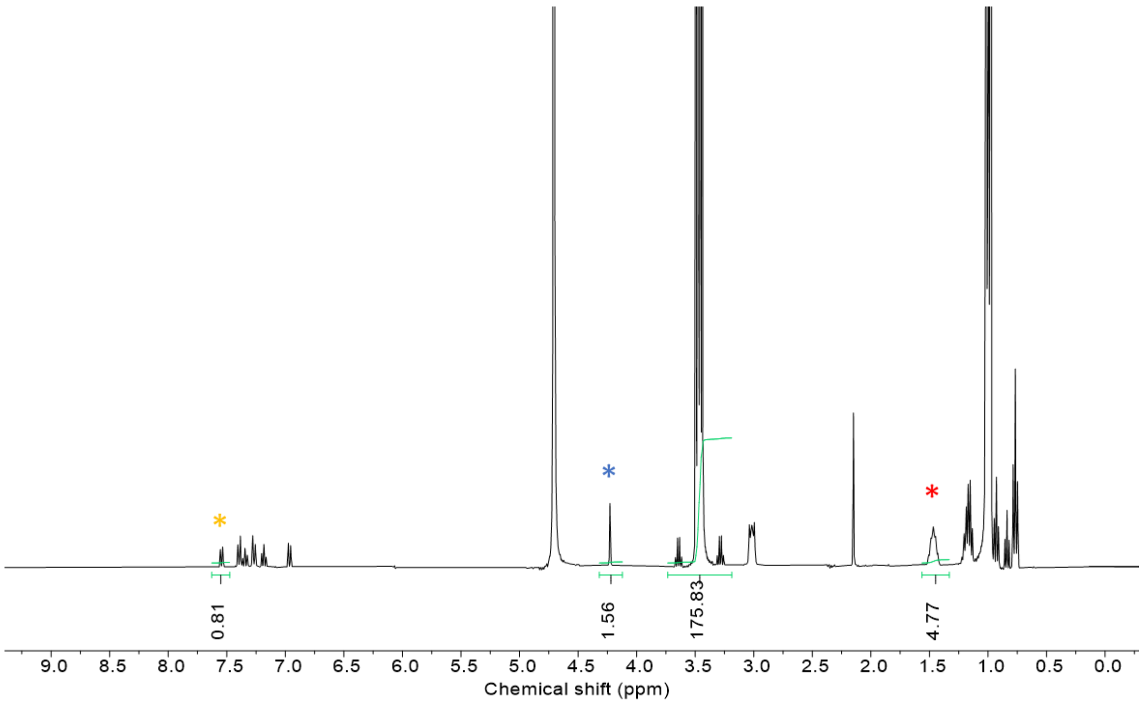

Figure S58 - ^{1}H NMR spectrum with a delay (d_1 = 60 s) of co-formulation ***j*** (9.78 mM) in D_2O/ 5.0 % EtOH. Comparative integration indicated 22 % of the anionic component of SSA, 40 % of TBA and 19 % of the acetylsalicylic acid has become NMR silent (acetylsalicylic acid*, anionic component of SSA*, TBA*).

^{1}H NMR DOSY studies

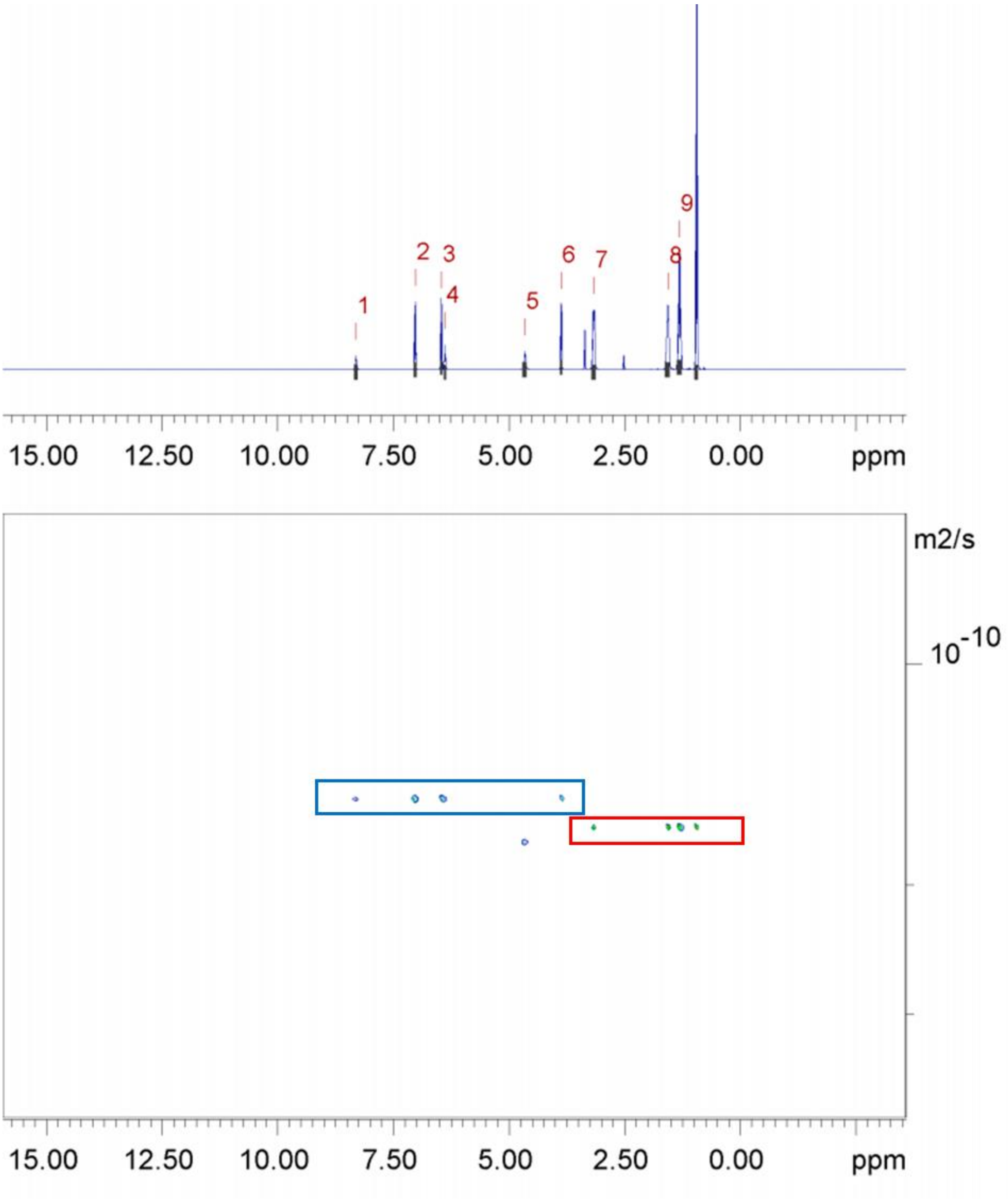

Peak name	F2 [ppm]	Io	error	D [m2/s]	error
1	8.304	1.02e+09	7.533e+04	1.53e-10	2.471e-14
2	7.023	3.19e+09	6.694e+04	1.53e-10	7.045e-15
3	6.459	3.23e+09	6.528e+04	1.52e-10	6.774e-15
4	6.379	1.35e+09	6.518e+04	1.53e-10	1.625e-14
5	4.646	1.52e+09	6.966e+04	1.75e-10	1.741e-14
6	3.867	2.89e+09	5.940e+04	1.52e-10	6.884e-15
7	3.163	7.55e+09	7.699e+04	1.66e-10	3.702e-15
8	1.562	8.74e+09	8.616e+04	1.66e-10	3.578e-15
9	1.310	1.12e+10	8.744e+04	1.66e-10	2.831e-15
10	0.937	1.88e+10	7.856e+04	1.67e-10	1.521e-15

Figure S59 - ^{1}H DOSY NMR spectrum of compound **2** (111.12 mM) in DMSO-d_6 at 298 K and a table reporting the diffusion constants calculated for each peak used to determine the hydrodynamic diameter of the anionic components of **2** (d_H = 1.43 nm). Peaks 1-6 correspond to the anionic component of **2** and peaks 7-10 correspond to the cationic component of **2.**

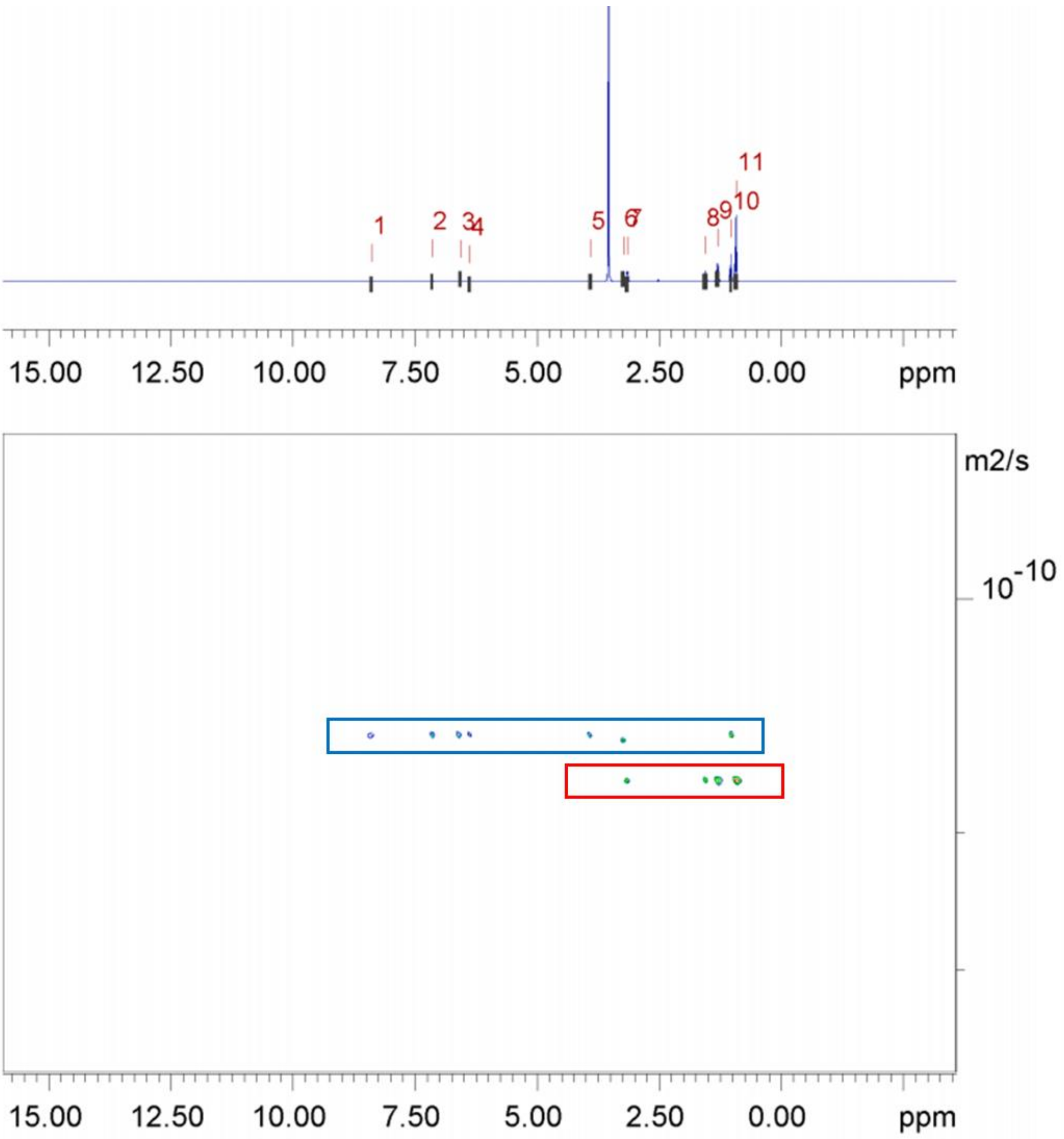

1
2
3
4
5
6
7
8
9
10
11
15.00
12.50
10.00
7.50
5.00
2.50
0.00
ppm
m2/s
10^{-10}

Peak name	F2 [ppm]	Io	error	D [m2/s]	error
1	8.387	1.61e+08	1.107e+04	1.50e-10	2.278e-14
2	7.148	4.53e+08	1.084e+04	1.50e-10	7.911e-15
3	6.573	4.46e+08	1.105e+04	1.49e-10	8.165e-15
4	6.383	1.93e+08	1.106e+04	1.49e-10	1.893e-14
5	3.903	4.21e+08	1.170e+04	1.50e-10	9.203e-15
6	3.234	7.98e+08	1.213e+04	1.51e-10	5.073e-15
7	3.148	1.40e+09	1.259e+04	1.72e-10	3.362e-15
8	1.554	1.60e+09	1.432e+04	1.71e-10	3.344e-15
9	1.302	2.00e+09	1.413e+04	1.71e-10	2.638e-15
10	1.025	1.38e+09	1.038e+04	1.49e-10	2.475e-15
11	0.920	3.45e+09	1.378e+04	1.71e-10	1.490e-15

Figure S60 - ^{1}H DOSY NMR spectrum of compounds **3** (117.61 mM) in DMSO-d_6 at 298 K and a table reporting the diffusion constants calculated for each peak used to determine the hydrodynamic diameter of the anionic components of **3** (d_H = 1.46 nm). Peaks 1-6 and 10 correspond to the anionic component of **3** and peaks 7-9 and 11 correspond to the cationic component of **3**.

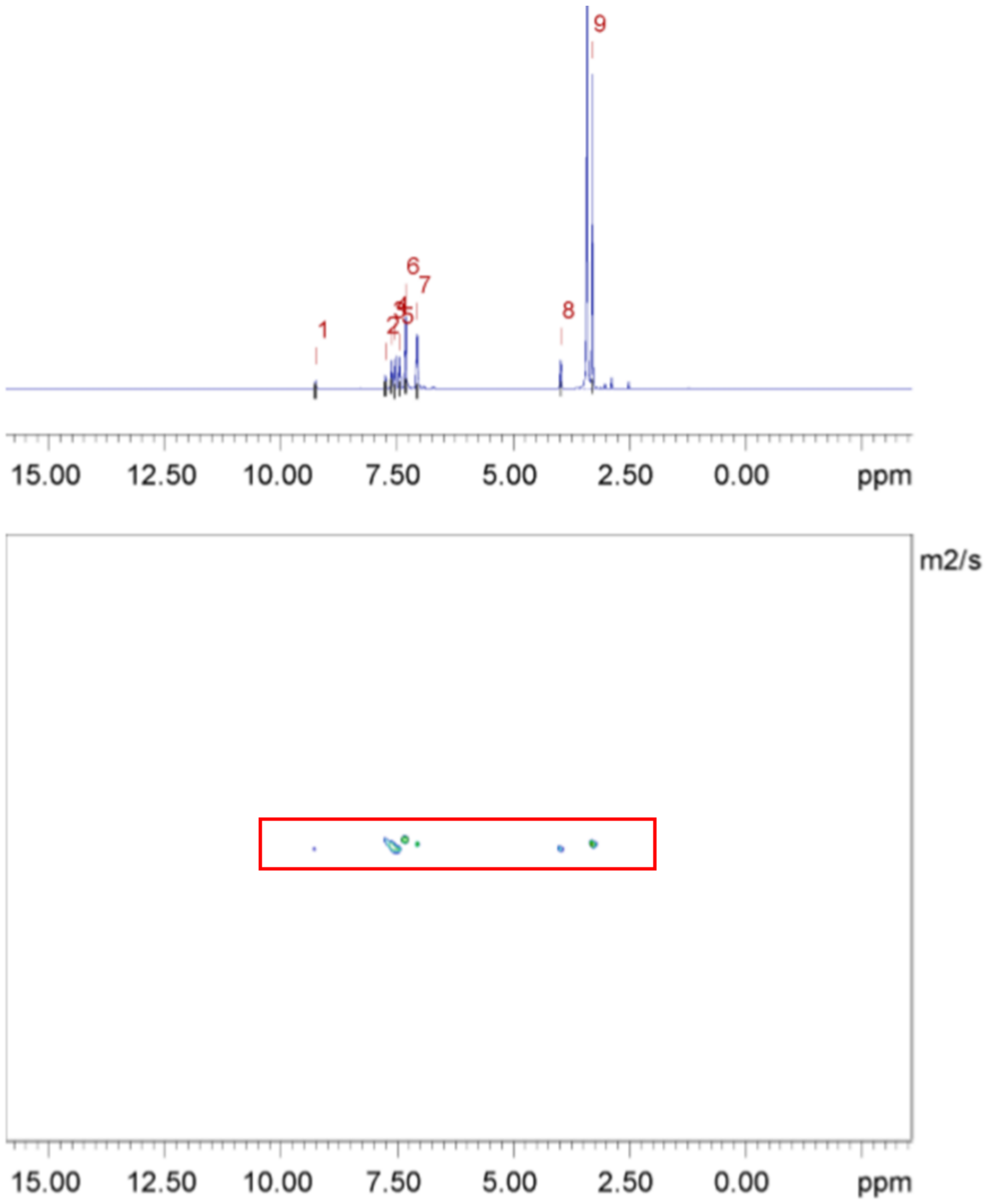
1
2
3
4
5
6
7
8
9
15.00
12.50
10.00
7.50
5.00
2.50
0.00
ppm
m2/s

Peak name	F2 [ppm]	Io	error	D [m2/s]	error
1	9.253	5.70e+08	4.539e+04	2.15e-10	3.765e-14
2	7.753	8.43e+08	4.396e+04	2.10e-10	2.411e-14
3	7.618	1.68e+09	4.054e+04	2.12e-10	1.126e-14
4	7.543	1.70e+09	3.664e+04	2.15e-10	1.018e-14
5	7.443	1.66e+09	3.661e+04	2.14e-10	1.038e-14
6	7.313	4.79e+09	3.902e+04	2.09e-10	3.755e-15
7	7.067	3.66e+09	4.174e+04	2.12e-10	5.314e-15
8	3.974	1.37e+09	3.046e+04	2.14e-10	1.046e-14
9	3.291	8.59e+09	3.347e+04	2.11e-10	1.811e-15

Figure S61 - ^{1}H DOSY NMR spectrum of compound **10** (109.88 mM) in DMSO-d_6 at 298 K and a table reporting the diffusion constants calculated for each peak used to determine the hydrodynamic diameter of the anionic components (d_H = 1.02 nm) and the cationic components (d_H = 1.04 nm) of **10**. Peaks 1, 4-5 and 8 correspond to the anionic component of **10** and peaks 2-3, 6-7 and 9 correspond to the cationic component of **10**.

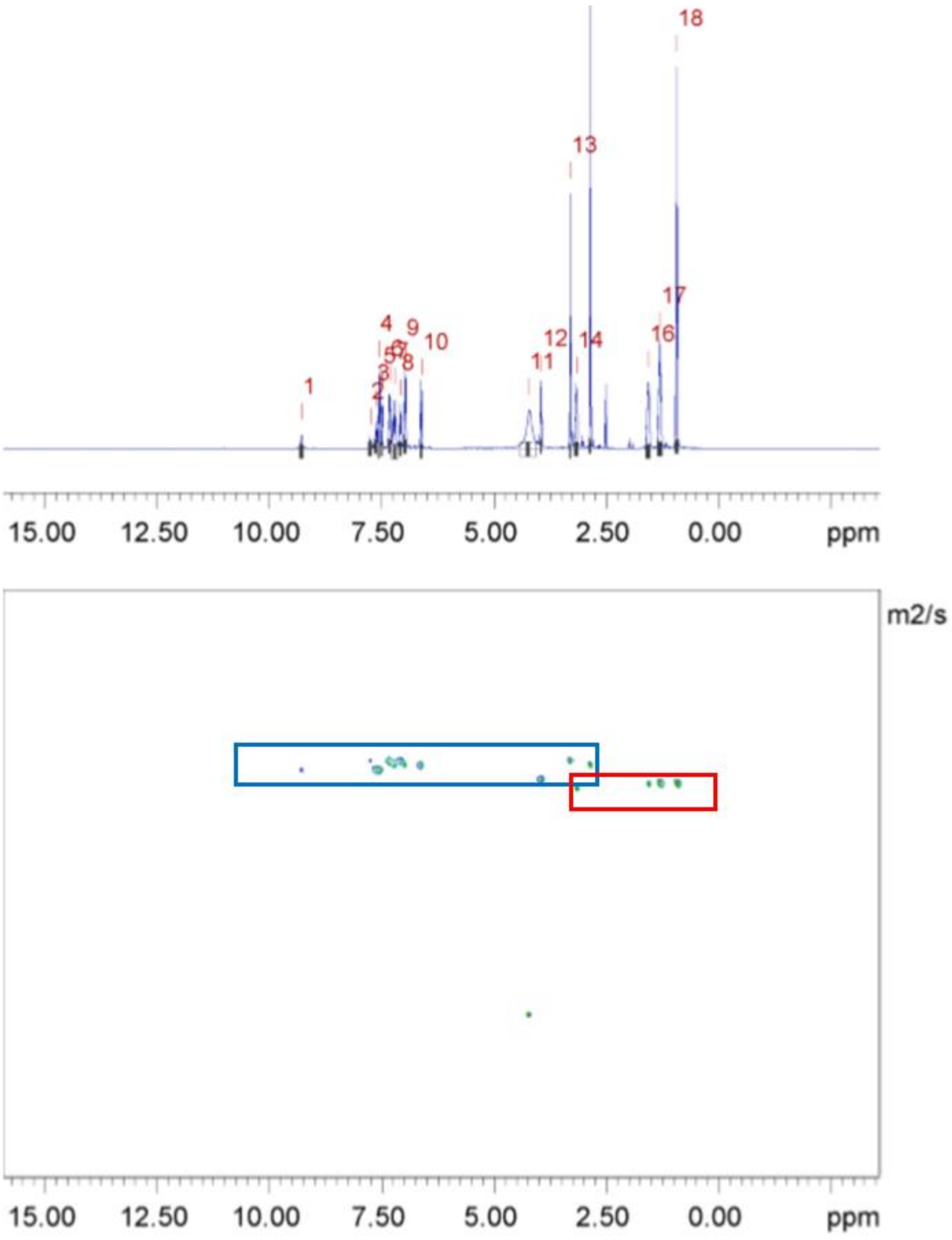

1
2
3
4
5
6
7
8
9
10
11
12
13
14
16
17
18
15.00
12.50
10.00
7.50
5.00
2.50
0.00
ppm
m2/s

Peak name	F2 [ppm]	Io	error	D [m2/s]	error
1	9.283	1.81e+09	3.539e+05	1.57e-10	6.696e-14
2	7.757	1.32e+09	2.955e+05	1.50e-10	7.398e-14
3	7.623	2.97e+09	2.814e+05	1.57e-10	3.241e-14
4	7.562	6.22e+09	2.483e+05	1.58e-10	1.375e-14
5	7.494	6.28e+09	2.426e+05	1.58e-10	1.337e-14
6	7.322	7.46e+09	2.644e+05	1.50e-10	1.167e-14
7	7.212	8.91e+09	4.219e+05	1.56e-10	1.615e-14
8	7.078	4.55e+09	2.829e+05	1.51e-10	2.056e-14
9	6.974	8.85e+09	2.764e+05	1.56e-10	1.063e-14
10	6.616	5.92e+09	2.423e+05	1.56e-10	1.395e-14
11	4.239	2.26e+10	9.961e+05	5.81e-10	5.259e-14
12	3.951	5.54e+09	2.180e+05	1.65e-10	1.415e-14
13	3.296	1.17e+10	2.127e+05	1.51e-10	6.009e-15
14	3.163	1.20e+10	3.373e+05	1.74e-10	1.061e-14
15	2.854	1.82e+10	2.574e+05	1.56e-10	4.803e-15
16	1.562	1.44e+10	3.802e+05	1.71e-10	9.779e-15
17	1.304	1.93e+10	3.900e+05	1.71e-10	7.495e-15
18	0.929	3.37e+10	3.148e+05	1.70e-10	3.462e-15

Figure S62 - ^{1}H DOSY NMR spectrum of co-formulation ***a*** (111.12 mM) in DMSO-d_6 at 298 K and a table reporting the diffusion constants calculated for each peak used to determine the hydrodynamic diameter of the anionic components (d_H = 1.42 nm) of co-formulation ***a***. Peaks 1-10, 12-13 and 15 correspond to the anionic component of co-formulation ***a*** and malachite green**,** peaks 14 and 16-18 correspond to the cationic TBA.

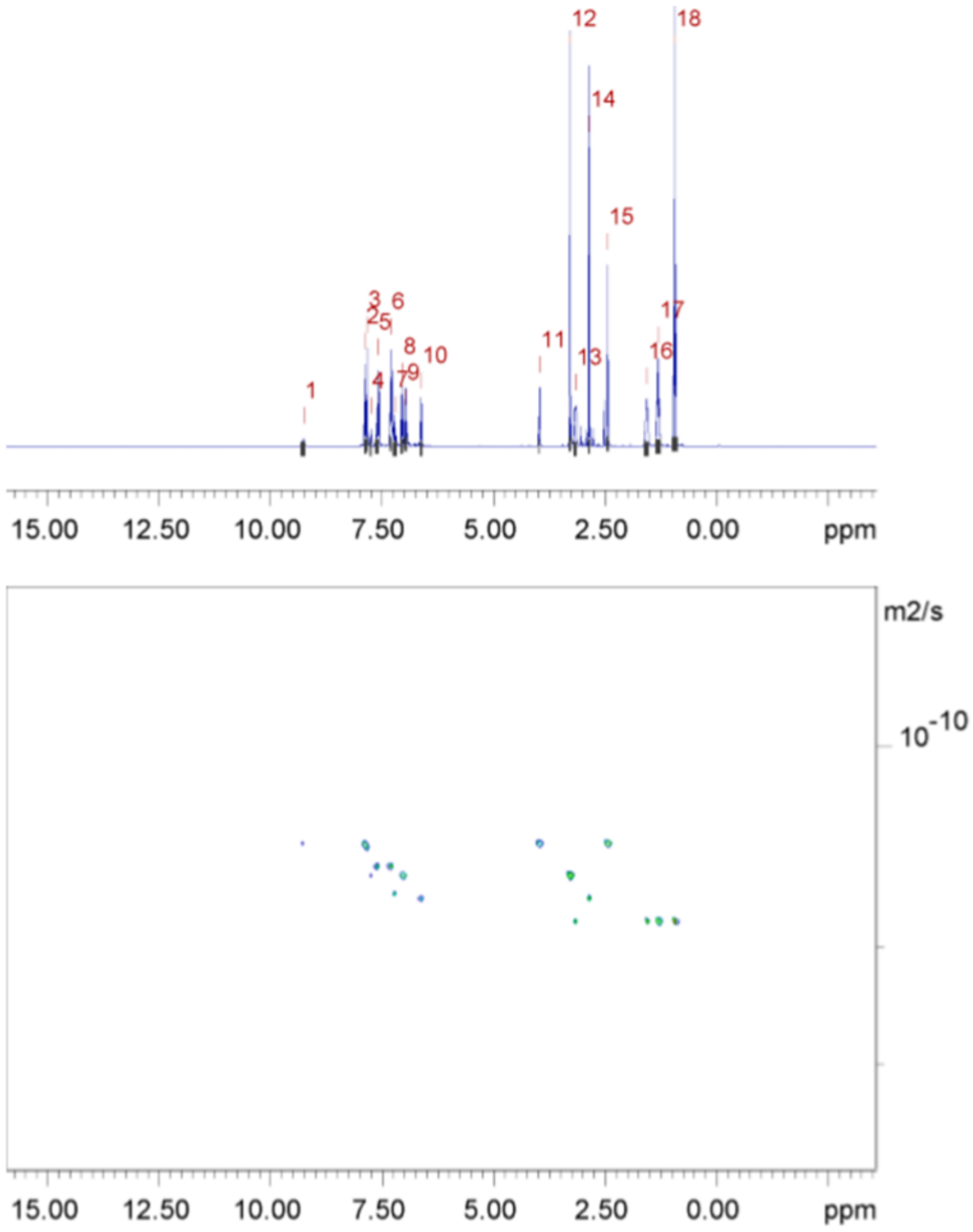

1
2
3
4
5
6
7
8
9
10
11
12
13
14
15
16
17
18
15.00
12.50
10.00
7.50
5.00
2.50
0.00
ppm
m2/s
10^{-10}

Peak name	F2 [ppm]	Io	error	D [m2/s]	error
1	9.260	8.57e+08	1.819e+05	1.41e-10	6.616e-14
2	7.867	3.69e+09	1.200e+05	1.40e-10	1.011e-14
3	7.826	2.23e+09	9.960e+04	1.41e-10	1.393e-14
4	7.751	9.16e+08	1.272e+05	1.56e-10	4.744e-14
5	7.607	3.84e+09	1.542e+05	1.51e-10	1.336e-14
6	7.303	4.98e+09	1.152e+05	1.51e-10	7.683e-15
7	7.211	2.72e+09	1.758e+05	1.65e-10	2.333e-14
8	7.050	2.79e+09	1.164e+05	1.57e-10	1.431e-14
9	6.963	3.32e+09	1.558e+05	1.56e-10	1.605e-14
10	6.617	1.82e+09	1.194e+05	1.69e-10	2.409e-14
11	3.967	2.50e+09	1.012e+05	1.40e-10	1.260e-14
12	3.280	8.55e+09	1.274e+05	1.57e-10	5.124e-15
13	3.158	5.61e+09	1.537e+05	1.82e-10	1.079e-14
14	2.849	5.59e+09	1.189e+05	1.69e-10	7.816e-15
15	2.436	4.40e+09	1.236e+05	1.40e-10	8.750e-15
16	1.566	7.23e+09	1.883e+05	1.83e-10	1.029e-14
17	1.305	9.78e+09	2.031e+05	1.82e-10	8.201e-15
18	0.931	1.75e+10	2.170e+05	1.82e-10	4.890e-15

Figure S63 - DOSY NMR spectrum of co-formulation ***b*** (111.12 mM) in DMSO-d_6 at 298 K and a table reporting the diffusion constants calculated for each peak. The hydrodynamic diameter could not be determined due to peak overlap.

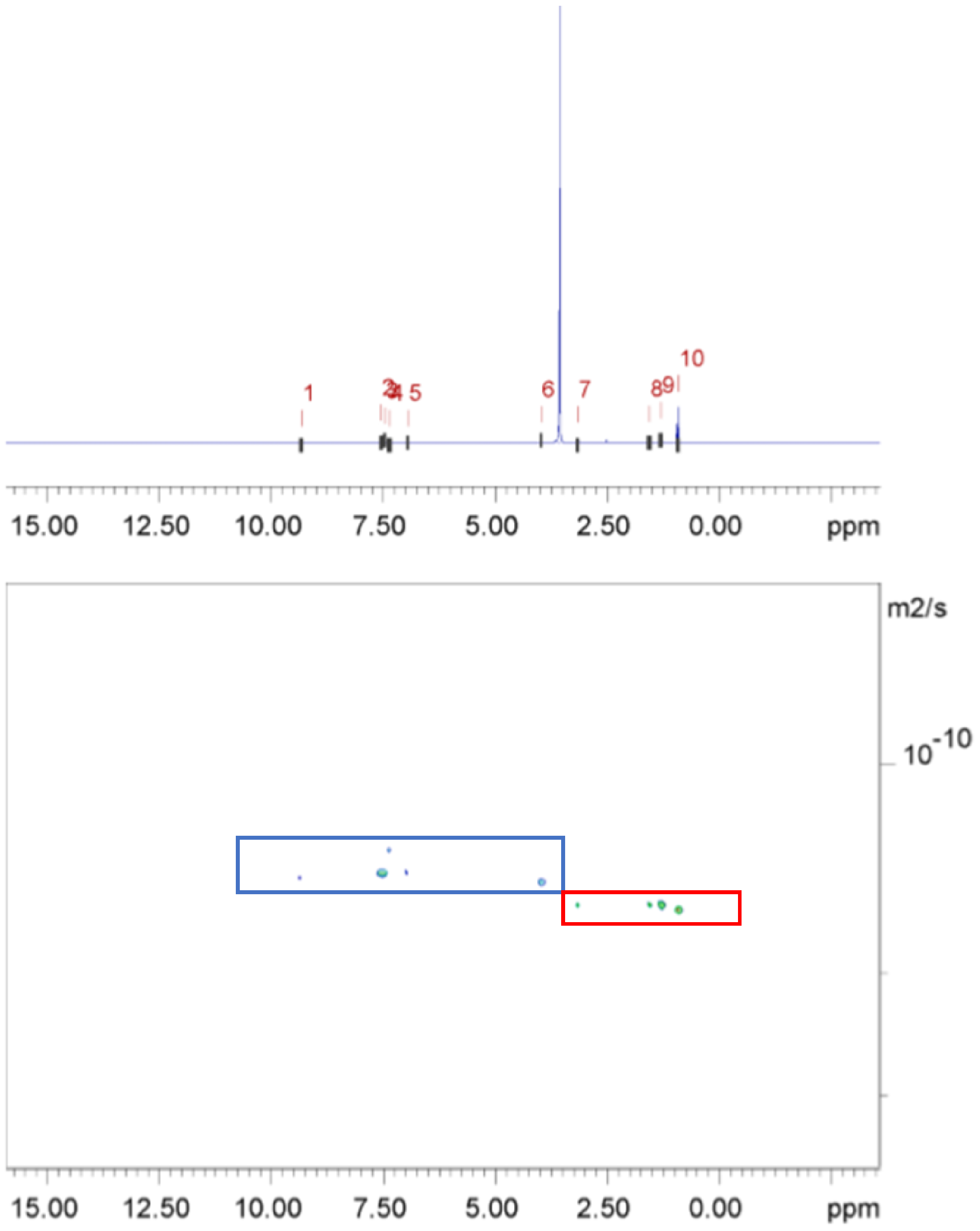
1
2
3
4
5
6
7
8
9
10
15.00
12.50
10.00
7.50
5.00
2.50
0.00
ppm
m2/s
10-10
15.00
12.50
10.00
7.50
5.00
2.50
0.00
ppm

Peak name	F2 [ppm]	Io	error	D [m2/s]	error
1	9.331	1.34e+08	1.593e+05	1.45e-10	3.790e-13
2	7.543	4.26e+08	2.750e+05	1.44e-10	2.043e-13
3	7.466	4.15e+08	3.739e+05	1.43e-10	2.839e-13
4	7.359	2.23e+08	2.357e+05	1.34e-10	3.133e-13
5	6.955	1.60e+08	1.151e+05	1.43e-10	2.264e-13
6	3.971	3.48e+08	8.687e+05	1.47e-10	8.059e-13
7	3.157	8.19e+08	1.308e+06	1.60e-10	5.576e-13
8	1.553	9.76e+08	1.118e+06	1.61e-10	4.017e-13
9	1.297	1.29e+09	1.441e+06	1.61e-10	3.932e-13
10	0.918	2.20e+09	2.147e+06	1.61e-10	3.430e-13

Figure S64 - ^{1}H DOSY NMR spectrum of co-formulation ***c*** (113.70 mM) in DMSO-d_6 at 298 K and a table reporting the diffusion constants calculated for each peak used to determine the hydrodynamic diameter of the anionic components (d_H = 1.52 nm) and the cationic components (d_H = 1.36 nm) of co-formulation ***c***. Peaks 1-6 correspond to the anionic component of co-formulation ***c*** and methylene blue**,** peaks 7-10 correspond to the cationic TBA.

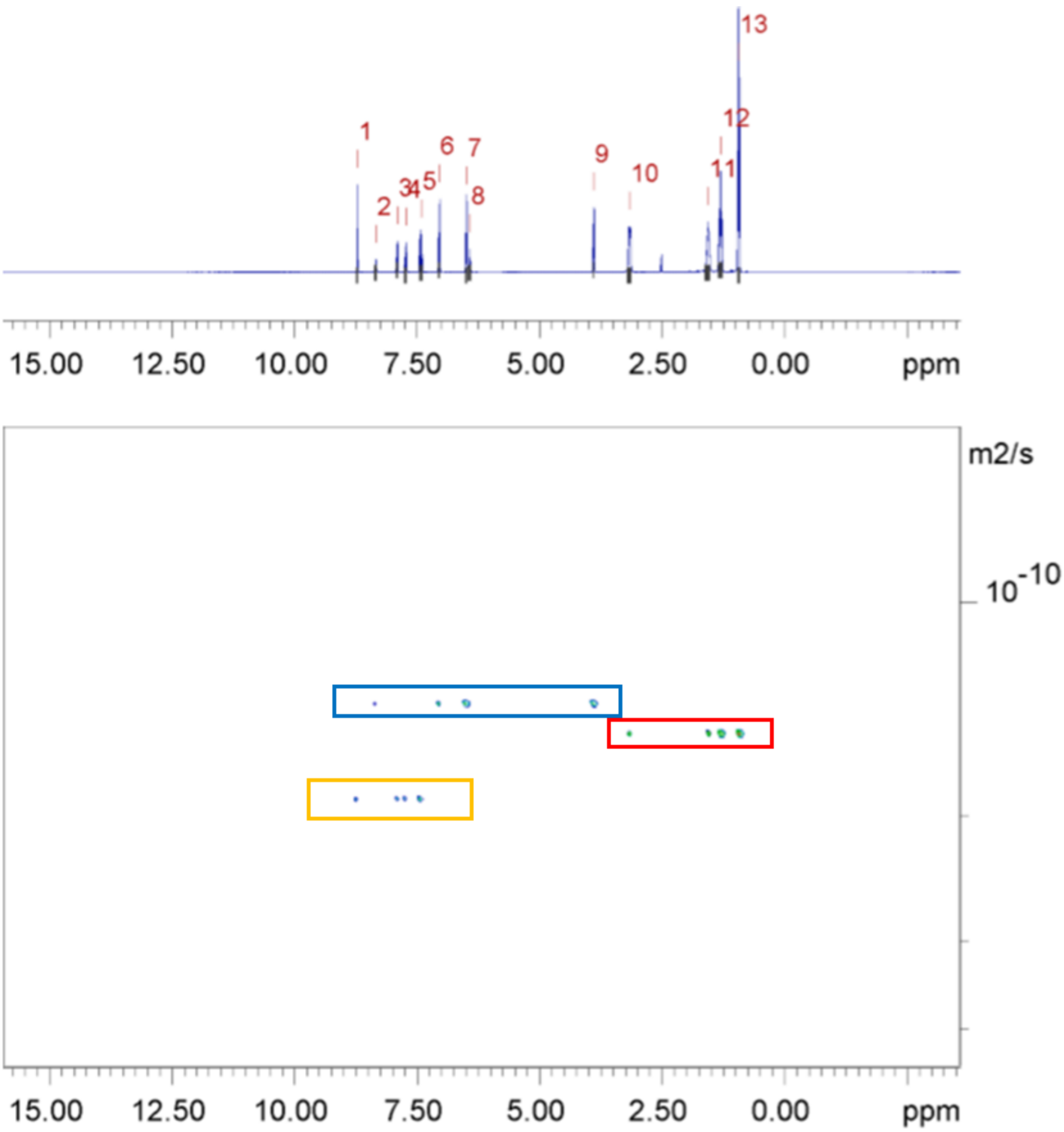
1
2
3
4
5
6
7
8
9
10
11
12
13
15.00
12.50
10.00
7.50
5.00
2.50
0.00
ppm
m2/s
10-10

Peak name	F2 [ppm]	Io	error	D [m2/s]	error
1	8.711	1.51e+09	5.860e+04	1.91e-10	1.555e-14
2	8.340	9.57e+08	6.797e+04	1.38e-10	2.105e-14
3	7.900	1.59e+09	6.310e+04	1.91e-10	1.592e-14
4	7.725	1.61e+09	7.318e+04	1.90e-10	1.818e-14
5	7.412	3.03e+09	8.146e+04	1.91e-10	1.081e-14
6	7.043	3.07e+09	5.966e+04	1.38e-10	5.738e-15
7	6.485	3.05e+09	5.597e+04	1.38e-10	5.423e-15
8	6.421	1.26e+09	6.900e+04	1.38e-10	1.625e-14
9	3.888	2.70e+09	5.311e+04	1.38e-10	5.812e-15
10	3.158	6.85e+09	9.023e+04	1.53e-10	4.272e-15
11	1.562	8.18e+09	9.697e+04	1.52e-10	3.844e-15
12	1.302	1.08e+10	9.489e+04	1.53e-10	2.846e-15
13	0.928	1.82e+10	7.370e+04	1.52e-10	1.315e-15

Figure 65 - ^{1}H DOSY NMR spectrum of co-formulation ***e*** (110.88 mM) in DMSO-d_6 at 298 K and a table reporting the diffusion constants calculated for each peak used to determine the hydrodynamic diameter of the zwitterionic components of co-formulation ***e*** (d_H = 1.58 nm) and coumarin (d_H = 1.15 nm). Peaks 2, 6-9 correspond to the zwitterionic component of co-formulation ***e*,** peaks 10-13 correspond to the cationic component of co-formulation ***e*** and peaks 1 and 3-5 correspond to the anionic coumarin.

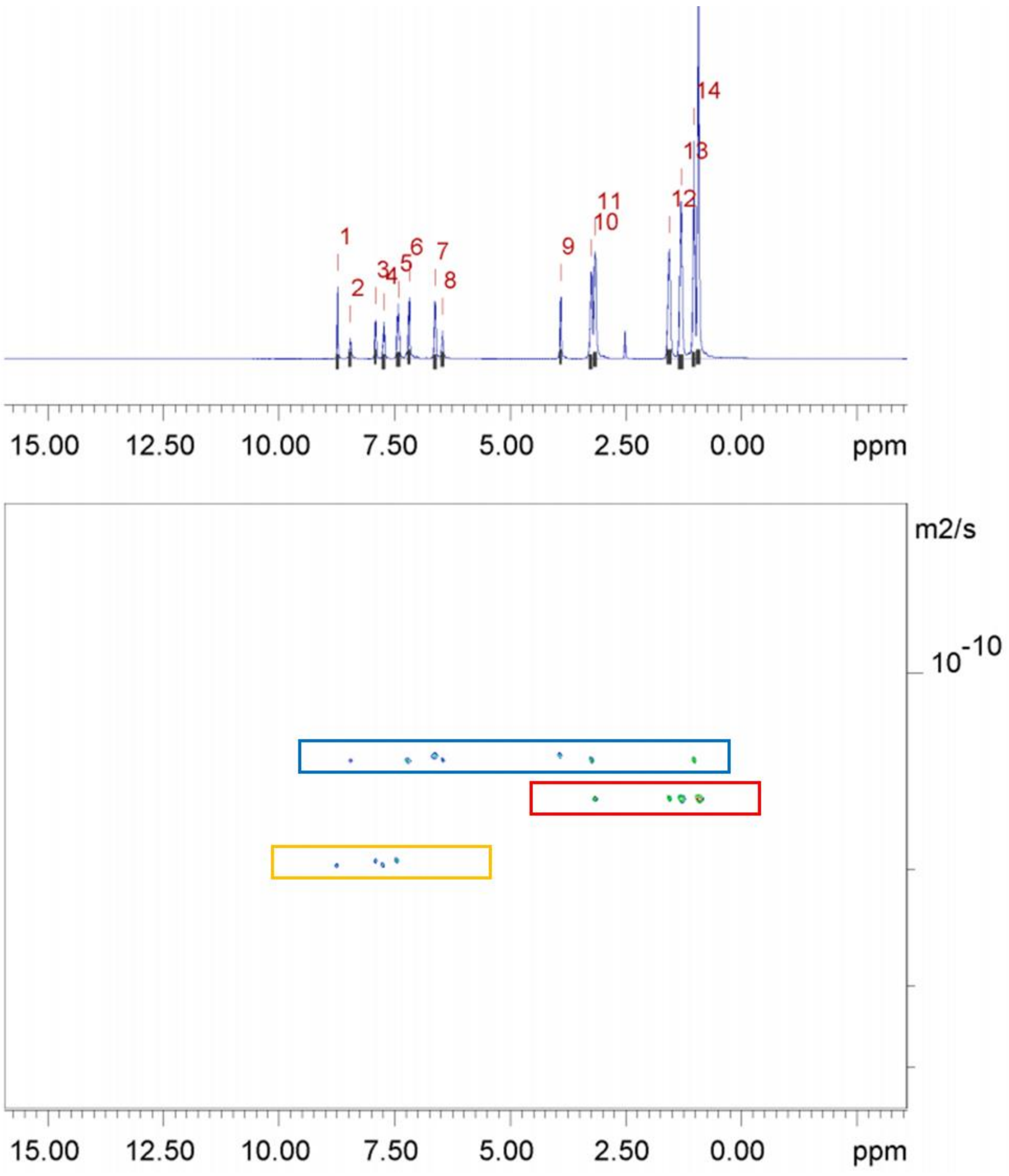
1
2
3
4
5
6
7
8
9
10
11
12
13
14
15.00
12.50
10.00
7.50
5.00
2.50
0.00
ppm
m2/s
10^{-10}

Peak name	F2 [ppm]	Io	error	D [m2/s]	error
1	8.732	2.63e+09	1.631e+05	1.95e-10	2.498e-14
2	8.452	1.58e+09	1.474e+05	1.35e-10	2.690e-14
3	7.906	2.75e+09	1.604e+05	1.95e-10	2.338e-14
4	7.728	2.83e+09	1.802e+05	1.96e-10	2.565e-14
5	7.416	5.53e+09	1.973e+05	1.95e-10	1.434e-14
6	7.183	4.81e+09	1.500e+05	1.36e-10	9.031e-15
7	6.618	4.88e+09	1.547e+05	1.35e-10	9.107e-15
8	6.456	2.23e+09	1.600e+05	1.36e-10	2.070e-14
9	3.902	4.35e+09	1.447e+05	1.35e-10	9.571e-15
10	3.246	8.57e+09	1.601e+05	1.36e-10	5.400e-15
11	3.159	1.36e+10	1.733e+05	1.56e-10	4.155e-15
12	1.554	1.54e+10	2.025e+05	1.57e-10	4.320e-15
13	1.303	2.02e+10	2.125e+05	1.57e-10	3.454e-15
14	1.026	1.61e+10	1.596e+05	1.35e-10	2.857e-15
15	0.919	3.41e+10	1.849e+05	1.56e-10	1.771e-15

Figure S66 - ^{1}H DOSY NMR spectrum of co-formulation ***f*** (111.12 mM) in DMSO-d_6 at 298 K and a table reporting the diffusion constants calculated for each peak used to determine the hydrodynamic diameter of the zwitterionic components of co-formulation ***f*** (d_H = 1.62 nm) and coumarin (d_H = 1.12 nm). Peaks 2, 6-10 and 14 correspond to the zwitterionic component of co-formulation ***f***, peaks 11-13 and 15 correspond to the cationic component of co-formulation ***f*** and peaks 1, and 3-5 correspond to the anionic coumarin.

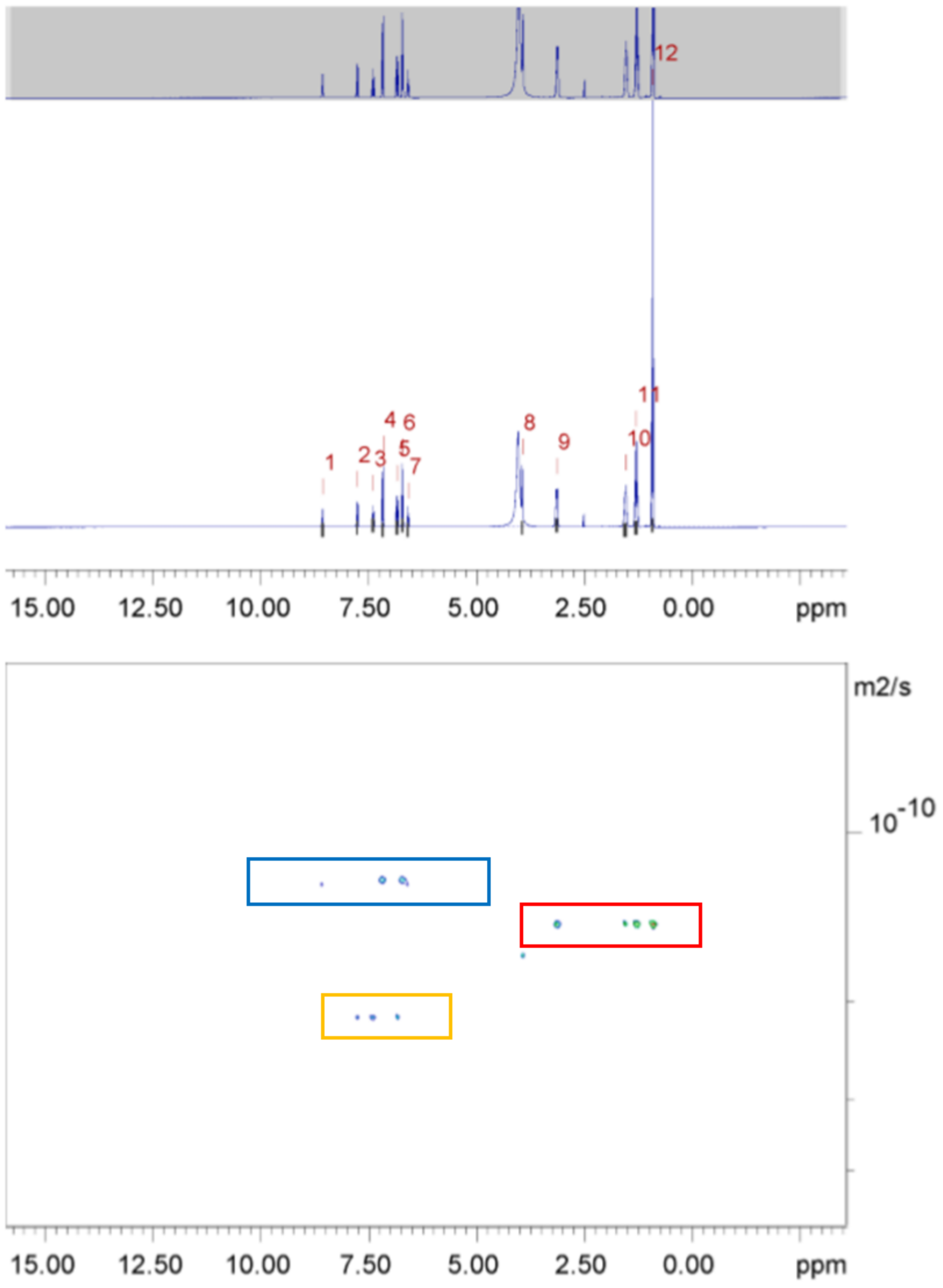
12
1
2 3
4 6
5
7
8
9
11
10
15.00
12.50
10.00
7.50
5.00
2.50
0.00
ppm
m2/s
10^{-10}
15.00
12.50
10.00
7.50
5.00
2.50
0.00
ppm

Peak name	F2 [ppm]	Io	error	D [m2/s]	error
1	8.563	1.18e+09	7.841e+04	1.23e-10	1.736e-14
2	7.763	1.52e+09	7.543e+04	2.13e-10	2.143e-14
3	7.396	1.52e+09	8.657e+04	2.12e-10	2.453e-14
4	7.170	3.23e+09	6.672e+04	1.23e-10	5.380e-15
5	6.836	2.97e+09	8.801e+04	2.12e-10	1.276e-14
6	6.713	3.17e+09	6.096e+04	1.22e-10	4.979e-15
7	6.585	1.29e+09	6.839e+04	1.24e-10	1.393e-14
8	3.935	3.65e+09	5.390e+04	1.67e-10	5.083e-15
9	3.134	6.80e+09	8.116e+04	1.47e-10	3.652e-15
10	1.541	8.13e+09	9.170e+04	1.47e-10	3.447e-15
11	1.296	1.02e+10	8.226e+04	1.47e-10	2.459e-15
12	0.915	1.85e+10	7.586e+04	1.47e-10	1.256e-15

Figure S67 - ^{1}H DOSY NMR spectrum of co-formulation ***g*** (110.88 mM) in DMSO-d_6 at 298 K and a table reporting the diffusion constants calculated for each peak used to determine the hydrodynamic diameter of the zwitterionic components of co-formulation ***g*** (d_H = 1.78 nm) and salicylic acid (d_H = 1.03 nm). Peaks 1, 4, and 6 -8 correspond to the zwitterionic component of co-formulation ***g*,** peaks 9-12 correspond to the cationic component of co-formulation ***g*** and peaks 2, 3, and 5 correspond to the anionic salicylic acid.

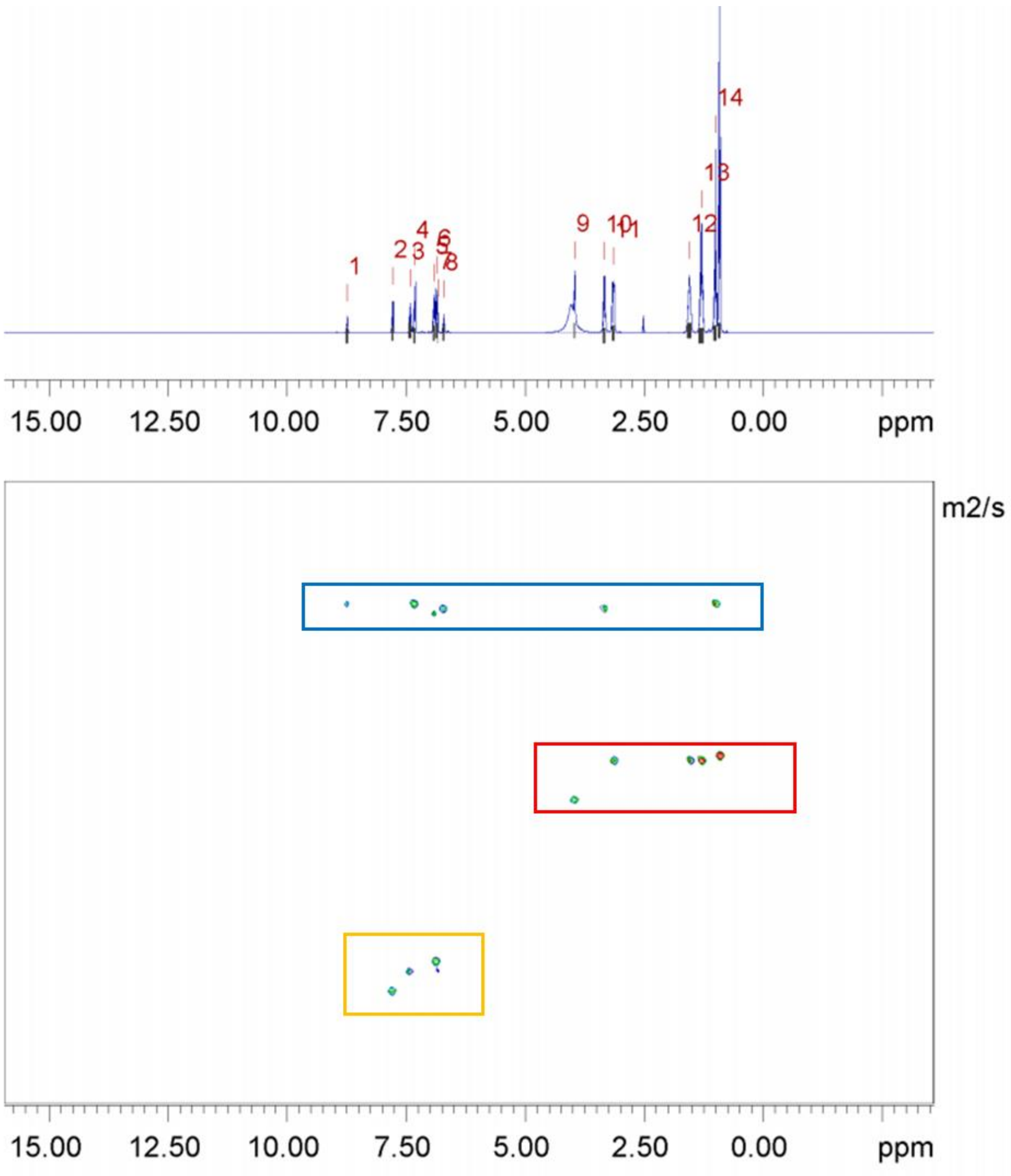
1
2
3
4
5
6
8
9
10
11
12
13
14
15.00
12.50
10.00
7.50
5.00
2.50
0.00
ppm
m2/s

Peak name	F2 [ppm]	Io	error	D [m2/s]	error
1	8.735	8.37e+08	5.995e+04	1.18e-10	1.862e-14
2	7.779	1.52e+09	6.372e+04	2.15e-10	1.918e-14
3	7.411	1.61e+09	7.622e+04	2.08e-10	2.096e-14
4	7.313	2.44e+09	5.672e+04	1.18e-10	6.035e-15
5	6.911	2.08e+09	5.350e+04	1.19e-10	6.746e-15
6	6.858	2.79e+09	6.626e+04	2.05e-10	1.037e-14
7	6.824	3.34e+08	3.277e+04	2.09e-10	4.376e-14
8	6.705	9.73e+08	5.738e+04	1.19e-10	1.539e-14
9	3.952	2.54e+09	4.735e+04	1.60e-10	6.437e-15
10	3.331	3.91e+09	6.705e+04	1.19e-10	4.465e-15
11	3.141	5.98e+09	7.241e+04	1.50e-10	3.934e-15
12	1.542	7.58e+09	9.164e+04	1.50e-10	3.923e-15
13	1.292	1.04e+10	9.338e+04	1.50e-10	2.918e-15
14	1.002	7.88e+09	6.614e+04	1.18e-10	2.176e-15
15	0.916	1.74e+10	7.285e+04	1.50e-10	1.355e-15

Figure S68 - ^{1}H DOSY NMR spectrum of co-formulation ***h*** (109.38 mM) in DMSO-d_6 at 298 K and a table reporting the diffusion constants calculated for each peak used to determine the hydrodynamic diameter of the zwitterionic components of co-formulation ***h*** (d_H = 1.84 nm) and salicylic acid (d_H = 1.05 nm). Peaks 1, 4, 5, 8, 10 and 14 correspond to the zwitterionic component of co-formulation ***h***, peaks 9, 11-13 and 15 correspond to the cationic component of co-formulation ***h*** and peaks 2, 3, 6 and 7 correspond to the anionic salicylic acid.

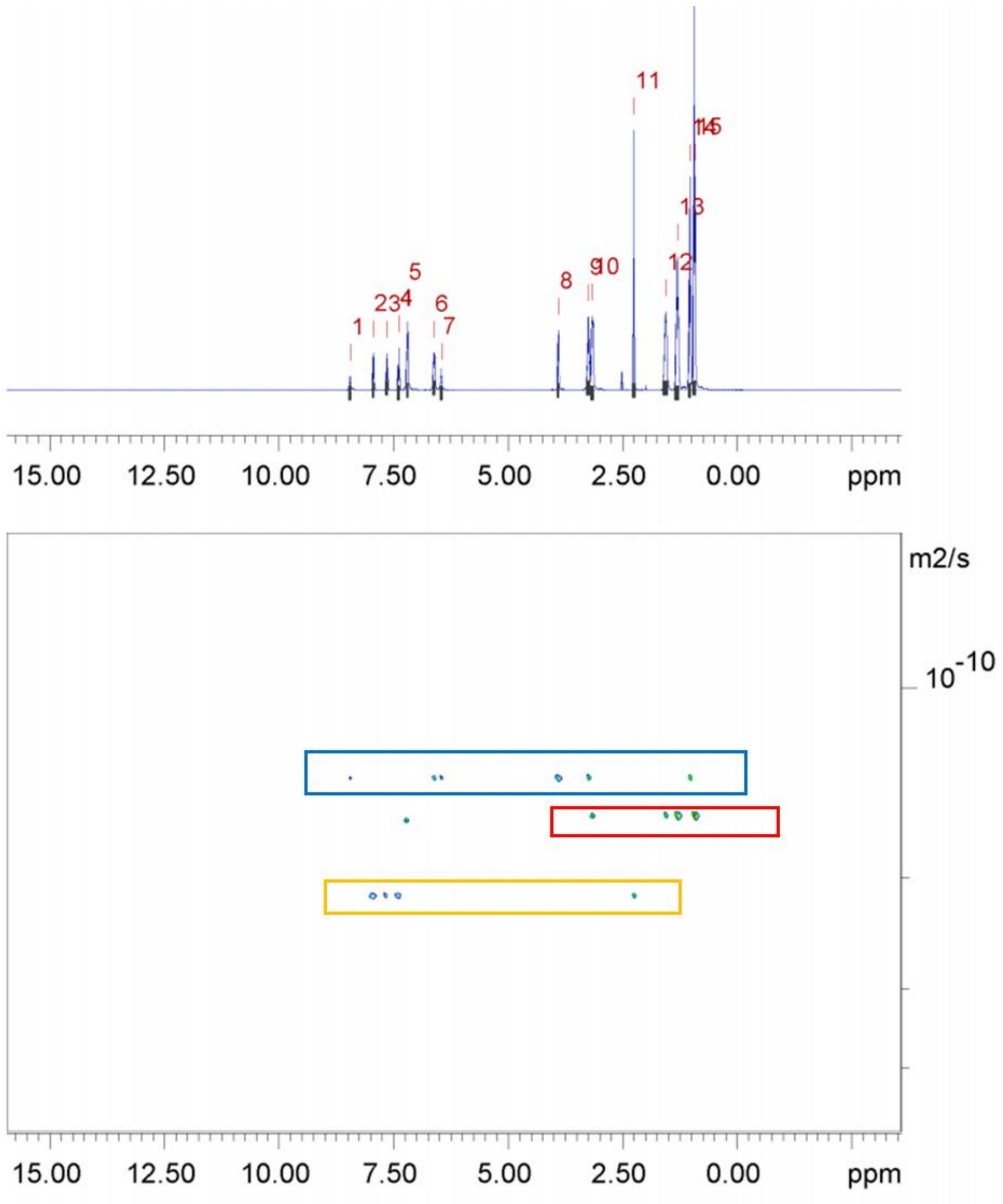

1
23
4
5
6
7
8
9
10
11
12
13
14
15
15.00
12.50
10.00
7.50
5.00
2.50
0.00
ppm
m2/s
10^{-10}

Peak name	F2 [ppm]	Io	error	D [m2/s]	error
1	8.442	9.38e+08	9.736e+04	1.39e-10	3.014e-14
2	7.935	1.90e+09	9.542e+04	2.14e-10	2.197e-14
3	7.639	2.07e+09	1.092e+05	2.13e-10	2.309e-14
4	7.382	2.11e+09	1.093e+05	2.12e-10	2.248e-14
5	7.187	5.01e+09	9.561e+04	1.61e-10	6.376e-15
6	6.610	3.02e+09	9.586e+04	1.39e-10	9.211e-15
7	6.452	1.41e+09	9.590e+04	1.39e-10	1.984e-14
8	3.898	2.98e+09	8.686e+04	1.39e-10	8.497e-15
9	3.246	5.75e+09	1.108e+05	1.39e-10	5.615e-15
10	3.156	8.43e+09	1.123e+05	1.58e-10	4.376e-15
11	2.246	5.90e+09	1.216e+05	2.12e-10	8.941e-15
12	1.558	9.78e+09	1.312e+05	1.60e-10	4.439e-15
13	1.304	1.31e+10	1.289e+05	1.59e-10	3.242e-15
14	1.029	1.06e+10	9.461e+04	1.39e-10	2.606e-15
15	0.925	2.26e+10	1.124e+05	1.59e-10	1.638e-15

Figure S69 - ^{1}H DOSY NMR spectrum of compounds co-formulation ***j*** (111.23 mM) in DMSO-d_6 at 298 K and a table reporting the diffusion constants calculated for each peak used to determine the hydrodynamic diameter of the zwitterionic components co-formulation ***j*** (d_H = 1.58 nm) and acetylsalicylic acid (d_H = 1.03 nm). Peaks 1, 6-9 and 14 correspond to the zwitterionic component of co-formulation ***j***, peaks 10, 12-13 and 15 correspond to the cationic component of co-formulation ***j*** and peaks 2-5 and 11 correspond to the anionic acetylsalicylic acid.

Overview

Table S4 - Overview of diffusion coefficients (m^2s^{-1}) for compounds **1-11** and co-formulation ***a-j*** in DMSO-d_6 at 298 K. Errors for diffusion constants are no greater than ± 1 x 10^{-13} m^2s^{-1}.

	Diffusion Coefficient (m^2s^{-1})		
**Compound**	**Anion**	**TBA**	**Guest**
**2**	1.53 x 10^{-10}	1.66 x 10^{-10}	n/a
**3**	1.50 x 10^{-10}	1.71 x 10^{-10}	n/a
**10**	2.12 x 10^{-10}	n/a	2.12 x 10^{-10}
a	1.54 x 10^{-10}	1.72 x 10^{-10}	1.54 x 10^{-10}
c	1.42 x 10^{-10}	1.61 x 10^{-10}	1.43 x 10^{-10}
e	1.38 x 10^{-10}	1.53 x 10^{-10}	1.91 x 10^{-10}
f	1.36 x 10^{-10}	1.57 x 10^{-10}	1.95 x 10^{-10}
g	1.23 x 10^{-10}	1.47 x 10^{-10}	2.12 x 10^{-10}
h	1.18 x 10^{-10}	1.50 x 10^{-10}	2.09 x 10^{-10}
j	1.39 x 10^{-10}	1.59 x 10^{-10}	2.13 x 10^{-10}

Table S5 - Overview of hydrodynamic diameters (nm) for compounds **1-11** and co-formulation ***a-j*** in DMSO-d_6 at 298 K.

Co-formulation	Compound	Anion	Cation	Co-formulant	Anion	Cation
n/a	**1** [1]	1.15	1.08	n/a	n/a	n/a
n/a	**2** [2]	1.43	1.32	n/a	n/a	n/a
n/a	**3**	1.46	2.38	n/a	n/a	n/a
n/a	**4** [3]	1.61	1.51	n/a	n/a	n/a

n/a	**5**	*a*	*a*	n/a	n/a	n/a
n/a	**6**	*a*	*a*	n/a	n/a	n/a
n/a	**7**	*a*	*a*	n/a	n/a	n/a
n/a	**8**	*a*	*a*	n/a	n/a	n/a
n/a	**9**	*a*	*a*	n/a	n/a	n/a
n/a	**10**	1.02	1.04	n/a	n/a	n/a
n/a	**11**	*b*	*b*	n/a	n/a	n/a
**a**	**1**	1.42	1.27	**5**	1.54	
**b**	**4**	*b*	*b*	**5**	*b*	*b*
**c**	**1**	1.52	1.36	**6**	1.52	
**d**	**4**	*c*	*c*	**6**	*c*	*c*
**e**	**2**	1.59	1.43	**7**	1.15	n/a
**f**	**3**	1.62	1.39	**7**	1.12	n/a
**g**	**2**	1.78	1.49	**8**	1.03	n/a
**h**	**3**	1.84	1.46	**8**	1.05	n/a
**i**	**2**	*c*	*c*	**9**	*c*	*c*
**j**	**3**	1.58	1.38	**9**	1.03	n/a

Cells have been merged where compound/co-formulant is neither anionic nor cationic.

a – studies not performed.

b – could not be determined due to peak overlap.

c – Loss of compound observed in ^{1}H quantitative NMR studies.

^{1}H NMR self-association studies

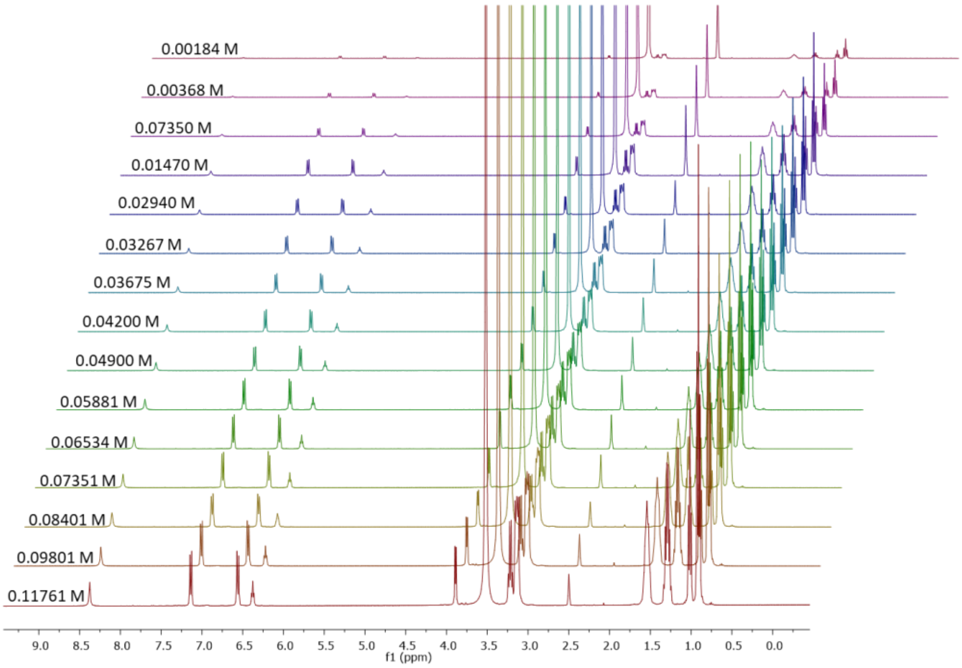

Figure S70 - ^{1}H NMR stack plot of compound **3** in DMSO-*d*$_6$ 0.5 % H_2O solution. Samples were prepared in series with an aliquot of the most concentrated solution undergoing serial dilution.

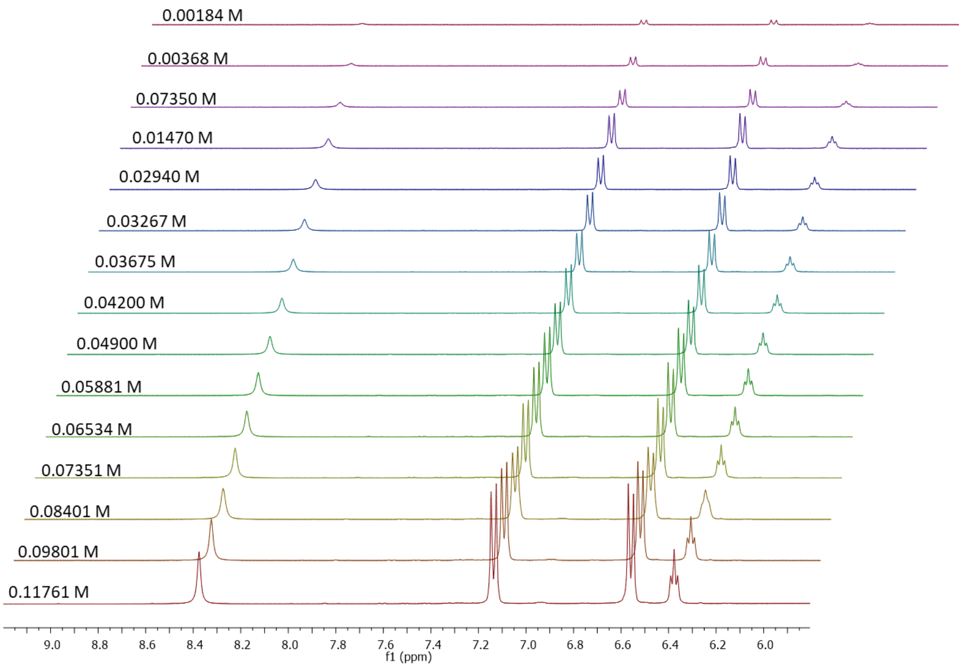

Figure S71 - Enlarged ^{1}H NMR stack plot of compound **3** in DMSO-*d*$_6$ 0.5 % H_2O solution. Samples were prepared in series with an aliquot of the most concentrated solution undergoing serial dilution.

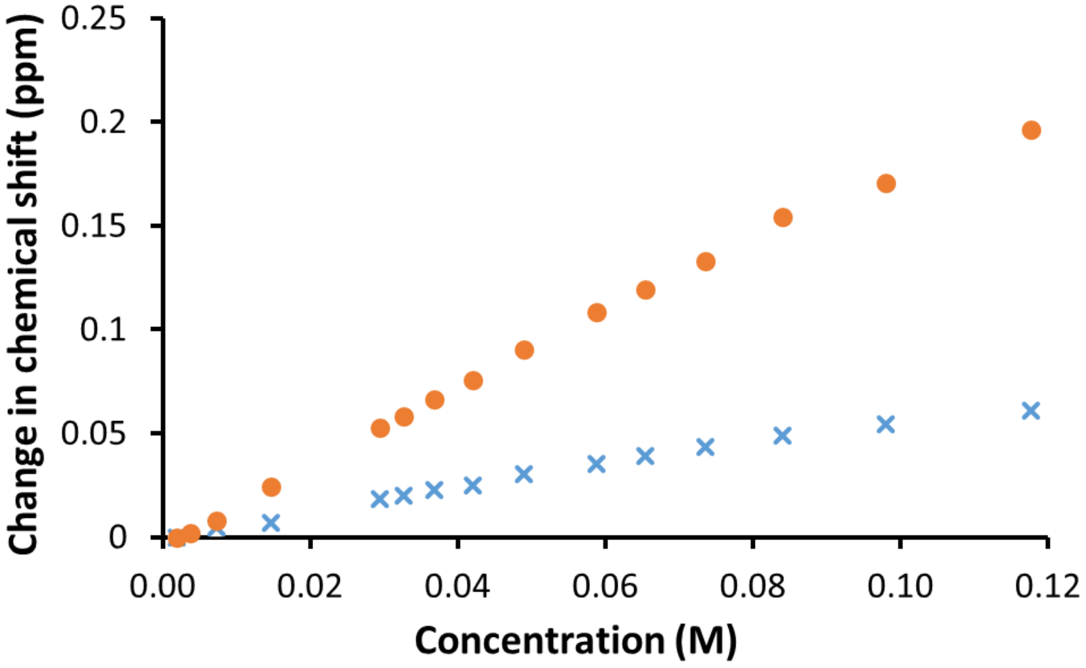

Figure S72 - Graph illustrating the ^{1}H NMR down-field change in chemical shift of urea NH resonances with increasing concentration of compound **3** in DMSO-d_6 0.5 % H_2O (298 K).

Self-association constant calculation

Compound **3** - Dilution study in DMSO-d_6 5 % H_2O. Values calculated from data gathered from both NH 1 and 2.

Equal K/Dimerization model

K_e = 1.19 M^{-1} ± 0.9941 % K_{dim} = 0.59 M^{-1} ± 0.4970 %

http://app.supramolecular.org/bindfit/view/4f441027-52b5-448b-afd0-908fb99a627a

CoEK model

K_e = 8.93 M^{-1} ± 2.1222 % K_{dim} = 4.46 M^{-1} ± 1.0611 %   ρ = 0.33 ± 5.6903 %

http://app.supramolecular.org/bindfit/view/4d3b7f73-fed7-4f4d-af3e-5f33cc8525a8

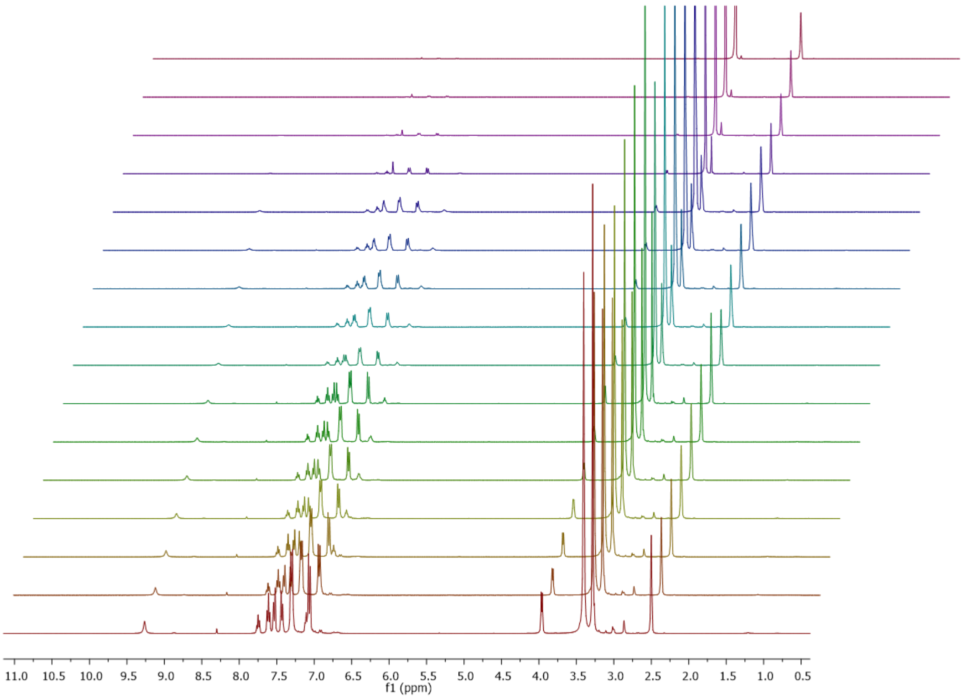

Figure S73 - ^{1}H NMR stack plot of compound **10** in DMSO-d_6 0.5 % H_2O solution. Samples were prepared in series with an aliquot of the most concentrated solution undergoing serial dilution.

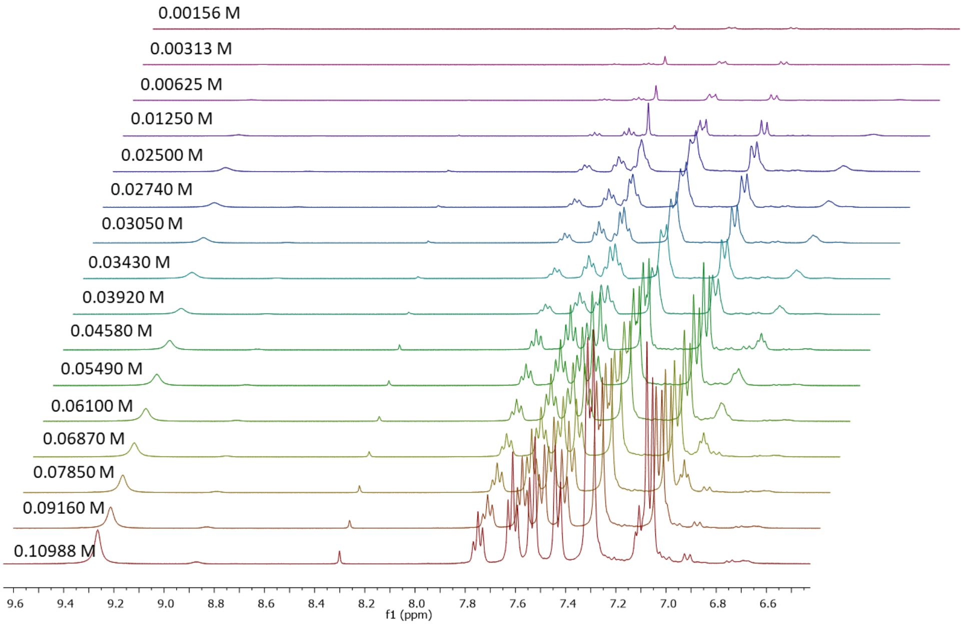

Figure S74 - Enlarged ^{1}H NMR stack plot of compound **10** in DMSO-d_6 0.5 % H_2O solution. Samples were prepared in series with an aliquot of the most concentrated solution undergoing serial dilution.

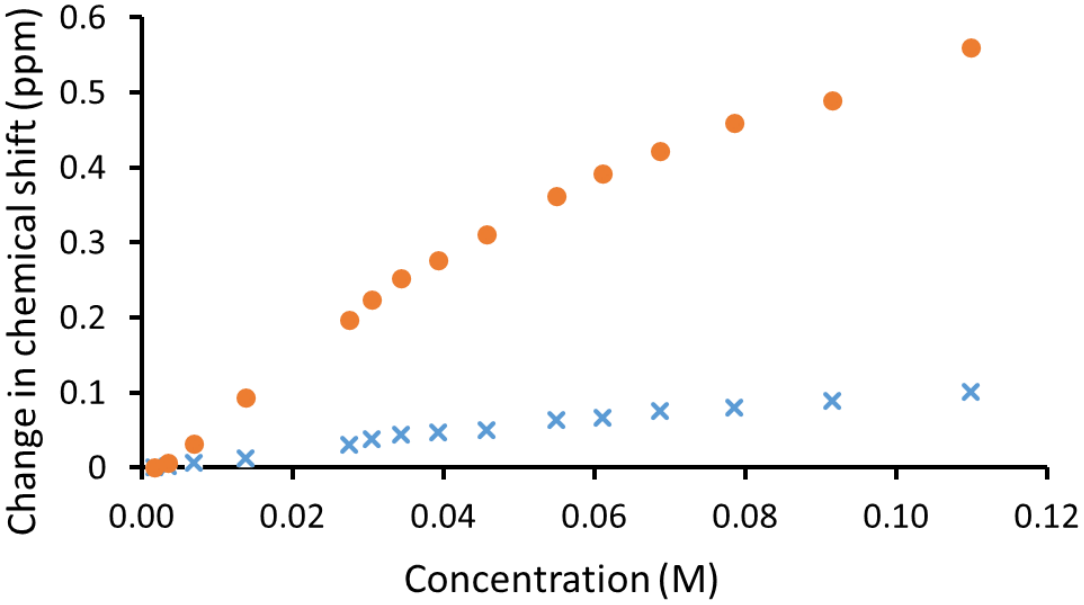

Figure S75 - Graph illustrating the ^{1}H NMR down-field change in chemical shift of urea NH resonances with increasing concentration of compound **10** in DMSO-d_6 0.5 % H_2O (298 K).

Self-association constant calculation

Compound **10** - Dilution study in DMSO-d_6 5 % H_2O. Values calculated from data gathered from both NH 1 and 2.

Equal K/Dimerization model

K_e =5.20 M^{-1} ± 1.162 % K_{dim} = 2.60 M^{-1} ± 0.5811 %

http://app.supramolecular.org/bindfit/view/373ed867-cc92-4837-9a15-dc640801c819

CoEK model

K_e = 13.84 M^{-1} ± 2.2781 % K_{dim} = 6.92 M^{-1} ± 1.1390 %   ρ = 0.47 ± 6.7985 %

http://app.supramolecular.org/bindfit/view/5cb8f87a-4027-4afe-b9d1-fda2a7ccd6b4

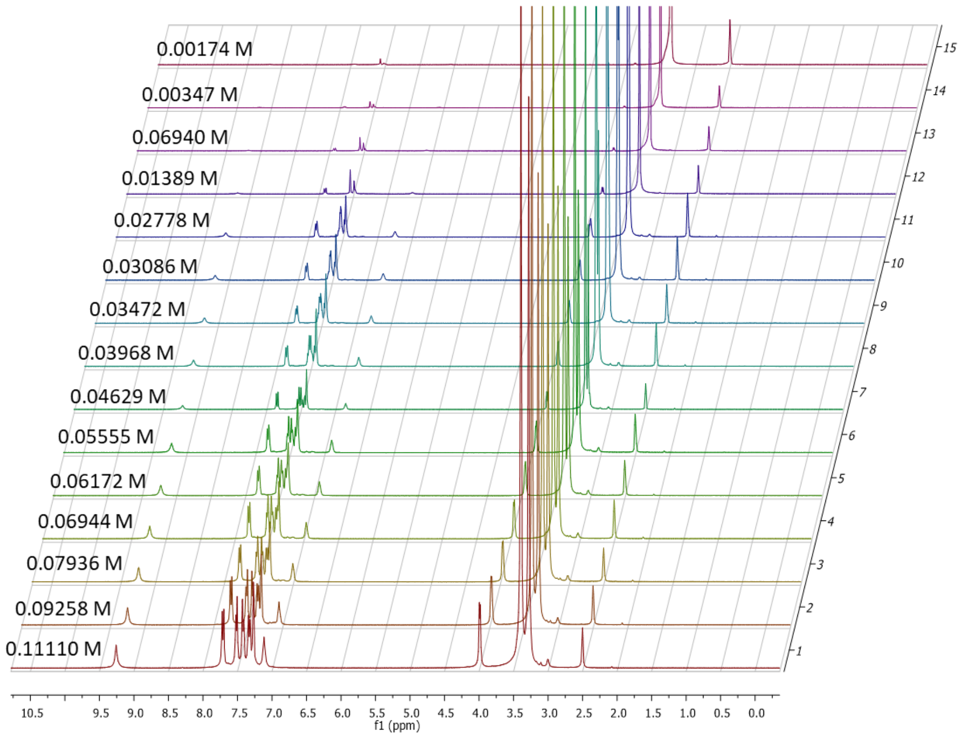

Figure S76 - ^{1}H NMR stack plot of compound **11** in DMSO-d_6 0.5 % H_2O solution. Samples were prepared in series with an aliquot of the most concentrated solution undergoing serial dilution.

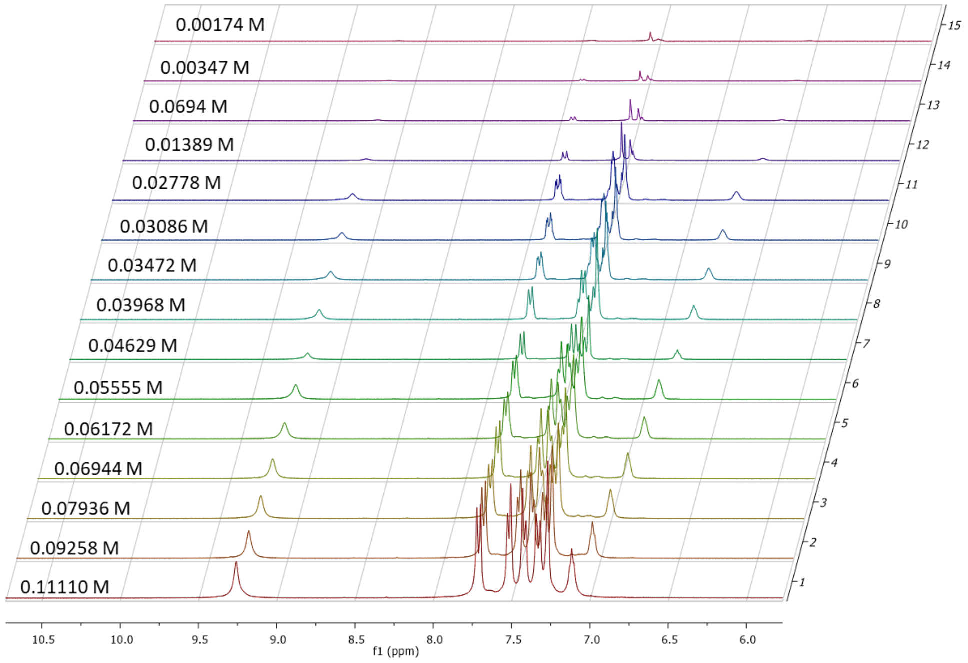

Figure S77 - Enlarged ^{1}H NMR stack plot of compound **11** in DMSO-d_6 0.5 % H_2O solution. Samples were prepared in series with an aliquot of the most concentrated solution undergoing serial dilution.

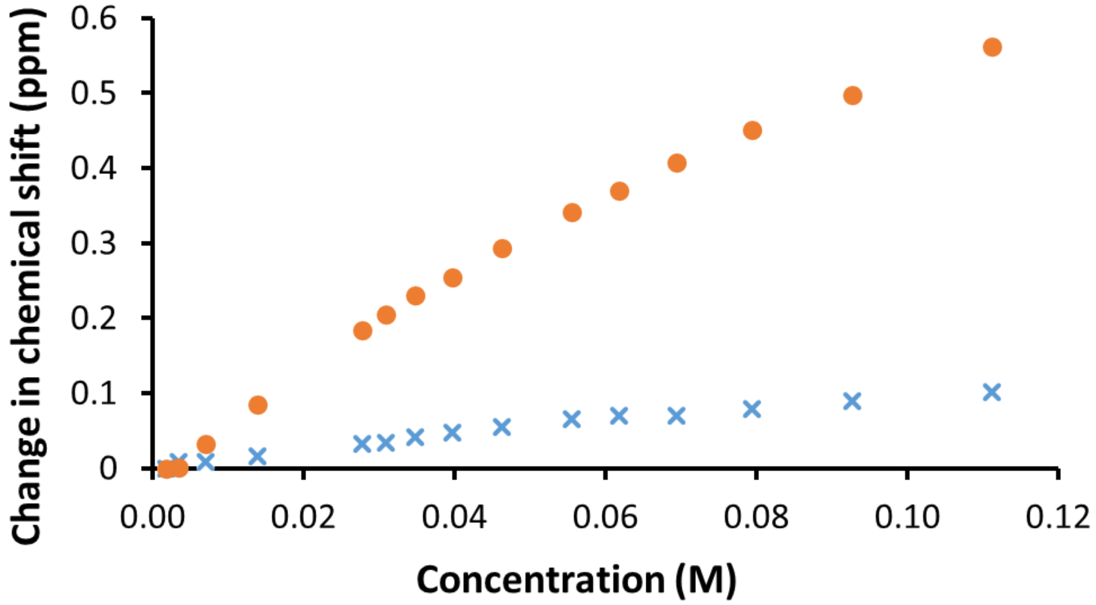

Figure S78 - Graph illustrating the ^{1}H NMR down-field change in chemical shift of urea NH resonances with increasing concentration of compound **11** in DMSO-d_6 0.5 % H_2O (298 K).

Self-association constant calculation

Compound **11** - Dilution study in DMSO-d_6 5 % H_2O. Values calculated from data gathered from both NH 1 and 2.

Equal K/Dimerization model

K_e = 3.50 M^{-1} ± 0.7492 % K_{dim} = 1.75 M^{-1} ± 0.3746 %

http://app.supramolecular.org/bindfit/view/68668b4f-8fb6-4a5f-9ae0-01236d942b96

CoEK model

K_e = 8.62 M^{-1} ± 2.2578 % K_{dim} = 4.31 M^{-1} ± 1.1289 % ρ = 0.55 ± 5.1491 %

http://app.supramolecular.org/bindfit/view/ab1fbab7-3f28-4f14-a7b0-ef20c2b2d02e

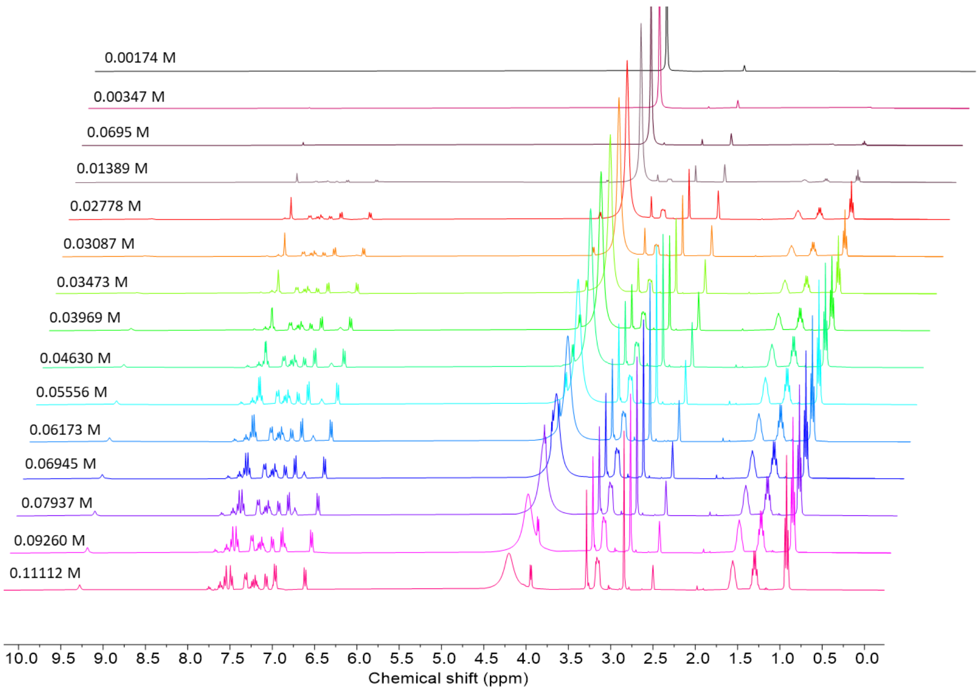

Figure S79 - ^{1}H NMR stack plot of compound co-formulation ***a*** in DMSO-d_6 0.5 % H_2O solution. Samples were prepared in series with an aliquot of the most concentrated solution undergoing serial dilution.

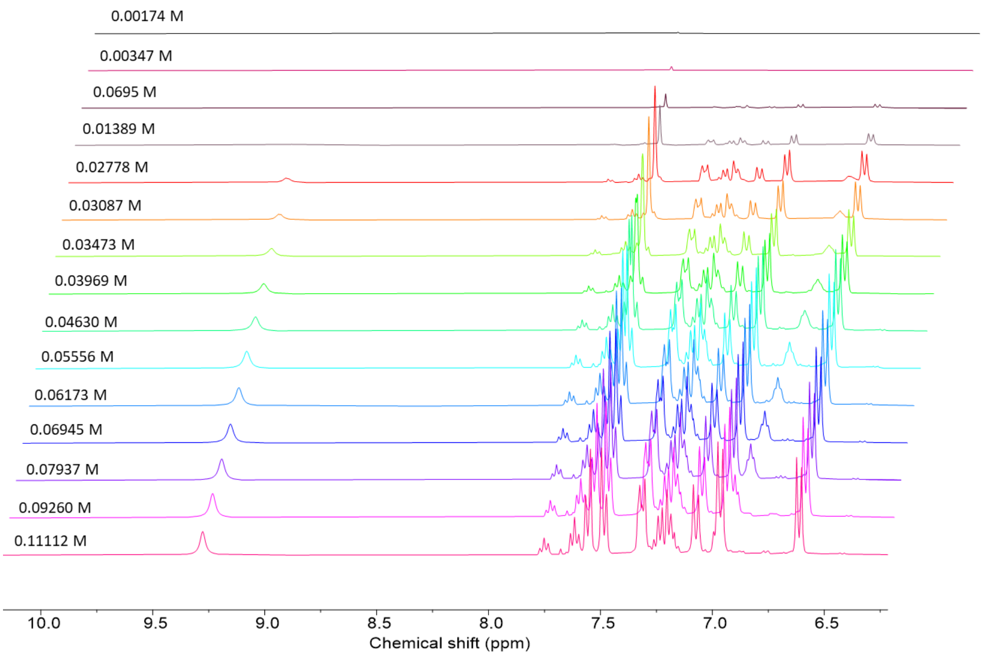

Figure S80 - Enlarged ^{1}H NMR stack plot of co-formulation ***a*** in DMSO-d_6 0.5 % H_2O solution. Samples were prepared in series with an aliquot of the most concentrated solution undergoing serial dilution.

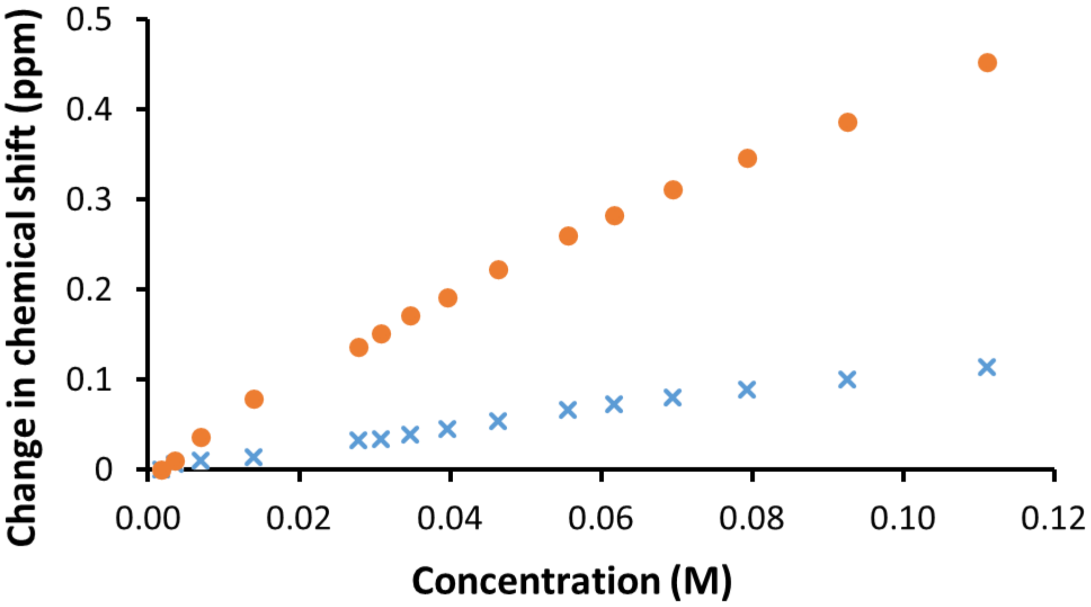

Figure 81 - Graph illustrating the ^{1}H NMR down-field change in chemical shift of urea NH resonances with increasing concentration of co-formulation ***a*** in DMSO-d_6 0.5 % H_2O (298 K).

Self-association constant calculation

Co-formulation *a*- Dilution study in DMSO-d_6 5 % H_2O. Values calculated from data gathered from both NH 1 and 2.

Equal K/Dimerization model

K_e = 1.97 M^{-1} ± 0.8489 % K_{dim} = 0.98 M^{-1} ± 0.4245 %

http://app.supramolecular.org/bindfit/view/b8094449-73ce-4543-a520-b8a9a9ced801

CoEK model

K_e = 0.94 M^{-1} ± 20.1556 % K_{dim} = 0.47 M^{-1} ± 10.0778 % ρ = 1.59 ± 22.7747 %

http://app.supramolecular.org/bindfit/view/b5d85326-de68-4a99-a001-5fe4291f94f0

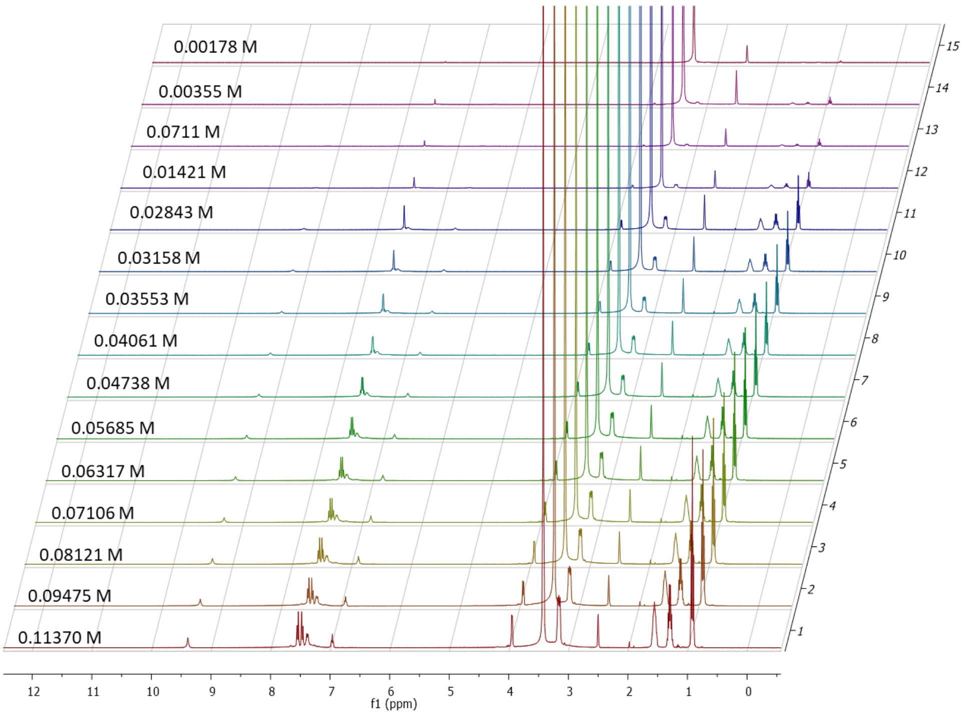

Figure S82 - ^{1}H NMR stack plot of co-formulation ***c*** in DMSO-d_6 0.5 % H_2O solution. Samples were prepared in series with an aliquot of the most concentrated solution undergoing serial dilution.

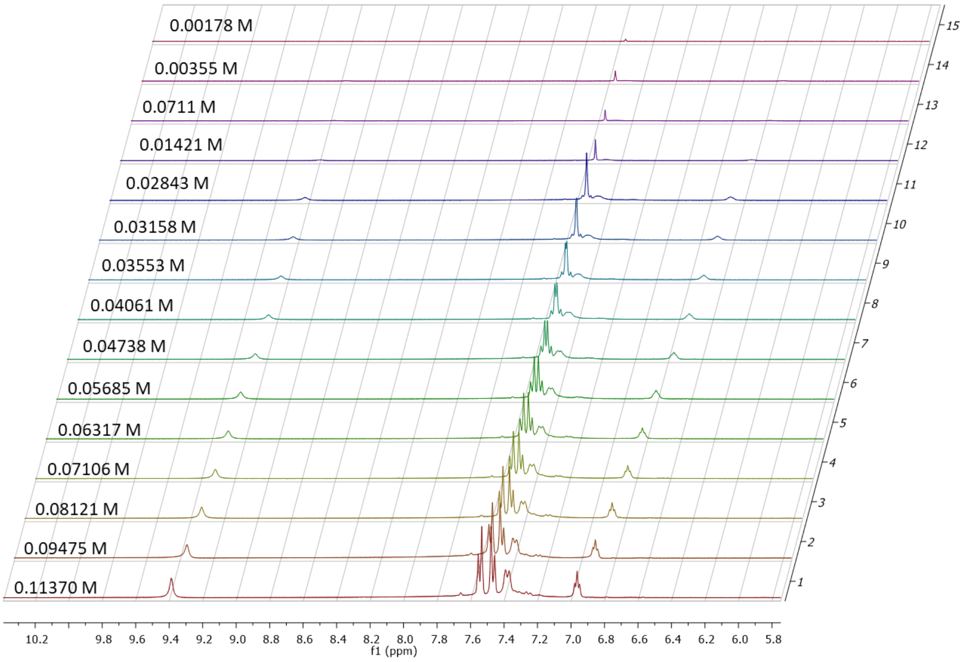

Figure S83 - Enlarged ^{1}H NMR stack plot of co-formulation ***c*** in DMSO-d_6 0.5 % H_2O solution. Samples were prepared in series with an aliquot of the most concentrated solution undergoing serial dilution.

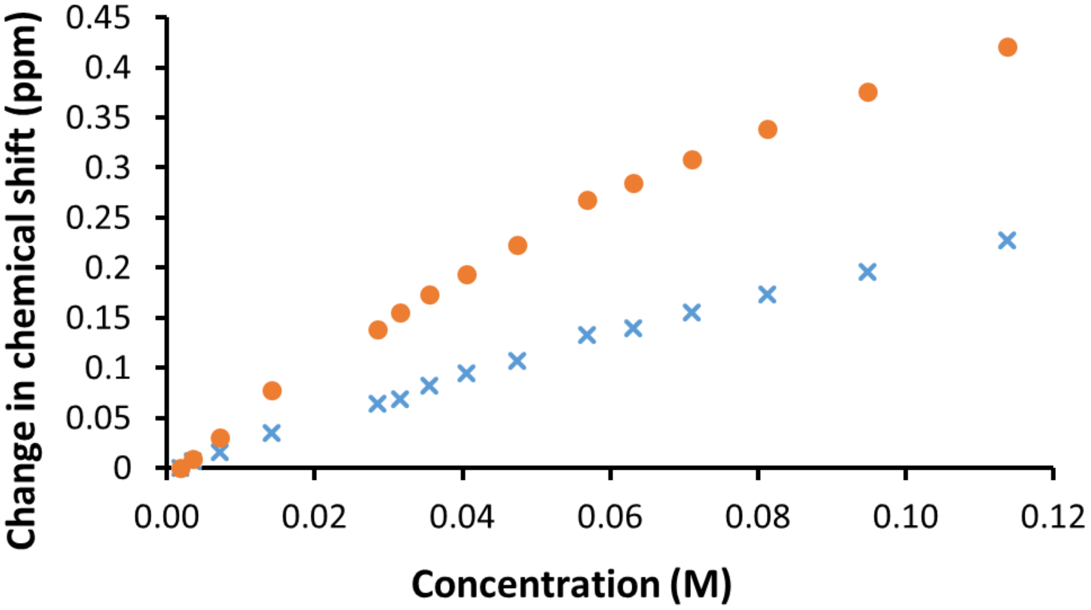

Figure S84 - Graph illustrating the ^{1}H NMR down-field change in chemical shift of urea NH resonances with increasing concentration of co-formulation ***c*** in DMSO-d_6 0.5 % H_2O (298 K).

Self-association constant calculation

Co-formulation ***c*** - Dilution study in DMSO-d_6 5 % H_2O. Values calculated from data gathered from both NH 1 and 2.

Equal K/Dimerization model

K_e = 3.04 M^{-1} ± 1.2523 % K_{dim} = 1.52 M^{-1} ± 0.6262 %

http://app.supramolecular.org/bindfit/view/f816d6d9-662c-4dbb-8cdc-b1383f588896

CoEK model

K_e = 8.26 M^{-1} ± 4.0444 % K_{dim} = 4.13 M^{-1} ± 2.0222 % ρ = 0.53 ± 9.2207 %

http://app.supramolecular.org/bindfit/view/7f5bdaf8-208a-4fc1-8b5f-40aae47b15c5

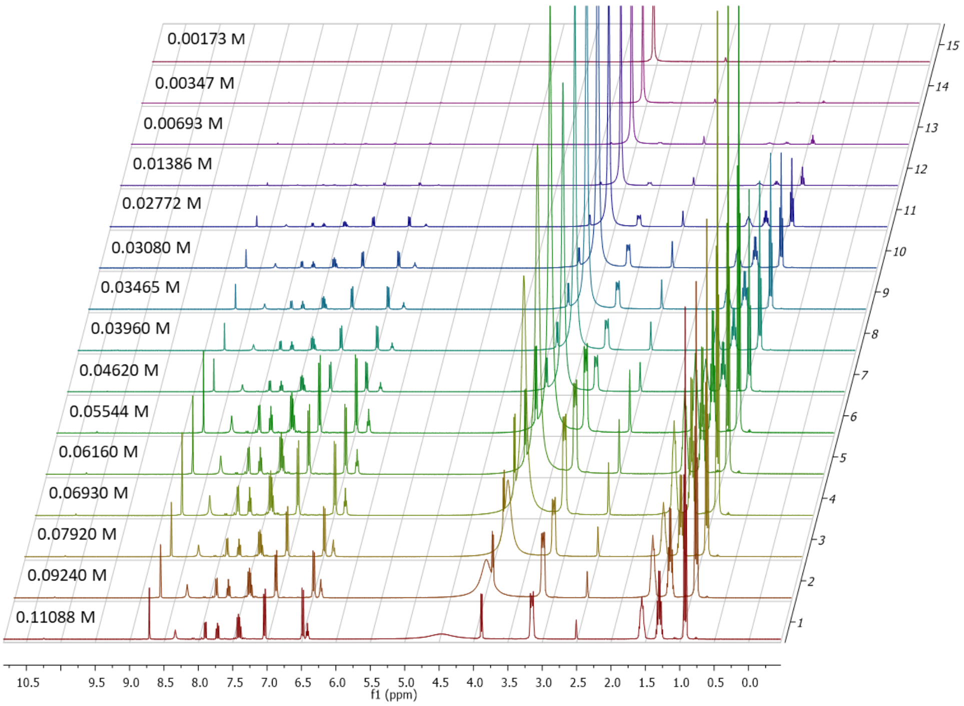

Figure S85 - ^{1}H NMR stack plot of co-formulation ***e*** in DMSO-d_6 0.5 % H_2O solution. Samples were prepared in series with an aliquot of the most concentrated solution undergoing serial dilution.

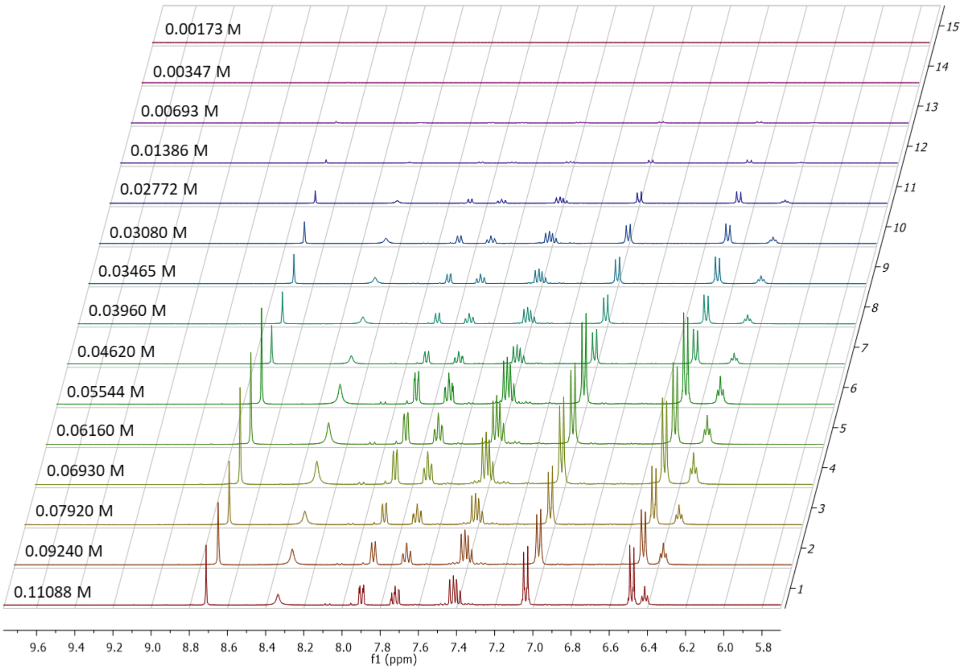

Figure S86 - Enlarged ^{1}H NMR stack plot of co-formulation ***e*** in DMSO-d_6 0.5 % H_2O solution. Samples were prepared in series with an aliquot of the most concentrated solution undergoing serial dilution.

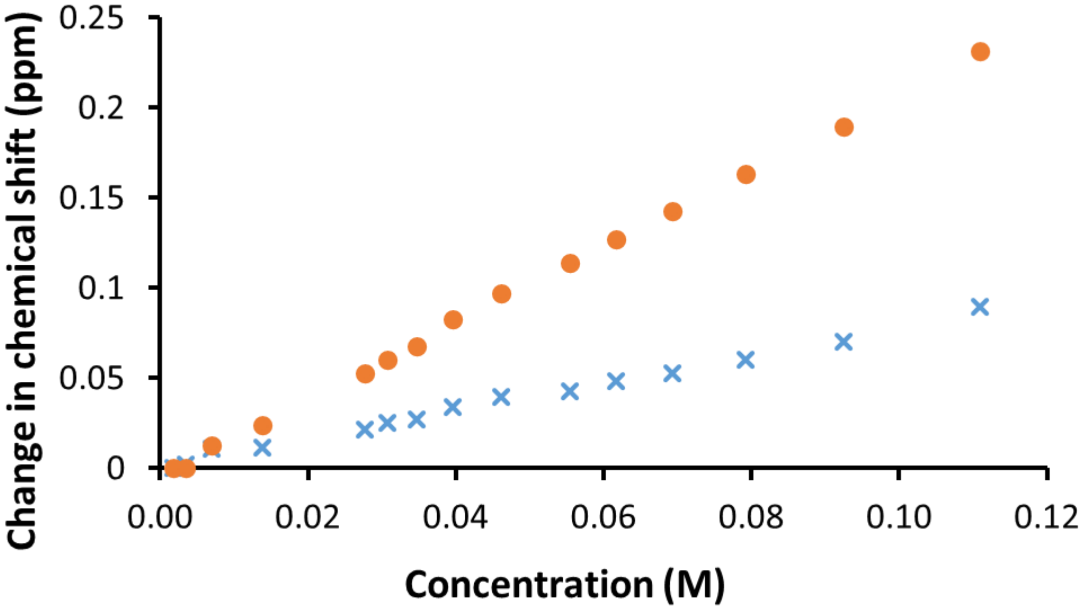

Figure S87 - Graph illustrating the ^{1}H NMR down-field change in chemical shift of urea NH resonances with increasing concentration of co-formulation ***e*** in DMSO-d_6 0.5 % H_2O (298 K).

Self-association constant calculation

Co-formulation ***e*** - Dilution study in DMSO-d_6 5 % H_2O. Values calculated from data gathered from both NH 1 and 2.

Equal K/Dimerization model

K_e = 0.444 M^{-1} ± 0.9113 % K_{dim} = 0.222 M^{-1} ± 0.4556 %

http://app.supramolecular.org/bindfit/view/68a49cda-b221-49c3-ac43-39fa882cba1d

CoEK model

K_e = 0.222 M^{-1} ± 826.6208 % K_{dim} = 0.111 M^{-1} ± 413.3104 % ρ = 1.50 ± 829.5311 %

http://app.supramolecular.org/bindfit/view/8154a4b6-25e5-421e-88ed-23281f88ff10

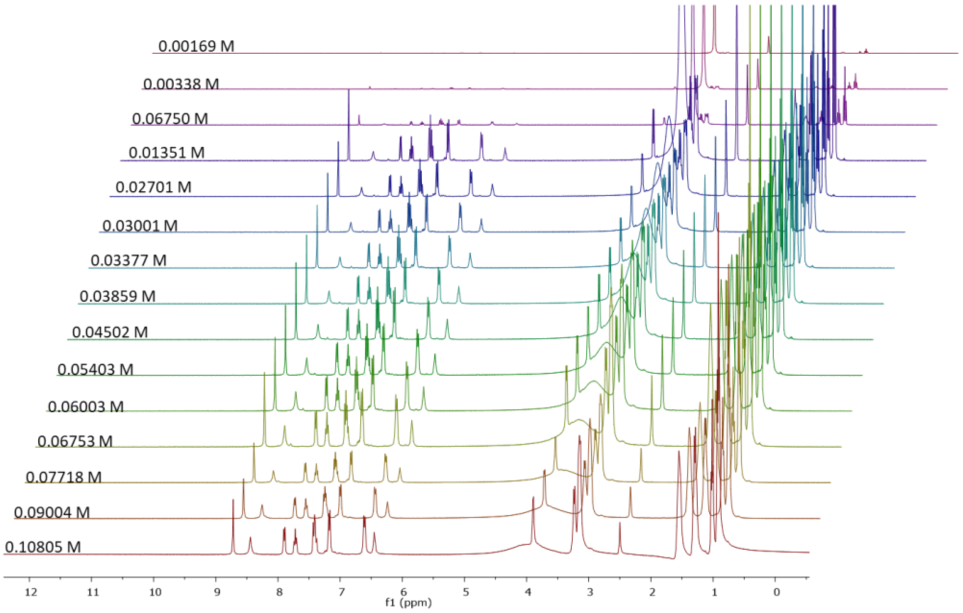

Figure S88 - H NMR stack plot of co-formulation ***f*** in DMSO-d_6 0.5 % H_2O solution. Samples were prepared in series with an aliquot of the most concentrated solution undergoing serial dilution.

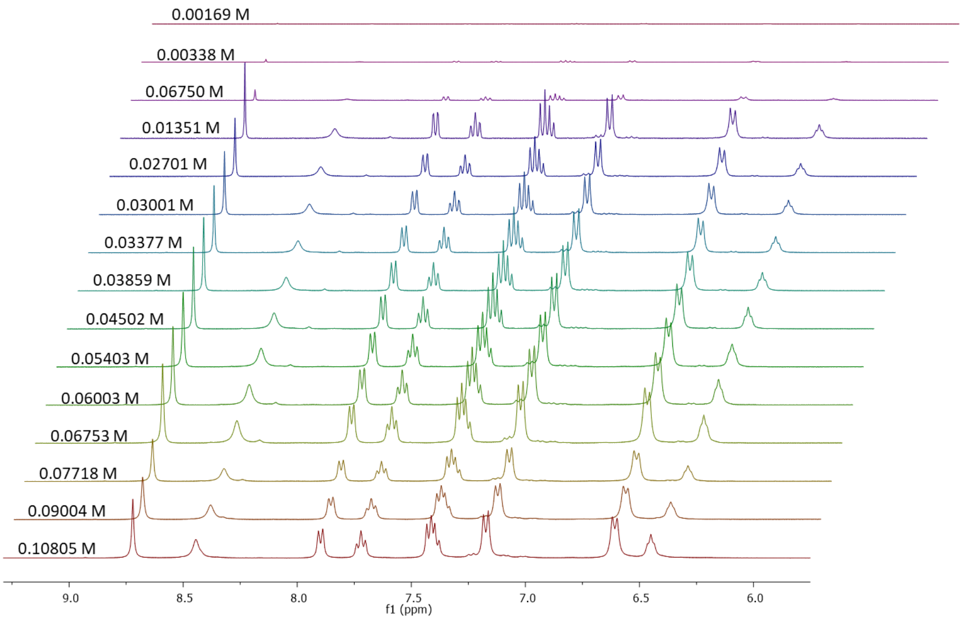

Figure S89 - Enlarged ^{1}H NMR stack plot co-formulation ***f*** in DMSO-d_6 0.5 % H_2O solution. Samples were prepared in series with an aliquot of the most concentrated solution undergoing serial dilution.

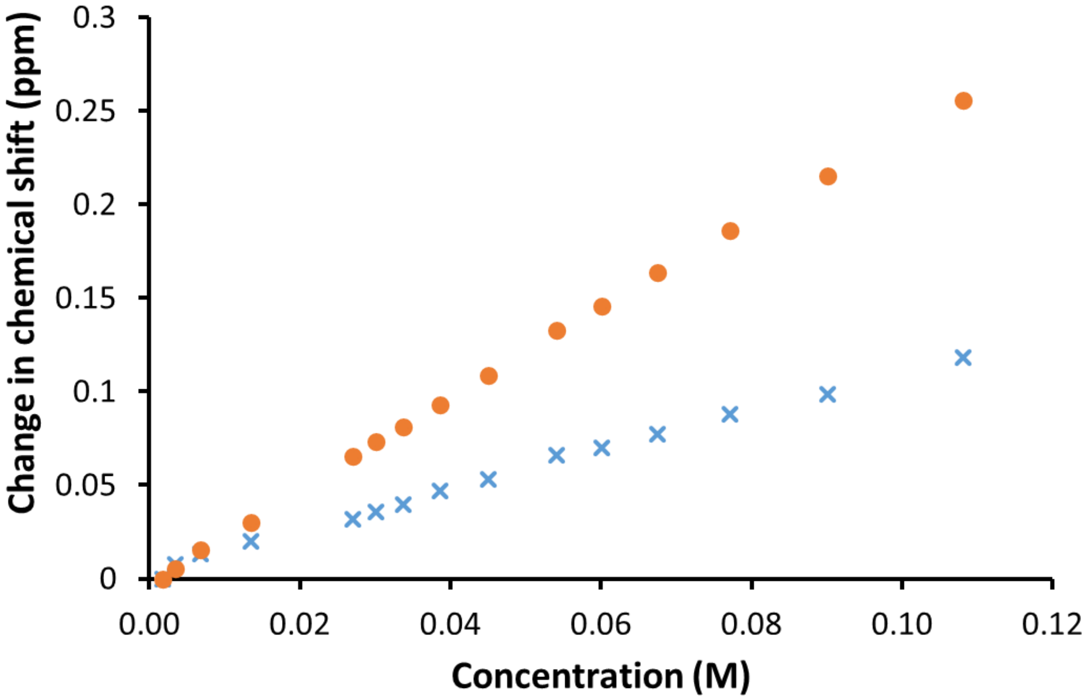

Figure S90 - Graph illustrating the ^{1}H NMR down-field change in chemical shift of urea NH resonances with increasing concentration of co-formulation ***f*** in DMSO-d_6 0.5 % H_2O (298 K).

Self-association constant calculation

<u>Co-formulation *f* - Dilution study in DMSO-d_6 5 % H_2O. Values calculated from data gathered from both NH 1 and 2.</u>

Equal K/Dimerization model

K_e =0.43 M^{-1} ± 0.6069 % K_{dim} = 0.22 M^{-1} ± 0.3035 %

http://app.supramolecular.org/bindfit/view/e911fde3-e4e5-4fd2-acce-633082bba6a8

CoEK model

K_e = 1.85 M^{-1} ± 7.5280 % K_{dim} = 0.93 M^{-1} ± 3.7640 % ρ = 0.56 ± 9.6842 %

http://app.supramolecular.org/bindfit/view/119798be-4dc0-407a-9da6-db8874993cd1

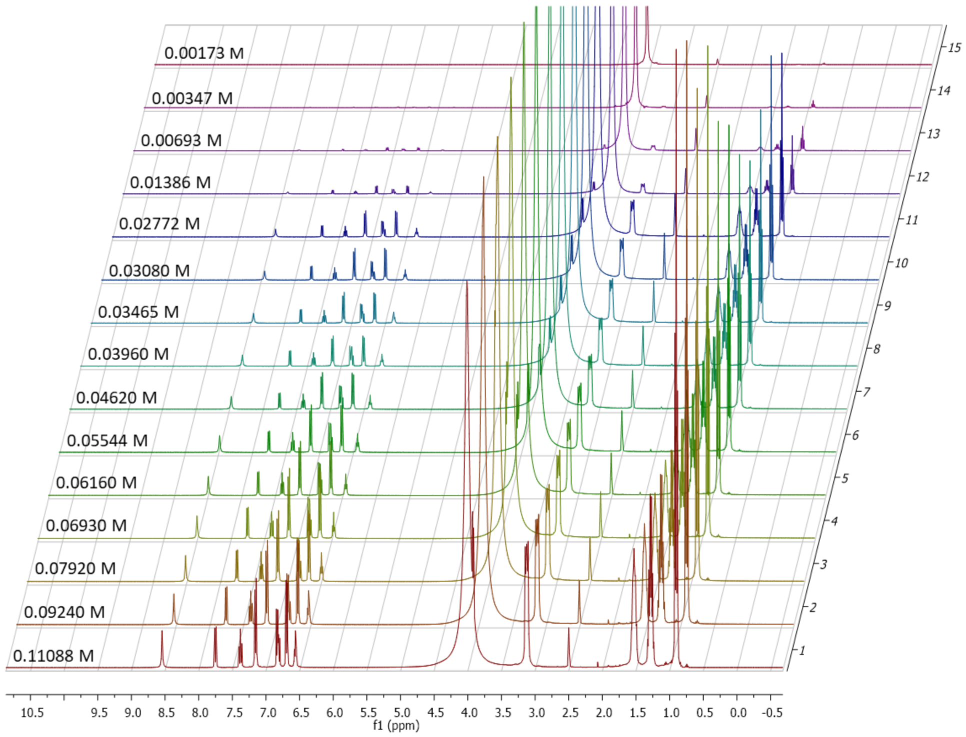

Figure S91 - ${}^{1}H$ NMR stack plot of co-formulation ***g*** in DMSO-d_6 0.5 % H_2O solution. Samples were prepared in series with an aliquot of the most concentrated solution undergoing serial dilution.

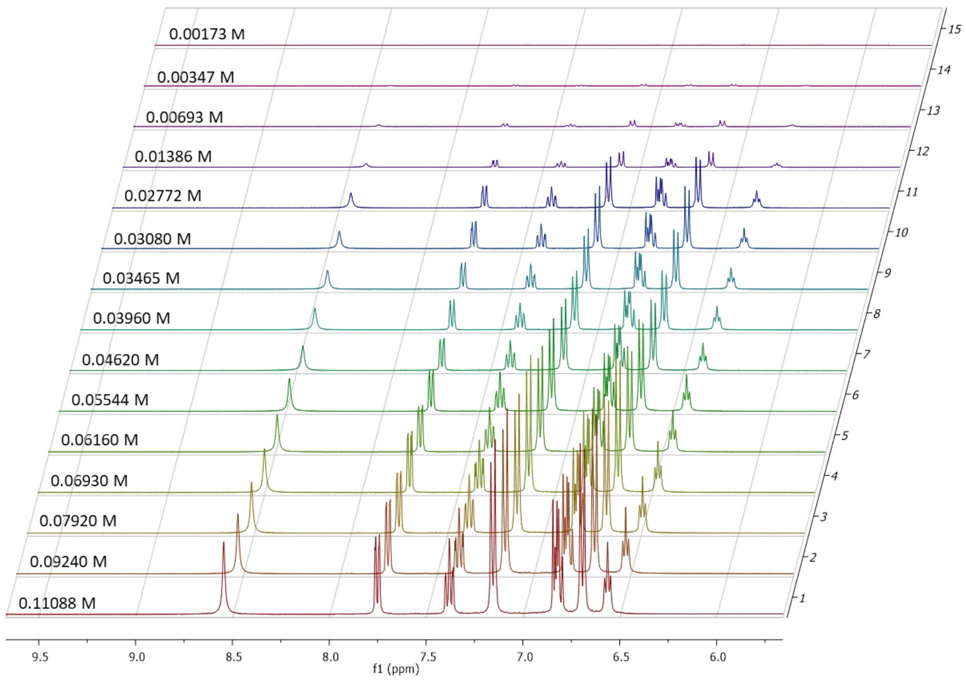

Figure S92 - Enlarged ${}^{1}H$ NMR stack plot of co-formulation ***g*** in DMSO-d_6 0.5 % H_2O solution. Samples were prepared in series with an aliquot of the most concentrated solution undergoing serial dilution.

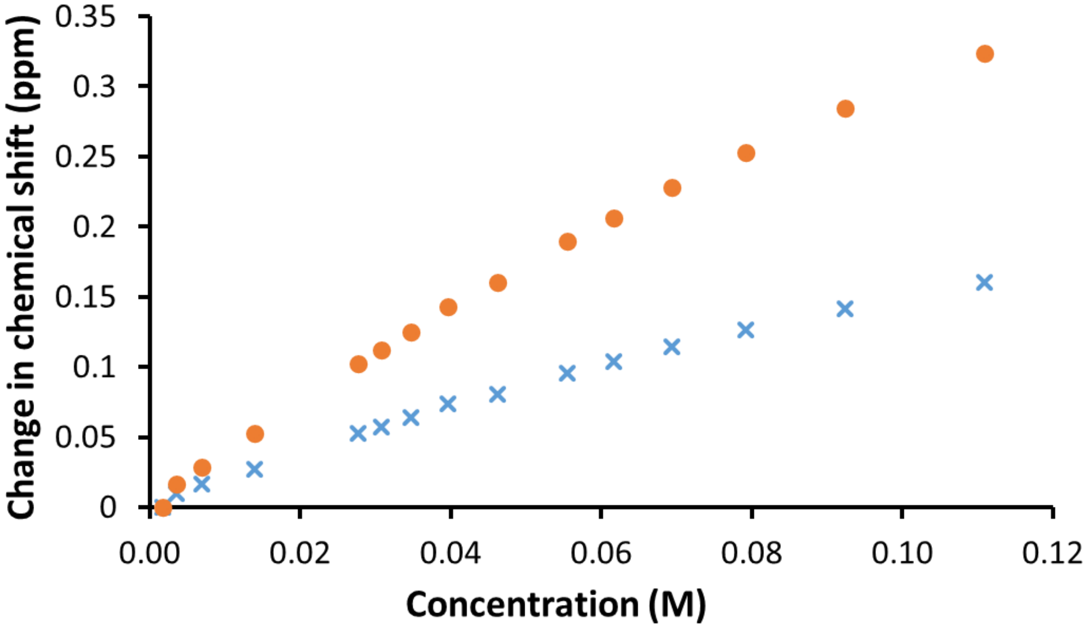

Figure S93 - Graph illustrating the ^{1}H NMR down-field change in chemical shift of urea NH resonances with increasing concentration of co-formulation ***g*** in DMSO-d_6 0.5 % H_2O (298 K).

Self-association constant calculation

Co-formulation ***g*** - Dilution study in DMSO-d_6 5 % H_2O. Values calculated from data gathered from both NH 1 and 2.

Equal K/Dimerization model

K_e 2.20 M^{-1} ± 0.6789 % K_{dim} = 1.10 M^{-1} ± 0.3395 %

http://app.supramolecular.org/bindfit/view/c5e25175-ab3f-4e47-9d87-efee681d5a98

CoEK model

K_e = 3.99 M^{-1} ± 4.2736 % K_{dim} = 2.00 M^{-1} ± 2.1368 %   ρ = 0.72 ± 6.7428 %

http://app.supramolecular.org/bindfit/view/8bece7ae-ac1a-475d-a8cd-57ae7ea0f045

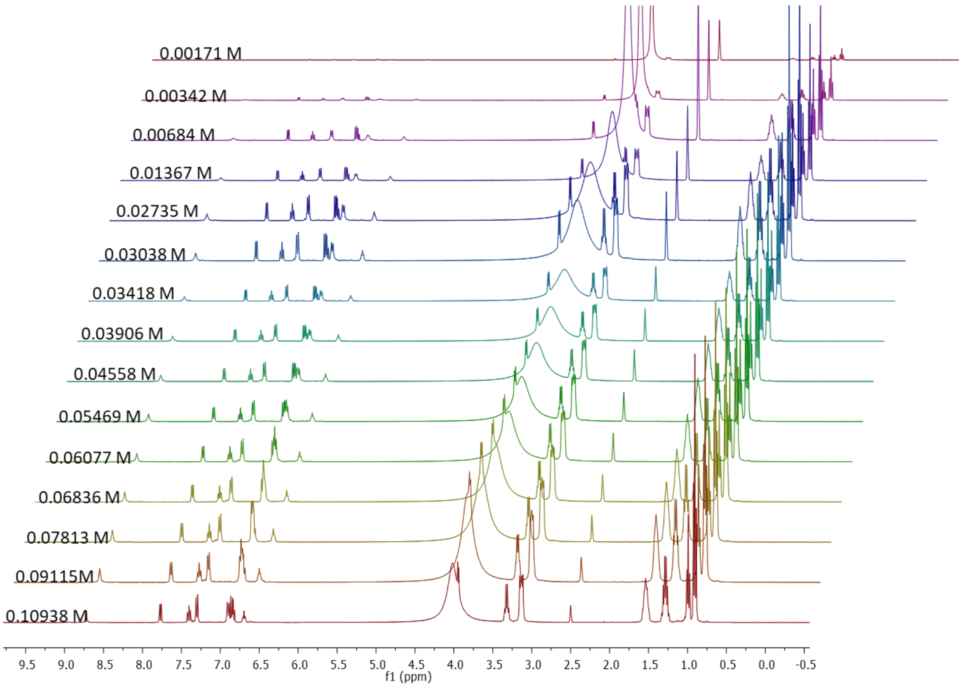

Figure S94 - ^{1}H NMR stack plot of co-formulation ***h*** in DMSO-d_6 0.5 % H_2O solution. Samples were prepared in series with an aliquot of the most concentrated solution undergoing serial dilution.

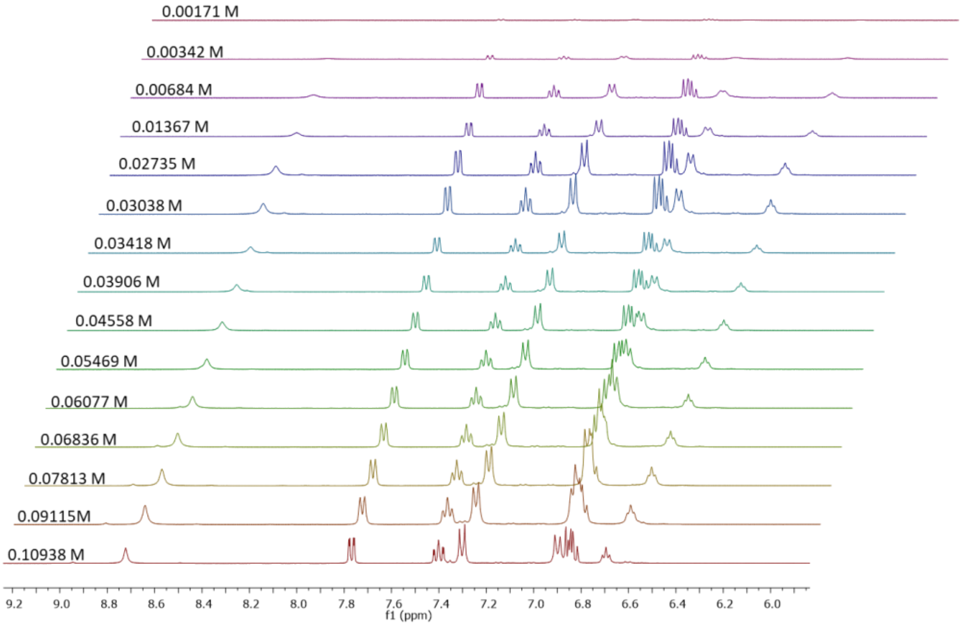

Figure S95 - Enlarged ^{1}H NMR stack plot of co-formulation ***h*** in DMSO-d_6 0.5 % H_2O solution. Samples were prepared in series with an aliquot of the most concentrated solution undergoing serial dilution.

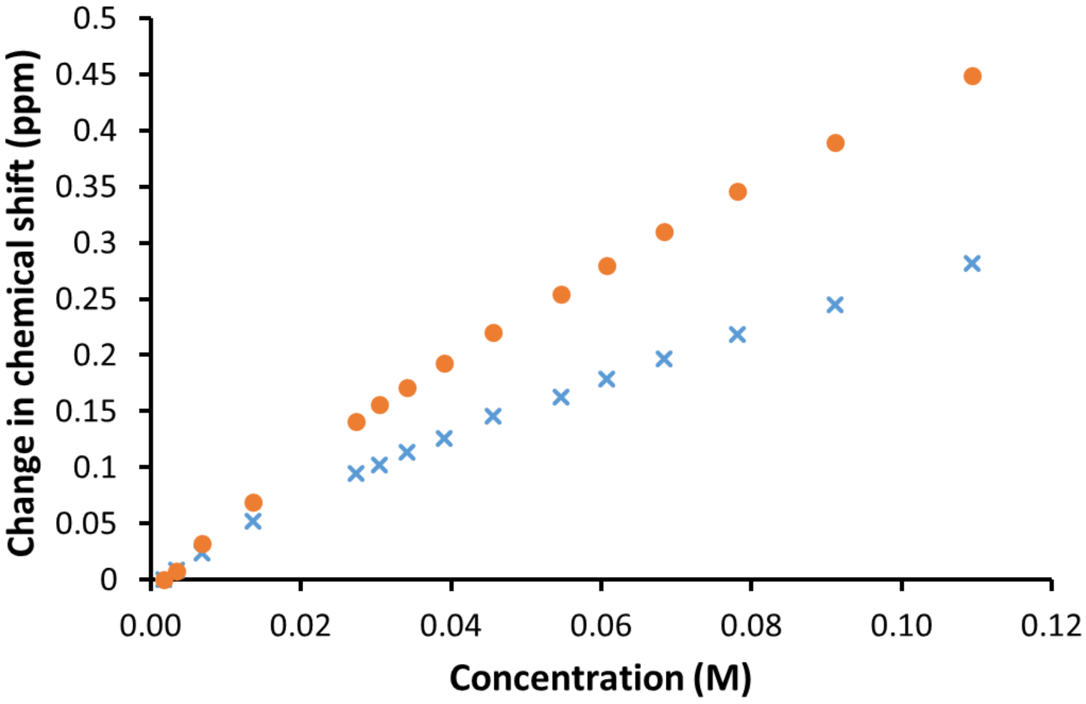

Figure S96 - Graph illustrating the ^{1}H NMR down-field change in chemical shift of urea NH resonances with increasing concentration of co-formulation ***h*** in DMSO-d_6 0.5 % H_2O (298 K).

Self-association constant calculation

Co-formulation ***h*** - Dilution study in DMSO-d_6 5 % H_2O. Values calculated from data gathered from both NH 1 and 2.

Equal K/Dimerization model

K_e = 2.40 M^{-1} ± 0.6268 % K_{dim} = 1.20 M^{-1} ± 0.33134 %

http://app.supramolecular.org/bindfit/view/d7621321-477d-4db3-8e31-65ee1093522b

CoEK model

K_e = 1.14 M^{-1} ± 12.4906 % K_{dim} = 0.57 M^{-1} ± 6.2453 %   ρ = 1.61 ± 14.3851 %

http://app.supramolecular.org/bindfit/view/a07bd758-6062-48c0-8e56-5217b2c1cd17

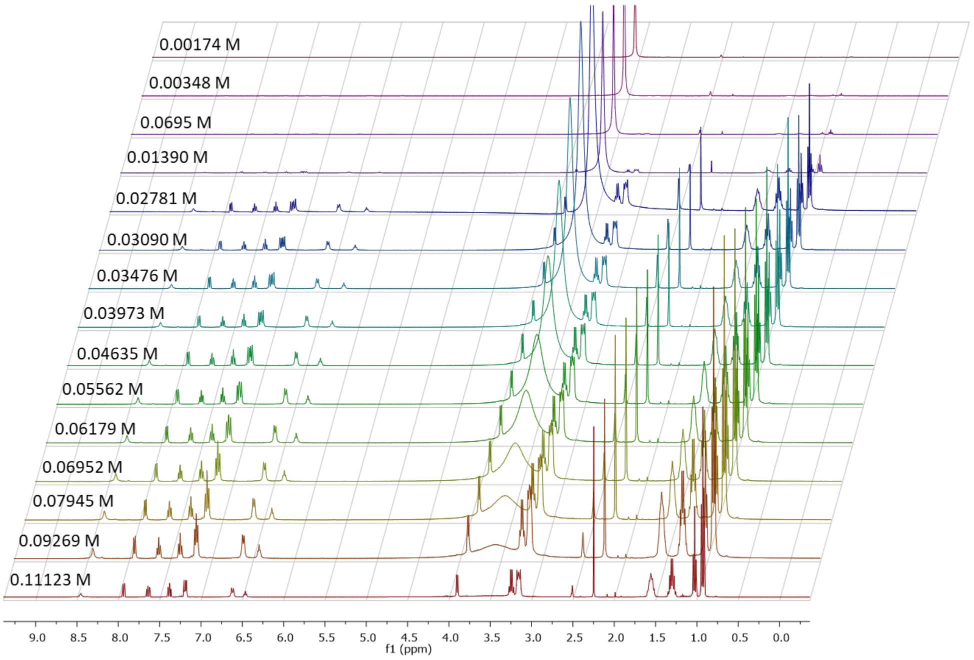

Figure S97 - ^{1}H NMR stack plot of co-formulation ***j*** in DMSO-d_6 0.5 % H_2O solution. Samples were prepared in series with an aliquot of the most concentrated solution undergoing serial dilution.

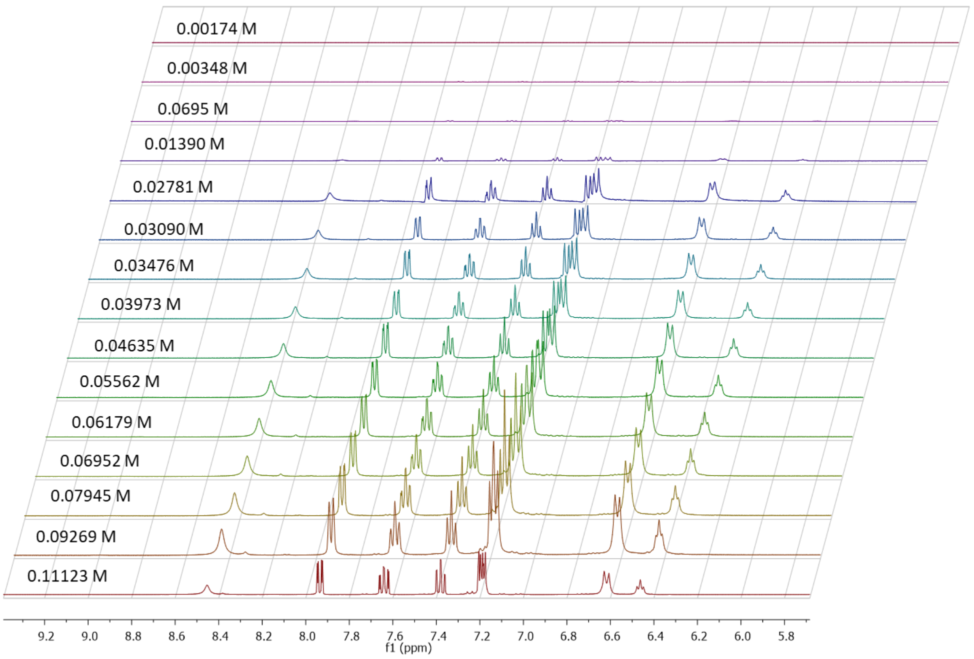

Figure S98 - Enlarged ^{1}H NMR stack plot of co-formulation ***j*** in DMSO-d_6 0.5 % H_2O solution. Samples were prepared in series with an aliquot of the most concentrated solution undergoing serial dilution.

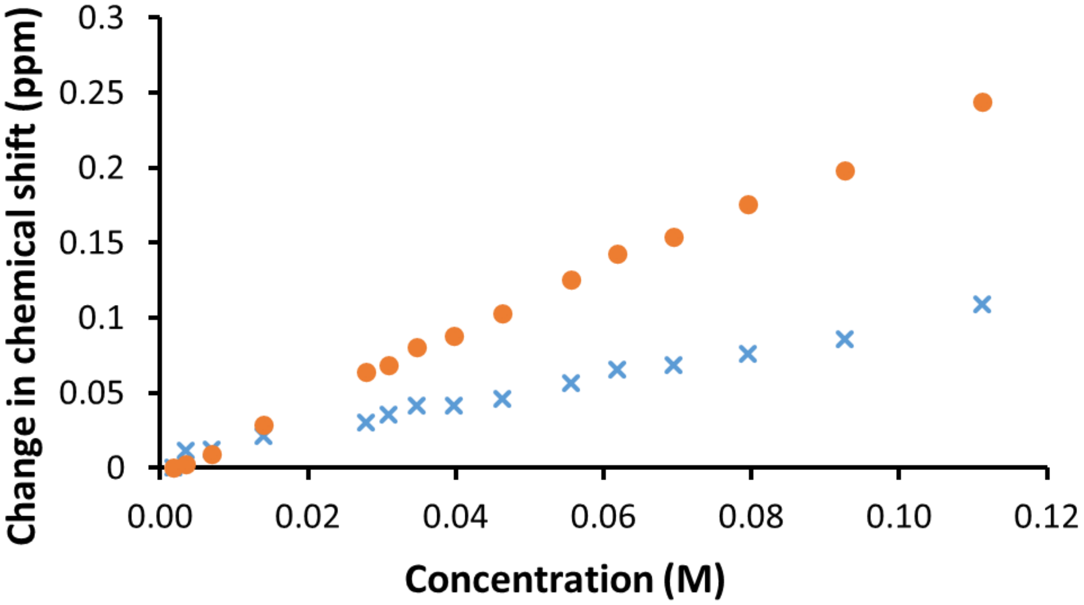

Figure S99 - Graph illustrating the ^{1}H NMR down-field change in chemical shift of urea NH resonances with increasing concentration of co-formulation ***j*** in DMSO-d_6 0.5 % H_2O (298 K).

Self-association constant calculation

Co-formulation ***j*** - Dilution study in DMSO-d_6 5 % H_2O. Values calculated from data gathered from both NH 1 and 2.

Equal K/Dimerization model

K_e = 0.58 M^{-1} ± 1.2668 % K_{dim} = 0.29 M^{-1} ± 0.6334 %

http://app.supramolecular.org/bindfit/view/e20bb02e-5f74-4bf4-bc37-b8efd7ecc28b

CoEK model

K_e = 0.28 M^{-1} ± 92.4876 % K_{dim} = 0.14 M^{-1} ± 46.4816 % ρ = 1.53 ± 96.4816 %

http://app.supramolecular.org/bindfit/view/6c85fddc-52a2-4344-b151-7c75dddb6eba

Timed ^{1}H NMR

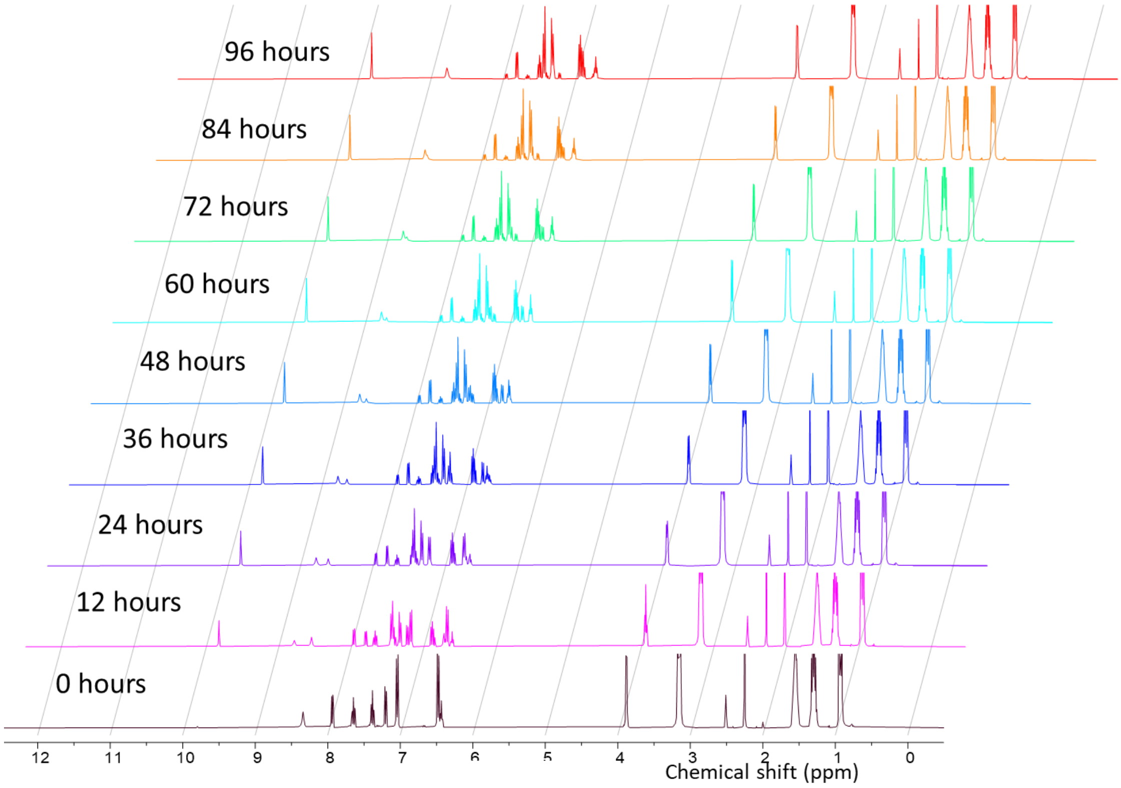

Figure S100 – Timed ^{1}H NMR stack plot of co-formulation ***i*** in DMSO-d_6 at 298 K.

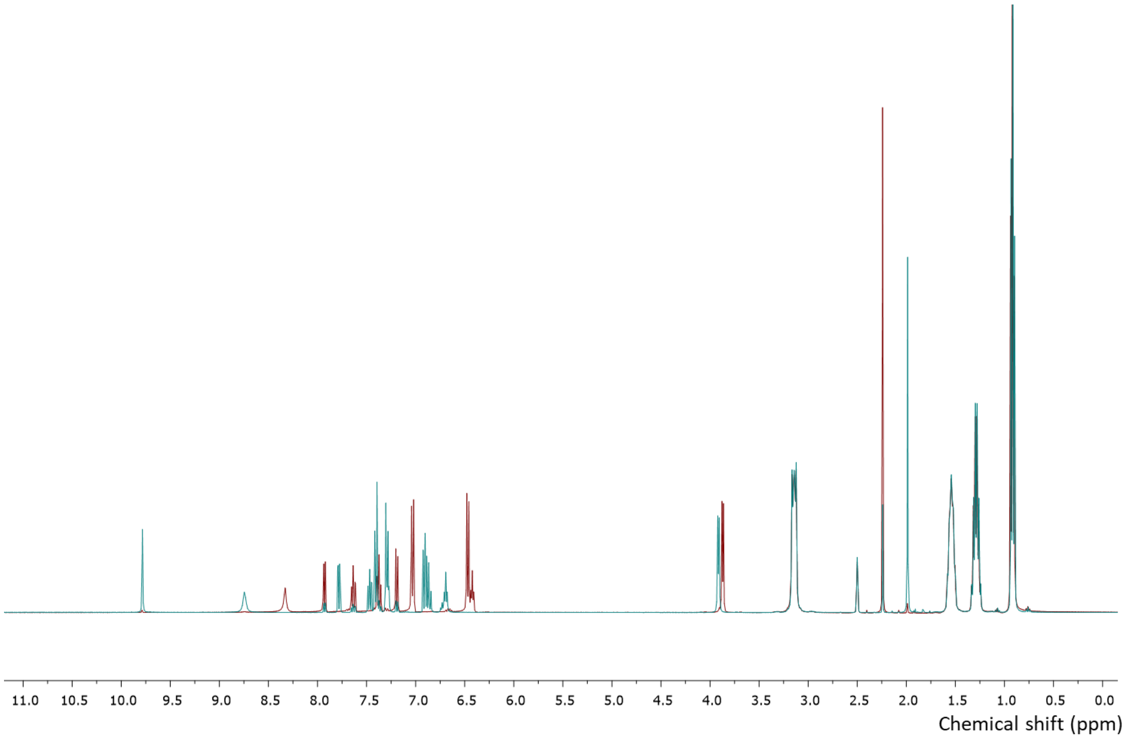

Figure S101 – Timed ^{1}H NMR of co-formulation ***i*** in DMSO-d_6 conducted at 298 K at 0 and 96 hours.

Dynamic Light Scattering data

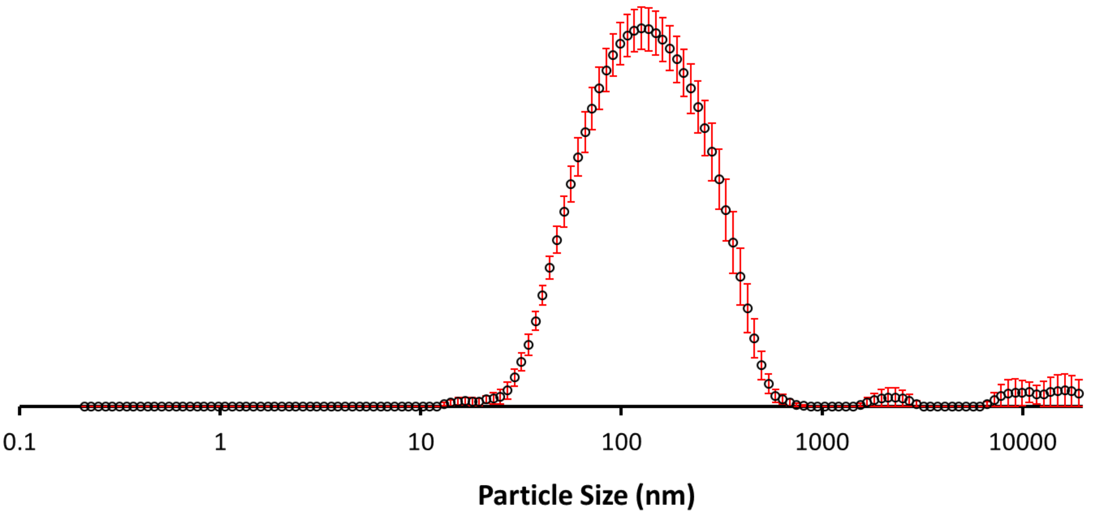

Figure S102 - The average intensity particle size distribution calculated using 9 DLS runs for compound **3** (5.56 mM) in an EtOH:H_2O (1:19) solution at 298 K.

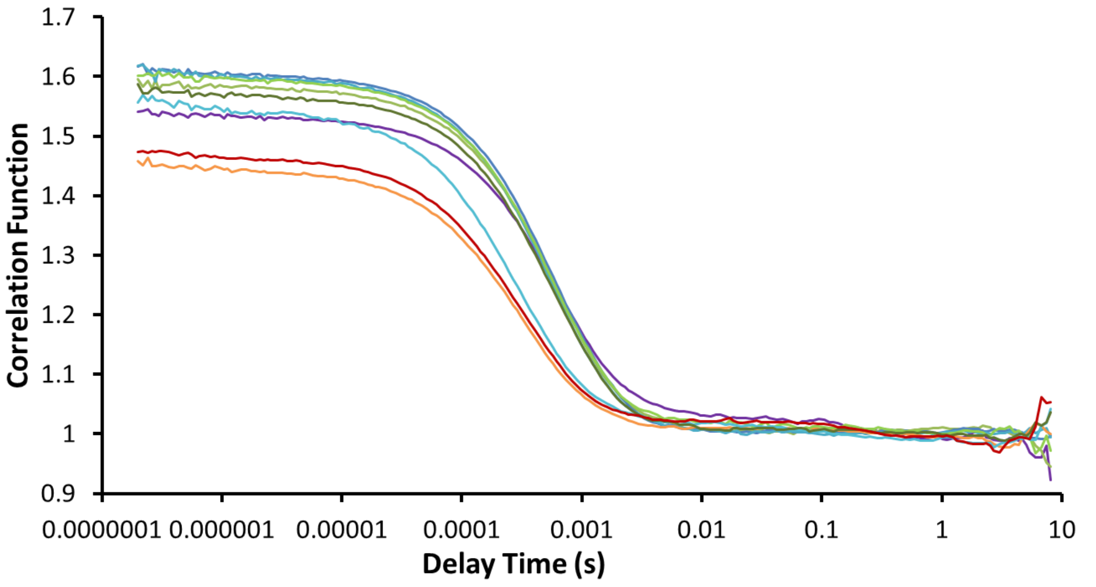

Figure S103 - Correlation function data for 9 DLS runs of compound **3** (5.56 mM) in an EtOH:H_2O (1:19) solution at 298 K.

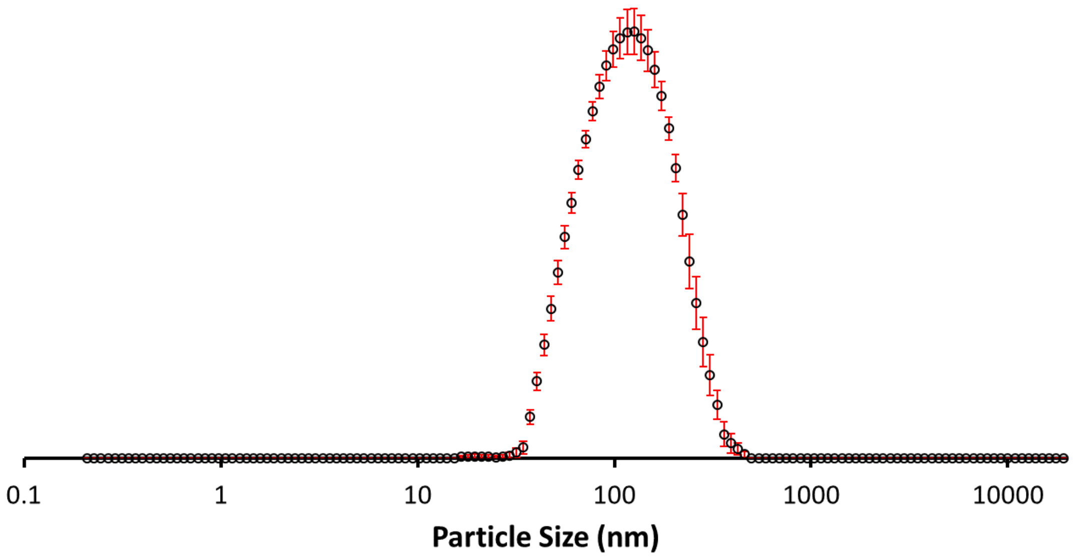

Figure S104 - The average intensity particle size distribution calculated using 10 DLS runs for compound **3** (0.56 mM) in an EtOH:H_2O (1:19) solution at 298 K.

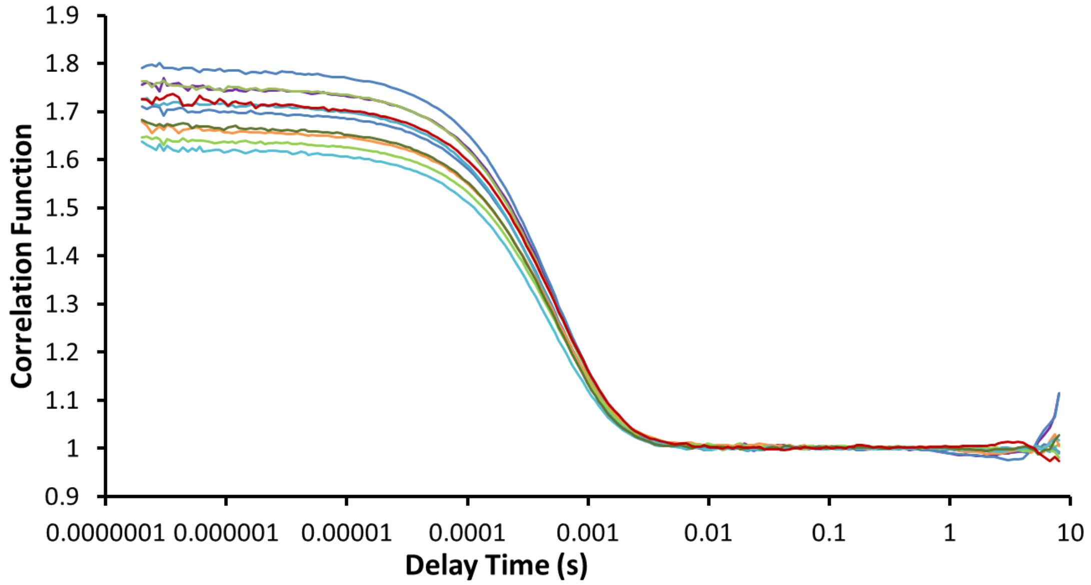

Figure S105 - Correlation function data for 10 DLS runs of compound **3** (0.56 mM) in an EtOH:H_2O (1:19) solution at 298 K.

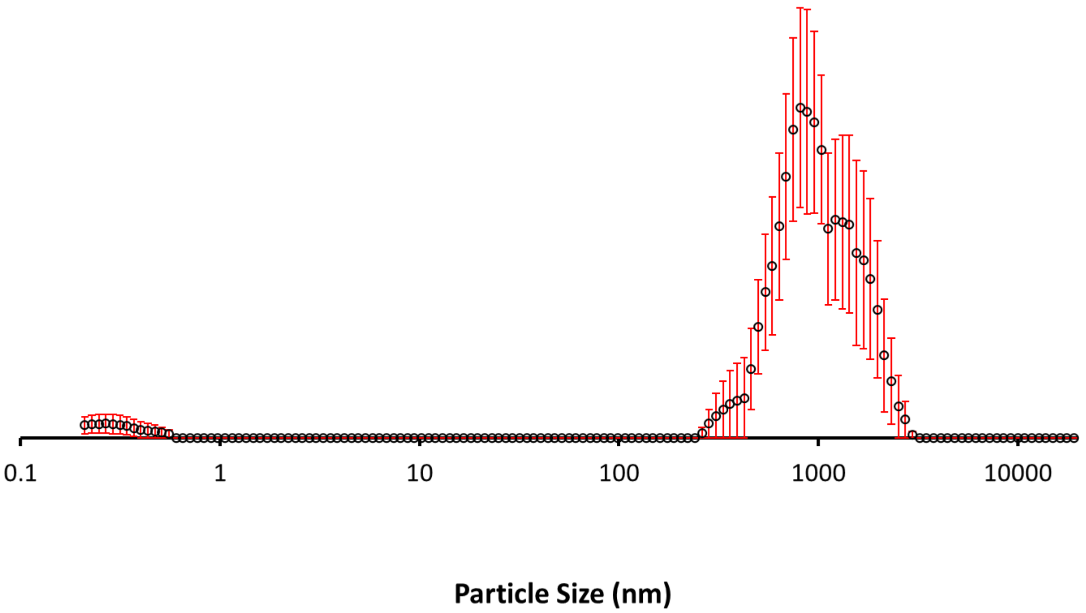

Figure S106 - The average intensity particle size distribution calculated using 10 DLS runs for compound **10** (5.56 mM) in an EtOH:H_2O (1:19) solution at 298 K.

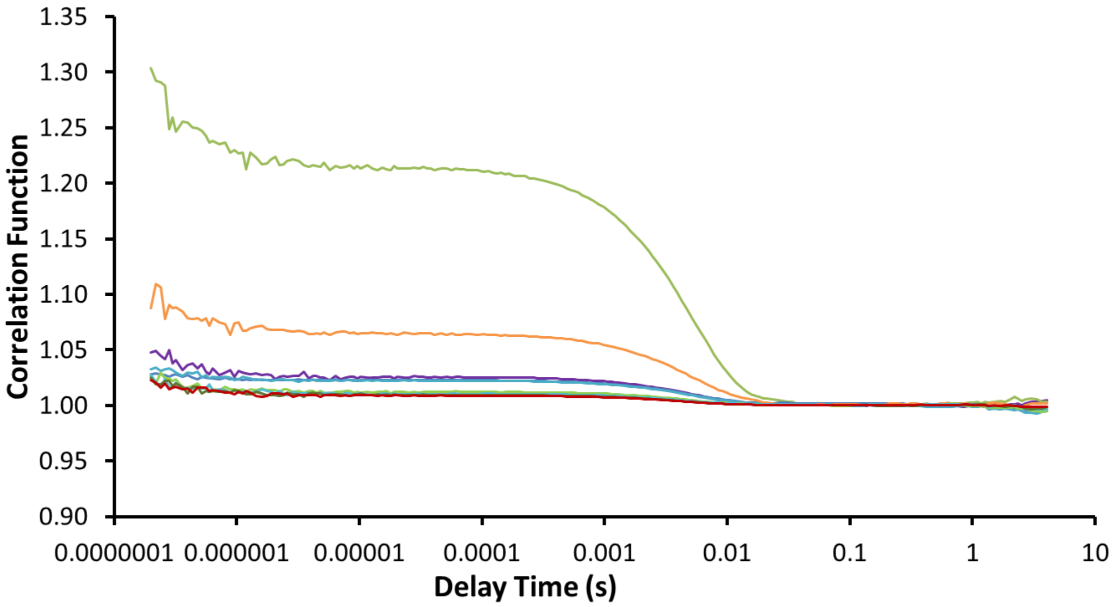

Figure S107 - Correlation function data for 10 DLS runs of compound **10** (5.56 mM) in an EtOH:H_2O (1:19) solution at 298 K.

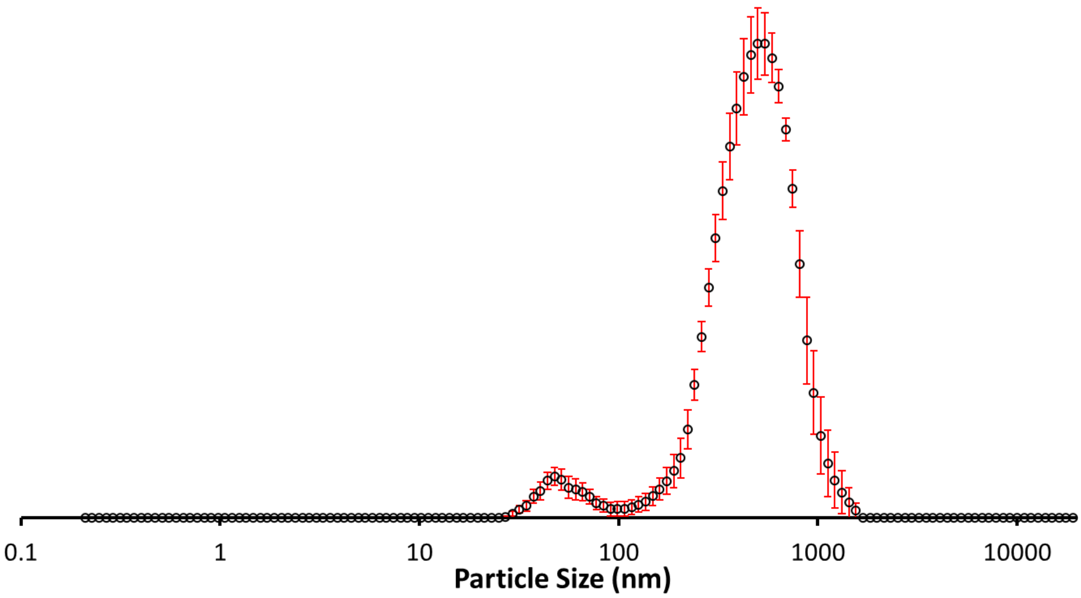

Figure S108 - The average intensity particle size distribution calculated using 10 DLS runs for compound **10** (0.56 mM) in an EtOH:H_2O (1:19) solution at 298 K.

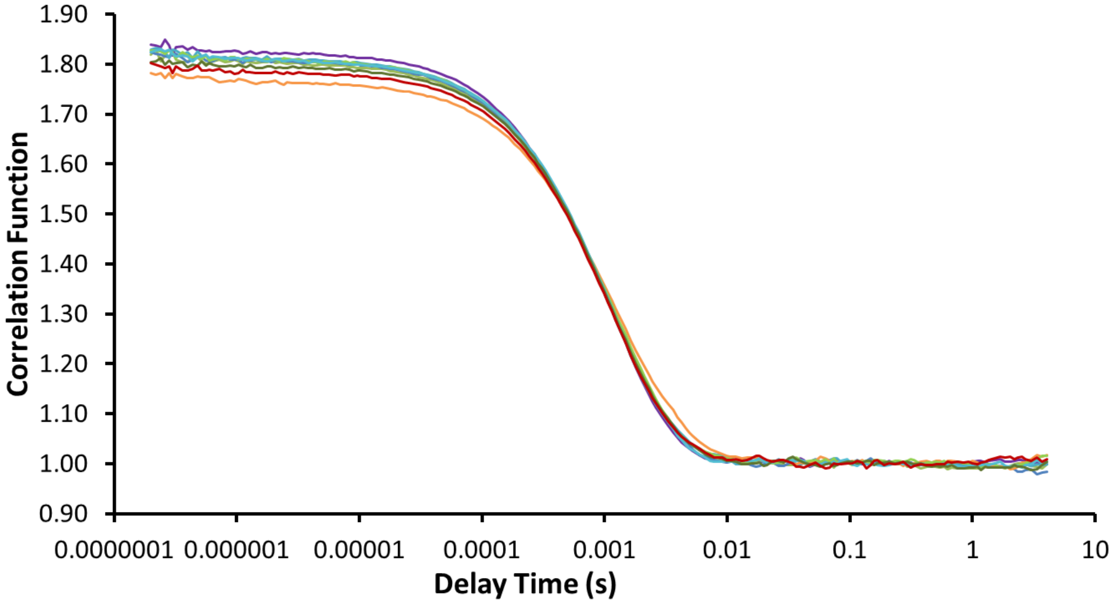

Figure S109 - Correlation function data for 10 DLS runs of compound **10** (0.56 mM) in an EtOH:H_2O (1:19) solution at 298 K.

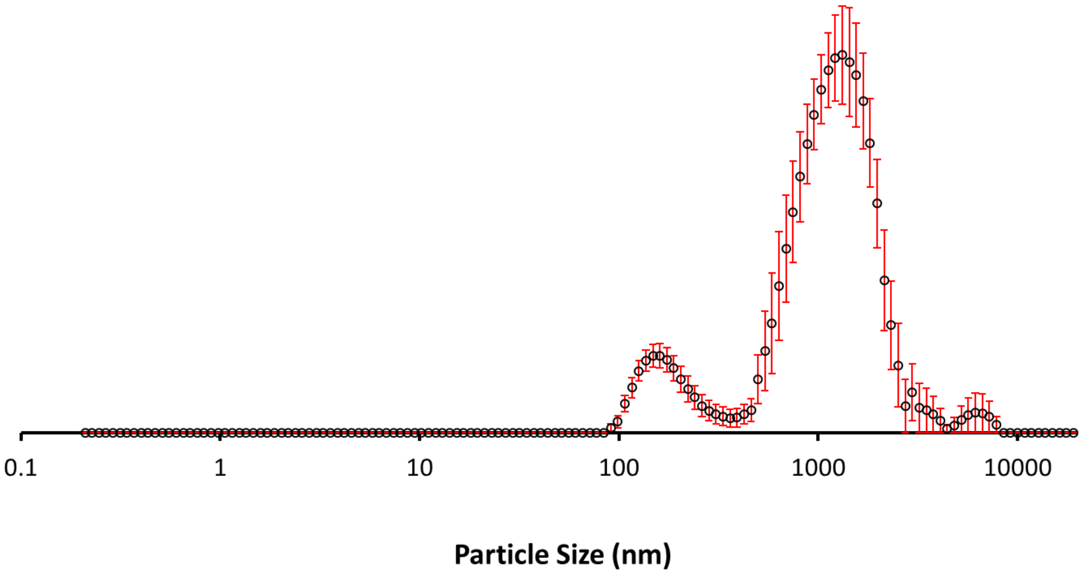

Figure S110 - The average intensity particle size distribution calculated using 10 DLS runs for co-formulation ***e*** (5.56 mM) in an EtOH:H_2O (1:19) solution at 298 K.

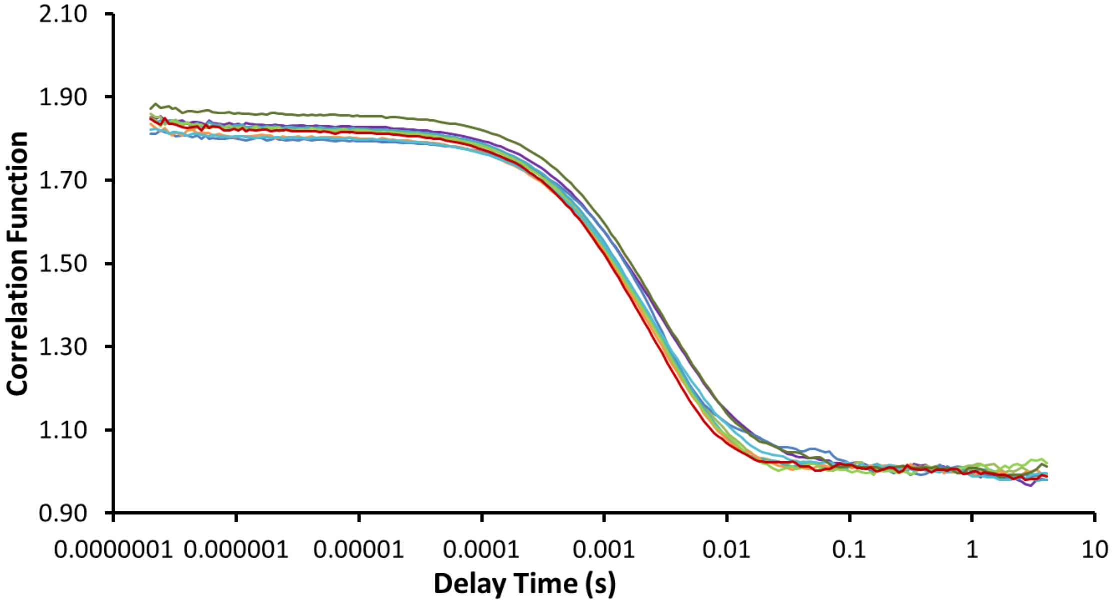

Figure S111 - Correlation function data for 10 DLS runs of co-formulation ***e*** (5.56 mM) in an EtOH:H_2O (1:19) solution at 298 K.

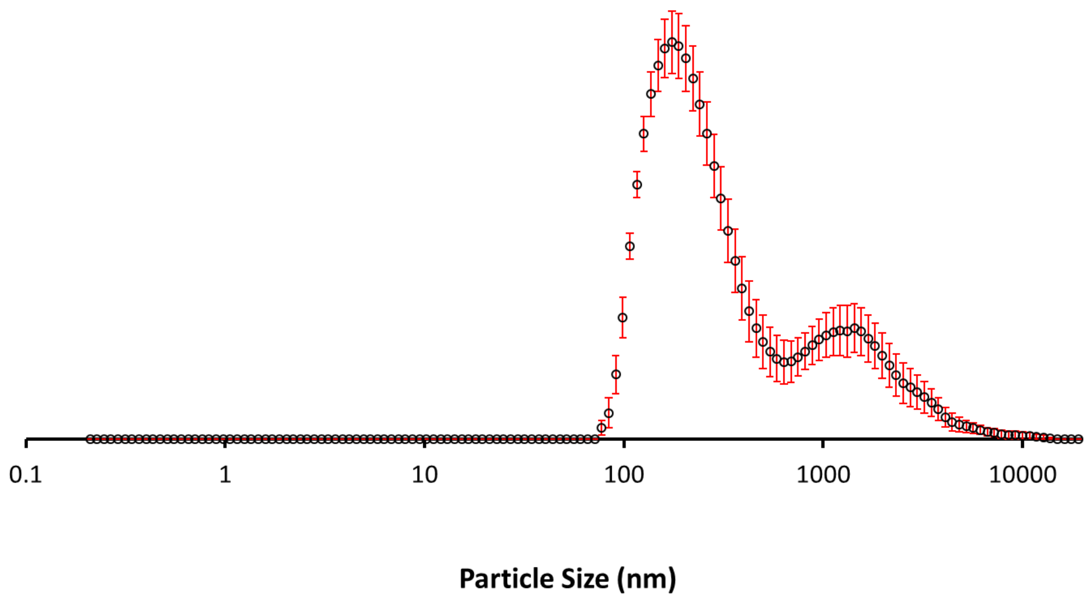

Figure S112 - The average intensity particle size distribution calculated using 10 DLS runs for co-formulation ***e*** (0.56 mM) in an EtOH:H_2O (1:19) solution at 298 K.

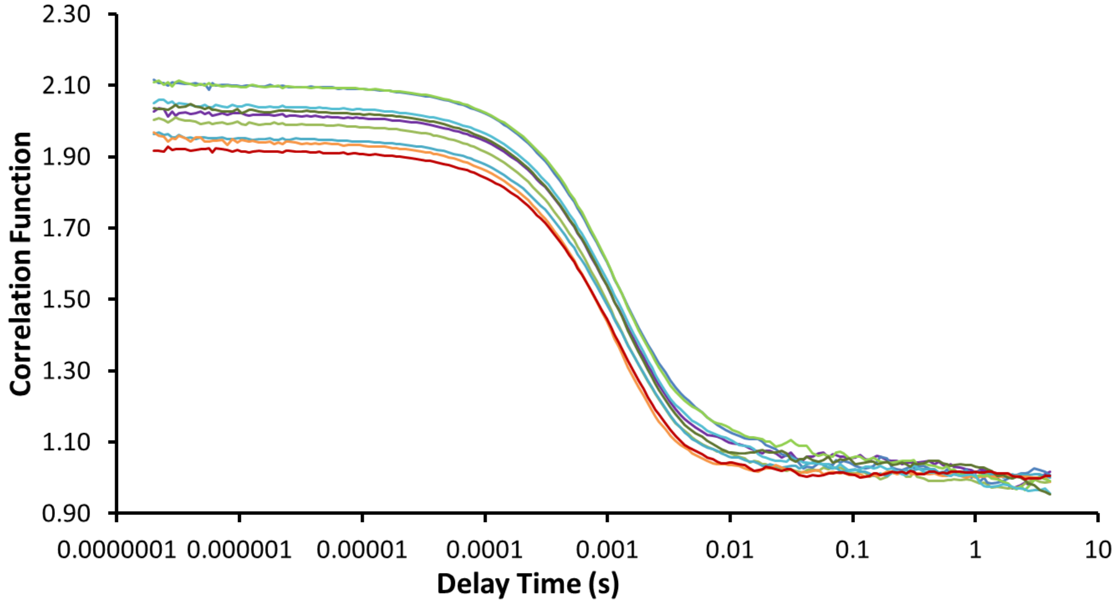

Figure S113 - Correlation function data for 10 DLS runs of co-formulation ***e*** (0.56 mM) in an EtOH:H_2O (1:19) solution at 298 K.

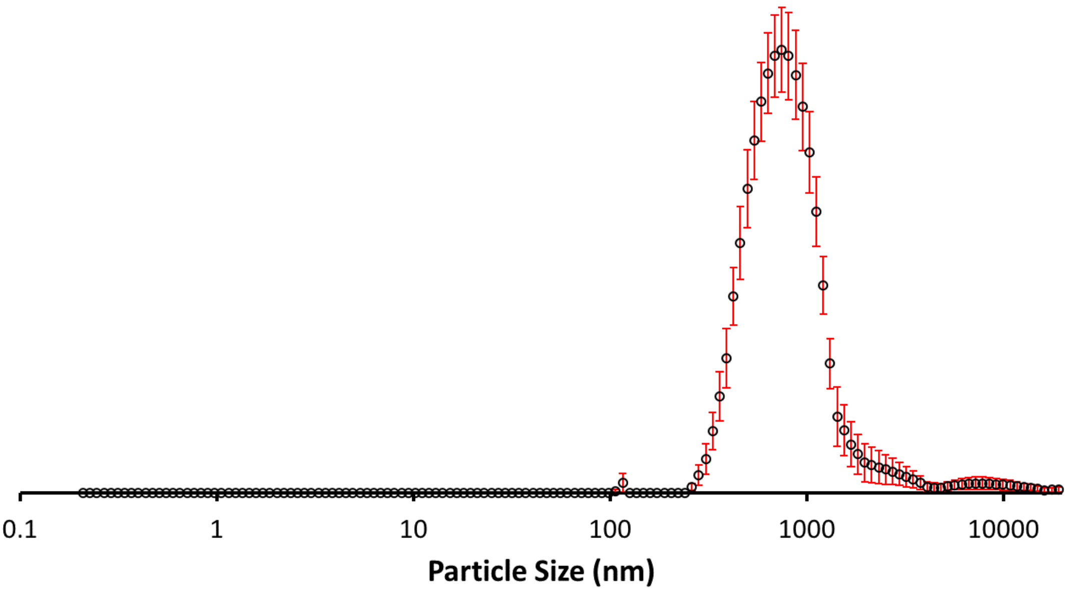

Figure S114 - The average intensity particle size distribution calculated using 10 DLS runs for co-formulation ***f*** (5.56 mM) in an EtOH:H_2O (1:19) solution at 298 K.

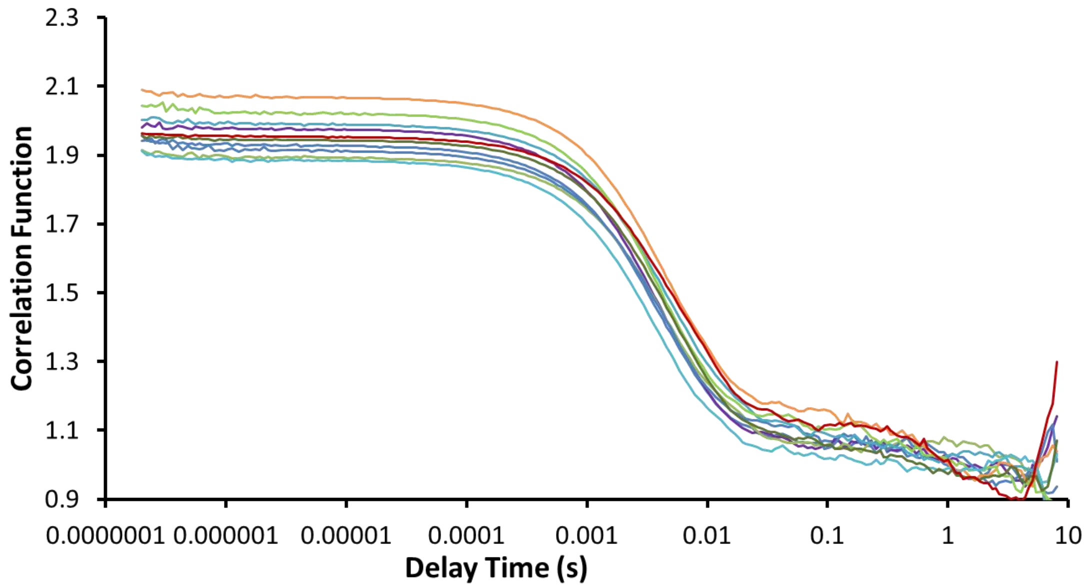

Figure S115 - Correlation function data for 10 DLS runs of co-formulation ***f*** (5.56 mM) in an EtOH:H_2O (1:19) solution at 298 K.

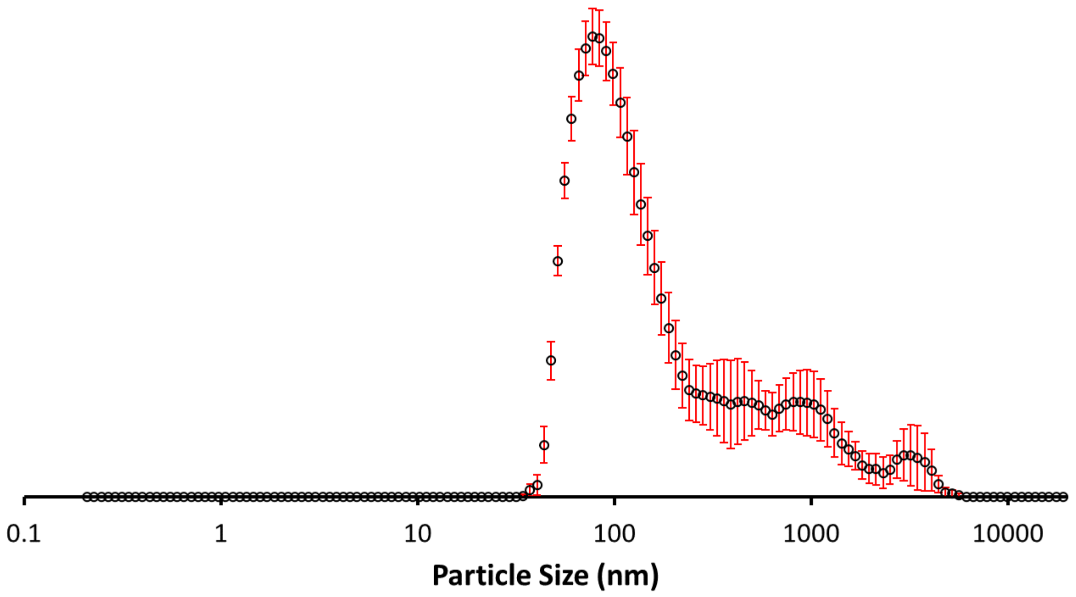

Figure S116 - The average intensity particle size distribution calculated using 10 DLS runs for co-formulation ***f*** (0.56 mM) in an EtOH:H_2O (1:19) solution at 298 K.

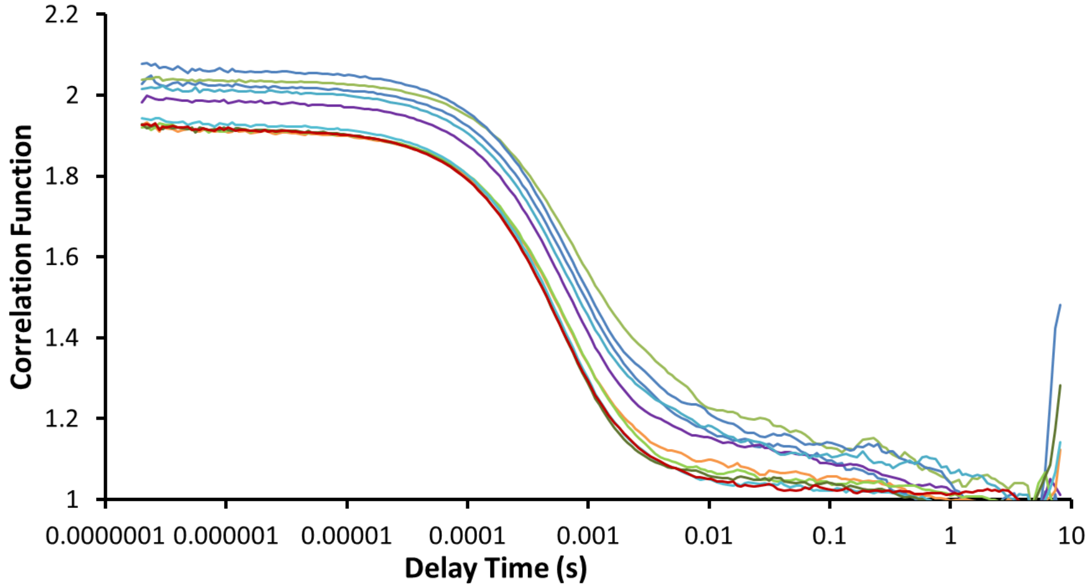

Figure S117 - Correlation function data for 10 DLS runs of co-formulation ***f*** (0.56 mM) in an EtOH:H_2O (1:19) solution at 298 K.

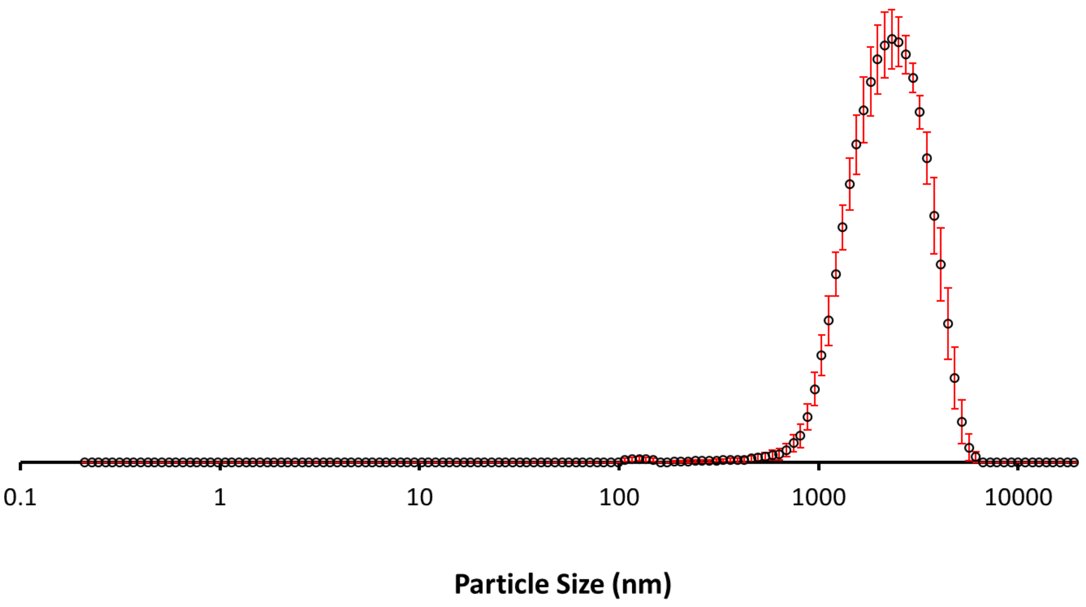

Figure S118 - The average intensity particle size distribution calculated using 10 DLS runs for co-formulation ***g*** (5.56 mM) in an EtOH:H_2O (1:19) solution at 298 K.

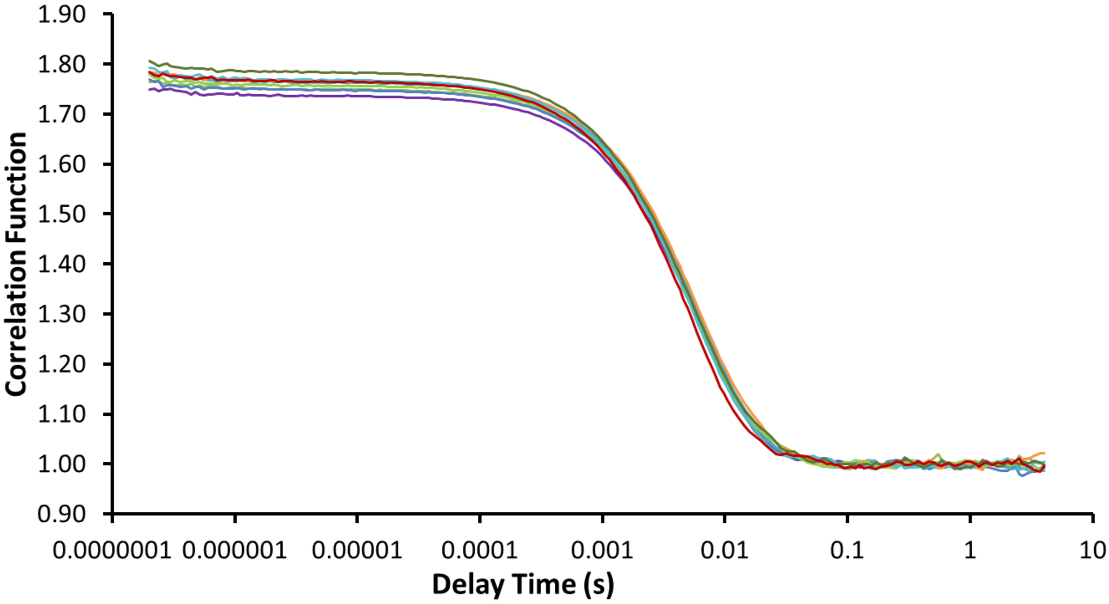

Figure 119 - Correlation function data for 10 DLS runs of co-formulation ***g*** (5.56 mM) in an EtOH:H_2O (1:19) solution at 298 K.

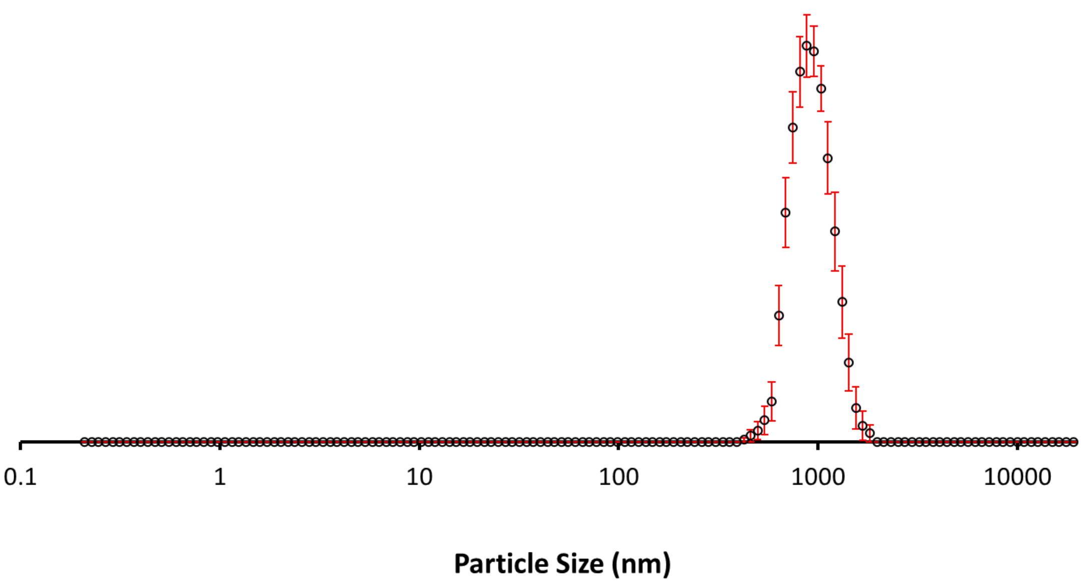

Figure S120 - The average intensity particle size distribution calculated using 10 DLS runs for co-formulation ***g*** (0.56 mM) in an EtOH:H_2O (1:19) solution at 298 K.

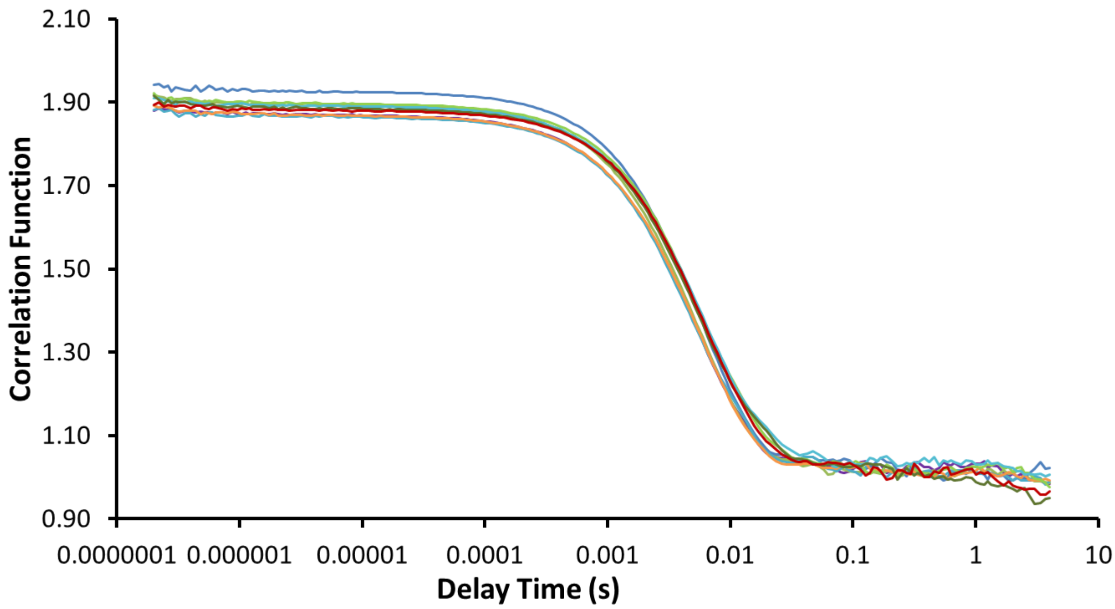

Figure S121 - Correlation function data for 10 DLS runs of co-formulation ***g*** (0.56 mM) in an EtOH:H_2O (1:19) solution at 298 K.

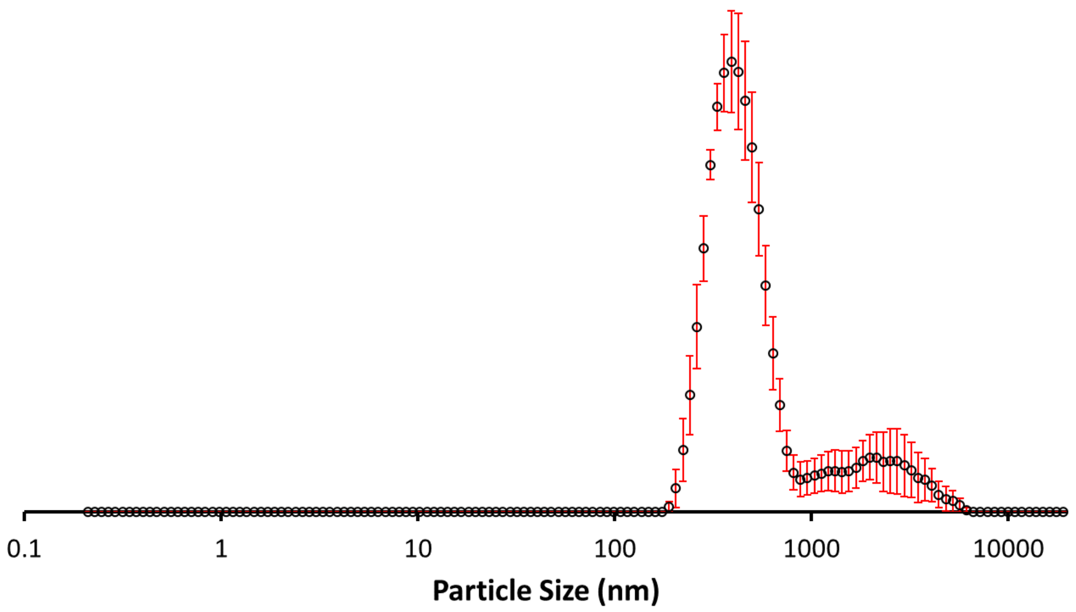

Figure S122 - The average intensity particle size distribution calculated using 9 DLS runs for co-formulation ***h*** (5.56 mM) in an EtOH:H_2O (1:19) solution at 298 K.

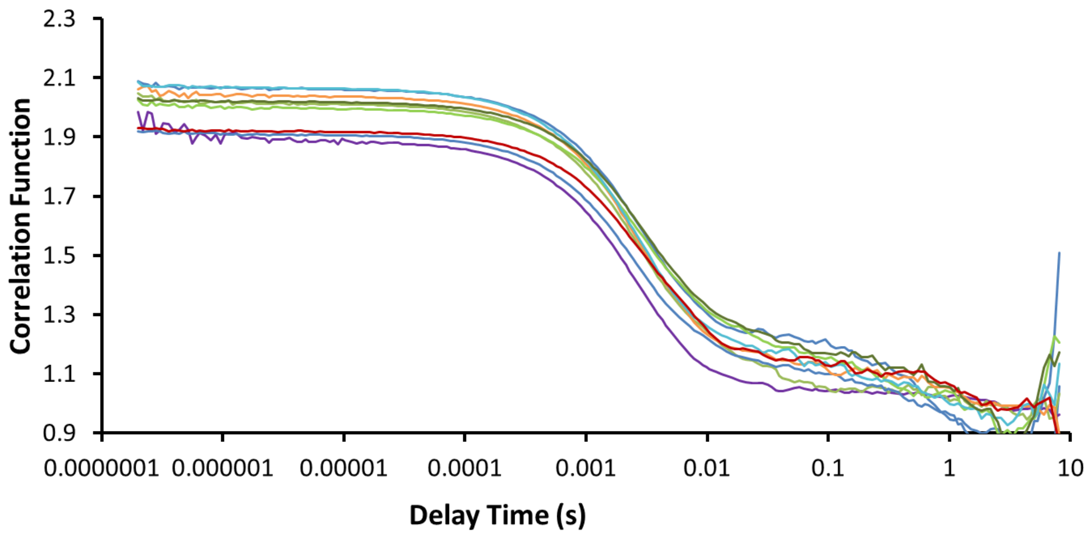

Figure S123 - Correlation function data for 9 DLS runs of co-formulation ***h*** (5.56 mM) in an EtOH:H_2O (1:19) solution at 298 K.

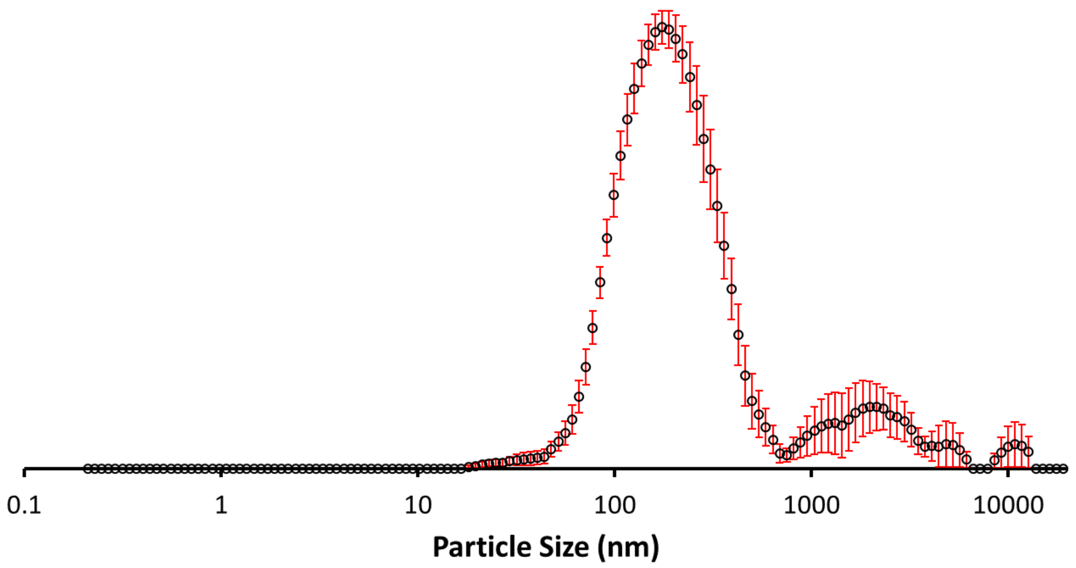

Figure S124 - The average intensity particle size distribution calculated using 9 DLS runs for co-formulation ***h*** (0.56 mM) in an EtOH:H_2O (1:19) solution at 298 K.

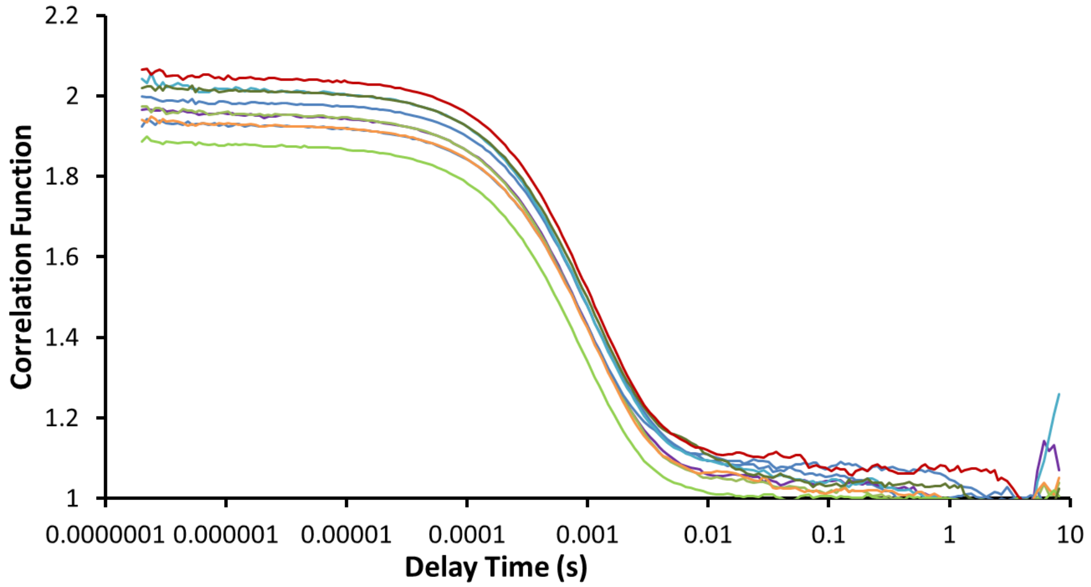

Figure S125 - Correlation function data for 9 DLS runs of co-formulation ***h*** (0.56 mM) in an EtOH:H_2O (1:19) solution at 298 K.

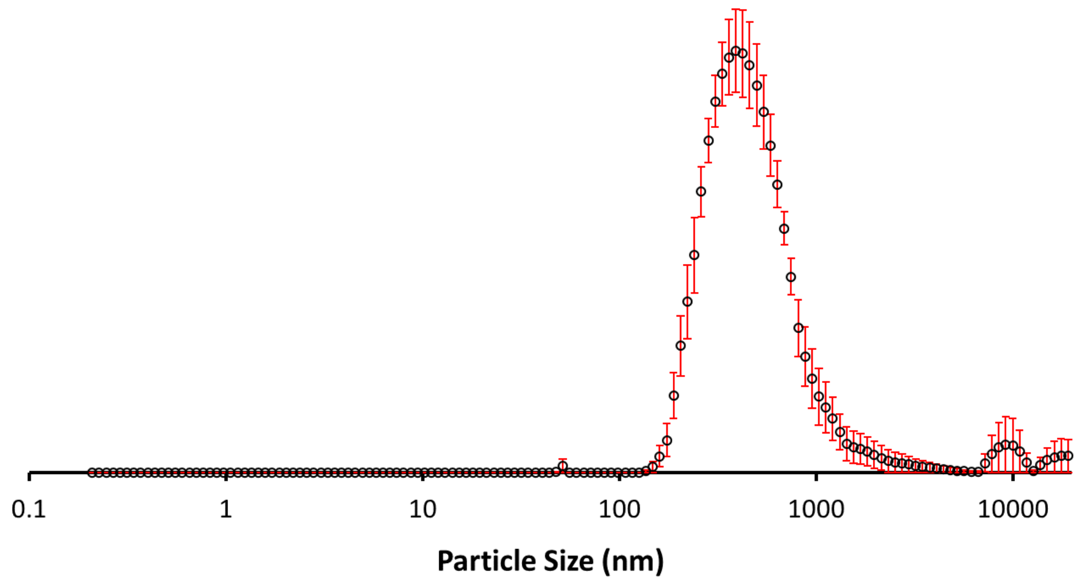

Figure S126 - The average intensity particle size distribution calculated using 10 DLS runs for co-formulation ***j*** (5.56 mM) in an EtOH:H_2O (1:19) solution at 298 K.

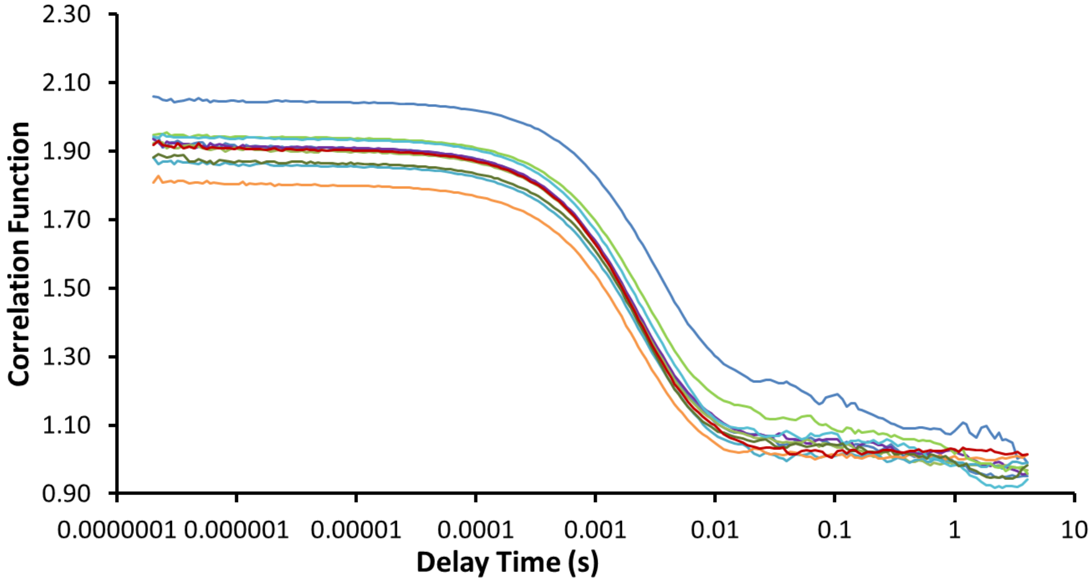

Figure S127 - Correlation function data for 10 DLS runs of co-formulation ***j*** (5.56 mM) in an EtOH:H_2O (1:19) solution at 298 K.

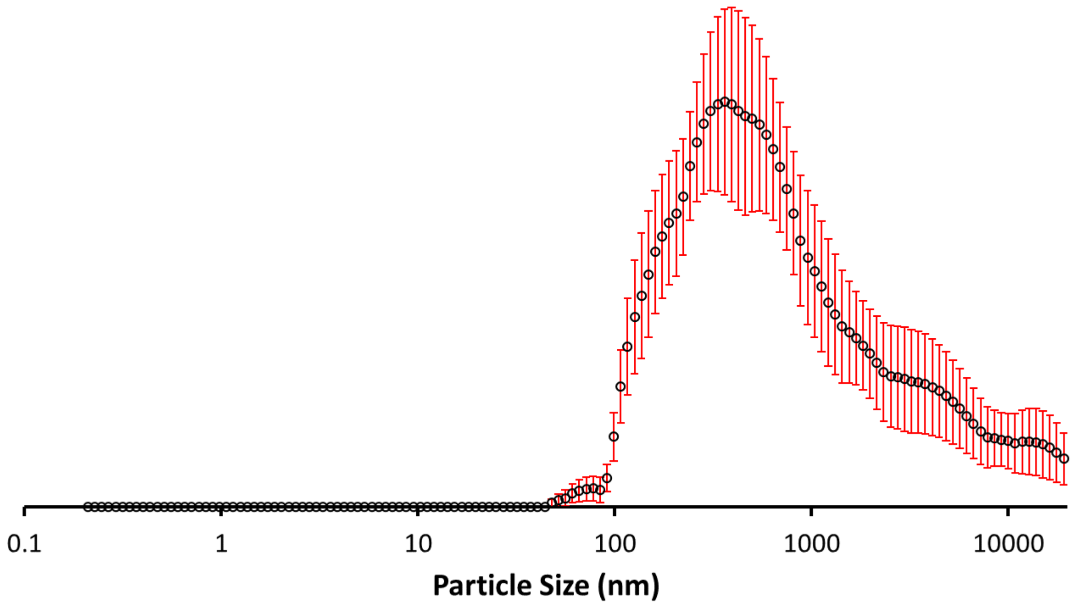

Figure S128 - The average intensity particle size distribution calculated using 10 DLS runs for co-formulation ***j*** (0.56 mM) in an EtOH:H_2O (1:19) solution at 298 K.

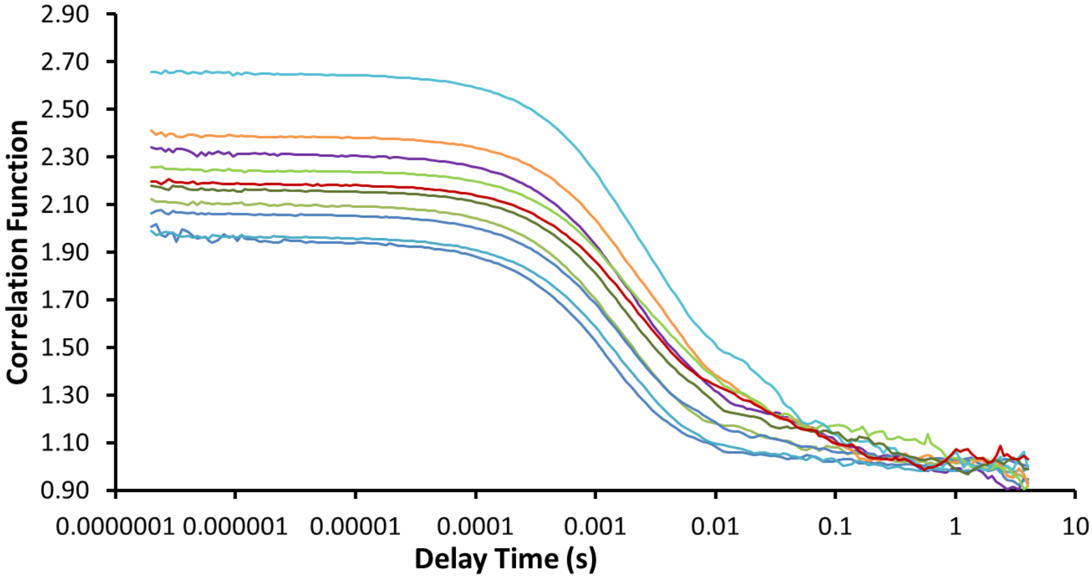

Figure S129 - Correlation function data for 10 DLS runs of co-formulation ***j*** (0.56 mM) in an EtOH:H_2O (1:19) solution at 298 K.

Overview

Table S6 – Summary of average intensity particle size distribution data. Error = standard error of the mean.

Compound	Concentration (mM)	Peak maxima (nm)	Polydispersity (%)
**3**	5.56	159 ± 5.3690	26 ± 1.0602
	0.56	129 ± 2.4277	24 ± 0.2458
**10**	5.56	1082 ± 134.3380	90800 ± 90751
	0.56	522 ± 23.46933	26 ± 0.4366
e	5.56	1317 ± 82.3700	29 ± 0.8785
	0.56	226 ± 17.1839	28 ± 0.6501
f	5.56	768 ± 23.2272	27 ± 0.7193
	0.56	105 ± 6.1937	28 ± 1.1428
g	5.56	2391 ± 63.5267	25 ± 1.2733
	0.56	946 ± 23.7185	17 ± 2.2732
h	5.56	427 ± 20.2907	27 ± 1.6853
	0.56	204 ± 9.2603	26 ± 0.8902
j	5.56	466 ± 15.8156	67 ± 1.2312
	0.56	527 ± 172.1825	26 ± 1.7973

Surface Tension and Stability data

Zeta Potential

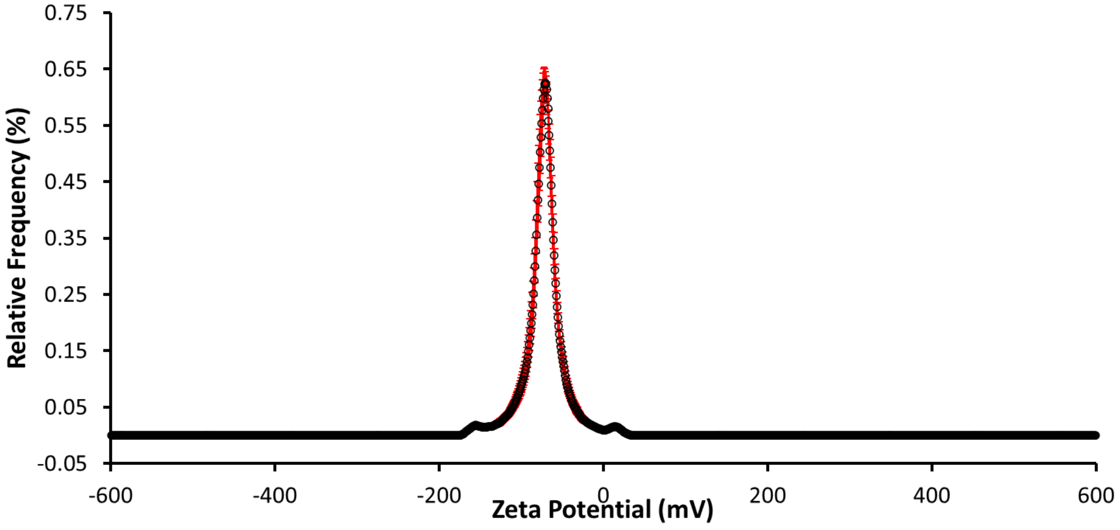

Figure S130 - The average zeta potential distribution calculated using 10 runs for compound **3** (5.56 mM) in an EtOH:H_2O (1:19) solution at 298 K. Average measurement value **-**65.2 mV.

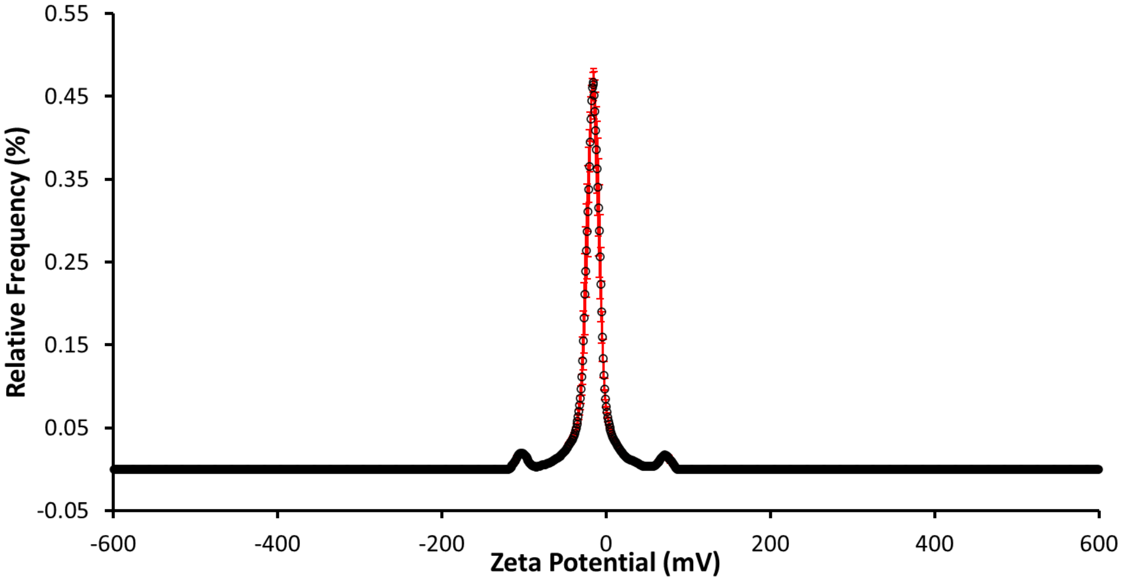

Figure S131 - The average zeta potential distribution calculated using 9 runs for compound **10** (5.56 mM) in an EtOH:H_2O (1:19) solution at 298 K. Average measurement value **-**13.6 mV.

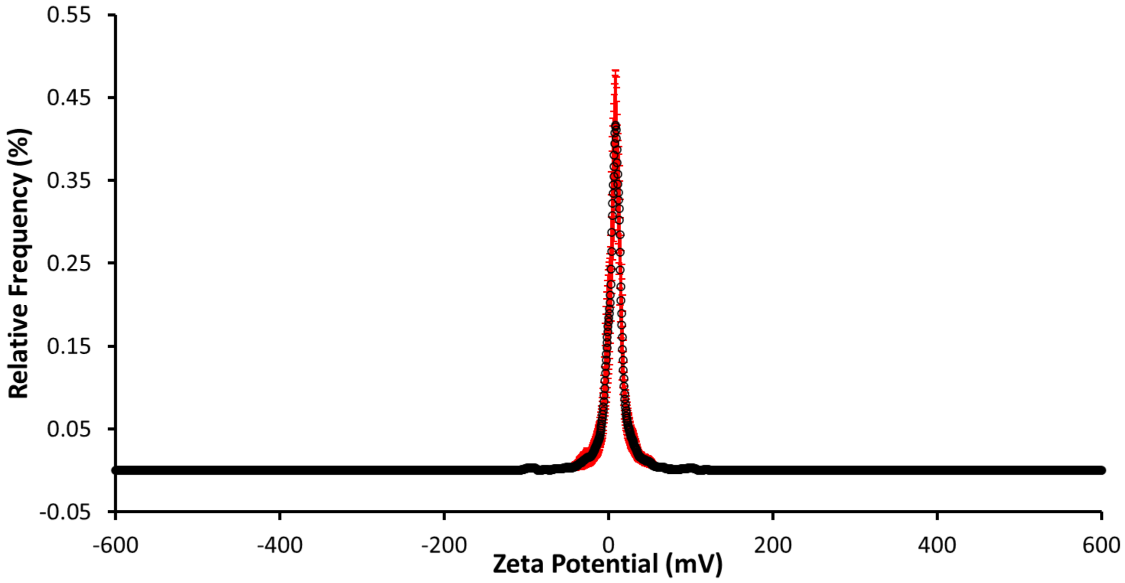

Figure S132 - The average zeta potential distribution calculated using 10 runs for compound **11** (5.56 mM) in an EtOH:H_2O (1:19) solution at 298 K. Average measurement value **-**7.4 mV.

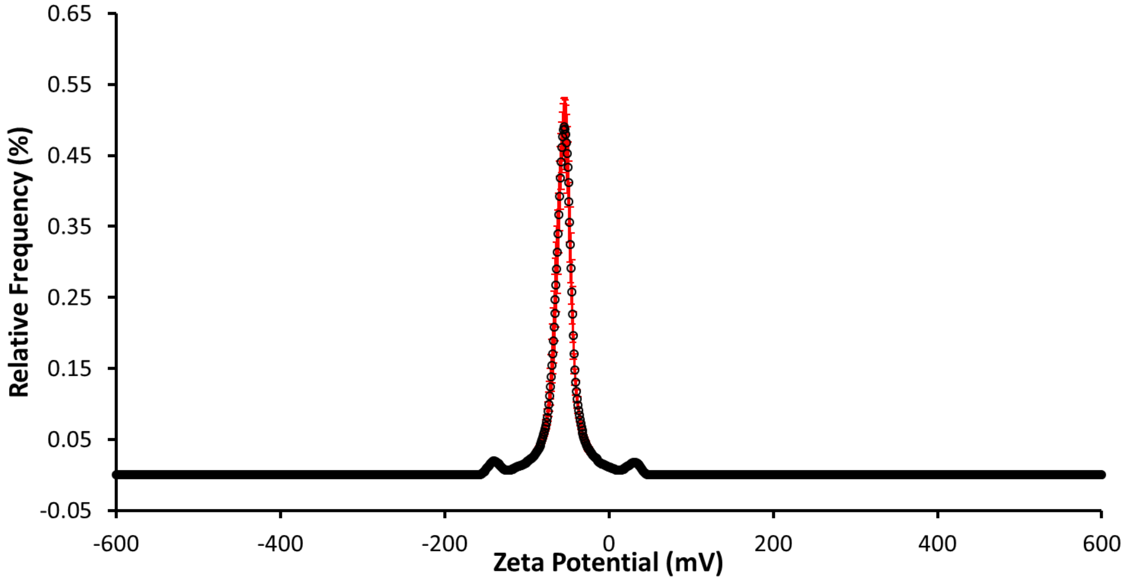

Figure S133 - The average zeta potential distribution calculated using 10 runs for co-formulation ***e*** (5.56 mM) in an EtOH:H_2O (1:19) solution at 298 K. Average measurement value **-**51.8 mV.

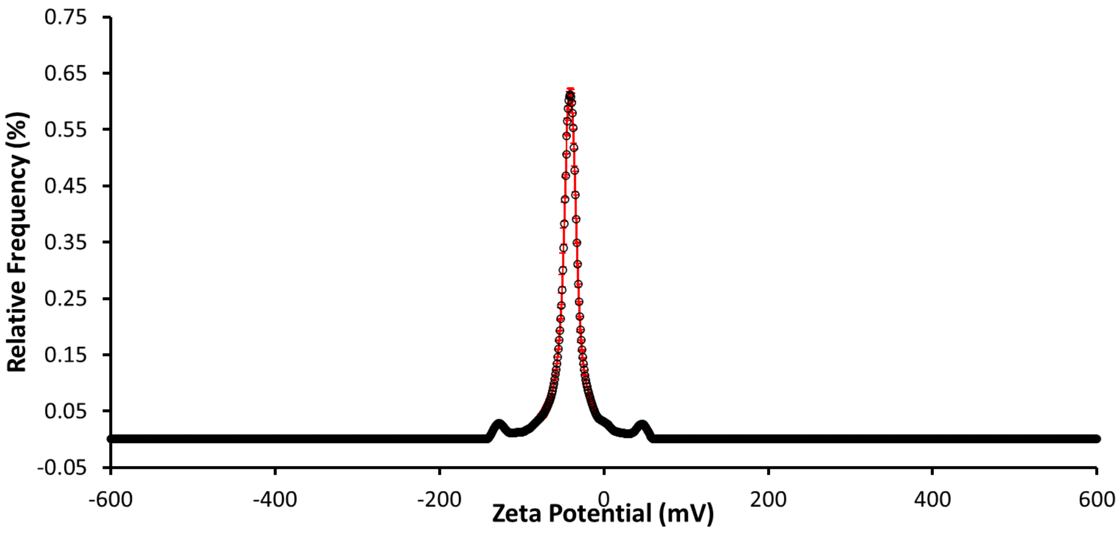

Figure S134 - The average zeta potential distribution calculated using 10 runs for co-formulation ***f*** (5.56 mM) in an EtOH:H_2O (1:19) solution at 298 K. Average measurement value -42.7 mV.

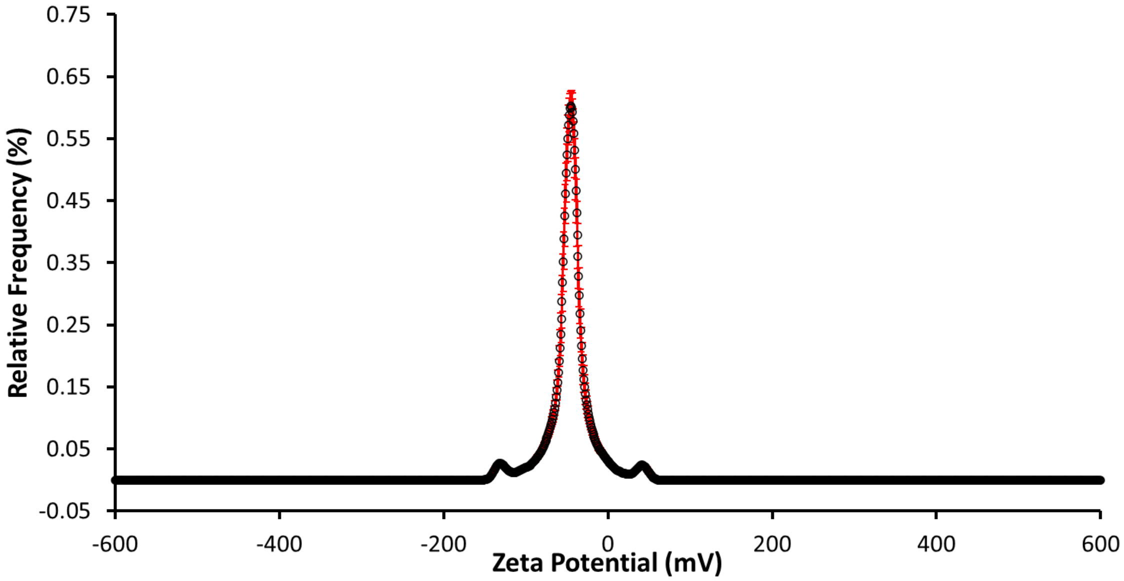

Figure S135 - The average zeta potential distribution calculated using 10 runs for co-formulation ***g*** (5.56 mM) in an EtOH:H_2O (1:19) solution at 298 K. Average measurement value -52.1 mV

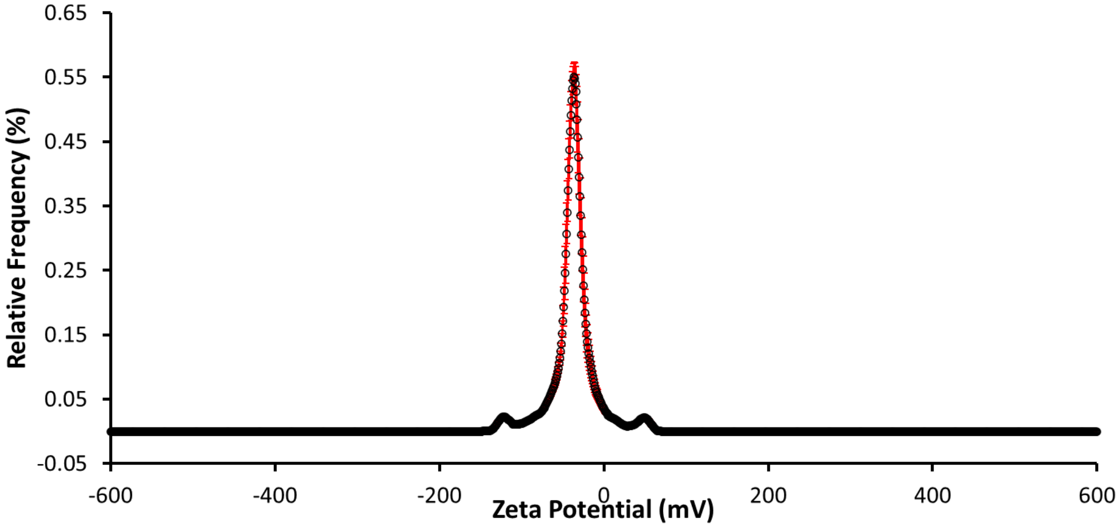

Figure S136 - The average zeta potential distribution calculated using 10 runs for co-formulation ***h*** (5.56 mM) in an EtOH:H_2O (1:19) solution at 298 K. Average measurement value **-**35.8 mV.

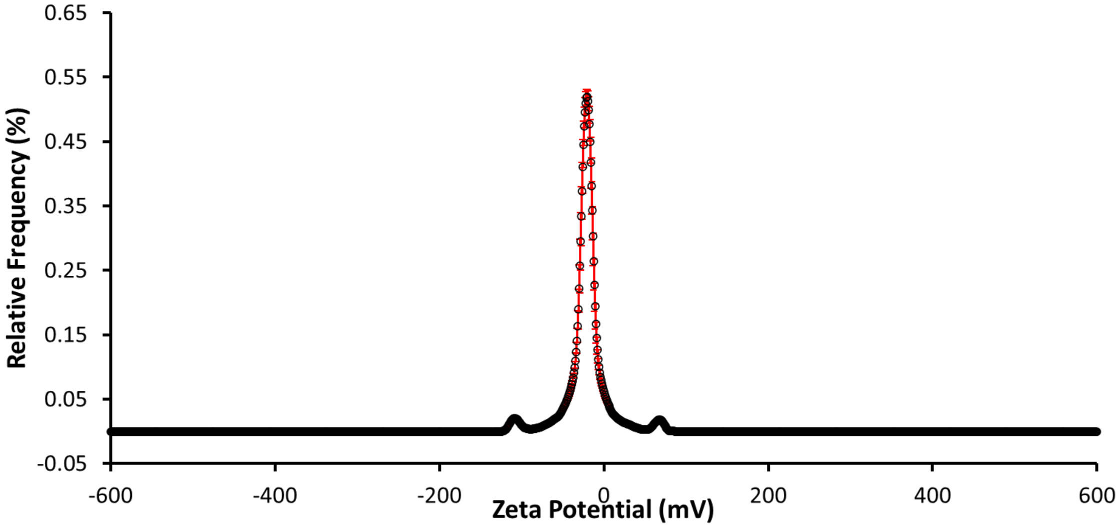

Figure S137 - The average zeta potential distribution calculated using 10 runs for co-formulation ***j*** (5.56 mM) in an EtOH:H_2O (1:19) solution at 298 K. Average measurement value **-**20.7 mV.

Surface Tension Measurements and Critical Micelle Concentration (CMC) Determination

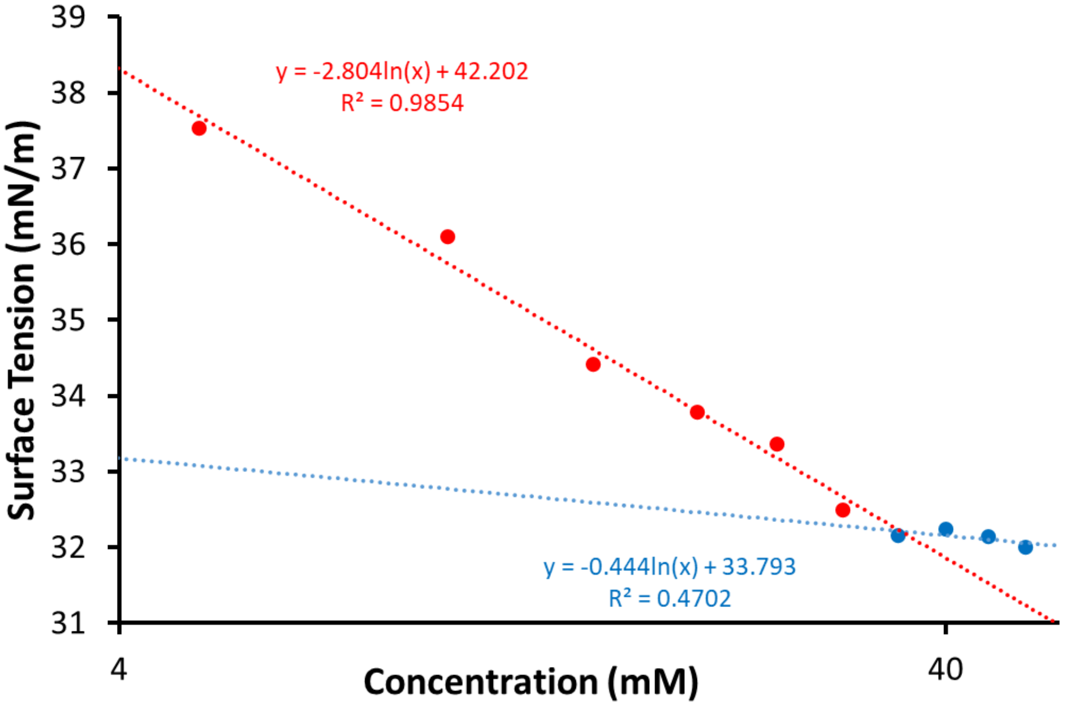

Figure 138 - Calculation of CMC (35.27 mM) for compound **3** in an EtOH:H_2O 1:19 mixture using surface tension measurements.

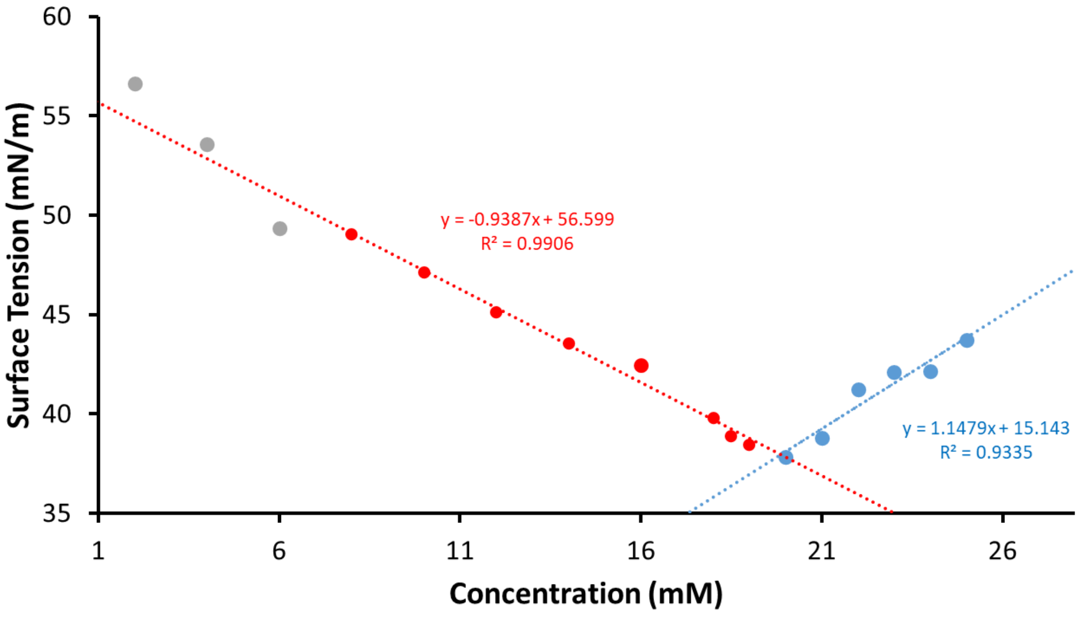

Figure S139 - Calculation of CMC (19.87 mM) for co-formulation ***f*** in an EtOH:H_2O 1:19 mixture using surface tension measurements.

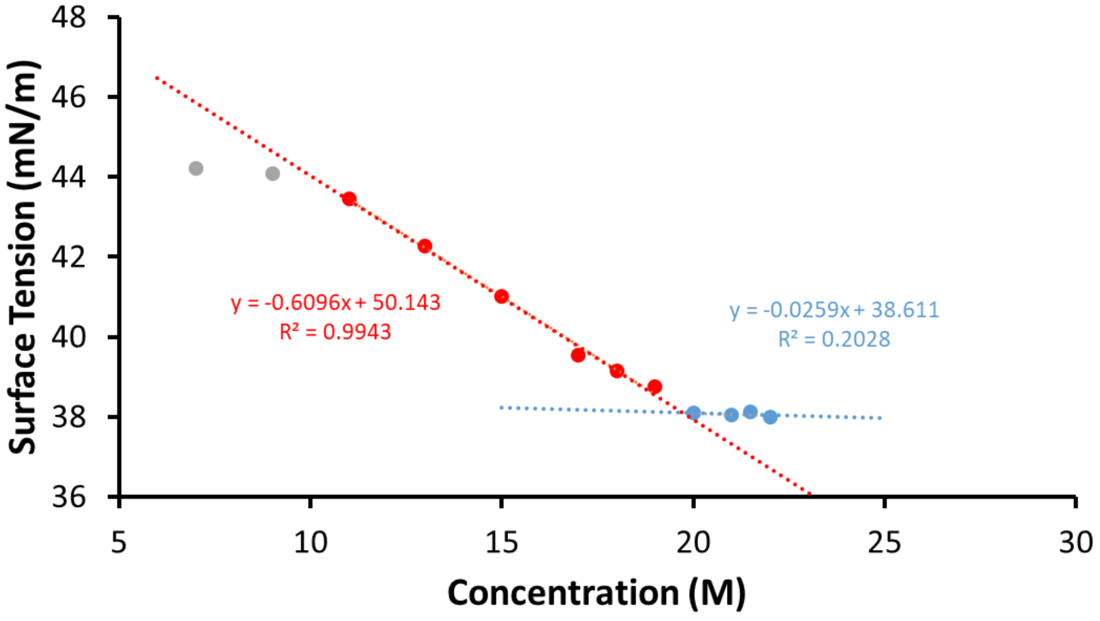

Figure S140 - Calculation of CMC (19.76 mM) for co-formulation ***h*** in an EtOH:H_2O 1:19 mixture using surface tension measurements.

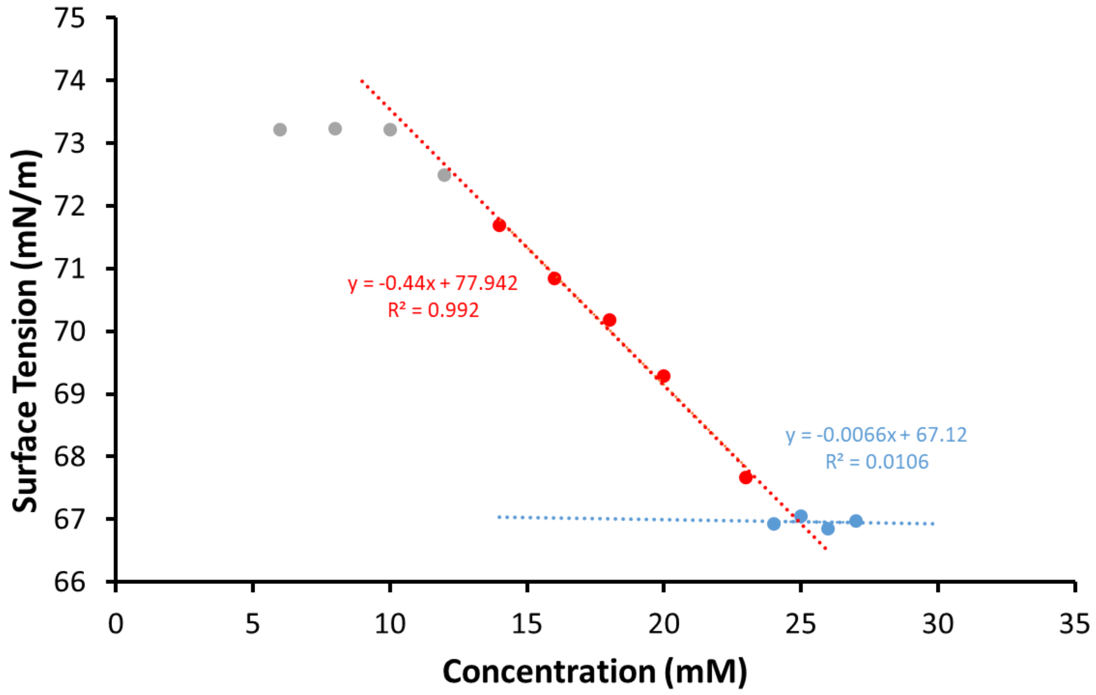

Figure S141 - Calculation of CMC (24.23 mM) for co-formulation ***j*** in an EtOH:H_2O 1:19 mixture using surface tension measurements.

Overview

Table S7 – Summary of zeta potential at 5.56 mM, CMC and surface tension at CMC. Data obtained in an EtOH:H_2O (1:19) solution.

Compound	Zeta potential (mV)	CMC (mM)	Surface tension at CMC (mN/m)
**3**	-65.2	35.27	32.21
**10**	-13.6	*a*	*a*
**11**	-7.4	*a*	*a*
e	-51.8	*a*	*a*
f	-42.7	19.87	37.95
h	-35.8	19.76	38.10
j	-52.1	*a*	*a*
j	-20.7	24.23	67.28

a – Could not be calculated due to compound solubility.

Single crystal X-ray structures

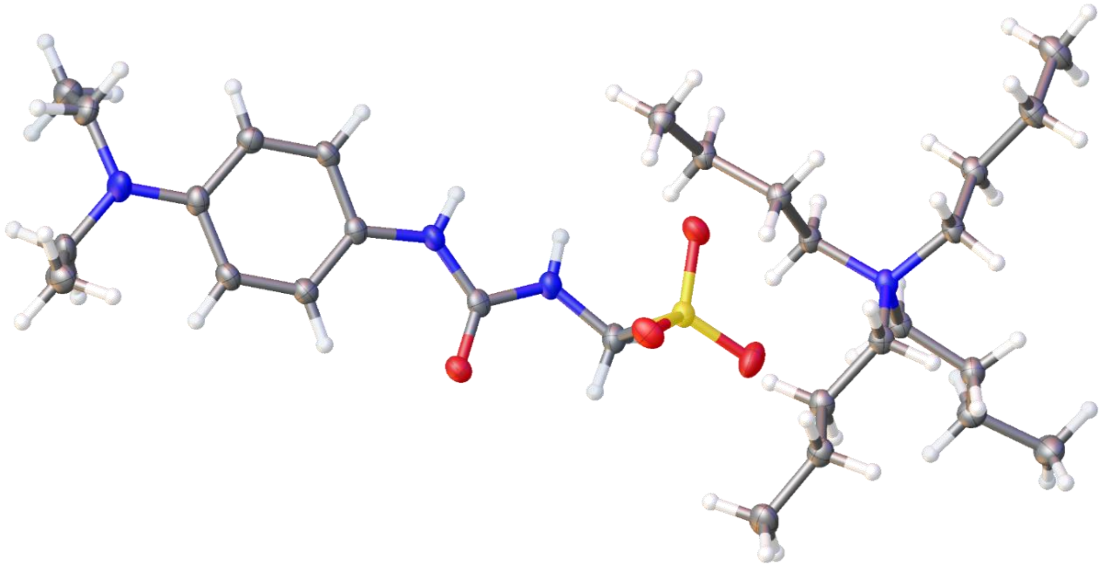

Figure S142 - Single crystal X-ray structure of compound **3**: red = oxygen; yellow = sulfur; blue = nitrogen; white = hydrogen; grey = carbon. CCDC 1997432, $C_{28}H_{54}N_4O_4S$ (M = 542.81): monoclinic, space group I 2/a, a = 16.6604(2) Å, b = 17.1219(2) Å, c = 22.4274(3) Å, α = 90°, β = 110.7979(15)°, γ = 90°, V = 5980.67(14) Å^3, Z = 8, T = 100(1) K, CuK\α = 1.5418 Å, Dcalc = 1.206 g/cm^3, 21185 reflections measured (7.674 ≤ 2Θ ≤ 146.186), 5901 unique (R_{int} = 0.0213, R_{sigma} = 0.0191) which were used in all calculations. The final R_1 was 0.0302 (I > 2σ(I)) and wR_2 was 0.0804 (all data).

Table S8 - Hydrogen bond distances and angles observed for compound **3** calculated from the single crystal X-ray structure shown above.

**Compound**	**Hydrogen bond donor**	**Hydrogen bond acceptor**	**Hydrogen bond length (D•••A) (Å)**	**Hydrogen bond angle (D-H•••A) (°)**
**6**	N1	O2	3.0076 (14)	153.4 (14)
**6**	N2	O2	2.8223 (12)	165.4 (14)

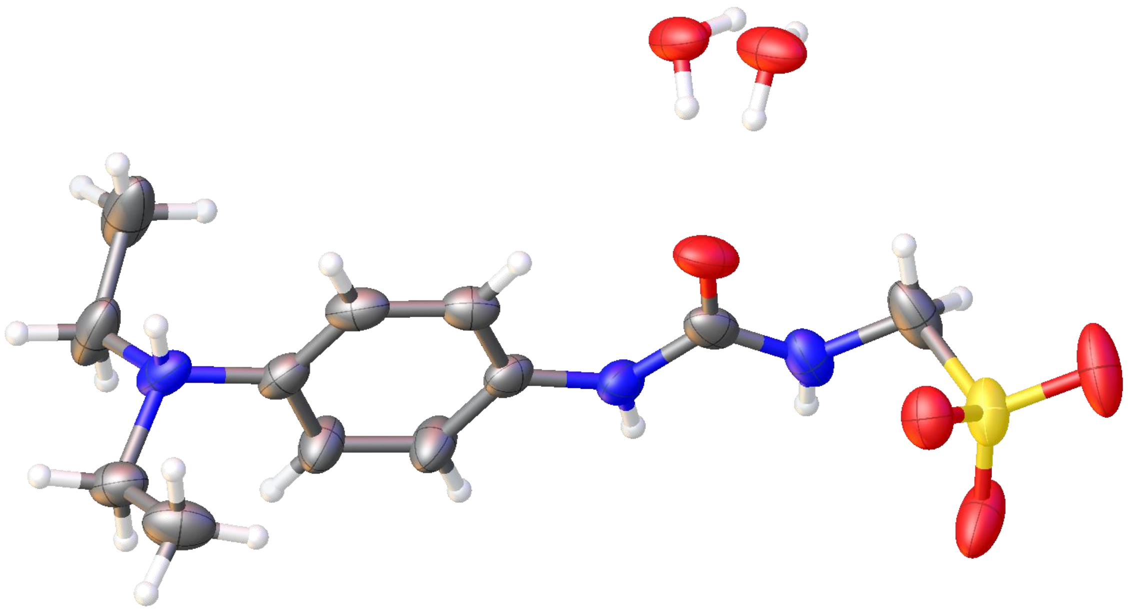

Figure S143 - Single crystal X-ray structure of compound **3**/co-formulation ***h*** zwitterion: red = oxygen; blue = nitrogen; white = hydrogen; yellow = sulfur; grey = carbon. CCDC 1997433, $C_{12}H_{21}N_3O_5S$ (M =319.38): monoclinic, space group C 2/c, a = 19.9354(7) Å, b = 12.8304(4) Å, c = 12.7910(5) Å, α = 90°, β = 97.976(3)°, γ = 90°, V = 3240.02(19) Å^3, Z = 8, T = 100(1) K, CuK\α = 1.5418 Å, Dcalc = 1.309 g/cm^3, 11531 reflections measured (8.218 ≤ 2Θ ≤ 146.046), 3194 unique (R_{int} = 0.0270, R_{sigma} = 0.0206) which were used in all calculations. The final R_1 was 0.0533 (I > 2σ(I)) and wR_2 was 0.1522 (all data). Disorder was observed for the hydrogen bonded water molecule.

Table S9 - Hydrogen bond distances and angles observed for compound **3**/co-formulation ***h*** zwitterion calculated from the single crystal X-ray structure shown above.

Compound	Hydrogen bond donor	Hydrogen bond acceptor	Hydrogen bond length (D•••A) (Å)	Hydrogen bond angle (D-H•••A) (°)
**8**	N1	O2	2.854 (3)	166.80 (15)
**8**	N2	O3	2.936 (3)	162.02 (14)
**8**	N3	O5	2.741 (4)	178 (3)
**8**	N3	O5A	2.590 (7)	136 (3)
**8**	O5	O4	2.686 (3)	174.85 (19)
**8**	O5	O2	2.686 (3)	176.48 (13)
**8**	O5A	O4	2.618 (6)	167.20 (50)
**8**	O5A	O2	2.618 (6)	167.20 (50)

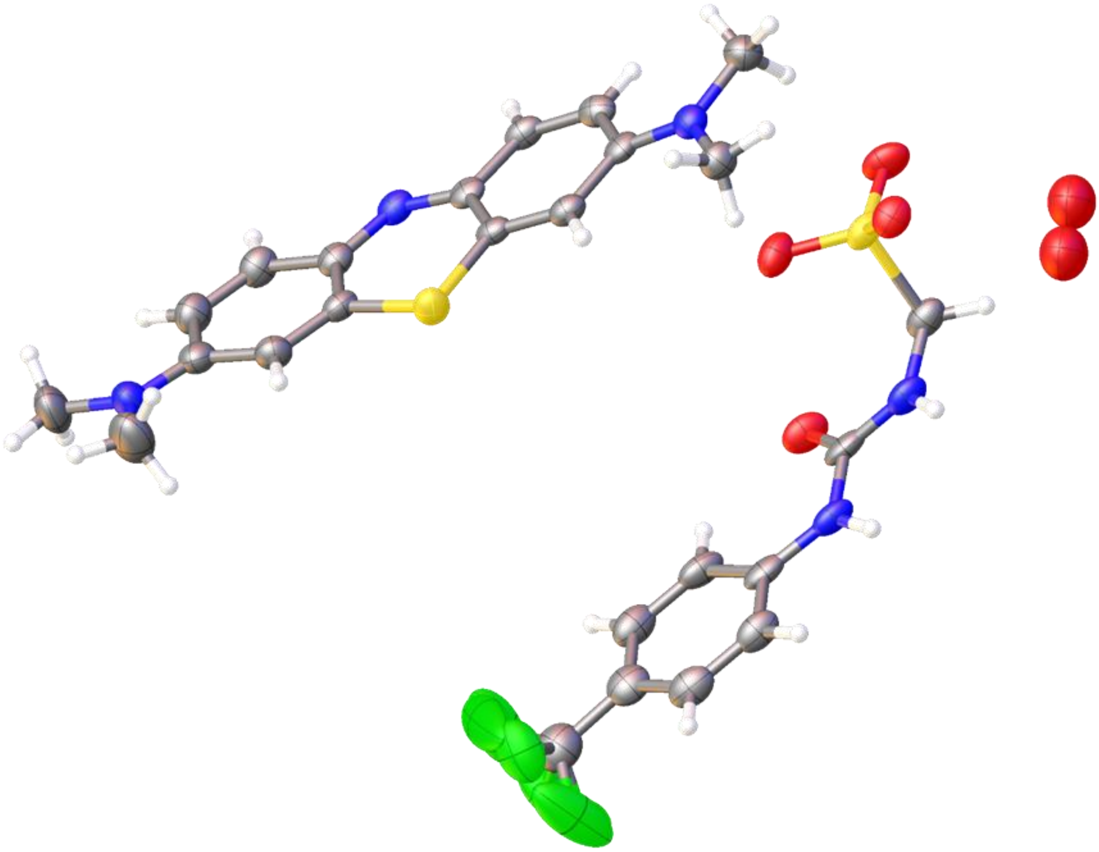

Figure S144 - Single crystal X-ray structure of compound **11** red = oxygen; yellow = sulfur; blue = nitrogen; white = hydrogen; grey = carbon; green = fluorine. CCDC 1997431, $C_{25}H_{26}F_3N_5O_5S_2$ (M = 597.63): monoclinic, space group P 21/c, a = 5.7994(2) Å, b = 29.6243(15) Å, c = 15.3739(5) Å, α = 90°, β = 90.375(3)°, γ = 90°, V = 2641.23(19) $Å^3$, Z = 4, T = 150(1) K, CuK\α = 1.5418 Å, Dcalc = 1.503 g/cm^3, 16388 reflections measured (8.290 ≤ 2Θ ≤ 133.158), 4655 unique (R_{int} = 0.0563, R_{sigma} = 0.0539) which were used in all calculations. The final R_1 was 0.0630 (I > 2σ(I)) and wR_2 was 0.1432 (all data). Disorder within the water molecule prevented the accurate modelling of the water molecules hydrogen atoms. For this reason, these hydrogen atoms are not included within this model. Disorder was also identified within the trifluoromethyl group.

Table S10 - Hydrogen bond distances and angles observed for compound **11** calculated from the single crystal X-ray structure shown above.

**Compound**	**Hydrogen bond donor**	**Hydrogen bond acceptor**	**Hydrogen bond length (D•••A) (Å)**	**Hydrogen bond angle (D-H•••A) (°)**
**15**	N1	O2	2.932 (4)	161.70 (20)
**15**	N2	O4	3.048 (5)	159.90 (30)

Low level *in silico* modelling

Computational calculations to identify primary hydrogen bond donating and accepting sites were conducted in line with studies reported by Hunter using Spartan 16''[4]. Calculations were performed using semi-empirical PM6 methods, after energy minimisation calculations, to identify E_{max}, E_{min} and LogP values. PM6 was used over AM1 in line with research conducted by Stewart [5].

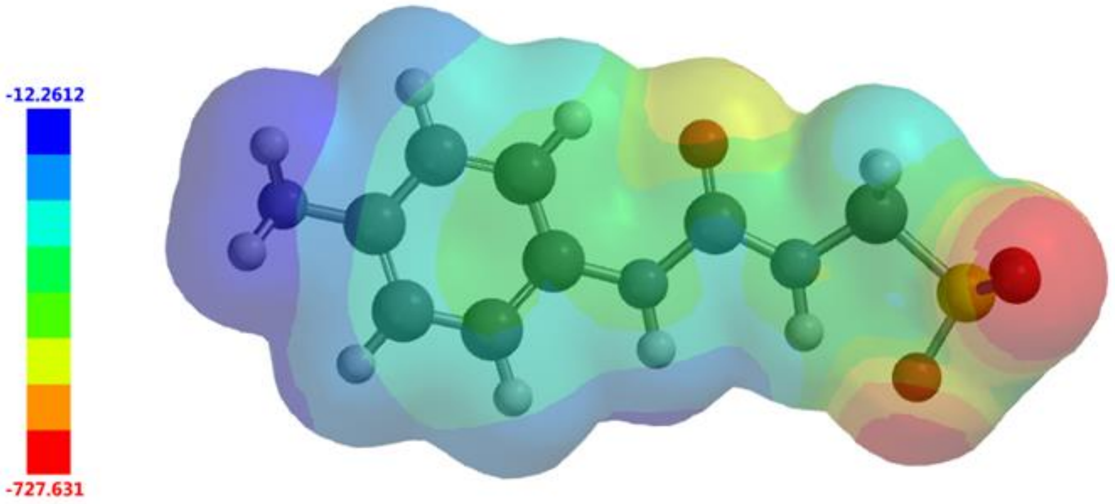

Figure S145 - Electrostatic potential map calculated for the anionic component of **2**. E_{max} and E_{min} values depicted in the figure legends are given in KJ mol^{-1}.

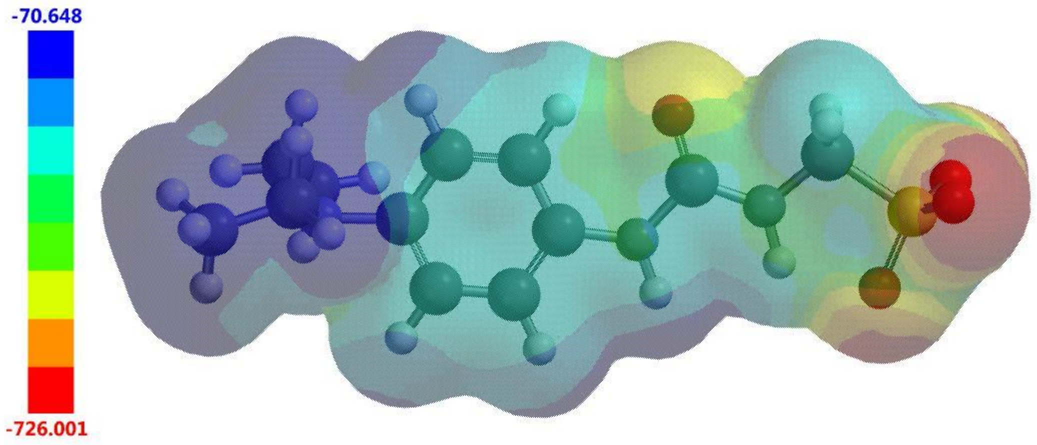

Figure S146 - Electrostatic potential map calculated for the anionic component of **3**. E_{max} and E_{min} values depicted in the figure legends are given in KJ mol^{-1}.

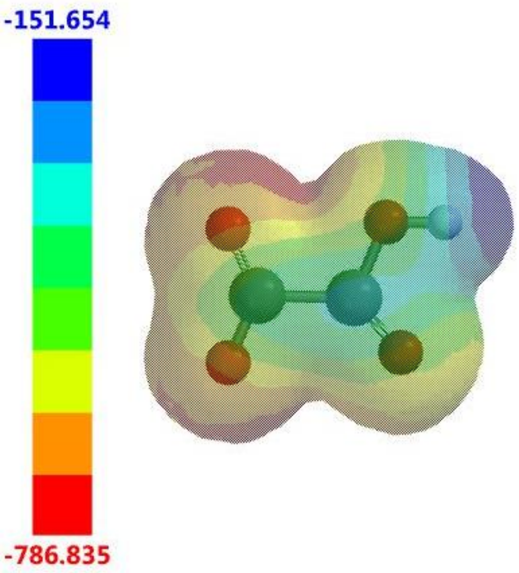

Figure S147 - Electrostatic potential map calculated for anionic component of **5**. E_{max} and E_{min} values depicted in the Figure legends are given in KJ mol^{-1}.

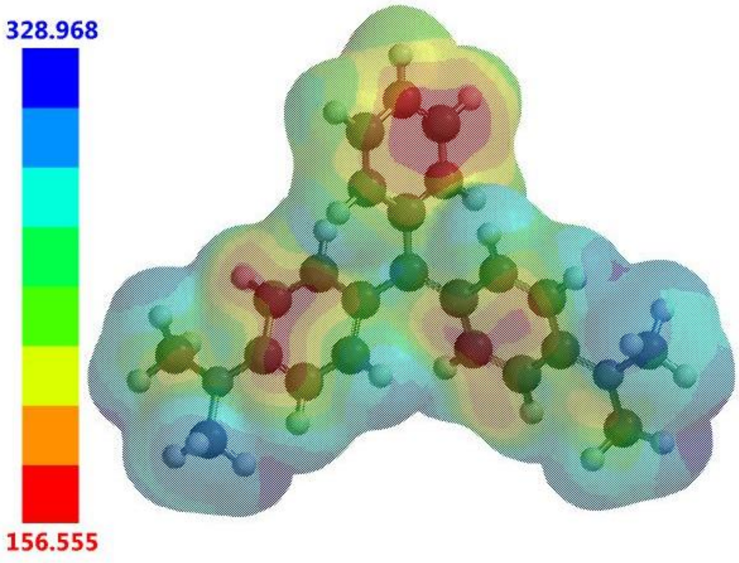

Figure S148 - Electrostatic potential map calculated for cationic component of compound **5**. E_{max} and E_{min} values depicted in the Figure legends are given in KJ mol^{-1}.

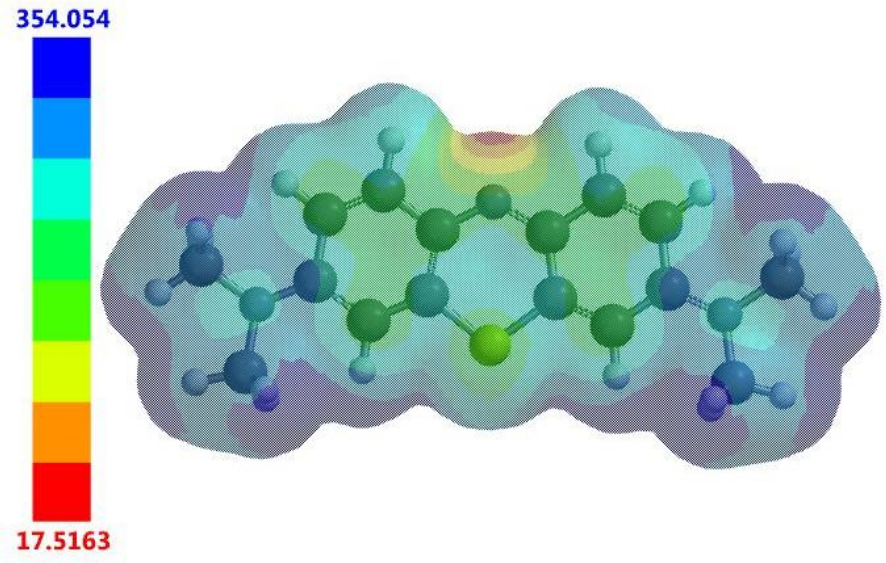

Figure S149 - Electrostatic potential map calculated for **6**. E_{max} and E_{min} values depicted in the Figure legends are given in KJ mol^{-1}.

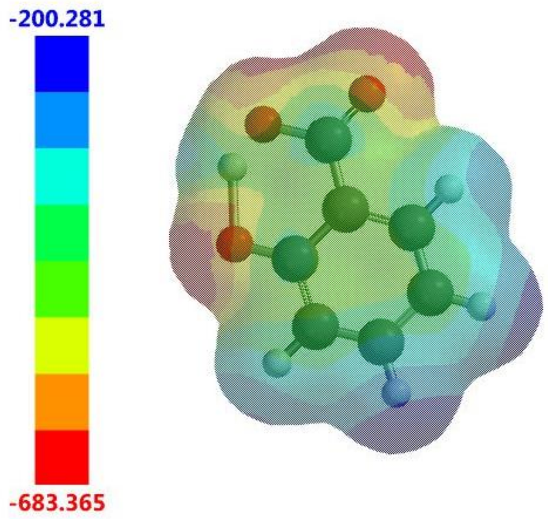

Figure S150 - Electrostatic potential map calculated for the anionic component of **8**. E_{max} and E_{min} values depicted in the figure legends are given in KJ mol^{-1}.

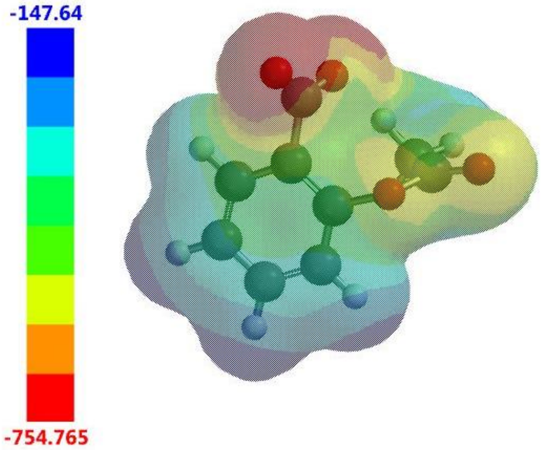

Figure S151 - Electrostatic potential map calculated for the anionic component of **9**. E_{max} and E_{min} values depicted in the figure legends are given in KJ mol^{-1}.

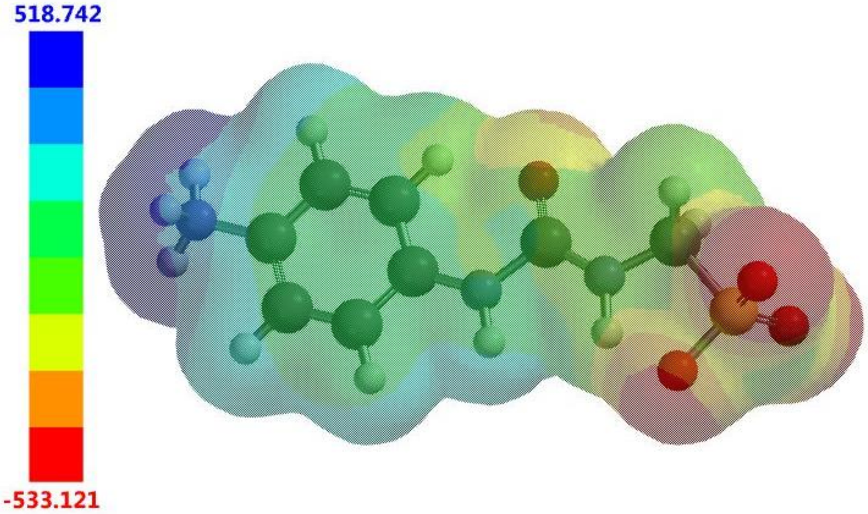

Figure S152 - Electrostatic potential map calculated for the zwitterionic component of co-formulation ***e***. E_{max} and E_{min} values depicted in the figure legends are given in KJ mol^{-1}.

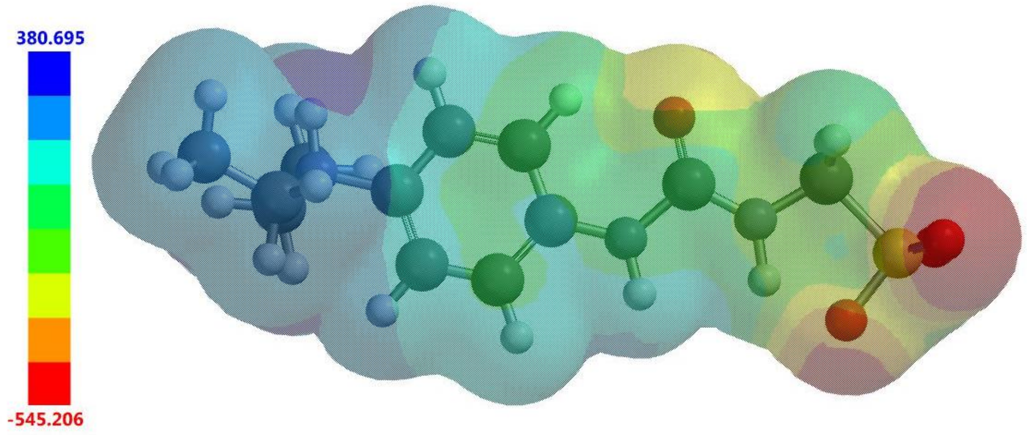

Figure S153 - Electrostatic potential map calculated for the zwitterionic component of co-formulation ***f***. E_{max} and E_{min} values depicted in the figure legends are given in KJ mol^{-1}.

Overview

Table S11 – Summary of E_{max}, E_{min} and Log*P* values.

Compound	E_{max} (KJ mol-1)	E_{min} (KJ mol^{-1})	Log*P*
**2**	-727.631	-12.261	-0.42
**3**	-726.001	-70.648	0.79
**5** anionic	-786.835	-151.654	-1.32
**5** cationic	156.555	328.968	1.94 *a*/ 2.33 *b*
**6**	17.5163	354.054	1.38 *a*/ 2.39 b
**8**	-683.365	-200.281	0.97
**9**	-754.765	-147.64	1.02
e	-533.121	518.742	-1.30
f	-545.206	380.695	-0.21

a = with Cl^-

b = without Cl^-

Mass Spectrum data

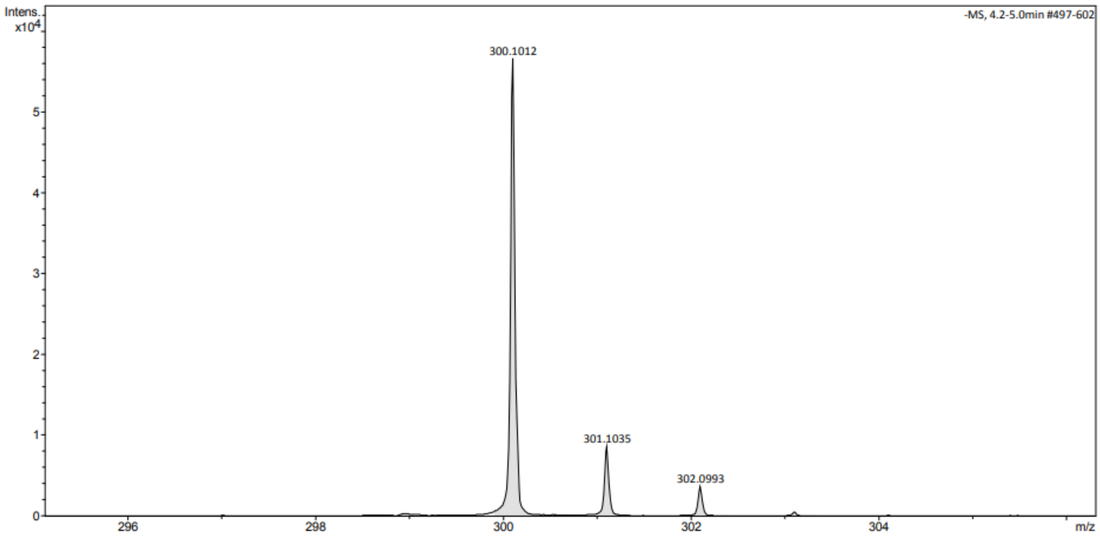

Figure S154 - A high-resolution mass spectrum (ESI$^-$) obtained for compound **3** in methanol.

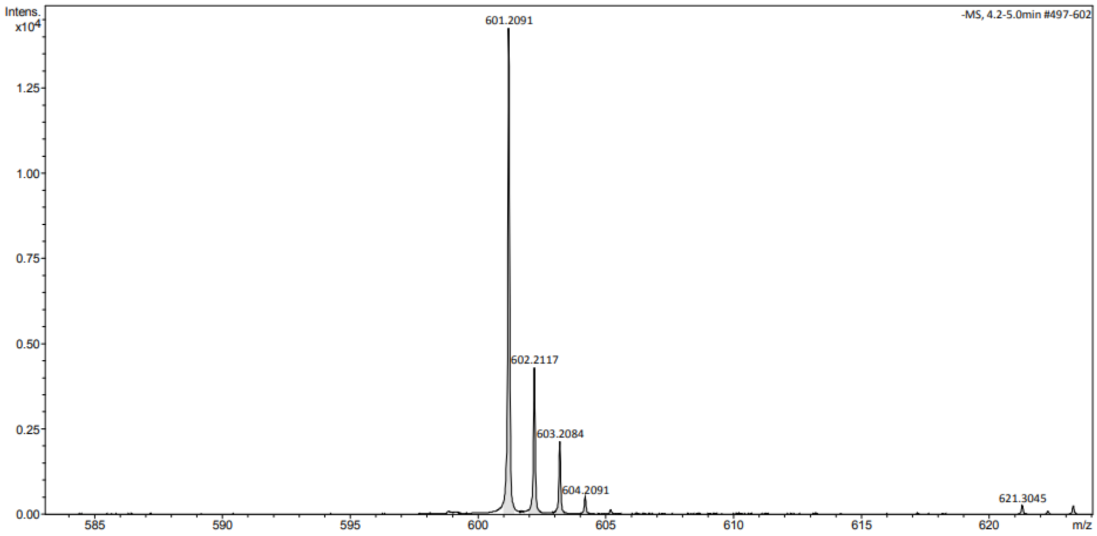

Figure S155 - A high-resolution mass spectrum (ESI$^-$) obtained for dimeric species of compound **3** in methanol, *m/z* [M + M + H$^+$]$^-$.

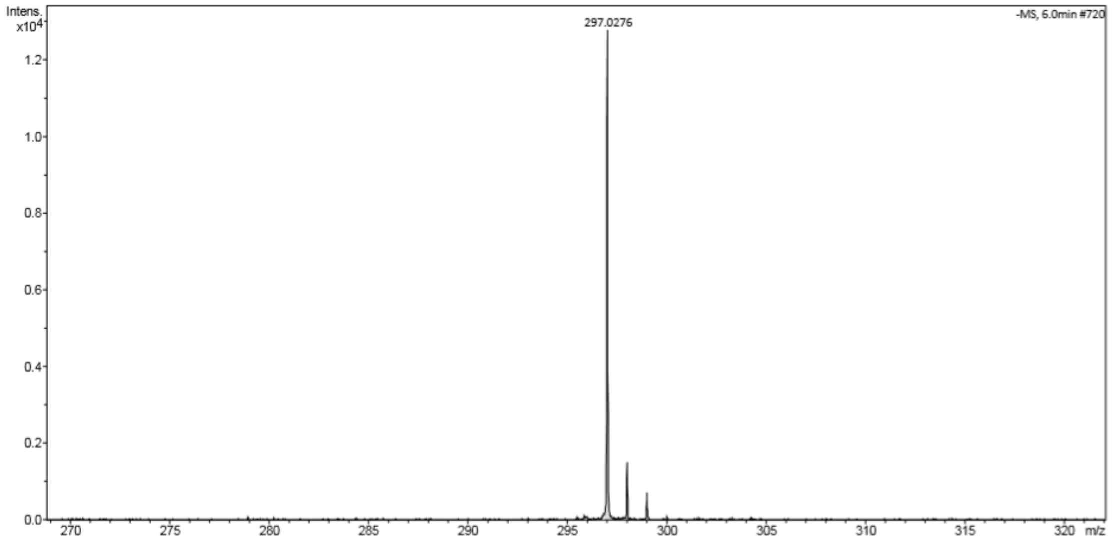

Figure S156 - A high-resolution mass spectrum (ESI$^-$) obtained for compound **10** in methanol.

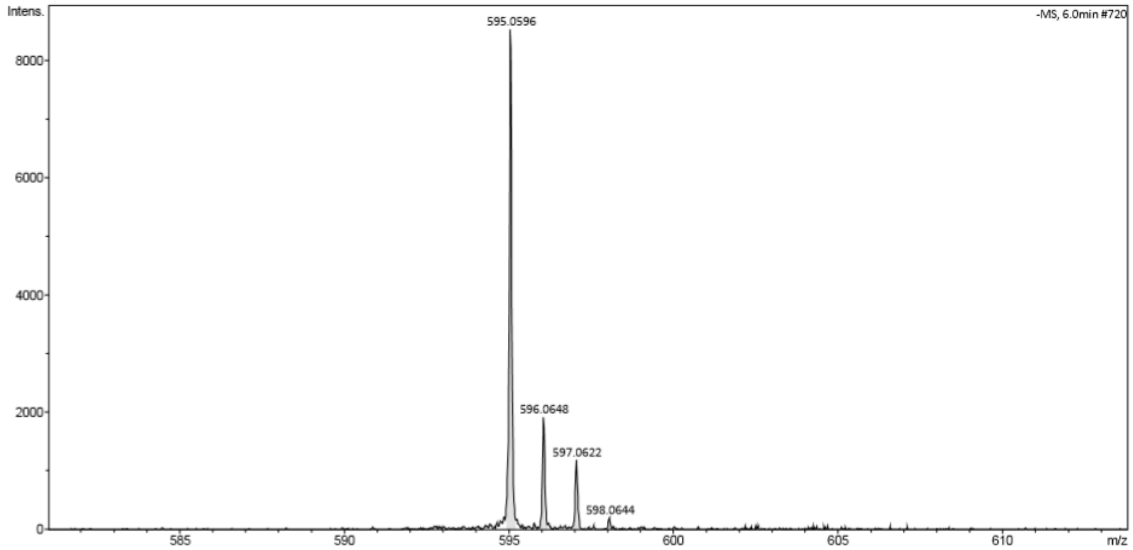

Figure S157 - A high-resolution mass spectrum (ESI$^-$) obtained for dimeric species of compound **10** in methanol, *m/z* [M + M + H$^+$]$^-$.

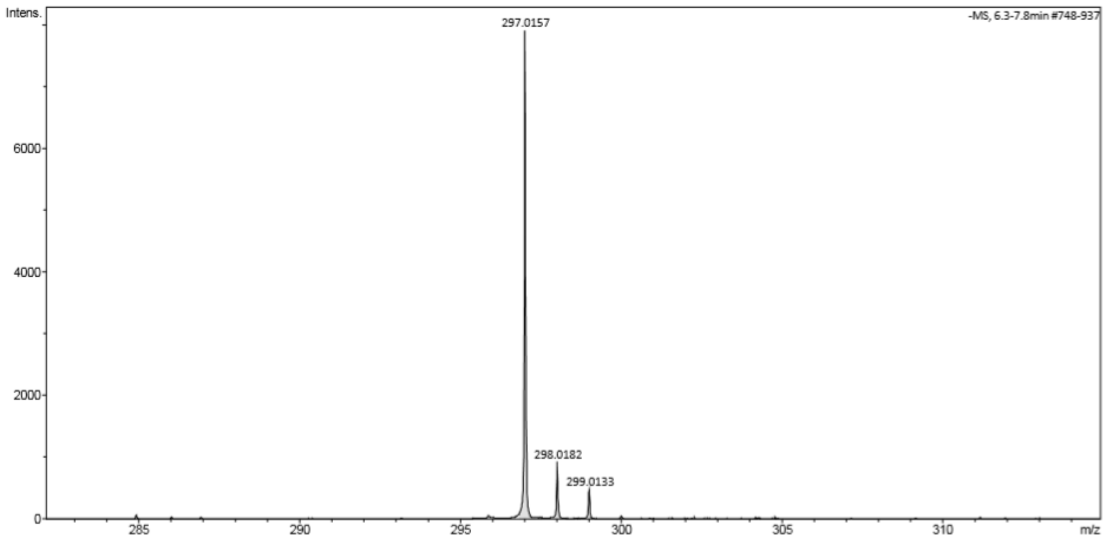

Figure S158 - A high-resolution mass spectrum (ESI$^-$) obtained for compound **11** in methanol.

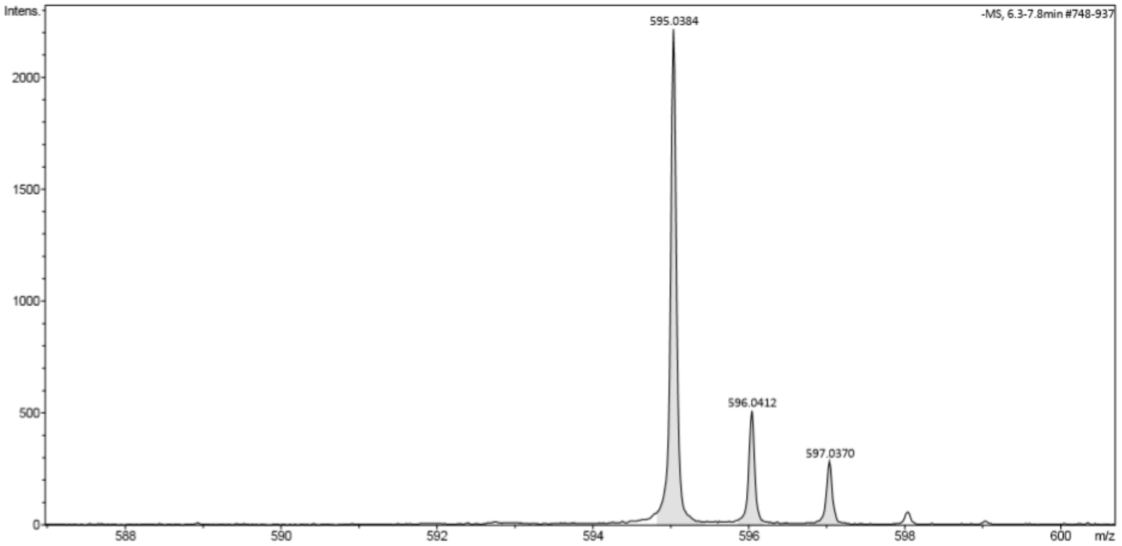

Figure S159 - A high-resolution mass spectrum (ESI$^-$) obtained for dimeric species of compound **11** in methanol, *m/z* [M + M + H$^+$]$^-$.

Overview

Table S12 – High resolution ESI$^-$ mass spectrometry theoretical and experimentally derived values.

	m/z [M]-		m/z [M + M + H]	
**Compound**	**Theoretical**	**Actual**	**Theoretical**	**Actual**
**3**	300.1024	300.1012	601.2048	601.2091
**10**	297.0162	297.0276	595.0324	595.0596
**11**	297.0162	297.0157	595.0324	595.0384

References

1 J. R. Hiscock, G. P. Bustone, B. Wilson, K. E. Belsey and L. R. Blackholly, *Soft Matter*, 2016, **12**, 4221–4228.

2 L. J. White, S. N. Tyuleva, B. Wilson, H. J. Shepherd, K. K. L. Ng, S. J. Holder, E. R. Clark and J. R. Hiscock, *Chem. - A Eur. J.*, 2018, **24**, 7761–7773.

3 L. J. White, N. J. Wells, L. R. Blackholly, H. J. Shepherd, B. Wilson, G. P. Bustone, T. J. Runacres and J. R. Hiscock, *Chem. Sci.*, 2017, **8**, 7620–7630.

4 C. A. Hunter, *Angew. Chemie Int. Ed.*, 2004, **43**, 5310–5324.

5 J. J. P. Stewart, *J. Mol. Model.*, 2007, **13**, 1173–1213.
