# Supplementary material for: Towards the Application of Supramolecular Self-Associating Amphiphiles as Next-Generation Delivery Vehicles
Source: Molecules. 2020 Sep 9;25(18):4126. doi: 10.3390/molecules25184126 (PMC7570936; doi:10.3390/molecules25184126)

checkCIF/PLATON report

Structure factors have been supplied for datablock(s) 2019jrh0005_res

THIS REPORT IS FOR GUIDANCE ONLY. IF USED AS PART OF A REVIEW PROCEDURE FOR PUBLICATION, IT SHOULD NOT REPLACE THE EXPERTISE OF AN EXPERIENCED CRYSTALLOGRAPHIC REFEREE.

No syntax errors found. CIF dictionary Interpreting this report

Datablock: 2019jrh0005_res

```
Bond precision:  C-C = 0.0061 A          Wavelength=1.54184

Cell:            a=5.7994(2)     b=29.6243(15)      c=15.3739(5)
                 alpha=90        beta=90.375(3)     gamma=90
Temperature:     150 K
```

	Calculated	Reported
Volume	2641.22(18)	2641.23(19)
Space group	P 21/c	P 1 21/c 1
Hall group	-P 2ybc	-P 2ybc
Moiety formula	C9 H8 F3 N2 O4 S, C16 H18 N3 S, O	C9 H8 F3 N2 O4 S, C16 H18 N3 S, O
Sum formula	C25 H26 F3 N5 O5 S2	C25 H26 F3 N5 O5 S2
Mr	597.63	597.63
Dx,g cm-3	1.503	1.503
Z	4	4
Mu (mm-1)	2.436	2.436
F000	1240.0	1240.0
F000’	1246.76	
h,k,lmax	6,35,18	6,35,18
Nref	4657	4655
Tmin,Tmax	0.849,0.932	0.782,1.000
Tmin’	0.637	

```
Correction method= # Reported T Limits: Tmin=0.782 Tmax=1.000
AbsCorr = GAUSSIAN

Data completeness= 1.000           Theta(max)= 66.579

R(reflections)= 0.0630( 3427)      wR2(reflections)= 0.1432( 4655)

S = 1.111                  Npar= 396
```

The following ALERTS were generated. Each ALERT has the format
test-name_ALERT_alert-type_alert-level.
Click on the hyperlinks for more details of the test.

Alert level C

```
CRYSC01_ALERT_1_C The word below has not been recognised as a standard
            identifier.
            dull
PLAT018_ALERT_1_C _diffrn_measured_fraction_theta_max .NE. *_full          ! Check
PLAT213_ALERT_2_C Atom F1             has ADP max/min Ratio .....        3.4 prolat
PLAT213_ALERT_2_C Atom F3A            has ADP max/min Ratio .....        3.9 prolat
PLAT220_ALERT_2_C NonSolvent Resd 1  F   Ueq(max) / Ueq(min) Range       3.1 Ratio
PLAT340_ALERT_3_C Low Bond Precision on  C-C Bonds ..............    0.00605 Ang.
PLAT430_ALERT_2_C Short Inter D...A Contact  O3       ..O5A      .      2.87 Ang.
                                                     x,y,z  =      1_555 Check
PLAT430_ALERT_2_C Short Inter D...A Contact  O4       ..O5A      .      2.85 Ang.
                                                  -1+x,y,z  =      1_455 Check
PLAT790_ALERT_4_C Centre of Gravity not Within Unit Cell: Resd.  #          1 Note
              C9 H8 F3 N2 O4 S
PLAT906_ALERT_3_C Large K Value in the Analysis of Variance ......      5.806 Check
PLAT934_ALERT_3_C Number of (Iobs-Icalc)/Sigma(W) > 10 Outliers ..          1 Check
```

Alert level G

```
PLAT007_ALERT_5_G Number of Unrefined Donor-H Atoms ..............          2 Report
PLAT083_ALERT_2_G SHELXL Second Parameter in WGHT  Unusually Large       5.40 Why ?
PLAT171_ALERT_4_G The CIF-Embedded .res File Contains EADP Records          1 Report
PLAT242_ALERT_2_G Low    'MainMol' Ueq as Compared to Neighbors of         C1 Check
PLAT300_ALERT_4_G Atom Site Occupancy of O5         Constrained at        0.5 Check
PLAT300_ALERT_4_G Atom Site Occupancy of O5A        Constrained at        0.5 Check
PLAT301_ALERT_3_G Main Residue  Disorder ..............(Resd  1  )        16% Note
PLAT302_ALERT_4_G Anion/Solvent/Minor-Residue Disorder (Resd  3  )       100% Note
PLAT302_ALERT_4_G Anion/Solvent/Minor-Residue Disorder (Resd  4  )       100% Note
PLAT304_ALERT_4_G Non-Integer Number of Atoms in ..... (Resd  3  )       0.50 Check
PLAT304_ALERT_4_G Non-Integer Number of Atoms in ..... (Resd  4  )       0.50 Check
PLAT311_ALERT_2_G Isolated Disordered Oxygen Atom (No H's ?) .....         O5 Check
PLAT311_ALERT_2_G Isolated Disordered Oxygen Atom (No H's ?) .....        O5A Check
PLAT790_ALERT_4_G Centre of Gravity not Within Unit Cell: Resd.  #          4 Note
              O
PLAT909_ALERT_3_G Percentage of I>2sig(I) Data at Theta(Max) Still        63% Note
PLAT910_ALERT_3_G Missing # of FCF Reflection(s) Below Theta(Min).          2 Note
PLAT978_ALERT_2_G Number C-C Bonds with Positive Residual Density.          1 Info
```

---

0 **ALERT level A** = Most likely a serious problem - resolve or explain
0 **ALERT level B** = A potentially serious problem, consider carefully
11 **ALERT level C** = Check. Ensure it is not caused by an omission or oversight
17 **ALERT level G** = General information/check it is not something unexpected

2 ALERT type 1 CIF construction/syntax error, inconsistent or missing data
10 ALERT type 2 Indicator that the structure model may be wrong or deficient
6 ALERT type 3 Indicator that the structure quality may be low
9 ALERT type 4 Improvement, methodology, query or suggestion
1 ALERT type 5 Informative message, check

It is advisable to attempt to resolve as many as possible of the alerts in all categories. Often the minor alerts point to easily fixed oversights, errors and omissions in your CIF or refinement strategy, so attention to these fine details can be worthwhile. In order to resolve some of the more serious problems it may be necessary to carry out additional measurements or structure refinements. However, the purpose of your study may justify the reported deviations and the more serious of these should normally be commented upon in the discussion or experimental section of a paper or in the "special_details" fields of the CIF. checkCIF was carefully designed to identify outliers and unusual parameters, but every test has its limitations and alerts that are not important in a particular case may appear. Conversely, the absence of alerts does not guarantee there are no aspects of the results needing attention. It is up to the individual to critically assess their own results and, if necessary, seek expert advice.

Publication of your CIF in IUCr journals

A basic structural check has been run on your CIF. These basic checks will be run on all CIFs submitted for publication in IUCr journals (*Acta Crystallographica*, *Journal of Applied Crystallography*, *Journal of Synchrotron Radiation*); however, if you intend to submit to *Acta Crystallographica Section C* or *E* or *IUCrData*, you should make sure that full publication checks are run on the final version of your CIF prior to submission.

Publication of your CIF in other journals

Please refer to the *Notes for Authors* of the relevant journal for any special instructions relating to CIF submission.

PLATON version of 16/04/2020; check.def file version of 09/03/2020

Datablock 2019jrh0005_res - ellipsoid plot

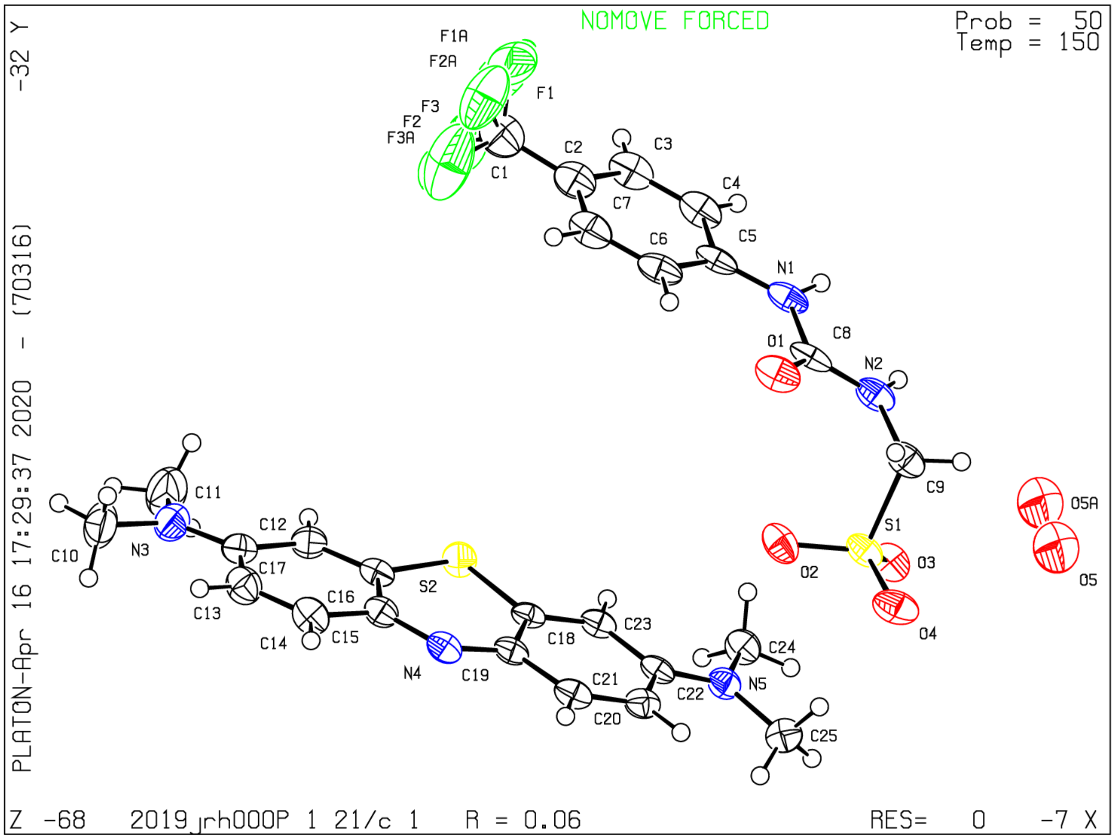

Supplement: Supplementary file 1 [file molecules-25-04126-s001.zip › supplimentary information/XRD data/2019jrh0005_res_cifreport.pdf]
