# Supplementary material for: Towards the Application of Supramolecular Self-Associating Amphiphiles as Next-Generation Delivery Vehicles
Source: Molecules. 2020 Sep 9;25(18):4126. doi: 10.3390/molecules25184126 (PMC7570936; doi:10.3390/molecules25184126)

checkCIF/PLATON report

Structure factors have been supplied for datablock(s) 2019jrh0028

THIS REPORT IS FOR GUIDANCE ONLY. IF USED AS PART OF A REVIEW PROCEDURE FOR PUBLICATION, IT SHOULD NOT REPLACE THE EXPERTISE OF AN EXPERIENCED CRYSTALLOGRAPHIC REFEREE.

No syntax errors found. CIF dictionary Interpreting this report

Datablock: 2019jrh0028

```
Bond precision:  C-C = 0.0017 A          Wavelength=1.54184

Cell:           a=16.6604(2)    b=17.1219(2)          c=22.4274(3)
                alpha=90        beta=110.7979(15)     gamma=90
Temperature:    100 K
```

	Calculated	Reported
Volume	5980.71(14)	5980.67(14)
Space group	I 2/a	I 1 2/a 1
Hall group	-I 2ya	-I 2ya
Moiety formula	C12 H18 N3 O4 S, C16 H36 N	C12 H18 N3 O4 S, C16 H36 N
Sum formula	C28 H54 N4 O4 S	C28 H54 N4 O4 S
Mr	542.81	542.81
Dx,g cm-3	1.206	1.206
Z	8	8
Mu (mm-1)	1.261	1.261
F000	2384.0	2384.0
F000’	2392.91	
h,k,lmax	20,21,27	20,21,27
Nref	5995	5901
Tmin,Tmax	0.874,0.904	0.794,1.000
Tmin’	0.866	

```
Correction method= # Reported T Limits: Tmin=0.794 Tmax=1.000
AbsCorr = GAUSSIAN

Data completeness= 0.984          Theta(max)= 73.093

R(reflections)= 0.0302( 5409)     wR2(reflections)= 0.0804( 5901)

S = 1.020                 Npar= 550
```

The following ALERTS were generated. Each ALERT has the format
test-name_ALERT_alert-type_alert-level.
Click on the hyperlinks for more details of the test.

Alert level G

```
PLAT910_ALERT_3_G Missing # of FCF Reflection(s) Below Theta(Min).          1 Note
PLAT912_ALERT_4_G Missing # of FCF Reflections Above STh/L=  0.600         93 Note
PLAT978_ALERT_2_G Number C-C Bonds with Positive Residual Density.         15 Info
```

```
   0 ALERT level A = Most likely a serious problem - resolve or explain
   0 ALERT level B = A potentially serious problem, consider carefully
   0 ALERT level C = Check. Ensure it is not caused by an omission or oversight
   3 ALERT level G = General information/check it is not something unexpected

   0 ALERT type 1 CIF construction/syntax error, inconsistent or missing data
   1 ALERT type 2 Indicator that the structure model may be wrong or deficient
   1 ALERT type 3 Indicator that the structure quality may be low
   1 ALERT type 4 Improvement, methodology, query or suggestion
   0 ALERT type 5 Informative message, check
```

It is advisable to attempt to resolve as many as possible of the alerts in all categories. Often the minor alerts point to easily fixed oversights, errors and omissions in your CIF or refinement strategy, so attention to these fine details can be worthwhile. In order to resolve some of the more serious problems it may be necessary to carry out additional measurements or structure refinements. However, the purpose of your study may justify the reported deviations and the more serious of these should normally be commented upon in the discussion or experimental section of a paper or in the "special_details" fields of the CIF. checkCIF was carefully designed to identify outliers and unusual parameters, but every test has its limitations and alerts that are not important in a particular case may appear. Conversely, the absence of alerts does not guarantee there are no aspects of the results needing attention. It is up to the individual to critically assess their own results and, if necessary, seek expert advice.

Publication of your CIF in IUCr journals

A basic structural check has been run on your CIF. These basic checks will be run on all CIFs submitted for publication in IUCr journals (*Acta Crystallographica*, *Journal of Applied Crystallography*, *Journal of Synchrotron Radiation*); however, if you intend to submit to *Acta Crystallographica Section C* or *E* or *IUCrData*, you should make sure that full publication checks are run on the final version of your CIF prior to submission.

Publication of your CIF in other journals

Please refer to the *Notes for Authors* of the relevant journal for any special instructions relating to CIF submission.

PLATON version of 18/03/2020; check.def file version of 09/03/2020

Datablock 2019jrh0028 - ellipsoid plot

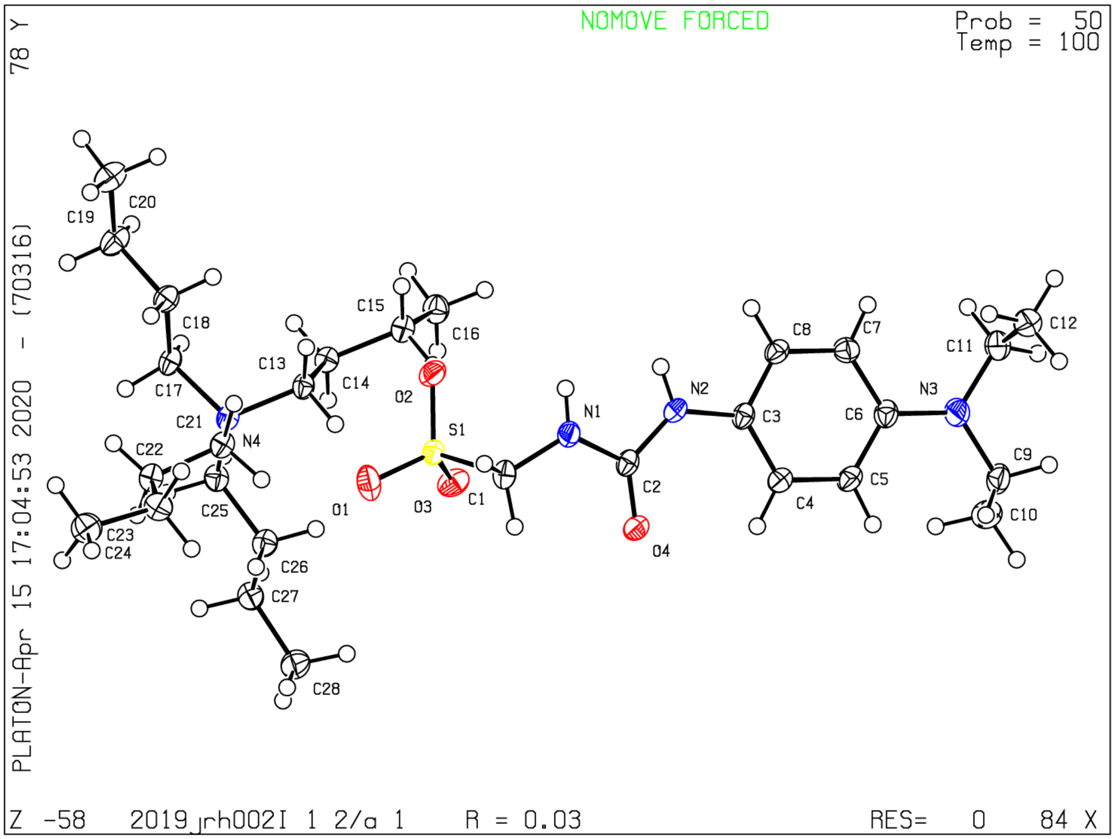

Supplement: Supplementary file 1 [file molecules-25-04126-s001.zip › supplimentary information/XRD data/2019jrh0028_cifreport.pdf]
